# Cage escape governs photoredox reaction rates and quantum yields

**DOI:** 10.1038/s41557-024-01482-4

**Published:** 2024-03-18

**Authors:** Cui Wang, Han Li, Tobias H. Bürgin, Oliver S. Wenger

**Affiliations:** 1 Department of Chemistry, University of Basel, Basel, Switzerland; 2 Present Address: Department of Biology and Chemistry, Osnabrück University, Osnabrück, Germany

**Keywords:** Photocatalysis, Coordination chemistry, Optical spectroscopy, Electron transfer, Catalytic mechanisms

## Abstract

Photoredox catalysis relies on light-induced electron transfer leading to a radical pair comprising an oxidized donor and a reduced acceptor in a solvent cage. For productive onward reaction to occur, the oxidized donor and the reduced acceptor must escape from that solvent cage before they undergo spontaneous reverse electron transfer. Here we show the decisive role that cage escape plays in three benchmark photocatalytic reactions, namely, an aerobic hydroxylation, a reductive debromination and an aza-Henry reaction. Using ruthenium(II)- and chromium(III)-based photocatalysts, which provide inherently different cage escape quantum yields, we determined quantitative correlations between the rates of photoredox product formation and the cage escape quantum yields. These findings can be largely rationalized within the framework of Marcus theory for electron transfer.

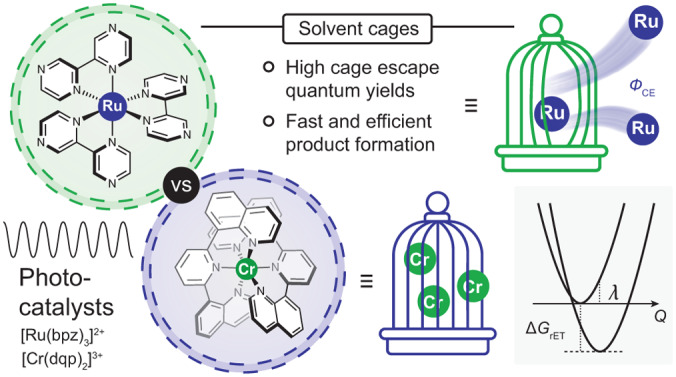

## Abstract (web summary)

The spontaneous recombination of photogenerated radicals surrounded by solvent molecules is an important energy-wasting elementary step in photoredox reactions. Now the decisive role that cage escape plays in these reactions is shown in three benchmark photocatalytic reactions, with quantitative correlations observed between photoredox product formation rates and cage escape quantum yields.

## Main

**Fig. 1:**
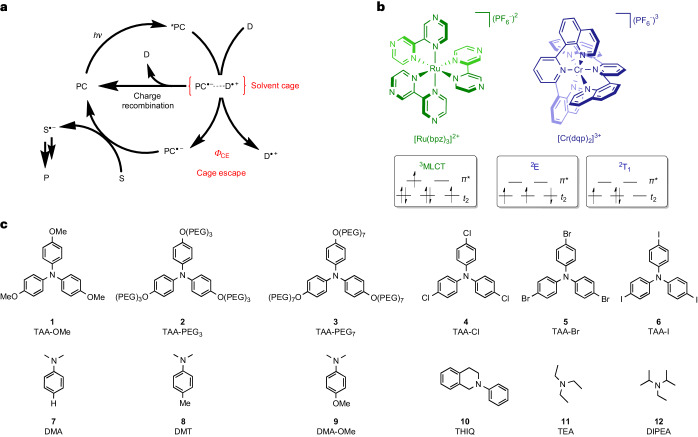
Photoinduced electron transfer between metal complexes and tertiary amines. **a**, Catalytic cycle of a photocatalyst (PC) reacting with an electron donor (D) via a so-called reductive quenching mechanism (oxidative quenching mechanisms are also common but are not considered here)^7^. Following excitation of the photocatalyst, photoinduced electron transfer leads to the reduced photocatalyst and the oxidized donor embedded in a solvent cage. Escape from this solvent cage competes with unproductive thermal reverse electron transfer (charge recombination). Only successful cage escape can lead to productive photoredox chemistry, here electron transfer to the substrate (S), which reacts onwards to the desired product (P) in subsequent (light-independent) elementary reaction steps. **b**, Molecular structures of the investigated photocatalysts [Ru(bpz)_3_]^2+^ and [Cr(dqp)_2_]^3+^ showing the pertinent microstates of the photoactive ^3^MLCT excited state and the ^2^E and ^2^T_1_ spin-flip excited states, respectively. **c**, Molecular structures of the investigated electron donors **1**–**12**. PEG, polyethylene glycol.

Photocatalysis has become a powerful method in chemistry^1–6^, but some key mechanistic aspects remain underexplored and poorly understood^7–10^. Many photoredox studies have monitored the reactivity of electronically excited states by luminescence quenching experiments and equated the disappearance of luminescent excited states to the formation of photochemical products, but this simplification is not generally valid^11,12^. Diffusion processes can bring together an electronically excited photocatalyst (*PC) and an electron donor in a so-called encounter complex, and then photoinduced electron transfer can quench the photocatalyst’s luminescence. However, the primary quenching products, comprising the reduced photocatalyst (PC^**·**^^−^) and the oxidized donor (D^**·**^^+^), are embedded in a solvent cage (Fig. 1a), from which they must escape for product formation to become possible. Thermal reverse electron transfer from PC^**·**^^−^ to D^**·**^^+^ leading to charge recombination can spontaneously occur within the solvent cage^13,14^, in which case no product is formed even though luminescence quenching is detected. The importance of cage escape has long been overlooked in photoredox catalysis, but is now beginning to be recognized^11,12,15–17^.

Cage escape quantum yields are affected by many different factors, including the driving force, the reorganization energy and the electronic coupling associated with photoinduced electron transfer and thermal in-cage reverse electron transfer^16,18,19^, spin and heavy-atom effects^11,13,14,20–22^, solvent polarity and viscosity^11,20,23–25^, molecular size effects^26^, ionic strength and ion-pairing effects^13,24,25,27^, electrostatic interactions^28^ and temperature^24,25,29^. Despite numerous fundamental investigations, cage escape quantum yields have remained extremely difficult to predict. In this study, we found that the [Ru(bpz)_3_]^2+^ (bpz, 2,2′-bipyrazine) and [Cr(dqp)_2_]^3+^ (dqp, 2,6-di(quinolin-8-yl)pyridine) photocatalysts (Fig. 1b) show a similar driving-force dependence for light-induced electron transfer with 12 different donors (Fig. 1c), but the cage escape quantum yields are consistently much higher with the Ru^II^ complex than with the Cr^III^ compound. We also determined quantitative correlations between the cage escape quantum yields and the product formation rates in three different photoredox reactions. Each of these three model reactions relies on a multitude of individual elementary steps, and thus the finding that the overall photoredox product formation rates are governed in large part by cage escape seems remarkable. Two key findings emerge from our work: (1) photoredox reaction product formation rates and quantum yields correlate with the cage escape quantum yield (*Ф*_CE_) and (2) the photocatalyst seems to govern the achievable magnitude of *Ф*_CE_ by dictating the rate of unwanted charge recombination within the solvent cage.

## Results and discussion

### Photoinduced electron transfer of Ru and Cr complexes

The electron donors shown in Fig. 1c were chosen to explore the influence of the following factors on cage escape: (1) the variation in the driving force for photoinduced electron transfer (Δ*G*_ET_), (2) the effect of donor size (TAA-OMe, TAA-PEG_3_ and TAA-PEG_7_), (3) the influence of heavy atoms (TAA-Cl, TAA-Br and TAA-I), (4) structural differences in the aromatic amines (triarylamines (TAAs) versus *N*,*N*-dimethylanilines) and (5) aromatic amines versus aliphatic amines (2-phenyl-1,2,3,4-tetrahydroisoquinoline (THIQ), triethylamine (TEA) and *N*,*N*-diisopropylethylamine (DIPEA)).

**Fig. 2:**
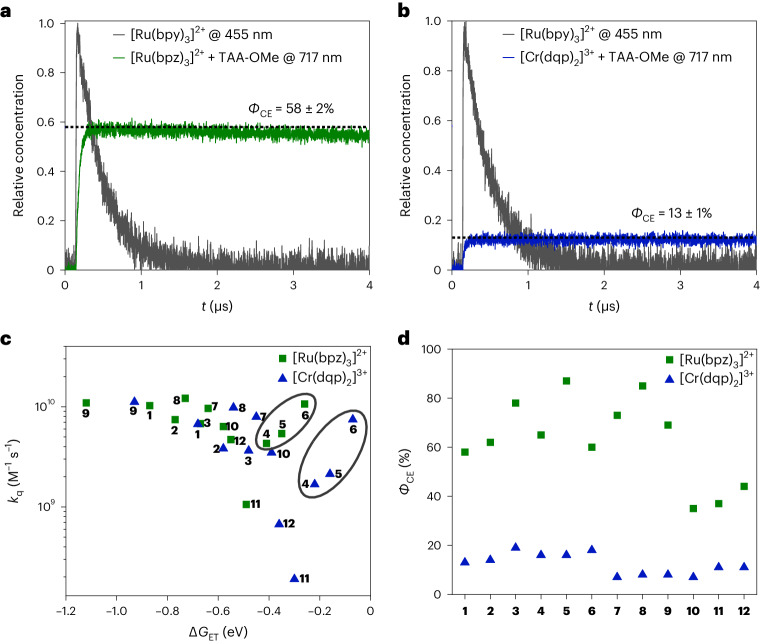
Cage escape quantum yields and excited-state quenching kinetics. **a**, Transient absorption decays of the reference complex [Ru(bpy)_3_]^2+^ (12 µM in aerated H_2_O) at 455 nm and the [Ru(bpz)_3_]^2+^ (12 µM)–TAA-OMe (2 mM) pair in aerated CH_3_CN at 293 K at 717 nm under 422 nm excitation. Both solutions have identical absorbance at 422 nm (Supplementary Fig. 55a). The concentrations of the ^3^MLCT-excited [Ru(bpy)_3_]^2+^ and TAA-OMe^**·**^^+^ formed in this experiment were 3.14 and 1.82 ± 0.06 μM, respectively. **b**, Transient absorption decays of the reference complex [Ru(bpy)_3_]^2+^ (12 µM in aerated H_2_O) at 455 nm and the [Cr(dqp)_2_]^3+^ (30 µM)–TAA-OMe (2 mM) pair in aerated CH_3_CN at 293 K at 717 nm under 416 nm excitation. Both solutions have identical absorbance at 416 nm (Supplementary Fig. 56a). The concentrations of the ^3^MLCT-excited [Ru(bpy)_3_]^2+^ and TAA-OMe^**·**^^+^ were 1.56 and 0.20 ± 0.02 μM, respectively. In **a** and **b**, the relative concentrations of the photoproducts were derived from the measured ΔOD values at 455 nm for the reference (^3^MLCT-excited [Ru(bpy)_3_]^2+^) and at 717 nm for the donor–acceptor pairs (TAA-OMe^**·**^^+^), as well as the respective Δ*ε* values at the relevant observation wavelengths (Δ*ε*_455_ = −10,100 M^−1^ cm^−1^ for [Ru(bpy)_3_]^2+^ (ref. ^33^) and Δ*ε*_717_ = 32,600 ± 300 M^−1^ cm^−1^ for TAA-OMe^**·**^^+^). This analysis yielded *Ф*_CE_ values of 58 ± 2% for the [Ru(bpz)_3_]^2+^–TAA-OMe pair and 13 ± 1% for the [Cr(dqp)_2_]^3+^–TAA-OMe pair in CH_3_CN at room temperature, as marked by the dashed horizontal lines. **c**, Quenching rate constants (*k*_q_) for photoinduced electron transfer from the individual donors **1**–**12** to [Ru(bpz)_3_]^2+^ and [Cr(dqp)_2_]^3+^ plotted as a function of the reaction free energy (Δ*G*_ET_). The halide-substituted donors are marked by the ellipsoids. **d**, Cage escape quantum yields (*Ф*_CE_) obtained with [Ru(bpz)_3_]^2+^ and [Cr(dqp)_2_]^3+^ for the electron donors **1**–**12**. Source data

[Ru(bpz)_3_]^2+^ and [Cr(dqp)_2_]^3+^ have similar excited-state reduction potentials (1.45 and 1.26 V versus the saturated calomel electrode, respectively)^30^. All 12 donors quenched the luminescent excited states of the two complexes with rate constants (*k*_q_) in the range of 10^8^–10^10^ M^−1^ s^−1^ (Supplementary Figs. 15–40), revealing very similar driving-force dependence in both cases (Fig. 2c), in line with previous studies^30–32^. Deviations from this Rehm–Weller behaviour were observed for the halide-substituted TAA electron donors **4**–**6** (highlighted in the ellipsoids in Fig. 2c). Their heavy atoms accelerate photoinduced electron transfer^22^, despite the decreasing driving force (−Δ*G*_ET_) along the series TAA-Cl > TAA-Br > TAA-I (Supplementary Section 7.13.3). The driving forces for the photoinduced electron transfer and in-cage thermal charge recombination for irreversible donors were estimated according to the oxidation peak potentials because standard redox potentials were not available in these cases.

### Trends in cage escape quantum yields

The cage escape quantum yields for the electron transfer photoproducts formed after the excitation of [Ru(bpz)_3_]^2+^ or [Cr(dqp)_2_]^3+^ in the presence of the donors **1**–**12** (Fig. 1c) in aerated acetonitrile were determined by relative actinometry using laser flash photolysis (Supplementary Sections 6 and 7). The basic principle of this method is to compare the concentrations of the photoproducts formed after pulsed excitation of a sample and a reference solution. The reference, here an aqueous aerated solution of [Ru(bpy)_3_]Cl_2_ (bpy, 2,2′-bipyridine), has a known quantum yield for the formation of its photoproduct(s) and serves to determine the number of photons absorbed by the reference. By adjusting the photocatalyst concentration in the main sample and in the reference solution, an equal absorbance at the excitation wavelength and an equal concentration of the excited photocatalysts are ensured for both solutions (Supplementary Section 7). Measurement of the concentration of the photoproducts formed in the main sample then permits the determination of the unknown quantum yield.

The excitation of [Ru(bpy)_3_]^2+^ at 422 nm causes a depletion of its diagnostic singlet metal-to-ligand charge transfer (^1^MLCT) ground-state absorption band, manifesting in a known change in its molar extinction coefficient (Δ*ε*_455_ = −10,100 M^−1^ cm^−1^ at 455 nm, ref. ^33^). This bleaching reflects the quantitative formation of a ^3^MLCT excited state, which decays on a microsecond timescale (Fig. 2a,b). For ease of comparison with the main samples under test, the experimentally observed changes in the optical density (ΔOD) of this reference at 455 nm were normalized to an initial value of 1.0. Concentration-adjusted samples containing the [Ru(bpz)_3_]^2+^–TAA-OMe and [Cr(dqp)_2_]^3+^–TAA-OMe donor–acceptor pairs were then excited under identical conditions. The donors were present in large excess relative to the metal complexes (millimolar versus micromolar concentrations) to ensure rapid and maximally efficient (quantitative) photoinduced electron transfer, yet selective excitation of the metal complexes remained possible in all cases due to the negligible extinction coefficient of the donors at the relevant wavelengths. The TAA-OMe^**·**^^+^ oxidation product was readily detected by its characteristic absorption in the red spectral range with a change in the molar extinction coefficient (Δ*ε*_717_) of 32,600 ± 300 M^−1^ cm^−1^ at 717 nm (Supplementary Fig. 44). The transient absorption decay curves for the two donor–acceptor pairs are shown in Fig. 2a,b, respectively. Cage escape quantum yields (*Ф*_CE_) of 58 ± 2% for the [Ru(bpz)_3_]^2+^–TAA-OMe pair and 13 ± 1% for the [Cr(dqp)_2_]^3+^–TAA-OMe pair were determined from the relative intensities reached immediately after excitation. The decays of the respective transients reflect reverse electron transfer from the reduced metal complexes to TAA-OMe^**·**^^+^ after successful cage escape.

The same method was applied to all of the donor*–*acceptor pairs, which required the measurement of the extinction-calibrated difference spectra for the oxidized and neutral forms of the individual donors, as well as for the native and reduced forms of the two metal complexes (Supplementary Sections 4, 6 and 7). The resulting *Ф*_CE_ values obtained with [Ru(bpz)_3_]^2+^ and [Cr(dqp)_2_]^3+^ for the 12 electron donors are presented in Fig. 2d. All of the TAA-based donors underwent reversible electron transfer, whereas the other electron donors were irreversible donors due to deprotonation after the initial one-electron oxidation^34^. In principle, such proton-coupled electron transfer reactions could artificially enhance the cage escape quantum yields obtained for the two photosensitizers with the non-TAA donors, potentially complicating the direct comparison between TAA and non-TAA donors.

The following findings emerge from these experiments. (1) For all of the electron donors explored here, the *Ф*_CE_ values obtained with [Ru(bpz)_3_]^2+^ are substantially higher than those obtained with [Cr(dqp)_2_]^3+^. (2) There is no clear evidence of a heavy-atom effect on cage escape (Supplementary Section 7.13.3). (3) Increasing donor size in the series TAA-OMe < TAA-PEG_3_ < TAA-PEG_7_ leads to increasing *Ф*_CE_ values, which could be due to better distancing of the donor–acceptor pairs (Supplementary Section 7.13.4)^26^. (4) No substantial change in cage escape quantum yield was observed on variation of the electron donor concentration (Supplementary Fig. 71). (5) The presence of oxygen does not affect the cage escape quantum yields (Supplementary Figs. 55–57).

### Different cage escape for Ru and Cr complexes

[Ru(bpz)_3_]^2+^ and [Cr(dqp)_2_]^3+^ have fundamentally different photoactive excited states (Fig. 1b) as a result of their different valence electron configurations (low-spin *d*^6^ versus *d*^3^)^30,35^. The ^3^MLCT excited state of [Ru(bpz)_3_]^2+^ is predisposed to charge transfer, yet the ^2^E and ^2^T_1_ spin-flip excited states of [Cr(dqp)_2_]^3+^ promote photoinduced electron transfer similarly well according to the data in Fig. 2c. However, due to substantial in-cage charge recombination (Fig. 1a), much lower cage escape quantum yields were systematically obtained with the Cr^III^ complex compared with the Ru^II^ complex (Fig. 2d). At first glance, therefore, it seems plausible that the differences in the electronic structures of [Ru(bpz)_3_]^2+^ and [Cr(dqp)_2_]^3+^ are the cause of the very different cage escape quantum yields. We initially considered different spin-correlated radical pairs emerging from the ^3^MLCT and ^2^E/^2^T_1_ excited states and spin effects for in-cage charge recombination as a possible reason for the systematic differences between the Ru^II^ and Cr^III^ complexes (Supplementary Section 7.13.2), but the data currently available do not permit corroboration of this hypothesis. Instead, it seems that Marcus theory is able to account for the observed behaviour, making superfluous the involvement of spin and other effects as possible explanations. The cage escape quantum yield (*Ф*_CE_) for both complexes is governed by the relative magnitudes of the cage escape rate (*k*_CE_) and in-cage reverse electron transfer (*k*_rET_; equation (1a,b)).

1a $${\phi }_{{\rm{CE}}}\left({\rm{Ru}}\right)=\frac{{k}_{{\rm{CE}}}\left({\rm{Ru}}\right)}{{k}_{{\rm{CE}}}\left({\rm{Ru}}\right)+{k}_{{\rm{rET}}}\left({\rm{Ru}}\right)}$$

1b $${\phi }_{{\rm{CE}}}\left({\rm{Cr}}\right)=\frac{{k}_{{\rm{CE}}}\left({\rm{Cr}}\right)}{{k}_{{\rm{CE}}}\left({\rm{Cr}}\right)+{k}_{{\rm{rET}}}\left({\rm{Cr}}\right)}$$

Assuming equal cage escape rate constants for the Ru^II^ and Cr^III^ complexes (*k*_CE_(Ru) = *k*_CE_(Cr)), the ratio of rate constants for in-cage reverse electron transfer can be formulated as shown in equation (2):

2 $$\frac{{k}_{{\rm{rET}},\exp .}\left({\rm{Ru}}\right)}{{k}_{{\rm{rET}},\exp .}\left({\rm{Cr}}\right)}=\frac{\frac{1}{{\phi }_{{\rm{CE}}}\left({\rm{Ru}}\right)}-1}{\frac{1}{{\phi }_{{\rm{CE}}}\left({\rm{Cr}}\right)}-1}$$

Thus, under the assumption that PC^**·**^^−^ and D^**·**^^+^ escape the solvent cage equally quickly regardless of whether PC^**·**^^−^ corresponds to [Ru(bpz)_3_]^+^ or [Cr(dqp)_2_]^2+^, the experimentally determined cage escape quantum yields can be used to estimate the ratio of in-cage reverse electron transfer for the Ru^II^ and Cr^III^ complexes for any given electron donor D (equation (2)). Based on the data shown in Fig. 2d, the experimentally determined ratios *k*_rET,exp._(Ru)/*k*_rET,exp._(Cr) vary between 0.015 and 0.21 (Supplementary Table 4).

Within the framework of semi-classical Marcus theory (equation (3)), the rate constants for (in-cage) reverse electron transfer (*k*_rET,calc._) can be expressed as a function of the electronic coupling (*H*_AB_) between the reduced photocatalyst (PC^**·**^^−^) and the oxidized donor (D^**·**^^+^), the reaction free energy (Δ*G*_rET_) for reverse electron transfer from PC^**·**^^−^ to D^**·**^^+^ and the reorganization energy (*λ*) accompanying that reaction. Equation (3) describes the ratio of the reverse electron transfer rate constants for the Ru^II^ and Cr^III^ complexes, where the individual parameters (*H*_AB_, Δ*G*_rET_ and *λ*) can be different depending on the photocatalyst–donor pair considered.

3 $$\frac{{k}_{{\rm{rET}},{\rm{calc}}.}\left({\rm{Ru}}\right)}{{k}_{{\rm{rET}},{\rm{calc}}.}\left({\rm{Cr}}\right)}=\frac{{\left[{H}_{{\rm{AB}}}({\rm{Ru}})\right]}^{2}{\rm{\times }}\exp \left[-\frac{{\left({\Delta G}_{{\rm{rET}}}\left({\rm{Ru}}\right)+\lambda \right)}^{2}}{{4\lambda k}_{{\rm{B}}}{\rm{T}}}\right]}{{\left[{H}_{{\rm{AB}}}({\rm{Cr}})\right]}^{2}{\rm{\times }}\exp \left[-\frac{{\left({\Delta G}_{{\rm{rET}}}\left({\rm{Cr}}\right)+\lambda \right)}^{2}}{{4\lambda k}_{{\rm{B}}}{\rm{T}}}\right]}$$

**Fig. 3:**
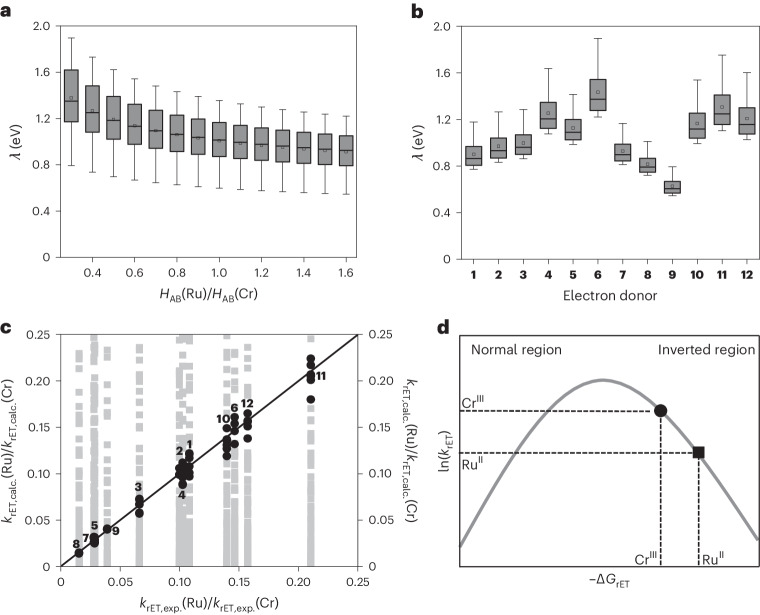
Screening of in-cage reverse electron transfer parameters. **a**, Reorganization energies for given ratios of electronic couplings able to produce *k*_rET,calc._(Ru)/*k*_rET,calc._(Cr) ratios (equation (3)) in line with the experimental *k*_rET,exp._(Ru)/*k*_rET,exp._(Cr) ratios derived from equation (2). The distribution here is shown for specific coupling ratios over the applied donors. **b**, Reorganization energies found for the individual donors based on the analysis in **a** (Supplementary Table 3). The distribution here is shown for specific donors over the considered coupling ratios. In **a**, the data size (*n*) for each distribution is 12 (for 12 applied donors) and in **b**, the data size is 14 (*H*_AB_ ratios from 0.3 to 1.6 eV in increments of 0.1 eV). The grey boxes in **a** and **b** contain the middle 50% of data, the so-called interquartile range (IQR). The whisker boundaries are drawn at the most extreme data point that is no more than ±1.5IQR from the top and bottom edges of the box^53^. The horizontal black lines in the boxes mark the median values, and the open squares represent the average values. **c**, Correlation between the calculated and experimental ratios of the rate constants for in-cage reverse electron transfer. In total, 2,184 combinations of *H*_AB_(Ru)/*H*_AB_(Cr) and *λ* (Supplementary Table 5) were considered (grey squares, right-hand *y* axis), of which 63 (Supplementary Table 4) provided agreement between calculation and experiment within a deviation of 15% (black circles, left-hand *y* axis). The numbers shown in **c** correspond to the donors **1**–**12**. **d**, Generic Marcus parabola illustrating the dependence of the rate constant for in-cage reverse electron transfer (*k*_rET_) on its driving force (Δ*G*_rET_). For a given electron donor, in-cage reverse electron transfer with the reduced Ru^II^ complex (black square) occurs more deeply in the inverted regime and thus is slower than in-cage reverse electron transfer with the reduced Cr^III^ complex (black circle). Source data

All of the relevant Δ*G*_rET_ values are known (Supplementary Table 1), but the *H*_AB_ and *λ* values are unknown. Equation (3) can be used to determine what combinations of *H*_AB_(Ru)/*H*_AB_(Cr) and *λ* make the ratio of calculated (in-cage) reverse electron transfer rate constants *k*_rET,calc._(Ru)/*k*_rET,calc._(Cr) equal to the corresponding experimental ratio determined with equation (2). The reorganization energy for in-cage reverse electron transfer depends on the individual photocatalyst–donor pair; however, in our analysis, the reorganization energy with different donors was allowed to vary, but for a given electron donor, identical *λ* values were assumed for both the Ru^II^ and Cr^III^ complexes. Varying the *H*_AB_(Ru)/*H*_AB_(Cr) ratio between 0.3 and 1.6 (in increments of 0.1) led to the distribution of *λ* values shown in Fig. 3a (Supplementary Table 3). For *H*_AB_(Ru)/*H*_AB_(Cr) ratios above 0.8, plausible *λ* values of ~1 eV account for the experimentally observed differences in the cage escape quantum yields. Figure 3b shows the distribution of *λ* values collected in Fig. 3a for the individual donors. The key finding is that for any given *H*_AB_(Ru)/*H*_AB_(Cr) ratio between 0.3 and 1.6, a sizeable fraction (79–86%) of the calculated *λ* values fall within a range of ±15% of the median reorganization energy obtained for a given donor (Supplementary Table 3). Analogous calculations were performed by considering a plausible range of *λ* values and solving for the *H*_AB_ ratio (Supplementary Fig. 83b). Broader screening of combinations of the *H*_AB_(Ru)/*H*_AB_(Cr) ratios (in increments of 0.1 between 0.3 and 1.6) and *λ* values (in increments of 0.1 eV between 0.4 and 1.6 eV) revealed that 63 out of 2,184 combinations are able to emulate the experimentally derived *k*_rET,exp._(Ru)/*k*_rET,exp._(Cr) ratios with a deviation of 15% or less (Supplementary Tables 4 and 5 and Supplementary Fig. 84). Figure 3c shows the results of screening in the same range as the experimentally obtained *k*_rET,exp._(Ru)/*k*_rET,exp._(Cr) ratios, showing the correlation between the calculated and experimental ratios of the rate constants for in-cage reverse electron transfer for 63 of the combinations.

The modulations of the *H*_AB_ ratios and *λ* values were to some extent arbitrary, and this analysis was unable to provide unambiguous insights into trends in the electronic couplings and reorganization energies. This is because the identified *H*_AB_(Ru)/*H*_AB_(Cr) and *λ* combinations were not necessarily mathematically unique solutions, and because other factors could vary, for example, the cage escape rate constants *k*_CE_(Ru) and *k*_CE_(Cr) in equation (1a,b). Nonetheless, some of the differences in the reorganization energies calculated for the various donors studied seem to make chemical sense. For example, the positive charge in the radical cation forms of the aliphatic donors **10**–**12** is probably not delocalized much further than the nitrogen and proximal carbon atoms, leading to relatively small ionic radii and large reorganization energies, whereas the cationic charge in the TAAs **1**–**6** and anilines **7**–**9** is delocalized into the aromatic rings, increasing the ionic radius and resulting in smaller reorganization energies (Fig. 3b)^26^. The increase in *λ* along the series **1**–**3** could be due to an increase in the degree of solvation^36^. The quintessential point of this analysis is that reasonable values for *H*_AB_ and *λ* can be fed into semi-classical Marcus theory and quantitatively explain the difference between the cage escape quantum yields observed with [Ru(bpz)_3_]^2+^ and [Cr(dqp)_2_]^3+^. Other possible explanations for the different cage escape quantum yields, while not fully discarded at this point, are therefore currently pushed into the background. Within this overall framework, for any given electron donor, in-cage reverse electron transfer with the Ru^II^ complex occurs ~0.3 eV more deeply in the Marcus inverted regime than with the Cr^III^ complex, making in-cage charge recombination slower and cage escape quantum yields higher for the Ru^II^ complex (Fig. 3d).

### Correlation of photocatalytic performance with cage escape

**Fig. 4:**
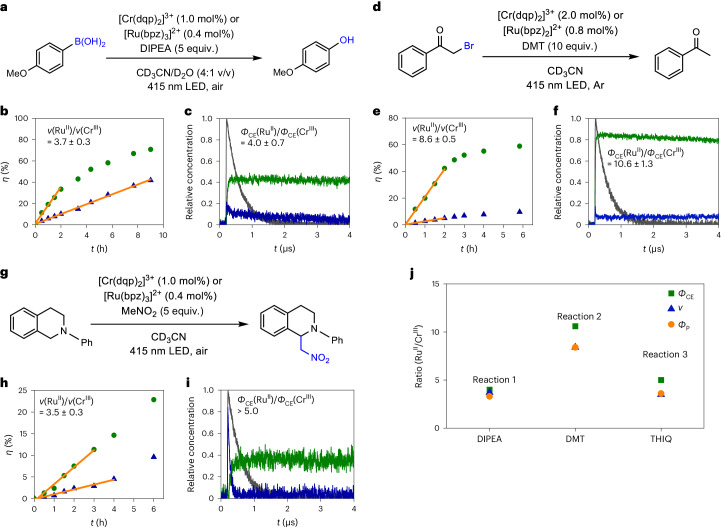
Photoredox reactions performed with Ru and Cr catalysts. **a**,**d**,**g**, Aerobic hydroxylation of 4-methoxyphenylboronic acid (**a**), reductive debromination of 2-bromoacetophenone (**d**) and aza-Henry reaction (**g**). The concentrations of [Ru(bpz)_3_]^2+^ and [Cr(dqp)_2_]^3+^ were adjusted to ensure equal absorbance at the irradiation wavelength of 415 nm. **b**,**e**,**h**, The product yields (*η*) of 4-methoxyphenol (**b**), acetophenone (**e**) and THIQ-MeNO_2_ (**h**) as a function of irradiation time for [Ru(bpz)_3_]^2+^ (green circles) and [Cr(dqp)_2_]^3+^ (blue triangles). Linear regression fits (orange lines) to the data points over the initial irradiation period provide the initial product formation rates *ν*(Ru^II^) and *ν*(Cr^III^). **c**,**f**,**i**, Cage escape quantum yields (*Ф*_CE_) obtained with the [Ru(bpy)_3_]^2+^ reference (dark-grey traces), [Ru(bpz)_3_]^2+^ (green traces) and [Cr(dqp)_2_]^3+^ (dark-blue traces) and the electron donors DIPEA (10 mM) (**c**), DMT (10 mM) (**f**) and THIQ (2 mM) (**i**), used in the photoredox reactions in **a**, **d** and **g**, respectively. For donor THIQ, only an upper limit could be determined for *Ф*_CE_ with [Cr(dqp)_2_]^3+^ (Supplementary Section 7.10). **j**, Experimentally determined ratios of the cage escape quantum yield (*Ф*_CE_), the initial product formation rate (*ν*) and the initial product formation quantum yield (*Ф*_P_) for [Ru(bpz)_3_]^2+^ and [Cr(dqp)_2_]^3+^ in the three photochemical reactions in **a**, **d** and **g** using DIPEA, DMT and THIQ, respectively, as electron donors. Source data

As [Ru(bpz)_3_]^2+^ and [Cr(dqp)_2_]^3+^ show similar photoinduced electron transfer behaviour (Fig. 2c) but systematically different cage escape quantum yields (Fig. 2d), using these two photocatalysts permitted investigation of how photoredox reactions are affected by cage escape. To address this fundamental issue, three different photochemical reactions involving three distinct electron donors were investigated: (1) the photocatalytic aerobic hydroxylation of 4-methoxyphenylboronic acid using DIPEA (Fig. 4a and Supplementary Section 8.1), (2) the photocatalytic reductive debromination of 2-bromoacetophenone with *N*,*N*-dimethyl-*p*-toluidine (DMT; Fig. 4d and Supplementary Section 8.2) and (3) a photocatalytic aza-Henry reaction, where THIQ acts both as the electron donor and substrate (Fig. 4g and Supplementary Section 8.3). Each reaction was performed with both [Ru(bpz)_3_]^2+^ and [Cr(dqp)_2_]^3+^ under identical conditions to enable comparison (Supplementary Section 8). The concentrations of the photocatalysts were adjusted to ensure the absorption of the same amount of light emitted by the 415 nm light-emitting diode (LED) and thus the same concentration of the excited-state photocatalysts (*PC), considering the quantitative intersystem crossing for both complexes (Supplementary Section 7 and Supplementary Figs. 90, 100 and 109). This LED emitted a power density of 73 mW cm^−2^ at the sample position, which permitted monitoring of the reaction kinetics and minimized photodecomposition. The reaction set-up furthermore allowed the determination of the quantum yields for the formation of the photoproducts (*Ф*_P_) as a function of irradiation time (Supplementary Section 9).

The photocatalytic aerobic hydroxylation of phenylboronic acids (Fig. 4a) is well known and therefore was identified as a relevant benchmark reaction^37,38^. Its mechanism involves an initial electron transfer between the excited photocatalyst and an electron donor (Supplementary Fig. 89), for which DIPEA was a suitable choice. Both [Ru(bpz)_3_]^2+^ and [Cr(dqp)_2_]^3+^ photocatalysed the reaction of 4-methoxyphenylboronic acid to 4-methoxyphenol, but at very different rates (Fig. 4b), despite the identical amount of light absorbed. Based on ^1^H NMR experiments (Supplementary Section 8.1), unwanted side products were not formed in substantial quantities and the initial product formation rates (*v*) were determined to be 232 ± 4 mM h^−1^ with [Cr(dqp)_2_]^3+^ and 848 ± 77 mM h^−1^ with [Ru(bpz)_3_]^2+^, respectively (Fig. 4b), corresponding to a ratio of *ν*(Ru^II^)/*ν*(Cr^III^) of 3.7 ± 0.3. For the initial product formation quantum yields, a ratio of *Ф*_P_(Ru^II^)/*Ф*_P_(Cr^III^) of 3.3 ± 0.4 (Supplementary Fig. 117) was obtained. These ratios come close to the cage escape quantum yield ratio of *Ф*_CE_(Ru^II^)/*Ф*_CE_(Cr^III^) = 4.0 ± 0.7 determined for the [Ru(bpz)_3_]^2+^–DIPEA and [Cr(dqp)_2_]^3+^–DIPEA couples (Fig. 4c,j). Under the photocatalytic reaction conditions, the difference between *k*_q_ for the Ru^II^ and Cr^III^ complexes was unimportant because excited-state quenching by photoinduced electron transfer was essentially quantitative in the presence of 0.25 M DIPEA (Supplementary Section 8.1).

The debromination of α-bromoketones is another well-explored benchmark photoredox reaction^39,40^; we chose to perform this reaction with a different electron donor (DMT) that should result in a bigger difference in the cage escape quantum yields for [Ru(bpz)_3_]^2+^ and [Cr(dqp)_2_]^3+^ (Fig. 2d). Under the conditions used for the reductive debromination of 2-bromoacetophenone (Fig. 4d), 99% of the 415 nm excitation light was absorbed by the [Ru(bpz)_3_]^2+^ and [Cr(dqp)_2_]^3+^ photocatalysts, and any background reactivity caused by the direct absorption of light by the substrate was negligible (Supplementary Section 8.2). Analogously to the aerobic hydroxylation reaction, the initial step is a photoinduced electron transfer from the electron donor to the photocatalyst (Supplementary Fig. 99)^41^, which, in the presence of 0.1 M DMT, is essentially quantitative and therefore not performance limiting for either photocatalyst (Supplementary Section 8.2). Several side products, including a pinacol coupling product^42^ and a DMT dimer, were formed in minor amounts (Supplementary Figs. 99, 101 and 102), but the anticipated formation of acetophenone clearly dominated. The initial product formation rates were determined to be 2.07 ± 0.06 mM h^−1^ with [Ru(bpz)_3_]^2+^ and 0.24 ± 0.01 mM h^−1^ with [Cr(dqp)_2_]^3+^ (Fig. 4e), corresponding to a *ν*(Ru^II^)/*ν*(Cr^III^) ratio of 8.6 ± 0.5. The ratio of the initial product formation quantum yields *Ф*_P_(Ru^II^)/*Ф*_P_(Cr^III^) was 8.4 ± 0.9 (Supplementary Fig. 118). Both of these ratios come close to the ratio of the cage escape quantum yields, with *Ф*_CE_(Ru^II^)/*Ф*_CE_(Cr^III^) = 10.6 ± 1.3 for the DMT donor used here (Fig. 4f,j). After successful cage escape, there was no measurable difference in the onward reaction rates of the Ru^II^ and Cr^III^ complexes within the limits of the experiments performed (Supplementary Section 8.2 and Supplementary Fig. 107).

As a third example, we chose the visible light-mediated aza-Henry reaction of nitromethane with THIQ (Fig. 4g), which is a frequently explored photocatalytic process involving C–H activation^43,44^. This reaction is known to occur by both the excitation of a photocatalyst and direct excitation of THIQ (Supplementary Fig. 108)^43^. Control experiments performed in the absence of photocatalyst confirmed this earlier finding (Supplementary Fig. 112) and signalled the formation of a known hydroperoxide side product and a THIQ dimer. In the presence of 0.4 mol% [Ru(bpz)_3_]^2+^ or 1.0 mol% [Cr(dqp)_2_]^3+^, 87% of the 415 nm excitation light was absorbed by the photocatalyst (Supplementary Fig. 109) and consequently some background reactivity involving the direct excitation of THIQ cannot be excluded. In the aza-Henry reaction, the electron donor is not a sacrificial reagent but acts as the substrate, hence the scheme in Fig. 1a does not fully apply. Nonetheless, the initial photoinduced electron transfer from THIQ to excited [Ru(bpz)_3_]^2+^ and [Cr(dqp)_2_]^3+^ is quantitative given the relevant rate constants (*k*_q_, Fig. 2c) and THIQ concentrations (50 mM; Supplementary Section 8.3). The product formation rates were determined to be 1.97 ± 0.12 mM h^−1^ for [Ru(bpz)_3_]^2+^ and 0.56 ± 0.04 mM h^−1^ for [Cr(dqp)_2_]^3+^ (Fig. 4h), corresponding to a *ν*(Ru^II^)/*ν*(Cr^III^) ratio of 3.5 ± 0.3, whereas a ratio of *Ф*_P_(Ru^II^)/*Ф*_P_(Cr^III^) of 3.6 ± 0.5 for THIQ-MeNO_2_ formation was obtained (Supplementary Fig. 119). These two ratios approach that of the cage escape quantum yields *Ф*_CE_(Ru^II^)/*Ф*_CE_(Cr^III^) > 5.0 for the [Ru(bpz)_3_]^2+^–THIQ and [Cr(dqp)_2_]^3+^–THIQ donor–acceptor couples (Fig. 4i,j). The cage escape quantum yield for the latter is at the detection limit, hence only a lower limit of 5.0 can be given for the ratio of *Ф*_CE_ values in this case. The photoreaction with [Ru(bpz)_3_]^2+^ yielded appreciable amounts of a THIQ dimer (Supplementary Fig. 110), which was not the case with [Cr(dqp)_2_]^3+^ (Supplementary Fig. 111). When taking the formation of this THIQ dimer side product into account, an overall reaction rate ratio *ν*(Ru^II^)/*ν*(Cr^III^) of 6.6 ± 1.7 was obtained (Supplementary Fig. 115).

Overall, the key finding from these three photoredox reactions (Fig. 4) is that the initial product formation rates (*ν*) and the initial product formation quantum yields (*Ф*_P_) extracted from the photocatalysis experiments correlate with the cage escape quantum yields (*Ф*_CE_), determined by transient absorption spectroscopy (Fig. 4j).

## Conclusions

The ^3^MLCT-excited [Ru(bpz)_3_]^2+^ and the spin-flip excited states of [Cr(dqp)_2_]^3+^ underwent photoinduced electron-transfer reactions with 12 amine-based electron donors similarly well, but provided cage escape quantum yields differing by up to an order of magnitude. In three exemplary benchmark photoredox reactions performed with different electron donors, the differences in the reaction rates observed when using either [Ru(bpz)_3_]^2+^ or [Cr(dqp)_2_]^3+^ as photocatalyst correlated with the magnitude of the cage escape quantum yields. These correlations indicate that the cage escape quantum yields play a decisive role in the reaction rates and quantum efficiencies of the photoredox reactions, and also illustrate that luminescence quenching experiments are insufficient for obtaining quantitative insights into photoredox reactivity.

From a purely physical chemistry perspective, these findings are not a priori surprising as the rate of photoproduct formation in an overall reaction comprising several consecutive elementary steps can be expressed as the product of the quantum yields of the individual elementary steps^45,46^. A recent report on solvent-dependent cage escape and photoredox studies suggested that the correlations between photoredox product formation rates and cage escape quantum yields might be observable^11^, but we are unaware of previous reports that have been able to demonstrate that the rate of product formation in several batch-type photoreactions correlates with the cage escape quantum yields determined from laser experiments. Synthetic photochemistry and mechanistic investigations are often conducted under substantially different conditions, which can lead to controversial discrepancies^47–49^, whereas here their mutual agreement seems remarkable, particularly given the complexity of the overall reactions.

The available data and the presented analysis suggest that the different cage escape behaviours of [Ru(bpz)_3_]^2+^ and [Cr(dqp)_2_]^3+^ originate in the fact that for any given electron donor, in-cage reverse electron transfer is ~0.3 eV more exergonic for the Ru^II^ complex than for the Cr^III^ complex. Thermal reverse electron transfer between caged radical pairs therefore occurs more deeply in the Marcus inverted region with [Ru(bpz)_3_]^2+^ than with [Cr(dqp)_2_]^3+^, decelerating in-cage charge recombination in the Ru^II^ complex and increasing the cage escape quantum yields compared with the Cr^III^ complex (Fig. 3d). Previous transient absorption studies have already noted the special role of the Marcus inverted region in in-cage charge recombination^19,26^, and recent work on light-driven metal reduction processes with Ir^III^ complexes revealed that cage escape quantum yields depend substantially on Marcus theory considerations of the photocatalyst and are highly relevant to the reaction efficiency in photoredox catalysis^16^.

Our study demonstrates how the nature and redox properties of the photocatalyst affect the cage escape quantum yields and the achievable photoredox reaction rates, as well as the overall photoreaction quantum yields. Choosing a photocatalyst that makes in-cage charge recombination with a given reactant as highly exergonic as possible (relative to the reorganization energy) could represent a generally useful strategy for maximizing cage escape quantum yields and consequently photoredox reaction rates and quantum yields. Particularly strongly oxidizing or reducing photocatalysts^50–52^, ideally in combination with high excited-state energies, could be advantageous for that purpose.

## Online content

Any methods, additional references, Nature Portfolio reporting summaries, source data, extended data, supplementary information, acknowledgements, peer review information; details of author contributions and competing interests; and statements of data and code availability are available at 10.1038/s41557-024-01482-4.

### Supplementary information

Supplementary Information Supplementary Discussion, Figs. 1–133 and Tables 1–6.

### Source data

Source Data Fig. 2 Relative actinometry, photoinduced electron transfer and cage escape quantum yield data.

Source Data Fig. 3 Electron-transfer parameter screening data.

Source Data Fig. 4 Photoredox reaction and cage escape data.

## Data Availability

All the data that support the findings of this paper are available via figshare at 10.6084/m9.figshare.22294531 (ref. ^54^). Source data are provided with this paper.

## References

[1] Romero NA, Nicewicz DA (2016). Organic photoredox catalysis. Chem. Rev..

[2] Karkas MD, Porco JA, Stephenson CR (2016). Photochemical approaches to complex chemotypes: applications in natural product synthesis. Chem. Rev..

[3] Yoon TP, Ischay MA, Du J (2010). Visible light photocatalysis as a greener approach to photochemical synthesis. Nat. Chem..

[4] Marzo L, Pagire SK, Reiser O, König B (2018). Visible-light photocatalysis: does it make a difference in organic synthesis?. Angew. Chem. Int. Ed..

[5] Großkopf J, Kratz T, Rigotti T, Bach T (2022). Enantioselective photochemical reactions enabled by triplet energy transfer. Chem. Rev..

[6] Twilton J (2017). The merger of transition metal and photocatalysis. Nat. Rev. Chem..

[7] Arias-Rotondo DM, McCusker JK (2016). The photophysics of photoredox catalysis: a roadmap for catalyst design. Chem. Soc. Rev..

[8] Stevenson BG (2021). Mechanistic investigations of an α-aminoarylation photoredox reaction. J. Am. Chem. Soc..

[9] Buzzetti L, Crisenza GEM, Melchiorre P (2019). Mechanistic studies in photocatalysis. Angew. Chem. Int. Ed..

[10] Balzani V, Bergamini G, Ceroni P (2015). Light: a very peculiar reactant and product. Angew. Chem. Int. Ed..

[11] Aydogan A (2021). Accessing photoredox transformations with an iron(III) photosensitizer and green light. J. Am. Chem. Soc..

[12] Kjær KS (2019). Luminescence and reactivity of a charge-transfer excited iron complex with nanosecond lifetime. Science.

[13] Hoffman MZ (1988). Cage escape yields from the quenching of *Ru(bpy)_3_^2+^ by methylviologen in aqueous solution. J. Phys. Chem..

[14] Olmsted J, Meyer TJ (1987). Factors affecting cage escape yields following electron-transfer quenching. J. Phys. Chem..

[15] Ripak A (2023). Photosensitized activation of diazonium derivatives for C–B bond formation. Chem. Catal..

[16] DiLuzio S, Connell TU, Mdluli V, Kowalewski JF, Bernhard S (2022). Understanding Ir(III) photocatalyst structure–activity relationships: a highly parallelized study of light-driven metal reduction processes. J. Am. Chem. Soc..

[17] Sittel S (2023). Visible-light induced fixation of SO_2_ into organic molecules with polypyridine chromium(III) complexes. ChemCatChem.

[18] Sun H, Neshvad G, Hoffman MZ (2006). Energy gap dependence of the efficiency of charge separation upon the sacrificial reductive quenching of the excited states of Ru(II)-diimine photosensitizers in aqueous solution. Mol. Cryst. Liq. Cryst..

[19] Gould IR (1989). Electron-transfer reactions in the Marcus inverted region. Charge recombination versus charge shift reactions. J. Am. Chem. Soc..

[20] Wolff H-J, Bürßner D, Steiner UE (1995). Spin–orbit coupling controlled spin chemistry of Ru(bpy)_3_^2+^ photooxidation: detection of strong viscosity dependence of in-cage backward electron transfer rate. Pure Appl. Chem..

[21] Gibbons DJ, Farawar A, Mazzella P, Leroy-Lhez S, Williams RM (2020). Making triplets from photo-generated charges: observations, mechanisms and theory. Photochem. Photobiol. Sci..

[22] Kikuchi K, Hoshi M, Niwa T, Takahashi Y, Miyashi T (1991). Heavy-atom effects on the excited singlet-state electron-transfer reaction. J. Phys. Chem..

[23] Jayanthi S, Ramamurthy P (1999). Photoinduced electron transfer reactions of 2,4,6-triphenylpyrylium: solvent effect and charge-shift type of systems. Phys. Chem. Chem. Phys..

[24] Meidlar K, Das PK (1982). Tris(2,2′-bipyridine)ruthenium(II)-sensitized photooxidation of phenols. Environmental effects on electron transfer yields and kinetics. J. Am. Chem. Soc..

[25] Ohno T, Lichtin NN (1980). Electron transfer in the quenching of triplet methylene blue by complexes of iron(II). J. Am. Chem. Soc..

[26] Gould IR, Ege D, Moser JE, Farid S (1990). Efficiencies of photoinduced electron-transfer reactions: role of the Marcus inverted region in return electron transfer within geminate radical–ion pairs. J. Am. Chem. Soc..

[27] Kalyanasundaram, K. & Neumann-Spallart, M. Influence of added salts on the cage escape yields in the photoredox quenching of Ru(bpy)_3_^2+^ excited states. *Chem. Phys. Lett.* **88**, 7–12 (1982).

[28] Delaire JA, Sanquer-Barrie M, Webber SE (1988). Role of electrostatic interaction in light-induced charge separation in polyelectrolyte bound vinyldiphenylanthracene. J. Phys. Chem..

[29] Das PK, Encinas V, Scaiano JC (1981). Laser flash photolysis study of the reactions of carbonyl triplets with phenols and photochemistry of *p*-hydroxypropiophenone. J. Am. Chem. Soc..

[30] Bürgin TH, Glaser F, Wenger OS (2022). Shedding light on the oxidizing properties of spin-flip excited states in a Cr^III^ polypyridine complex and their use in photoredox catalysis. J. Am. Chem. Soc..

[31] Rehm D, Weller A (1969). Kinetik und Mechanismus der Elektronübertragung bei der Fluoreszenzlöschung in Acetonitril. Ber. Bunsen-Ges. Phys. Chem..

[32] Ballardini R, Varani G, Indelli MT, Scandola F, Balzani V (1978). Free energy correlation of rate constants for electron transfer quenching of excited transition metal complexes. J. Am. Chem. Soc..

[33] Neumann S, Kerzig C, Wenger OS (2019). Quantitative insights into charge-separated states from one- and two-pulse laser experiments relevant for artificial photosynthesis. Chem. Sci..

[34] DeLaive PJ, Foreman TK, Giannotti C, Whitten DG (1980). Photoinduced electron transfer reactions of transition-metal complexes with amines. Mechanistic studies of alternate pathways to back electron transfer. J. Am. Chem. Soc..

[35] Kitzmann WR, Heinze K (2022). Charge-transfer and spin-flip states: thriving as complements. Angew. Chem. Int. Ed..

[36] Gould IR, Moser JE, Ege D, Farid S (1988). Effect of molecular dimension on the rate of return electron transfer within photoproduced geminate radical ion pairs. J. Am. Chem. Soc..

[37] Pitre SP, McTiernan CD, Ismaili H, Scaiano JC (2013). Mechanistic insights and kinetic analysis for the oxidative hydroxylation of arylboronic acids by visible light photoredox catalysis: a metal-free alternative. J. Am. Chem. Soc..

[38] Zou Y-Q (2012). Highly efficient aerobic oxidative hydroxylation of arylboronic acids: photoredox catalysis using visible light. Angew. Chem. Int. Ed..

[39] Maji T, Karmakar A, Reiser O (2011). Visible-light photoredox catalysis: dehalogenation of vicinal dibromo-, α-halo-, and α,α-dibromocarbonyl compounds. J. Org. Chem..

[40] Prier CK, Rankic DA, MacMillan DWC (2013). Visible light photoredox catalysis with transition metal complexes: applications in organic synthesis. Chem. Rev..

[41] Nicewicz DA, MacMillan DWC (2008). Merging photoredox catalysis with organocatalysis: the direct asymmetric alkylation of aldehydes. Science.

[42] Glaser F, Kerzig C, Wenger OS (2021). Sensitization-initiated electron transfer via upconversion: mechanism and photocatalytic applications. Chem. Sci..

[43] Bartling H, Eisenhofer A, König B, Gschwind RM (2016). The photocatalyzed aza-Henry reaction of *N*-aryltetrahydroisoquinolines: comprehensive mechanism, H^·^- versus H^+^-abstraction, and background reactions. J. Am. Chem. Soc..

[44] Condie AG, González-Gómez JC, Stephenson CRJ (2010). Visible-light photoredox catalysis: aza-Henry reactions via C–H functionalization. J. Am. Chem. Soc..

[45] Scaiano, J. C. T. *Photochemistry Essentials* (American Chemical Society, 2022).

[46] Talbott ED, Burnett NL, Swierk JR (2023). Mechanistic and kinetic studies of visible light photoredox reactions. Chem. Phys. Rev..

[47] Marchini M, Bergamini G, Cozzi PG, Ceroni P, Balzani V (2017). Photoredox catalysis: the need to elucidate the photochemical mechanism. Angew. Chem. Int. Ed..

[48] Ghosh I, Bardagi JI, König B (2017). Reply to “Photoredox Catalysis: The Need to Elucidate the Photochemical Mechanism”. Angew. Chem. Int. Ed..

[49] Coles MS, Quach G, Beves JE, Moore EG (2020). A photophysical study of sensitization-initiated electron transfer: insights into the mechanism of photoredox activity. Angew. Chem. Int. Ed..

[50] Tlili A, Lakhdar S (2021). Acridinium salts and cyanoarenes as powerful photocatalysts: opportunities in organic synthesis. Angew. Chem. Int. Ed..

[51] Speckmeier E, Fischer TG, Zeitler K (2018). A toolbox approach to construct broadly applicable metal-free catalysts for photoredox chemistry: deliberate tuning of redox potentials and importance of halogens in donor–acceptor cyanoarenes. J. Am. Chem. Soc..

[52] Kim D, Teets TS (2022). Strategies for accessing photosensitizers with extreme redox potentials. Chem. Phys. Rev..

[53] Krzywinski M, Altman N (2014). Visualizing samples with box plots. Nat. Methods.

[54] Wang, C., Li, H., Bürgin, T. H. & Wenger, O. S. Data of figures and tables contained in the paper titled ‘Cage escape governs photoredox reaction rates and quantum yields’. *figshare*10.6084/m9.figshare.22294531 (2024). 10.1038/s41557-024-01482-4 PMC11230909 38499849

