# Supplementary material for: Cage escape governs photoredox reaction rates and quantum yields
Source: Nat Chem. 2024 Mar 18;16(7):1151–9. doi: 10.1038/s41557-024-01482-4 (PMC11230909; doi:10.1038/s41557-024-01482-4)
Supplement: Supplementary file 1 — Supplementary Discussion, Figs. 1–133 and Tables 1–6. [file 41557_2024_1482_MOESM1_ESM.pdf]

nature chemistry

Article

https://doi.org/10.1038/s41557-024-01482-4

Cage escape governs photoredox reaction rates and quantum yields

In the format provided by the authors and unedited

Table of Contents

1. Materials and methods 3

2. Synthesis and characterization 4

2.1 Synthesis of 4,4',4''-Nitrilotriphenol (TAA-OH) 4

2.2 Synthesis of TAA-PEG_3 4

2.3 Synthesis of PEG_7-OH 5

2.4 Synthesis of PEG_7-Br 5

2.5 Synthesis of TAA-PEG_7 6

3. Electrochemistry 7

4. UV-vis spectro-electrochemical studies of electron donors 9

5. Excited-state quenching studies 14

5.1 Quenching of $[Ru(bpz)_3]^{2+}$ 14

5.2 Quenching of $[Cr(dqp)_2]^{3+}$ 20

6. Determination of changes in extinction coefficients *Δε* 27

6.1 $\Delta\varepsilon$ of $[Ru(bpz)_3]^{+}$ 27

6.2 $\Delta\varepsilon$ of $[Cr(dqp)_2]^{2+}$ 28

6.3 $\Delta\varepsilon$ of TAA-$OMe^{\bullet+}$ 29

6.4 $\Delta\varepsilon$ of TAA-$PEG_3^{\bullet+}$ 30

6.5 $\Delta\varepsilon$ of TAA-$PEG_7^{\bullet+}$ 31

6.6 $\Delta\varepsilon$ of TAA-$Cl^{\bullet+}$ 33

6.7 $\Delta\varepsilon$ of TAA-$Br^{\bullet+}$ 34

6.8 $\Delta\varepsilon$ of TAA-$I^{\bullet+}$ 35

6.9 $\Delta\varepsilon$ of $DMA^{\bullet+}$ 35

6.10 $\Delta\varepsilon$ of $DMT^{\bullet+}$ 36

6.11 $\Delta\varepsilon$ of DMA-$OMe^{\bullet+}$ 37

7. Determination of cage escape quantum yields Φ_{CE} 38

7.1 TAA-OMe 40

7.2 TAA-PEG_3 41

7.3 TAA-PEG_7 43

7.4 TAA-Cl 45

7.5 TAA-Br 46

7.6 TAA-I 47

7.7 DMA 48

7.8 DMT 49

7.9 DMA-OMe 50

7.10 THIQ 51

7.11 TEA 53

7.12 DIPEA 54

7.13 Summary 55

7.13.1 Driving-force effects 56

7.13.2 Spin effects 67

7.13.3 Heavy atom effects 70

7.13.4 Size and viscosity effects 71

7.13.5 Ionic strength 71

7.13.6 Electrostatic repulsion 71

8. Photocatalytic reactions 71

8.1 Photocatalytic aerobic hydroxylation of arylboronic acid 73

Under 7.0 W LED irradiation 74

Under 230 mW LED irradiation 77

8.2 Photocatalytic reductive debromination of 2-bromoacetophenone 80

8.3 Photocatalytic Aza-Henry reaction 89

9. Determination of the quantum yields Φ_P for photoproduct formation 98

10. NMR and mass spectra 101

11. References 109

1. Materials and methods

Dry acetonitrile (99.9%, Thermo Fischer), deuterated acetonitrile (99.8%, Eurisotope), deuterium oxide (99.9%, Cambridge Isotope Laboratories (CIL)), and deuterated dimethyl sulfoxide (DMSO-d6, 99.8% Apollo Scientific) were used as commercially received. All chemicals for synthesis and optical spectroscopy with high purity were used as received from commercial suppliers (Acros Organics, Alfa Aesar, Fischer Scientific and Sigma-Aldrich), unless otherwise indicated.

2,6-Di(quinolin-8-yl)pyridine (dqp),[1] tri(4-methoxyphenyl)amine (TAA-OMe),[2] tris(4-chlorophenyl)amine (TAA-Cl),[3] 4-methoxy-N,N-dimethylaniline (DMA-OMe),[4] and N-phenyl tetrahydroisoquinoline (THIQ)[5] were synthesized according to literature procedures. $[Cr(dqp)_2][PF_6]_3$ was synthesized as previously reported by us.[6]

The solutions for UV-vis absorption and transient absorption studies were prepared in dry solvents under ambient conditions and measured in quartz cuvettes (10 x 10 mm) under air at room temperature, unless otherwise stated.

Reaction products were characterized on Bruker Avance III NMR instruments operating at 400 MHz or 500 MHz proton frequency, while a Bruker Avance III NMR instrument with 250 MHz proton frequency was used to record the reaction kinetics over time. Deuterated solvents were used, and chemical shifts in ppm are referenced to their respective residual proton NMR signal.[7] All coupling constants *J* are given in Hertz (Hz) with the coupling patterns termed as: s (singlet), d (doublet), t (triplet), q (quartet), m (multiplet) and combinations of these abbreviations.

UV-vis absorption spectra were recorded on a Cary 5000 (Varian) spectrophotometer.

UV-vis transient absorption spectra and decay kinetics were measured on a LP920-KS apparatus from Edinburgh Instruments. For these experiments, a frequency-tripled pulsed Nd:YAG laser (Quantel Q-smart 450, ca. 10 ns pulse width) with a beam expander (BE02-355 from Thorlabs) in the beam path was used for excitation at 355 nm (pulse energy of either ~ 40 or ~ 100 mJ). A frequency-tripled Nd:YAG laser (Quantel Brilliant, ca. 10 ns pulse width) coupled with an OPO from Opotek and a beam expander (GBE02-A from Thorlabs) in the beam path was used for tunable excitation between 410 and 470 nm (pulse energy of 6~14 mJ with the maximal power reached at ca. 450 nm). Transient absorption spectra were recorded with an iCCD camera from Andor, while the decay kinetics at individual detection wavelengths were measured with a photomultiplier tube (PMT).

For cyclic voltammetry measurements, a saturated calomel electrode (SCE) was used as the reference, a glassy carbon disk as the working electrode, and a silver wire as the counter electrode in a three-neck glass cell containing the sample solution (1 mM substrate and 100 mM tetra-*n*-butylammonium hexafluorophosphate, $TBAPF_6$) in aerated dry acetonitrile. A Versastat3-200 potentiostat (Princeton Applied Research) was used with a potential scan rate of 100 mV/s. For spectro-electrochemical absorption measurements, the same setup was used and combined with the Cary 5000 UV-vis absorption spectrometer. Solutions (0.2 ~ 0.5 mM compound of interest in aerated CH_3CN containing 0.1 M $TBAPF_6$) were prepared in a 1 mm quartz cuvette, equipped with a platinum grid as the working electrode, a platinum wire as the counter electrode, and a SCE reference.

For photocatalytic studies, a tunable SOLIS-415C high power 415 nm LED (7.0 W) from Thorlabs with a collimated beam was employed. A 400 nm long pass filter was attached to the LED. The irradiation power density at the sample position (13 cm distance between the sample and the LED) was

determined by measuring the irradiation power with a power meter (COHERENT, Field MaxII-TOP Laser Power and Energy Meter) and the beam size with a beam profiler (Newsport, BM-USB3-SP932U). To slow down the photocatalytic process and to minimize the photodecomposition of the catalysts, the irradiation power density at the sample position was decreased from the maximal output of ~ 600 mW cm^{-2} to ~ 73 mW cm^{-2}. Unwanted heating of the reaction mixture by the LED irradiation was minimized by placing the sample in a water bath with a running stirring system. The reaction mixtures were loaded in NMR tubes and irradiated over time.

2. Synthesis and characterization

2.1 Synthesis of 4,4',4''-Nitrilotriphenol (TAA-OH)

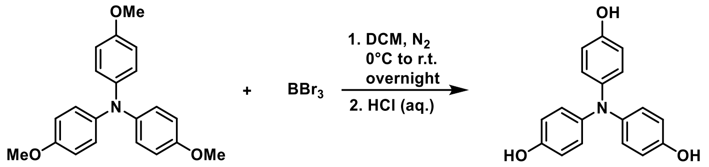

TAA-OH was synthesized according to the literature.[8] In a round bottom flask, tri(4-methoxyphenyl)amine (TAA-OMe) (400 mg, 1.2 mol) was weighted and 3 mL CH_2Cl_2 was added. The solution was cooled on an ice bath, and a solution of boron tribromide (1.11 mL, 6.5 mmol) in 10 mL dichloromethane (DCM) was added dropwise under nitrogen protection. While stirring, the mixture was heated up to room temperature and stirred overnight. After the reaction was completed, the mixture was hydrolyzed with water in an ice bath, and then was acidified with aqueous HCl solution (3 M). The resulting solution was extracted with ethyl acetate, washed twice with water, and dried over anhydrous Na_2SO_4. During the second washing with water, the organic phase became red/pink. Removing the solvent under reduced pressure yielded a blue-grey solid. The product was purified by flash chromatography (silica gel, eluent: petroleum ether/EtOAc = 2/1, v/v) to obtain the grey product **TAA-OH** (353.0 mg, 99%). NMR spectral data are in agreement with previously published spectra.[8]

^{1}H NMR (400 MHz, DMSO) δ 9.05 (s, 3H), 6.76 – 6.69 (m, 6H), 6.63 (d, *J* = 2.4 Hz, 6H).
^{13}C NMR (126 MHz, DMSO) δ 152.4, 140.4, 124.4, 115.8.

2.2 Synthesis of TAA-PEG_3

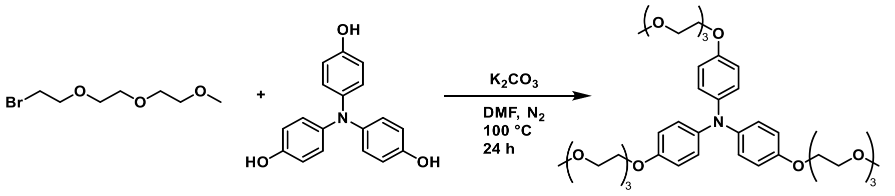

TAA-PEG_3 was synthesized by adapting a previously published protocol.[9] In a 10-15 mL microwave vial, **TAA-OH** (147 mg, 0.5 mmol), commercially available 1-bromo-3,6,9-trioxadecane (PEG_3-Br, 363 mg, 1.6 mmol), and potassium carbonate (484 mg, 3.5 mmol) were weighted. Air in the vial was removed by repeating vacuum-nitrogen cycles for three times. 10 mL degassed dimethylformamide (DMF) was then added to the mixture. The sealed mixture was then stirred and heated in an oil bath at 100°C for one day. After cooling to room temperature, the solvent was removed under reduced pressure. Adding DCM to the remaining oil, a precipitate was formed and removed. The organic phase was washed with

aqueous NaCl solution. The organic layer was collected and dried over anhydrous Na_2SO_4. Removing the solvent yielded the crude product. The product was purified by flash chromatography (silica gel, eluent: DCM/methanol = 100/1 to 10/1, v/v) to obtain the desired product as a brown-yellow oil (238.2 mg, 65%).

^{1}H NMR (400 MHz, CD_3CN) δ 6.90 (d, *J* = 7.5 Hz, 2H), 6.82 (d, *J* = 9.0 Hz, 2H), 4.05 (t, *J* = 4.6 Hz, 2H), 3.78 – 3.70 (m, 2H), 3.63 – 3.59 (m, 2H), 3.57 – 3.52 (m, 4H), 3.48 – 3.44 (m, 2H), 3.28 (s, 3H).

^{13}C NMR (126 MHz, CD_3CN) δ 155.3, 125.7, 118.3, 116.2, 72.6, 71.3, 71.1, 71.0, 70.3, 68.6, 58.9.

ESI-HRMS (m/z): calcd. for $[M + Na]^+$: 754.3773; found: 754.3776; calcd. for $[M + K]^+$: 770.3512; found: 770.3504.

Elemental analysis: $C_{39}H_{57}NO_{12}$•0.6 H_2O: Calculated (%): C, 63.07; H, 7.90; N, 1.89; Experiment (%): C, 63.03; H, 7.81; N, 1.98.

2.3 Synthesis of PEG_7-OH

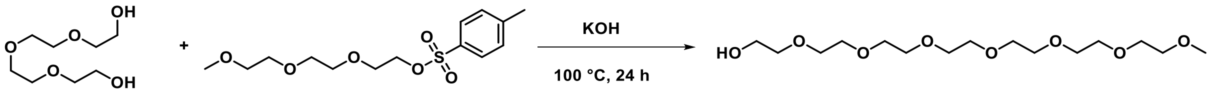

PEG_7-OH was synthesized following a previously published protocol.[10] To a 100 ml round bottom flask was added tetraethylene glycol (1.9468 g, 10.02 mmol), 2-(2-(2-methoxyethoxy)ethoxy)ethyl 4-methylbenzenesulfonate[10] (638.4 mg, 2.00 mmol), and freshly ground KOH (355.9 mg, 6.34 mmol). The heterogeneous mixture was refluxed at 100 ˚C for 24 h with stirring. The solution was allowed to cool down to room temperature, then 100 mL H_2O was added to the mixture, and the mixture was extracted with DCM (3 × 50 mL). The combined organic layers were dried with anhydrous Na_2SO_4, filtered, and the solvent was removed via evaporation. The crude product was purified via chromatography (on silica gel, eluent: DCM/MeOH = 25/1 to 10/1, v/v) to obtain a clear oil, **PEG_7-OH** (680 mg, 99%). NMR spectral data are in agreement with previously published spectra.[11]

^{1}H NMR (500 MHz, $CDCl_3$) δ 3.71 (d, J = 2.1 Hz, 2H), 3.65 (d, J = 3.1 Hz, 22H), 3.60 (d, J = 4.0 Hz, 2H), 3.56 – 3.52 (m, 2H), 3.37 (d, J = 2.9 Hz, 3H).
^{13}C NMR (126 MHz, $CDCl_3$) δ 72.7, 72.1,70.7, 70.6, 70.4, 70.1 (s), 61.9, 61.7, 59.2.

2.4 Synthesis of PEG_7-Br

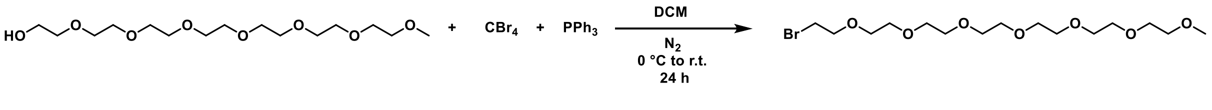

PEG_7-Br was synthesized according to literature.[12] 10 mL of a degassed dry DCM solution of PEG_7-OH (680 mg, 2.0 mmol) was added to CBr_4 (800.6 mg, 2.41 mmol) in an ice-water bath under nitrogen atmosphere. The reaction mixture was cooled at 0 °C for 10 minutes. Then 6 mL of a degassed dry DCM solution of PPh_3 (786.4 mg, 3.00 mmol) was added to the mixture at 0 °C under nitrogen. The reaction mixture was stirred overnight, while allowing the temperature to increase from 0 ˚C to room temperature. Then the solvent was evaporated under reduced pressure. The crude product was

purified via chromatography (on silica gel, eluent: DCM/ethyl acetate = 5/1 to DCM/MeOH = 100/1 to 25/1, v/v) to obtain a pale orange oil, **PEG_7-Br** (436.1 mg, 54%). The final product was washed with Et_2O to eliminate PPh_3-related impurities. NMR spectral data are in agreement with previously published spectra.[13]

^{1}H NMR (500 MHz, $CDCl_3$) δ 3.81 (t, J = 6.3 Hz, 2H), 3.68 – 3.64 (m, 22H), 3.57 – 3.53 (m, 2H), 3.47 (t, J = 6.3 Hz, 2H), 3.38 (s, 3H).
^{13}C NMR (126 MHz, $CDCl_3$) δ 72.1, 71.4, 70.8, 70.7, 70.7, 59.2, 30.5.

2.5 Synthesis of TAA-PEG_7

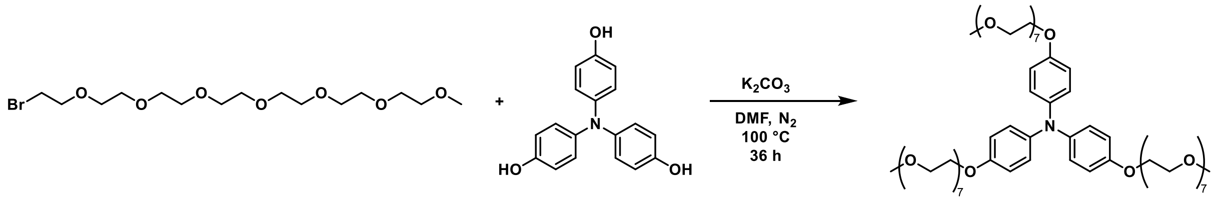

TAA-PEG_7 was synthesized by adapting a previously published method.[9] 8 mL of a degassed dry DMF solution of PEG_7-Br (369.2 mg, 0.92 mmol) was added to a mixture of K_2CO_3 (280.4 mg, 2.03 mmol) and 4,4',4''-nitrilotriphenol (83.5 mg, 0.28 mmol) in a microwave vial (10-15 mL) at room temperature under nitrogen. Then the sealed reaction mixture was heated at 100 ˚C for 36 h. After cooling to room temperature, the mixture was diluted with 30 mL H_2O and extracted with DCM (3 × 50 mL). Then the combined organic phases were washed with brine (20 mL), and afterwards dried over anhydrous Na_2SO_4. After filtration, the solvent was removed under reduced pressure. The crude product was purified via chromatography (on silica gel, eluent: DCM/MeOH = 100/1 to 10/1, v/v) to obtain a brown oil, **TAA-PEG_7** (260.0 mg, 74%).

^{1}H NMR (500 MHz, CD_3CN) δ 6.88 (d, *J* = 9.0 Hz, 6H), 6.82 – 6.76 (m, 6H), 4.09 – 3.92 (m, 6H), 3.73 (q, *J* = 4.2 Hz, 6H), 3.63 – 3.50 (m, 66H), 3.46 – 3.42 (m, 6H), 3.27 (s, 9H).
^{13}C NMR (126 MHz, CD_3CN) δ 155.22, 142.87, 125.64, 116.19, 72.54, 71.28, 71.14, 71.10, 71.07, 70.92, 70.29, 58.87.
ESI-HRMS (m/z): calcd. for $[M + 2Na]^{2+}$: 652.8405; found: 652.8400.
Elemental analysis: $C_{63}H_{105}NO_{24}$: Calculated (%): C, 60.03; H, 8.40; N, 1.11; Experiment (%): C, 59.96; H, 8.27; N, 1.24.

3. Electrochemistry

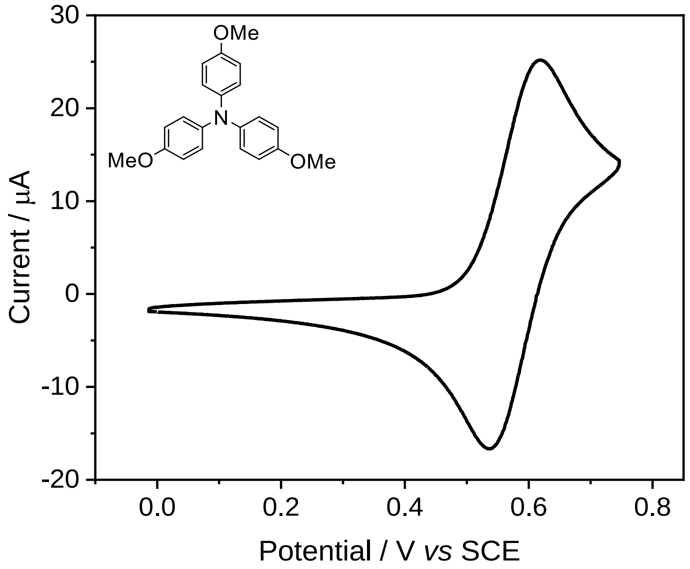

Supplementary Fig. 1: Cyclic voltammogram of 1 mM TAA-OMe in air-saturated dry CH_3CN against an SCE reference. 0.1 M $TBAPF_6$ was used as the supporting electrolyte. A glassy carbon disk was used as a working electrode, and a silver wire served as the counter electrode. The potential scan rate was 0.1 V/s.

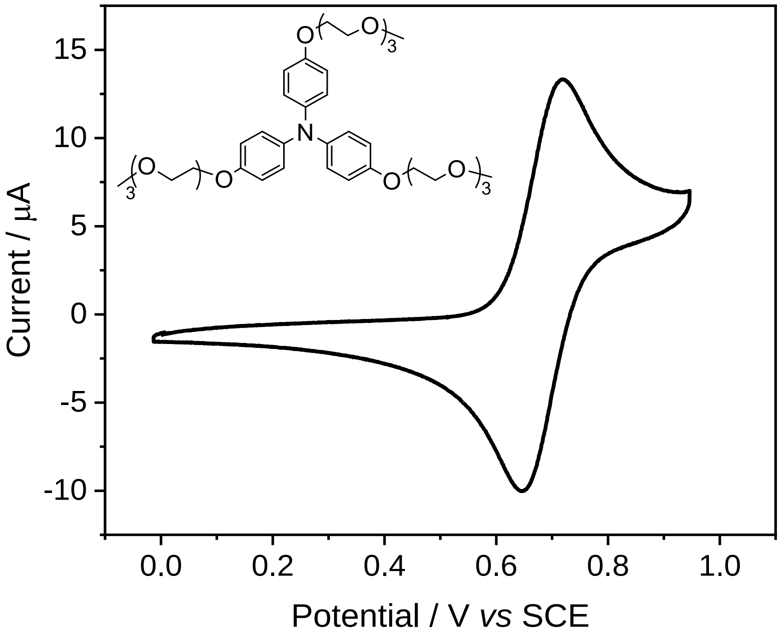

Supplementary Fig. 2: Cyclic voltammogram of 1 mM TAA-PEG_3 in air-saturated dry CH_3CN against an SCE reference. 0.1 M $TBAPF_6$ was used as the supporting electrolyte. A glassy carbon disk was used as a working electrode, and a silver wire served as the counter electrode. The potential scan rate was 0.1 V/s.

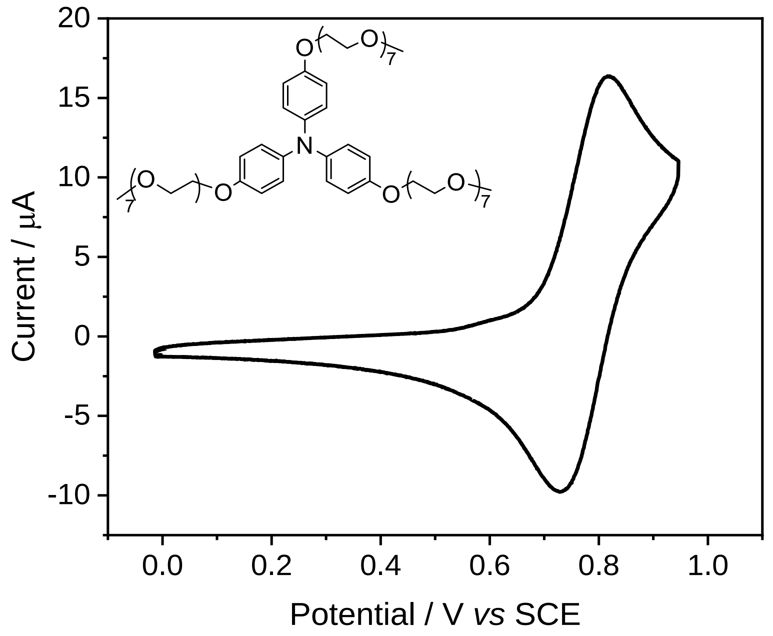

Supplementary Fig. 3: Cyclic voltammogram of 1 mM TAA-PEG$_7$ in air-saturated dry CH_3CN against an SCE reference. 0.1 M TBAPF$_6$ was used as the supporting electrolyte. A glassy carbon disk was used as a working electrode, and a silver wire served as the counter electrode. The potential scan rate was 0.1 V/s.

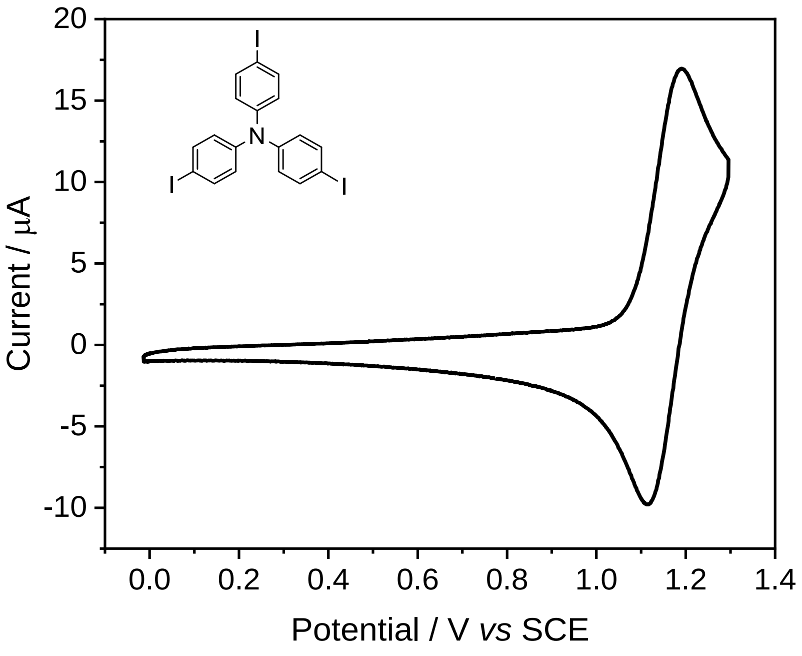

Supplementary Fig. 4: Cyclic voltammogram of 1 mM TAA-I in air-saturated dry CH_3CN against an SCE reference. 0.1 M TBAPF$_6$ was used as the supporting electrolyte. A glassy carbon disk was used as a working electrode, and a silver wire served as the counter electrode. The potential scan rate was 0.1 V/s.

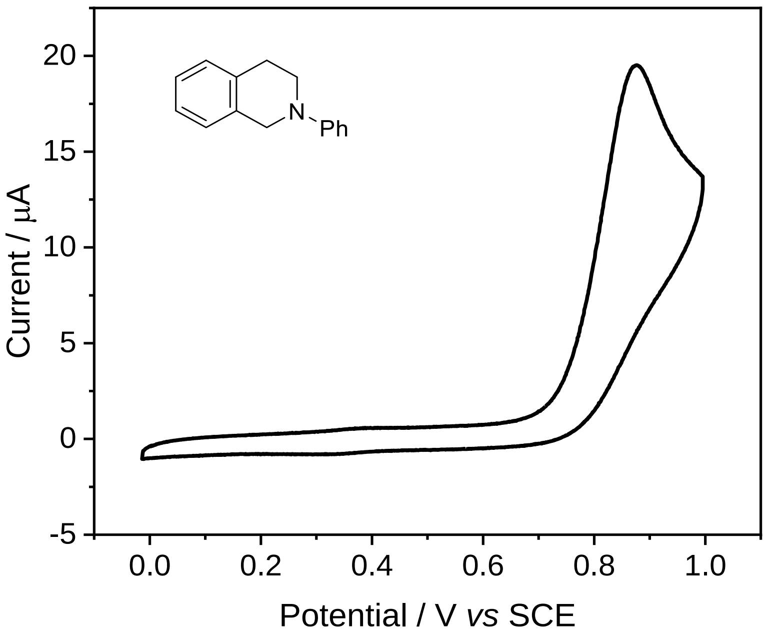

Supplementary Fig. 5: Cyclic voltammogram of 1 mM THIQ in air-saturated dry CH_3CN against an SCE reference. 0.1 M $TBAPF_6$ was used as the supporting electrolyte. A glassy carbon disk was used as a working electrode, and a silver wire served as the counter electrode. The potential scan rate was 0.1 V/s.

4. UV-vis spectro-electrochemical studies of electron donors

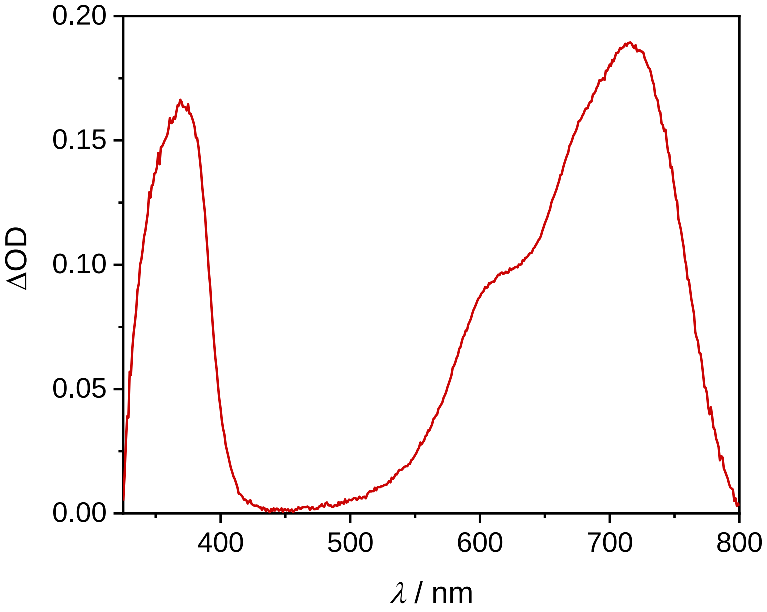

Supplementary Fig. 6: Spectro-electrochemical (SEC) UV-vis absorption spectrum of TAA-OMe (0.5 mM) in dry aerated acetonitrile containing 0.1 M $TBAPF_6$ at room temperature, obtained after applying a potential of 0.65 V vs SCE. The UV-vis spectrum recorded prior to applying this potential served as a baseline. The obtained spectrum is in agreement with the literature.[6]

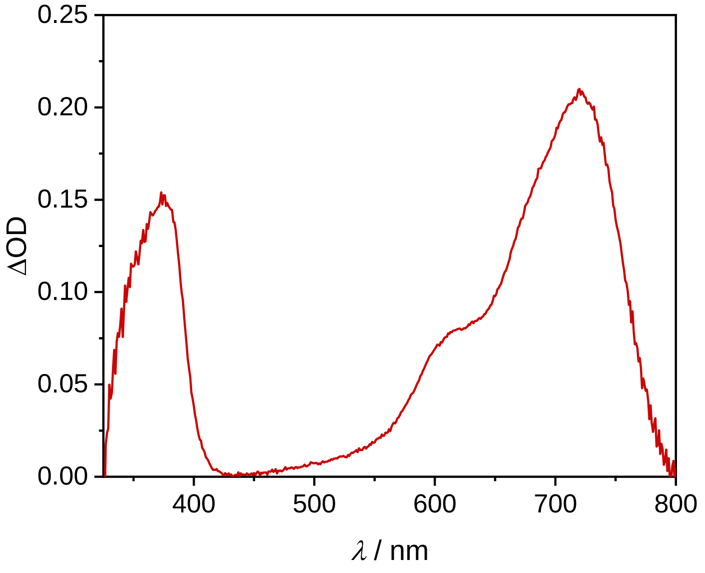

Supplementary Fig. 7: Spectro-electrochemical (SEC) UV-vis absorption spectrum of TAA-PEG$_3$ (0.3 mM) in dry aerated acetonitrile containing 0.1 M TBAPF$_6$ at room temperature, obtained after applying a potential of 0.75 V vs SCE. The UV-vis spectrum recorded prior to applying this potential served as a baseline.

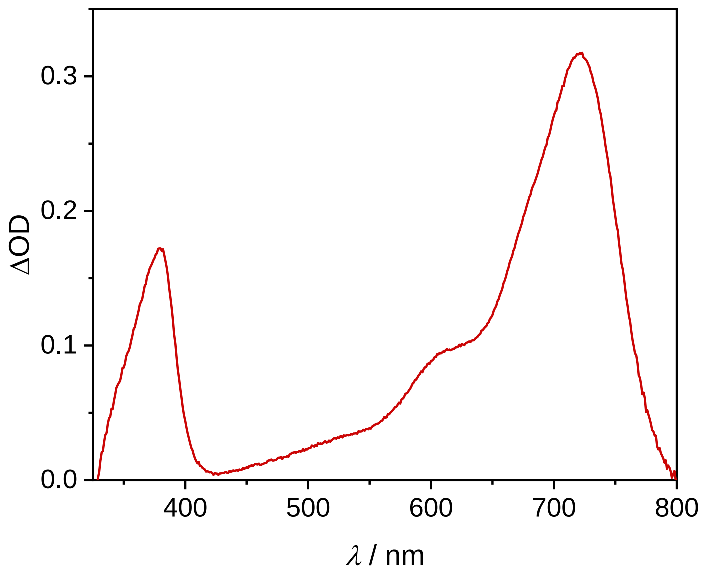

Supplementary Fig. 8: Spectro-electrochemical (SEC) UV-vis absorption spectrum of TAA-PEG$_7$ (0.5 mM) in dry aerated acetonitrile containing 0.1 M TBAPF$_6$ at room temperature, obtained after applying a potential of 0.85 V vs SCE. The UV-vis spectrum recorded prior to applying this potential served as a baseline.

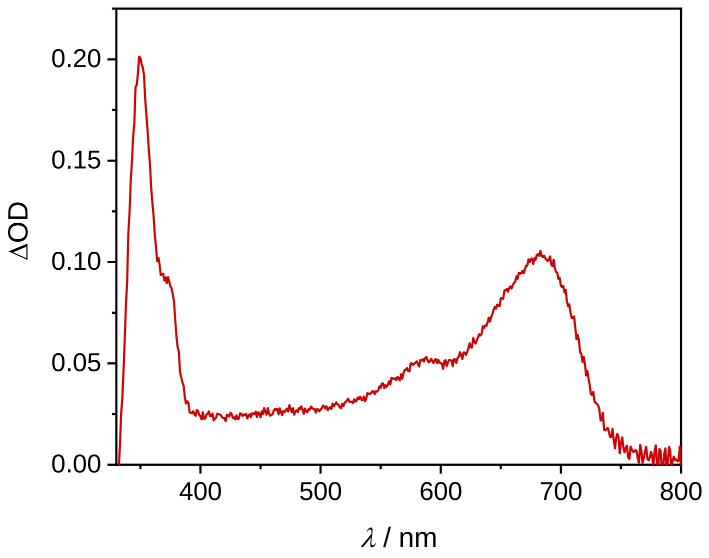

Supplementary Fig. 9: Spectro-electrochemical (SEC) UV-vis absorption spectrum of TAA-Cl (0.5 mM) in dry aerated acetonitrile containing 0.1 M $TBAPF_6$ at room temperature, obtained after applying a potential of 1.10 V vs SCE. The UV-vis spectrum recorded prior to applying this potential served as a baseline.

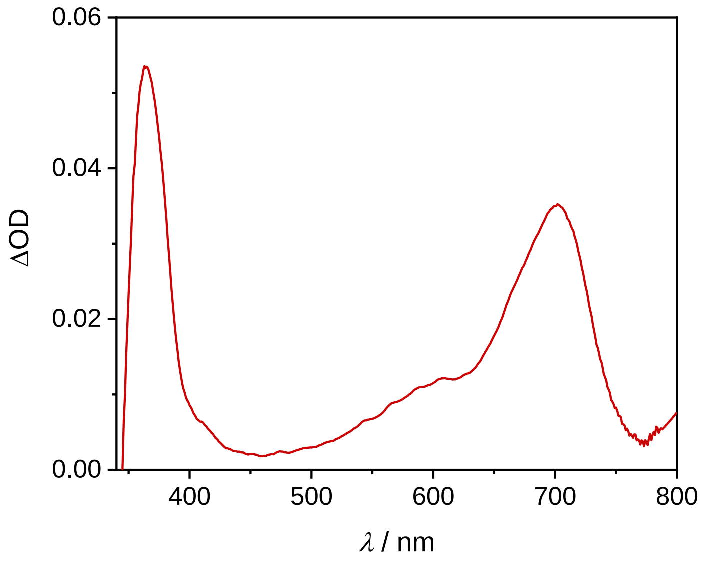

Supplementary Fig. 10: Spectro-electrochemical (SEC) UV-vis absorption spectrum of TAA-Br (0.5 mM) in dry aerated acetonitrile containing 0.1 M $TBAPF_6$ at room temperature, obtained after applying a potential of 1.15 V vs SCE. The UV-vis spectrum recorded prior to applying this potential served as a baseline.

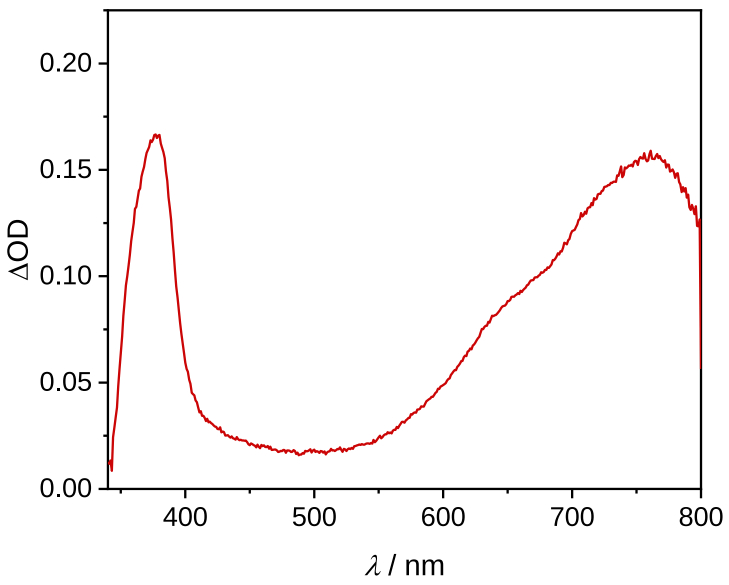

Supplementary Fig. 11: Spectro-electrochemical (SEC) UV-vis absorption spectrum of TAA-I (0.2 mM) in dry aerated acetonitrile containing 0.1 M $TBAPF_6$ at room temperature, obtained after applying a potential of 1.25 V vs SCE. The UV-vis spectrum recorded prior to applying this potential served as a baseline.

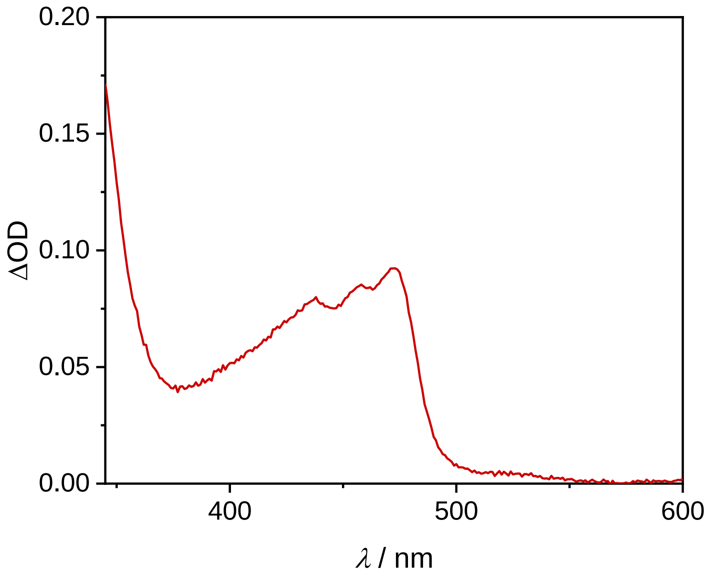

Supplementary Fig. 12*:* Spectro-electrochemical (SEC) UV-vis absorption spectrum of DMA (50 mM) in dry aerated acetonitrile containing 0.1 M $TBAPF_6$ at room temperature, obtained after applying a potential of 0.90 V vs SCE. The UV-vis spectrum recorded prior to applying this potential served as a baseline. The obtained spectrum is in agreement with the literature.[14]

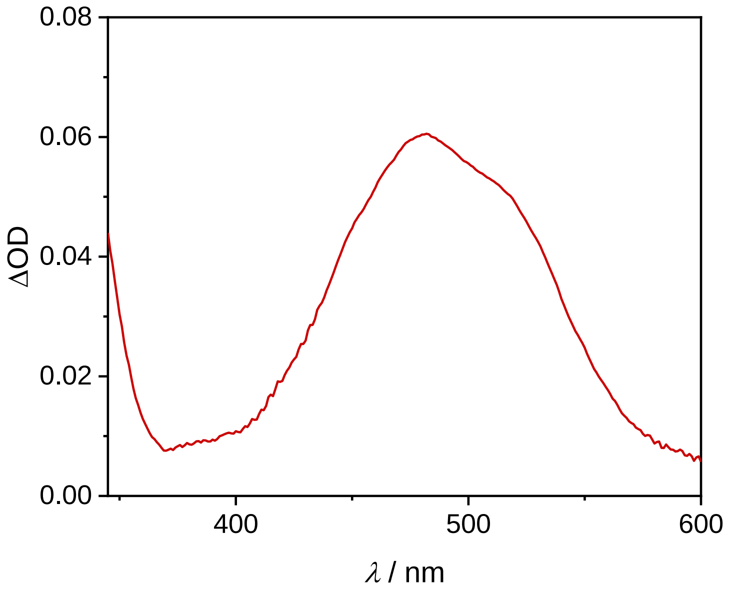

Supplementary Fig. 13: Spectro-electrochemical (SEC) UV-vis absorption spectrum of DMA-OMe (1 mM) in dry aerated acetonitrile containing 0.1 M $TBAPF_6$ at room temperature, obtained after applying a potential of 0.40 V vs SCE. The UV-vis spectrum recorded prior to applying this potential served as a baseline.

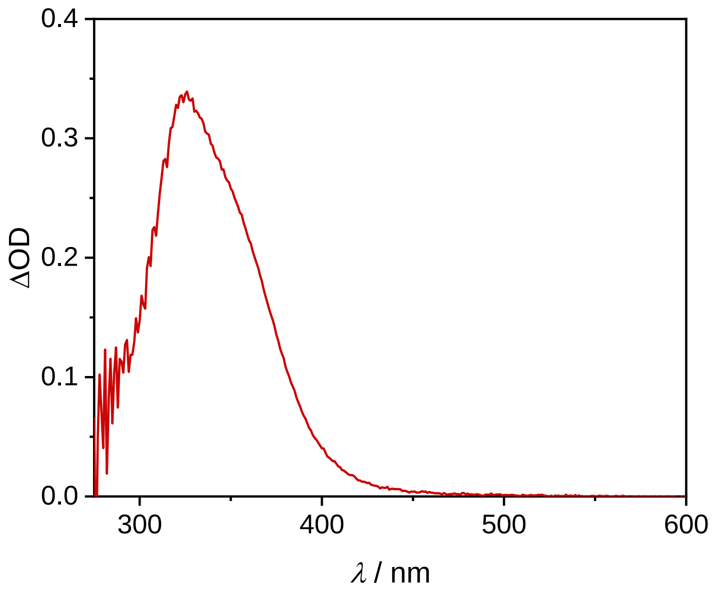

Supplementary Fig. 14*:* Spectro-electrochemical (SEC) UV-vis absorption spectrum of THIQ (1.5 mM) in dry aerated acetonitrile containing 0.1 M $TBAPF_6$ at room temperature, obtained after applying a potential of 1.00 V vs SCE. The UV-vis spectrum recorded prior to applying this potential served as a baseline. The obtained spectrum is in agreement with the literature.[15]

5. Excited-state quenching studies

5.1 Quenching of $[Ru(bpz)_3]^{2+}$

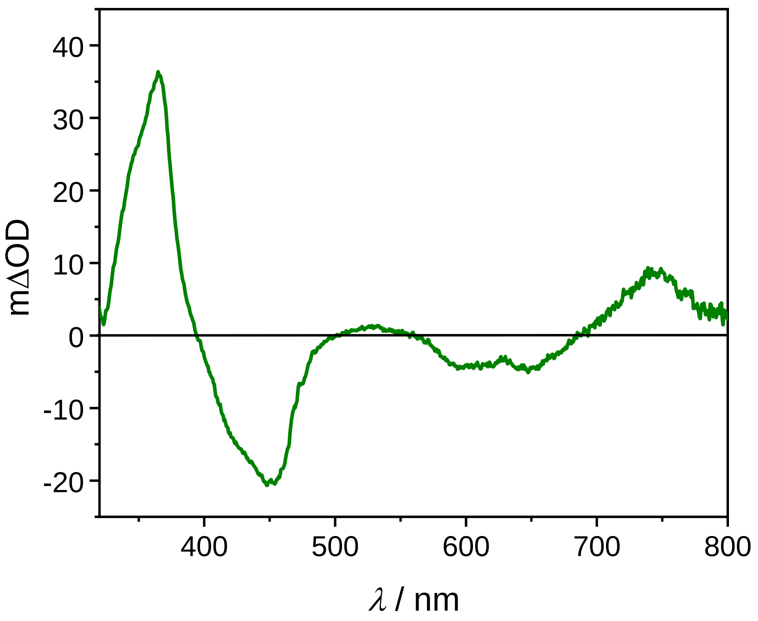

Supplementary Fig. 15: Transient absorption spectrum of $[Ru(bpz)_3]^{2+}$ (13 µM) in aerated CH_3CN under 415 nm pulsed laser excitation (pulse energy of ~ 7 mJ) at 20 °C. The spectrum was recorded with 10 ns time delay after the pulses, and the signal was time-integrated for 200 ns.

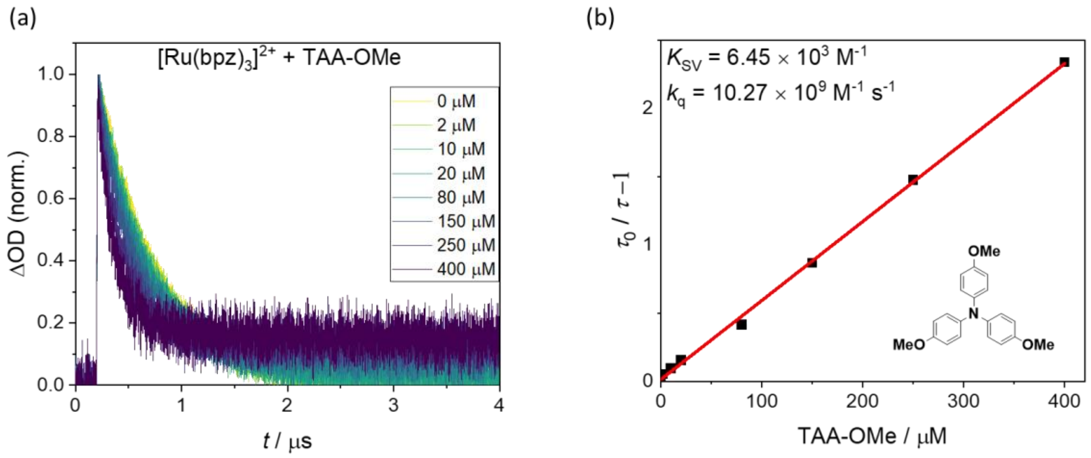

Supplementary Fig. 16: Stern-Volmer-type studies of $[Ru(bpz)_3]^{2+}$ (20 µM) in aerated CH_3CN at 20 °C with TAA-OMe: (a) normalized transient absorption decay of ^{3}MLCT-excited $[Ru(bpz)_3]^{2+}$ at 440 nm (ground state bleach; signals multiplied by a factor of -1) with increasing concentration of TAA-OMe under excitation with a ~ 10 ns pulsed 450 nm laser (pulse energy ~ 12 mJ); mono-exponential fit of the decays yields the lifetime τ. The residual long-lived signal, which becomes increasingly prominent at elevated TAA-OMe concentrations, is attributed to the transient absorption signal of $[Ru(bpz)_3]^{+}$ formed via electron transfer (see Supplementary Fig. 41 below); (b) linear Stern-Volmer plot based on lifetime quenching derived from the decays in (a).

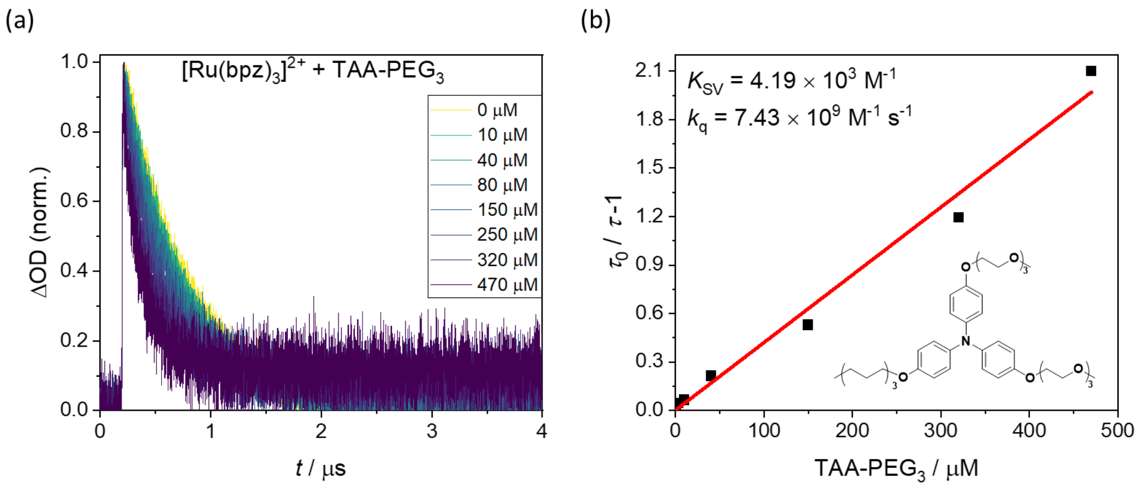

Supplementary Fig. 17: Stern-Volmer-type studies of $[Ru(bpz)_3]^{2+}$ (20 µM) in aerated CH_3CN at 20 °C with TAA-PEG$_3$: (a) normalized transient absorption decay of ^{3}MLCT-excited $[Ru(bpz)_3]^{2+}$ at 440 nm (ground state bleach; signals multiplied by a factor of -1) with increasing concentration of TAA-PEG$_3$ under excitation with a ~ 10 ns pulsed 450 nm laser (pulse energy ~ 10 mJ); mono-exponential fit of the decays yields lifetime τ. The residual long-lived signal, which becomes increasingly prominent at elevated TAA-PEG$_3$ concentrations, is attributed to the transient absorption signal of $[Ru(bpz)_3]^+$ formed via electron transfer (see Supplementary Fig. 41 below); (b) linear Stern-Volmer plot based on lifetime quenching derived from the decays in (a).

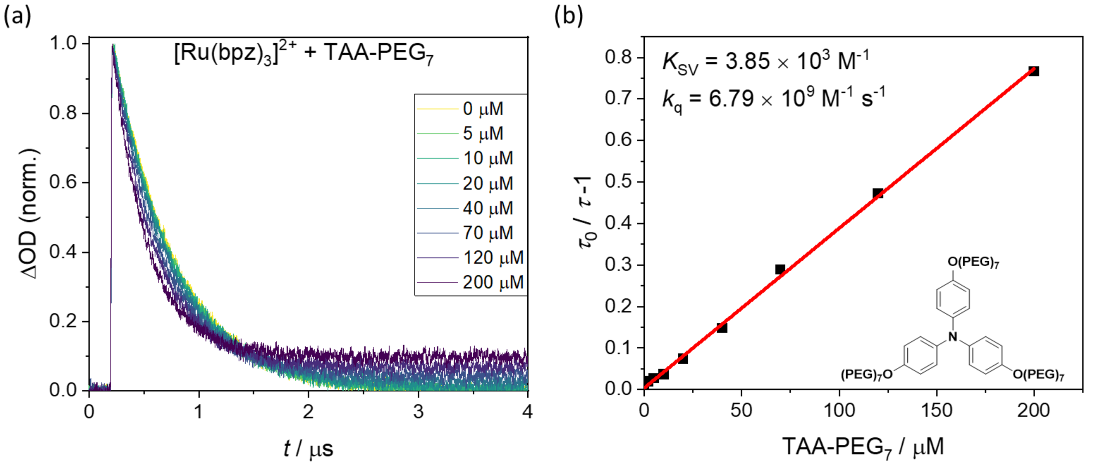

Supplementary Fig. 18: Stern-Volmer-type studies of $[Ru(bpz)_3]^{2+}$ (20 µM) in aerated CH_3CN at 20 °C with TAA-PEG$_7$: (a) normalized transient absorption decay of ^{3}MLCT-excited $[Ru(bpz)_3]^{2+}$ at 440 nm (ground state bleach; signals multiplied by a factor of -1) with increasing concentration of TAA-PEG$_7$ under excitation with a ~ 10 ns pulsed 450 nm laser (pulse energy ~ 12 mJ); mono-exponential fit of the decays yields lifetime τ. The residual long-lived signal, which becomes increasingly prominent at elevated TAA-PEG$_7$ concentrations, is attributed to the transient absorption signal of $[Ru(bpz)_3]^+$ formed via electron transfer (see Supplementary Fig. 41 below); (b) linear Stern-Volmer plot based on lifetime quenching derived from the decays in (a).

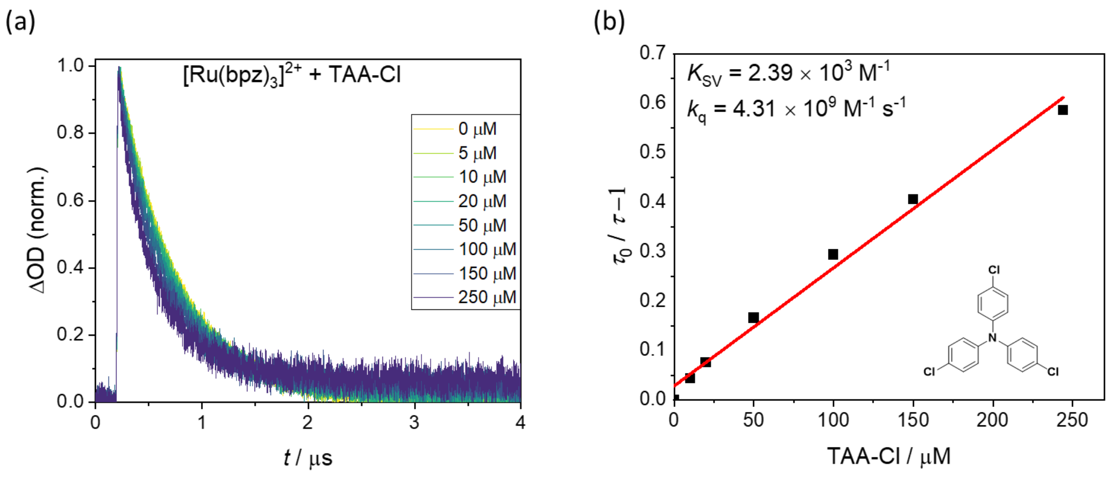

Supplementary Fig. 19: Stern-Volmer-type studies of $[Ru(bpz)_3]^{2+}$ (20 μM) in aerated CH_3CN at 20 °C with TAA-Cl: (a) normalized transient absorption decay of ^{3}MLCT-excited $[Ru(bpz)_3]^{2+}$ at 440 nm (ground state bleach; signals multiplied by a factor of -1) with increasing concentration of TAA-Cl under excitation with a ~ 10 ns pulsed 450 nm laser (pulse energy ~ 11 mJ); mono-exponential fit of the decays yields lifetime τ. The residual long-lived signal, which becomes increasingly prominent at elevated TAA-Cl concentrations, is attributed to the transient absorption signal of $[Ru(bpz)_3]^{+}$ formed via electron transfer (see Supplementary Fig. 41 below); (b) linear Stern-Volmer plot based on lifetime quenching derived from the decays in (a).

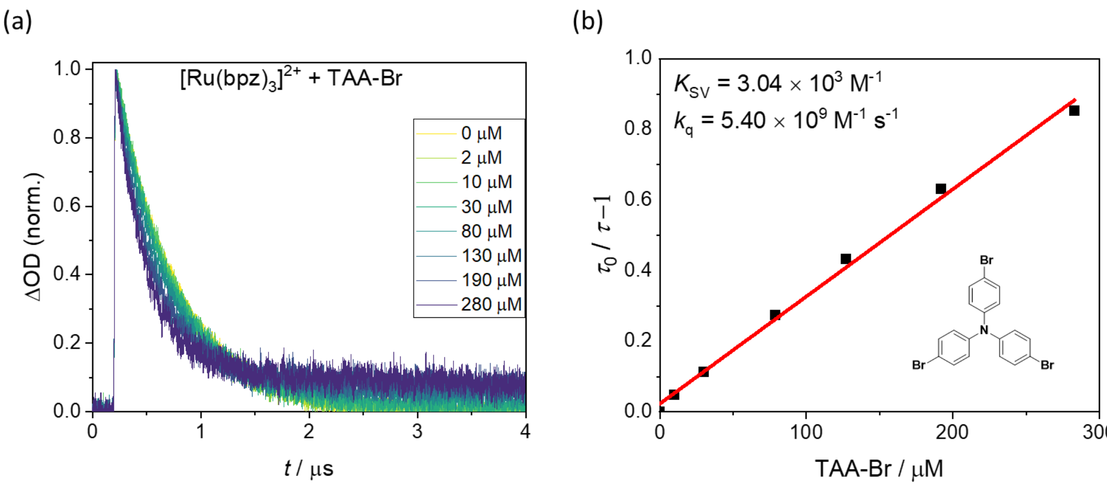

Supplementary Fig. 20: Stern-Volmer-type studies of $[Ru(bpz)_3]^{2+}$ (20 μM) in aerated CH_3CN at 20 °C with TAA-Br: (a) normalized transient absorption decay of ^{3}MLCT-excited $[Ru(bpz)_3]^{2+}$ at 440 nm (ground state bleach; signals multiplied by a factor of -1) with increasing concentration of TAA-Br under excitation with a ~ 10 ns pulsed 450 nm laser (pulse energy ~ 10 mJ); mono-exponential fit of the decays yields lifetime τ. The residual long-lived signal, which becomes increasingly prominent at elevated TAA-Br concentrations, is attributed to the transient absorption signal of $[Ru(bpz)_3]^{+}$ formed via electron transfer (see Supplementary Fig. 41 below); (b) linear Stern-Volmer plot based on lifetime quenching derived from the decays in (a).

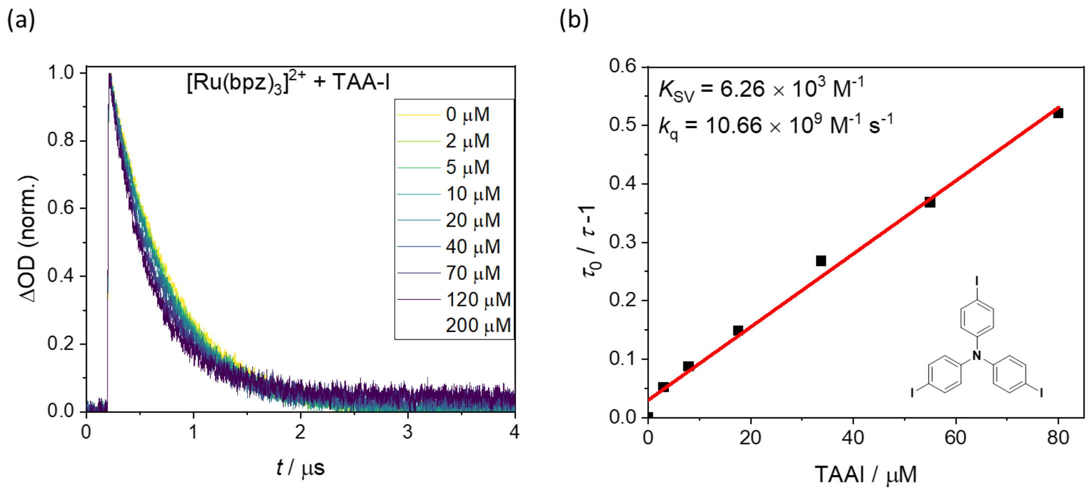

Supplementary Fig. 21: Stern-Volmer-type studies of $[Ru(bpz)_3]^{2+}$ (20 µM) in aerated CH_3CN at 20 °C with TAA-I: (a) normalized transient absorption decay of ^{3}MLCT-excited $[Ru(bpz)_3]^{2+}$ at 440 nm (ground state bleach; signals multiplied by a factor of -1) with increasing concentration of TAA-I under excitation with a ~ 10 ns pulsed 450 nm laser (pulse energy ~ 9 mJ); mono-exponential fit of the decays yields lifetime τ. The residual long-lived signal, which becomes increasingly prominent at elevated TAA-I concentrations, is attributed to the transient absorption signal of $[Ru(bpz)_3]^+$ formed via electron transfer (see Supplementary Fig. 41 below); (b) linear Stern-Volmer plot based on lifetime quenching derived from the decays in (a).

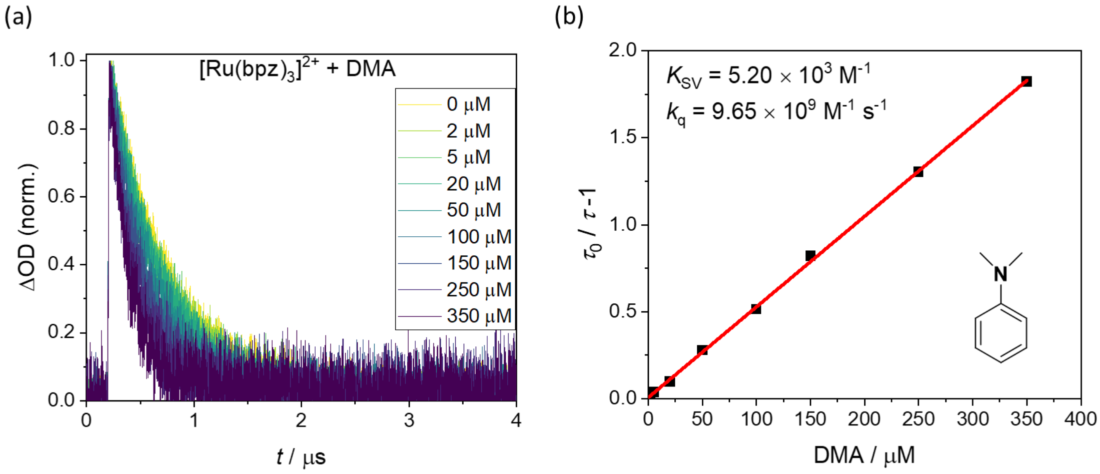

Supplementary Fig. 22: Stern-Volmer-type studies of $[Ru(bpz)_3]^{2+}$ (20 µM) in aerated CH_3CN at 20 °C with DMA: (a) normalized transient absorption decay of ^{3}MLCT-excited $[Ru(bpz)_3]^{2+}$ at 440 nm (ground state bleach; signals multiplied by a factor of -1) with increasing concentration of DMA under excitation with a ~ 10 ns pulsed 450 nm laser (pulse energy ~ 6 mJ); mono-exponential fit of the decays yields lifetime τ; (b) linear Stern-Volmer plot based on lifetime quenching derived from the decays in (a).

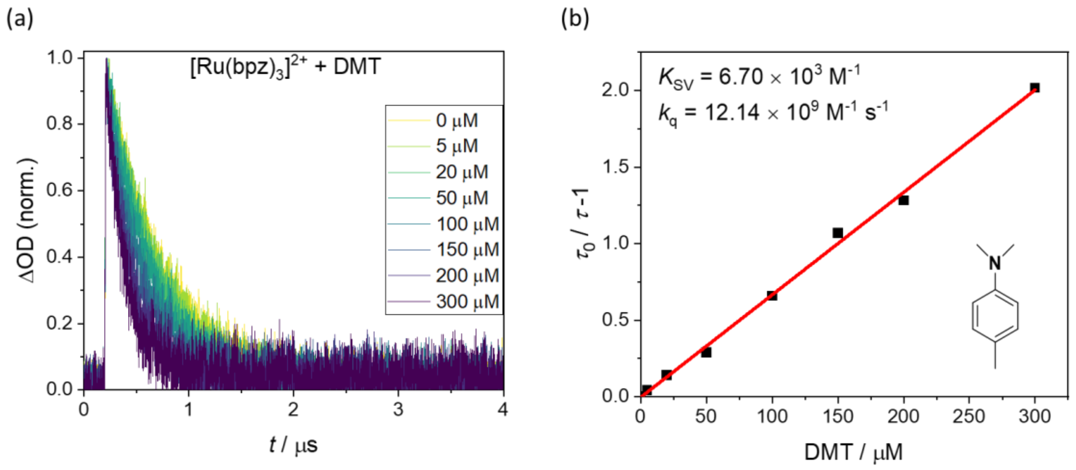

Supplementary Fig. 23: Stern-Volmer-type studies of $[Ru(bpz)_3]^{2+}$ (20 µM) in aerated CH_3CN at 20 °C with DMT: (a) normalized transient absorption decay of ^{3}MLCT-excited $[Ru(bpz)_3]^{2+}$ at 440 nm (ground state bleach; signals multiplied by a factor of -1) with increasing concentration of DMT under excitation with a ~ 10 ns pulsed 450 nm laser (pulse energy ~ 6 mJ); mono-exponential fit of the decays yields lifetime τ; (b) linear Stern-Volmer plot based on lifetime quenching derived from the decays in (a).

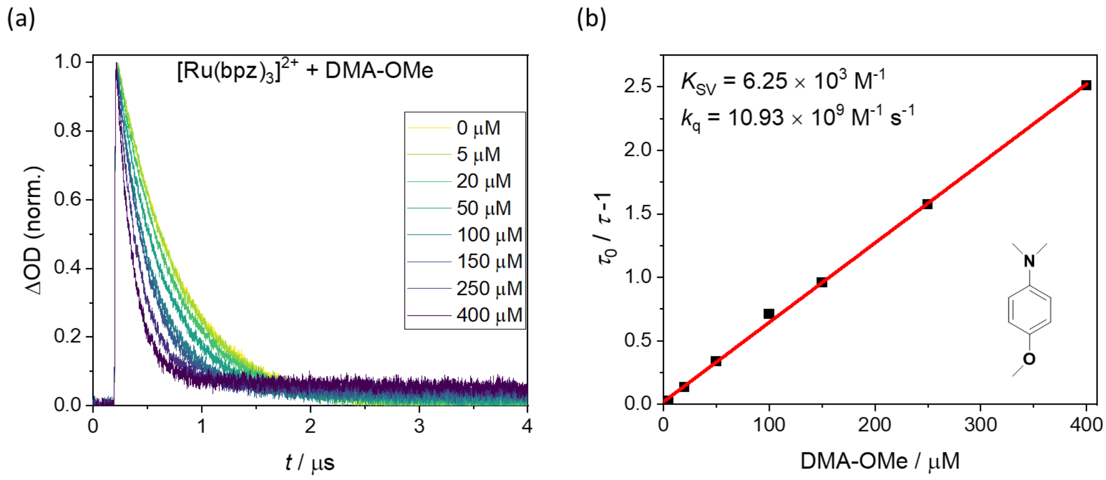

Supplementary Fig. 24: Stern-Volmer-type studies of $[Ru(bpz)_3]^{2+}$ (20 µM) in aerated CH_3CN at 20 °C with DMA-OMe: (a) normalized transient absorption decay of ^{3}MLCT-excited $[Ru(bpz)_3]^{2+}$ at 440 nm (ground state bleach; signals multiplied by a factor of -1) with increasing concentration of DMA-OMe under excitation with a ~ 10 ns pulsed 450 nm laser (pulse energy ~ 14 mJ); mono-exponential fit of the decays yields lifetime τ. The residual long-lived signal, which becomes increasingly prominent at elevated DMA-OMe concentrations, is attributed to the transient absorption signal of $[Ru(bpz)_3]^{+}$ formed via electron transfer (see Supplementary Fig. 41 below); (b) linear Stern-Volmer plot based on lifetime quenching derived from the decays in (a).

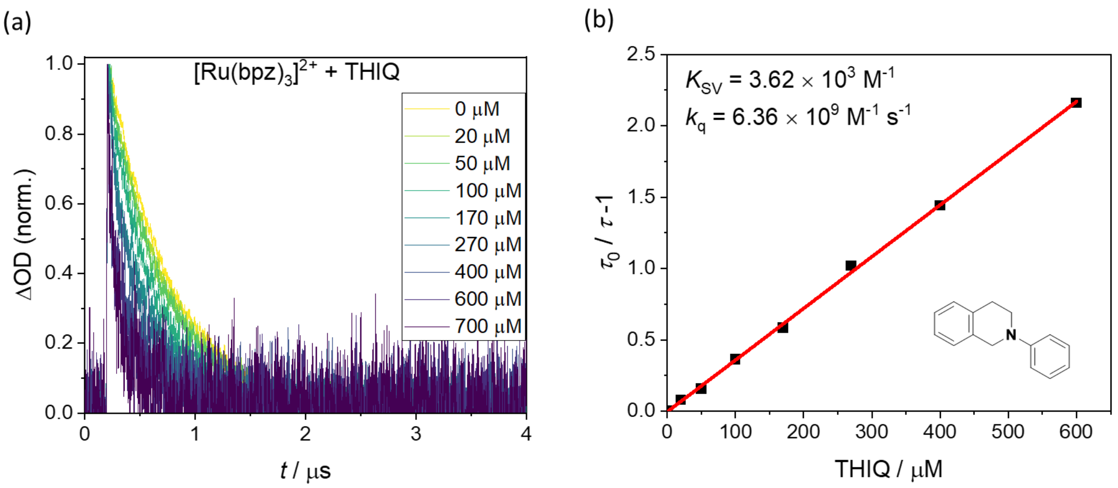

Supplementary Fig. 25: Stern-Volmer-type studies of $[Ru(bpz)_3]^{2+}$ (20 μM) in aerated CH_3CN at 20 °C with THIQ: (a) normalized transient absorption decay of ^{3}MLCT-excited $[Ru(bpz)_3]^{2+}$ at 440 nm (ground state bleach; signals multiplied by a factor of -1) with increasing concentration of THIQ under excitation with a ~ 10 ns pulsed 450 nm laser (pulse energy ~ 10 mJ); mono-exponential fit of the decays yields lifetime τ; (b) linear Stern-Volmer plot based on lifetime quenching derived from the decays in (a).

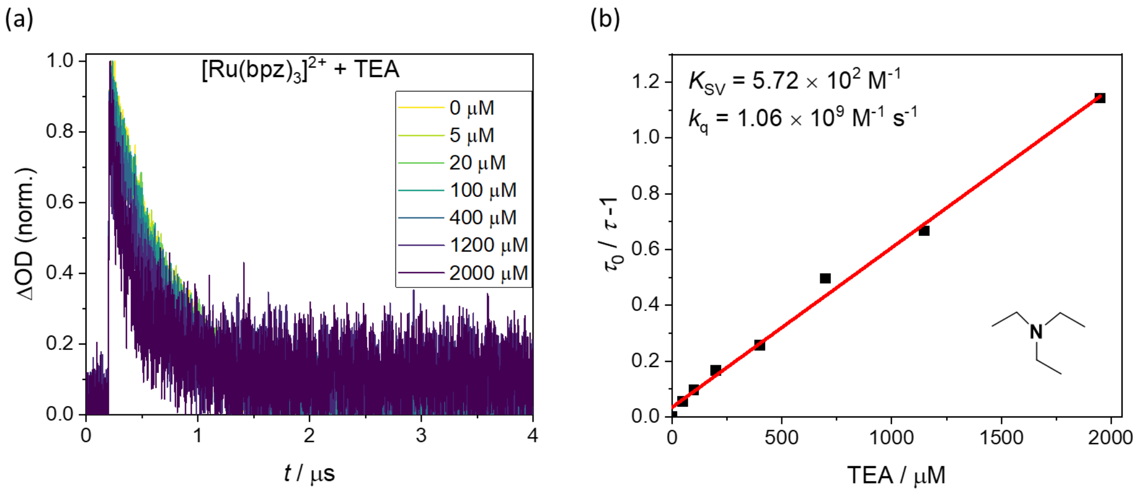

Supplementary Fig. 26: Stern-Volmer-type studies of $[Ru(bpz)_3]^{2+}$ (20 μM) in aerated CH_3CN at 20 °C with TEA: (a) normalized transient absorption decay of ^{3}MLCT-excited $[Ru(bpz)_3]^{2+}$ at 440 nm (ground state bleach; signals multiplied by a factor of -1) with increasing concentration of TEA under excitation with a ~ 10 ns pulsed 450 nm laser (pulse energy ~ 6 mJ); mono-exponential fit of the decays yields lifetime τ. The residual long-lived signal, which becomes increasingly prominent at elevated TEA concentrations, is attributed to the transient absorption signal of $[Ru(bpz)_3]^{+}$ formed via electron transfer (see Supplementary Fig. 41 below); (b) linear Stern-Volmer plot based on lifetime quenching derived from the decays in (a).

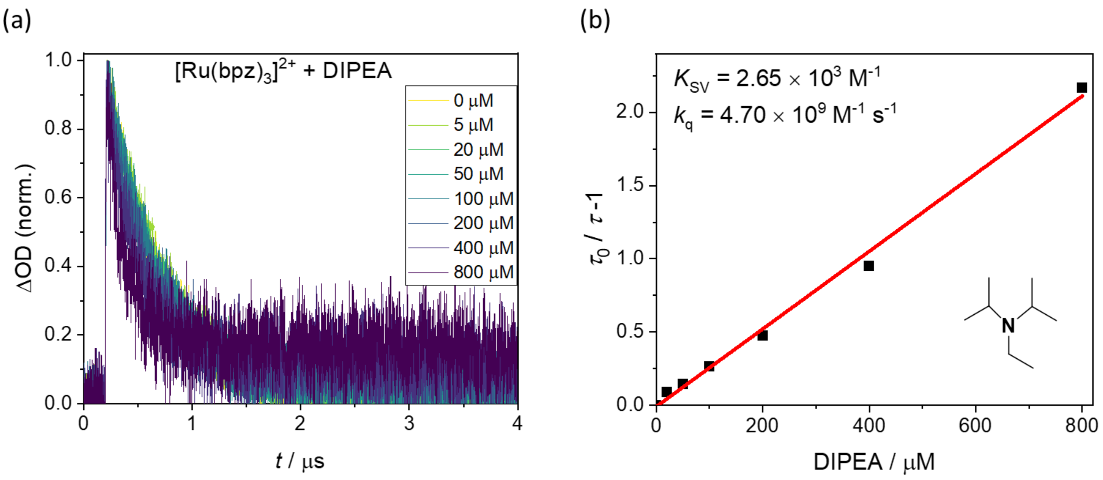

Supplementary Fig. 27: Stern-Volmer-type studies of $[Ru(bpz)_3]^{2+}$ (20 µM) in aerated CH_3CN at 20 °C with DIPEA: (a) normalized transient absorption decay of ^{3}MLCT-excited $[Ru(bpz)_3]^{2+}$ at 440 nm (ground state bleach; signals multiplied by a factor of -1) with increasing concentration of DIPEA under excitation with a ~ 10 ns pulsed 450 nm laser (pulse energy ~ 6 mJ); mono-exponential fit of the decays yields lifetime τ. The residual long-lived signal, which becomes increasingly prominent at elevated DIPEA concentrations, is attributed to the transient absorption signal of $[Ru(bpz)_3]^{+}$ formed via electron transfer (see Supplementary Fig. 41 below); (b) linear Stern-Volmer plot based on lifetime quenching derived from the decays in (a).

5.2 Quenching of $[Cr(dqp)_2]^{3+}$

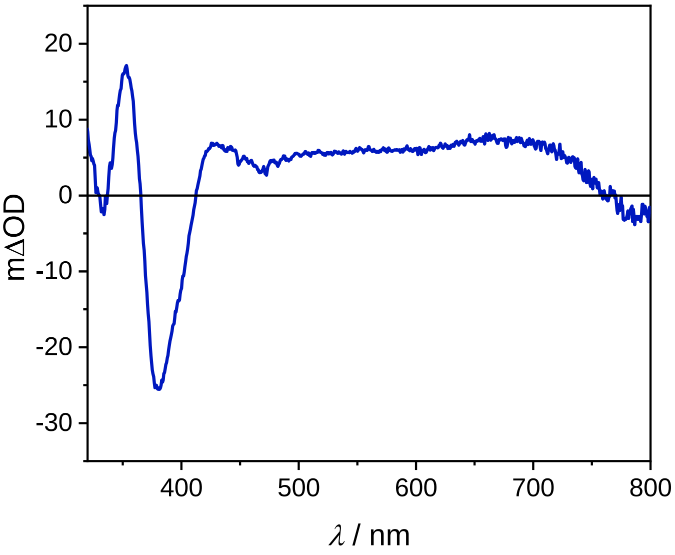

Supplementary Fig. 28: Transient absorption spectrum of $[Cr(dqp)_2]^{3+}$ (30 µM) in aerated CH_3CN under 415 nm pulsed laser excitation (pulse energy of ~ 7 mJ) at 20 °C. The spectrum was recorded with 10 ns time delay after the pulses, and the signal was time-integrated for 200 ns.

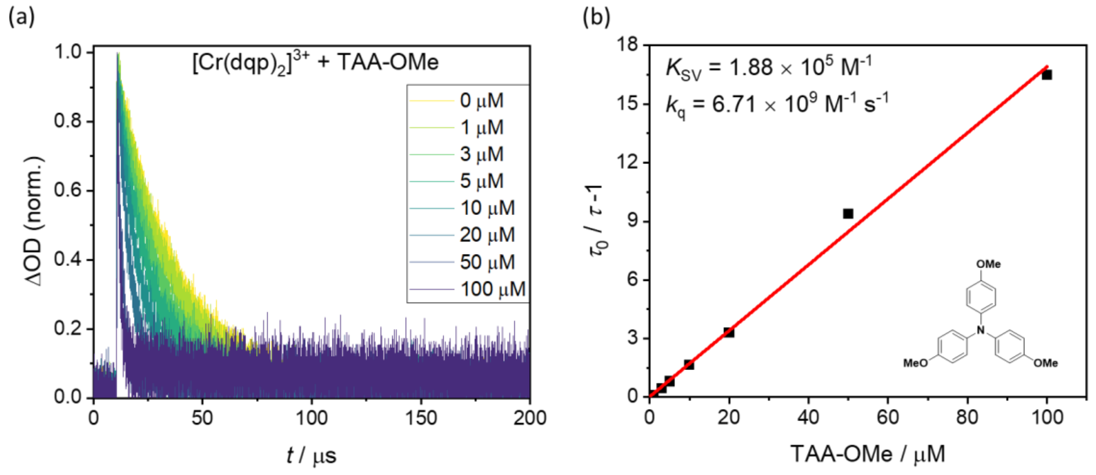

Supplementary Fig. 29: Stern-Volmer-type studies of $[Cr(dqp)_2]^{3+}$ (50 µM) in aerated CH_3CN at 20 °C with TAA-OMe: (a) normalized transient absorption decay of $[Cr(dqp)_2]^{3+}$ at 435 nm (excited state absorption signal) as a function of increasing concentration of TAA-OMe. Excitation occurred with a ~ 10 ns pulsed 425 nm laser (pulse energy ~ 9 mJ); mono-exponential fit of the decays yields lifetime τ; (b) linear Stern-Volmer plot based on the lifetime quenching derived from the decays in (a).

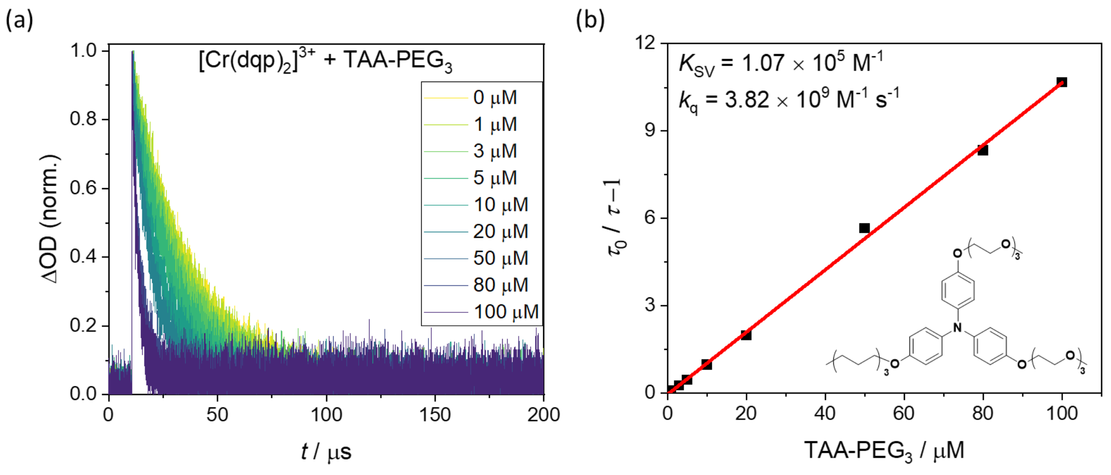

Supplementary Fig. 30: Stern-Volmer-type studies of $[Cr(dqp)_2]^{3+}$ (50 µM) in aerated CH_3CN at 20 °C with TAA-PEG_3: (a) normalized transient absorption decay of $[Cr(dqp)_2]^{3+}$ at 435 nm (excited state absorption signal) as a function of increasing concentration of TAA-PEG_3. Excitation occurred with a ~ 10 ns pulsed 425 nm laser (pulse energy ~ 9 mJ); mono-exponential fit of the decays yields lifetime τ; (b) linear Stern-Volmer plot based on the lifetime quenching derived from the decays in (a).

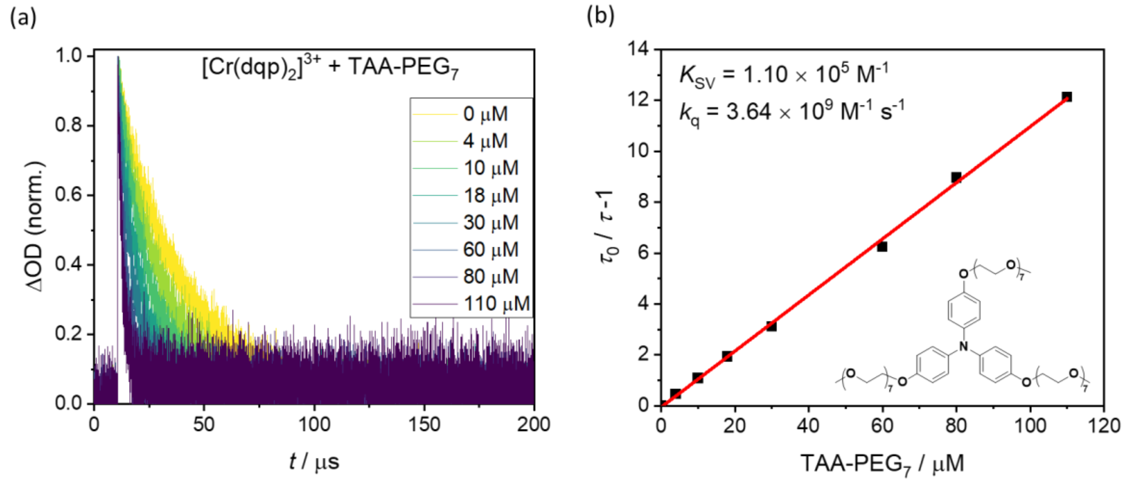

Supplementary Fig. 31: Stern-Volmer-type studies of $[Cr(dqp)_2]^{3+}$ (50 µM) in aerated CH_3CN at 20 °C with TAA-PEG$_7$: (a) normalized transient absorption decay of $[Cr(dqp)_2]^{3+}$ at 435 nm (excited state absorption signal) as a function of increasing concentration of TAA-PEG$_7$. Excitation occurred with a ~ 10 ns pulsed 425 nm laser (pulse energy ~ 9 mJ); mono-exponential fit of the decays yields lifetime τ; (b) linear Stern-Volmer plot based on the lifetime quenching derived from the decays in (a).

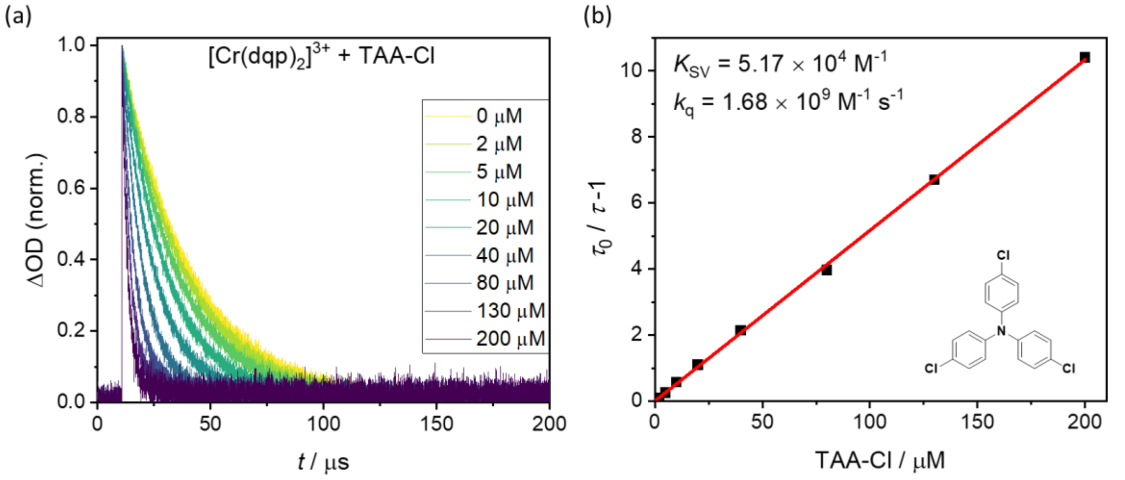

Supplementary Fig. 32: Stern-Volmer-type studies of $[Cr(dqp)_2]^{3+}$ (50 µM) in aerated CH_3CN at 20 °C with TAA-Cl: (a) normalized transient absorption decay of $[Cr(dqp)_2]^{3+}$ at 435 nm (excited state absorption signal) as a function of increasing concentration of TAA-Cl. Excitation occurred with a ~ 10 ns pulsed 425 nm laser (pulse energy ~ 9 mJ); mono-exponential fit of the decays yields lifetime τ; (b) linear Stern-Volmer plot based on the lifetime quenching derived from the decays in (a).

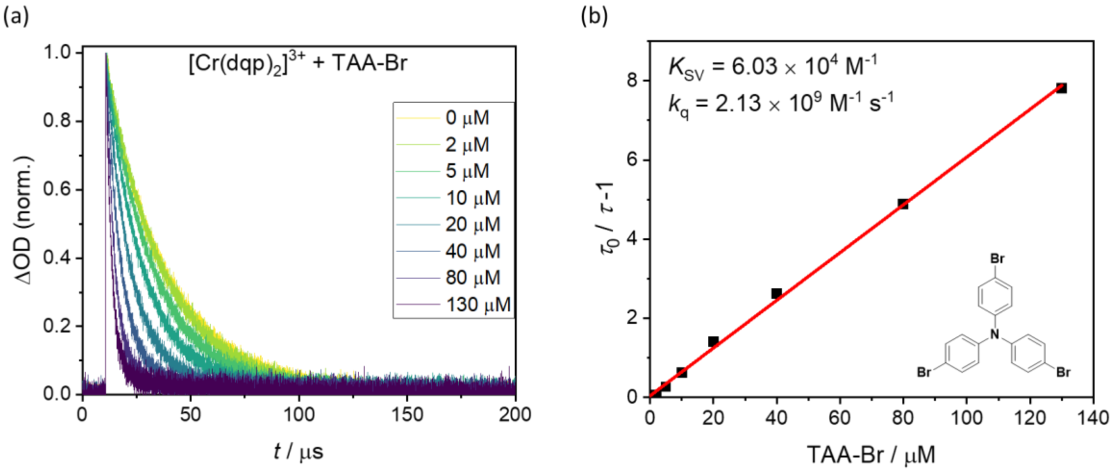

Supplementary Fig. 33: Stern-Volmer-type studies of $[Cr(dqp)_2]^{3+}$ (50 μM) in aerated CH_3CN at 20 °C with TAA-Br: (a) normalized transient absorption decay of $[Cr(dqp)_2]^{3+}$ at 435 nm (excited state absorption signal) as a function of increasing concentration of TAA-Br. Excitation occurred with a ~ 10 ns pulsed 425 nm laser (pulse energy ~ 9 mJ); mono-exponential fit of the decays yields lifetime τ; (b) linear Stern-Volmer plot based on the lifetime quenching derived from the decays in (a).

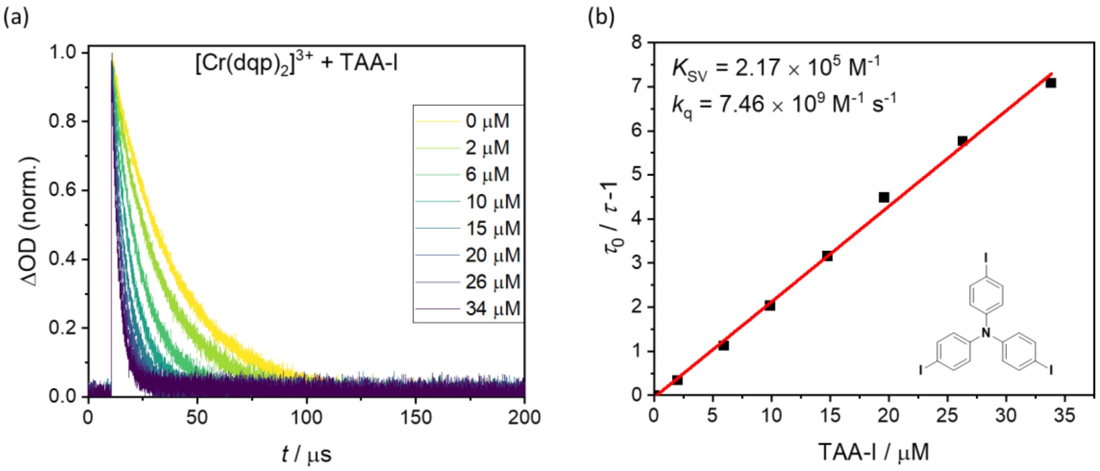

Supplementary Fig. 34: Stern-Volmer-type studies of $[Cr(dqp)_2]^{3+}$ (50 μM) in aerated CH_3CN at 20 °C with TAA-I: (a) normalized transient absorption decay of $[Cr(dqp)_2]^{3+}$ at 435 nm (excited state absorption signal) as a function of increasing concentration of TAA-I. Excitation occurred with a ~ 10 ns pulsed 425 nm laser (pulse energy ~ 9 mJ); mono-exponential fit of the decays yields lifetime τ; (b) linear Stern-Volmer plot based on the lifetime quenching derived from the decays in (a).

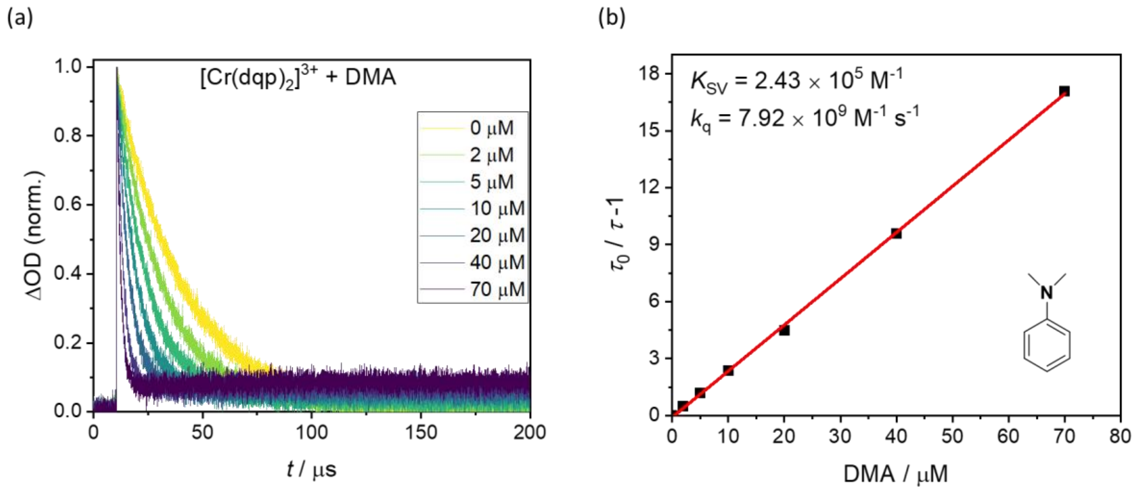

Supplementary Fig. 35: Stern-Volmer-type studies of $[Cr(dqp)_2]^{3+}$ (50 μM) in aerated CH_3CN at 20 °C with DMA: (a) normalized transient absorption decay of $[Cr(dqp)_2]^{3+}$ at 435 nm (excited state absorption signal) as a function of increasing concentration of DMA. Excitation occurred with a ~ 10 ns pulsed 425 nm laser (pulse energy ~ 10 mJ); mono-exponential fit of the decays yields lifetime τ. The residual long-lived signal, which becomes increasingly prominent at elevated DMA concentrations, is attributed to $DMA^{•+}$, which absorbs at 435 nm (see Supplementary Fig. 12); (b) linear Stern-Volmer plot based on the lifetime quenching derived from the decays in (a).

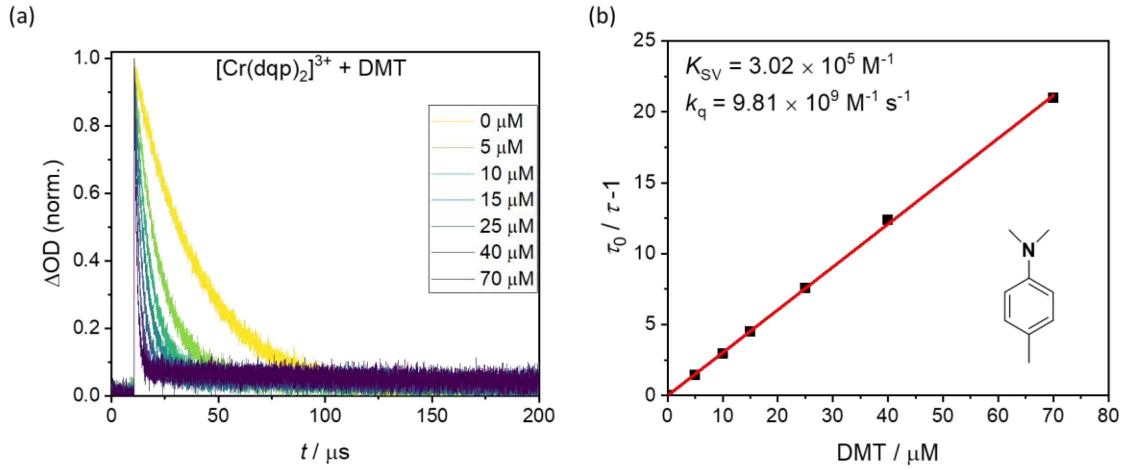

Supplementary Fig. 36: Stern-Volmer-type studies of $[Cr(dqp)_2]^{3+}$ (50 μM) in aerated CH_3CN at 20 °C with DMT: (a) normalized transient absorption decay of $[Cr(dqp)_2]^{3+}$ at 435 nm (excited state absorption signal) as a function of increasing concentration of DMA. Excitation occurred with a ~ 10 ns pulsed 425 nm laser (pulse energy ~ 10 mJ); mono-exponential fit of the decays yields lifetime τ. The residual long-lived signal, which becomes increasingly prominent at elevated DMT concentrations, is attributed to $DMT^{•+}$, which absorbs at 435 nm;[16] (b) linear Stern-Volmer plot based on the lifetime quenching derived from the decays in (a).

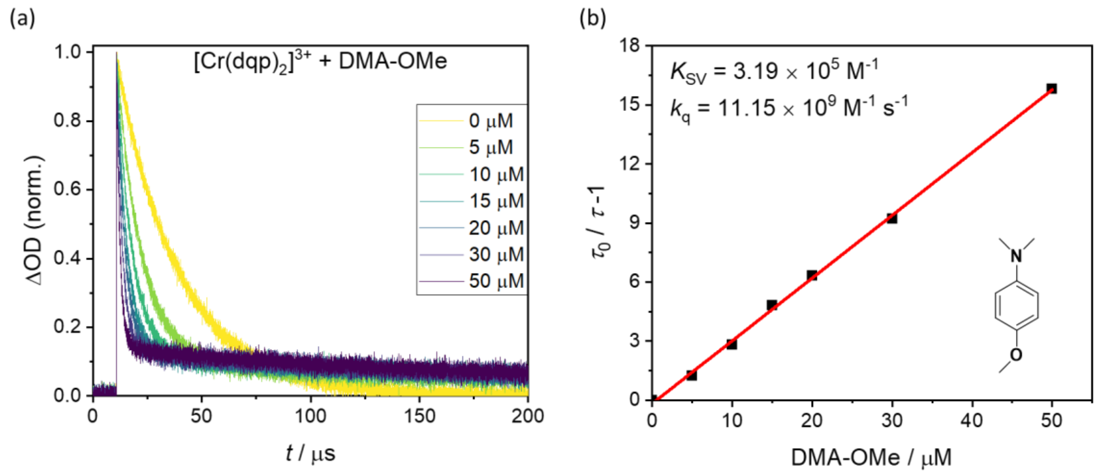

Supplementary Fig. 37: Stern-Volmer-type studies of $[Cr(dqp)_2]^{3+}$ (50 µM) in aerated CH_3CN at 20 °C with DMA-OMe: (a) normalized transient absorption decay of $[Cr(dqp)_2]^{3+}$ at 435 nm (excited state absorption signal) as a function of increasing concentration of DMA-OMe. Excitation occurred with a ~ 10 ns pulsed 425 nm laser (pulse energy ~ 10 mJ); mono-exponential fit of the decays yields lifetime τ. The residual long-lived signal, which becomes increasingly prominent at elevated DMA-OMe concentrations, is attributed to DMA-OMe$^{\bullet+}$, which absorbs at 435 nm (see Supplementary Fig. 13); (b) linear Stern-Volmer plot based on the lifetime quenching derived from the decays in (a).

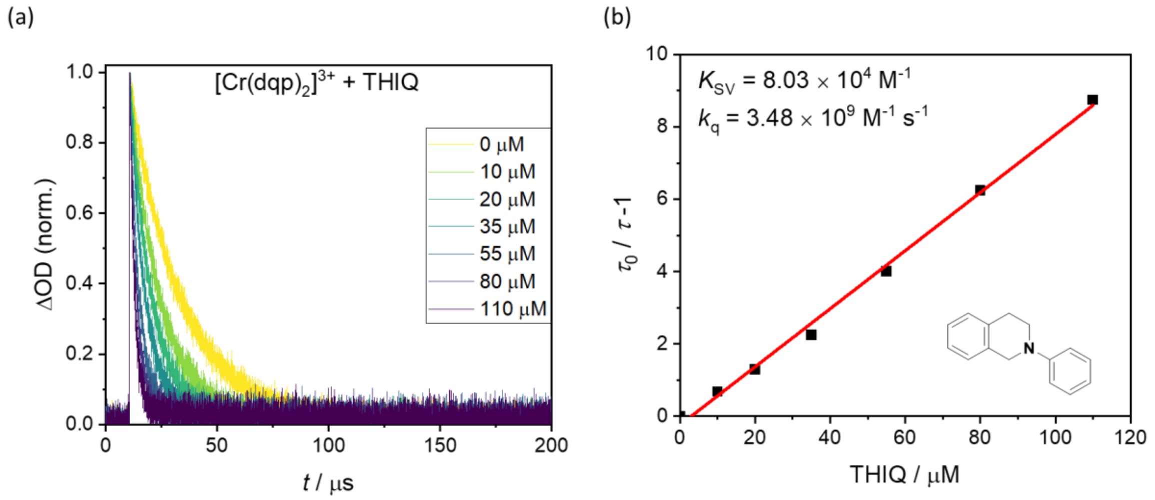

Supplementary Fig. 38: Stern-Volmer-type studies of $[Cr(dqp)_2]^{3+}$ (50 µM) in aerated CH_3CN at 20 °C with THIQ: (a) normalized transient absorption decay of $[Cr(dqp)_2]^{3+}$ at 435 nm (excited state absorption signal) as a function of increasing concentration of THIQ. Excitation occurred with a ~ 10 ns pulsed 425 nm laser (pulse energy ~ 10 mJ); mono-exponential fit of the decays yields lifetime τ; (b) linear Stern-Volmer plot based on the lifetime quenching derived from the decays in (a).

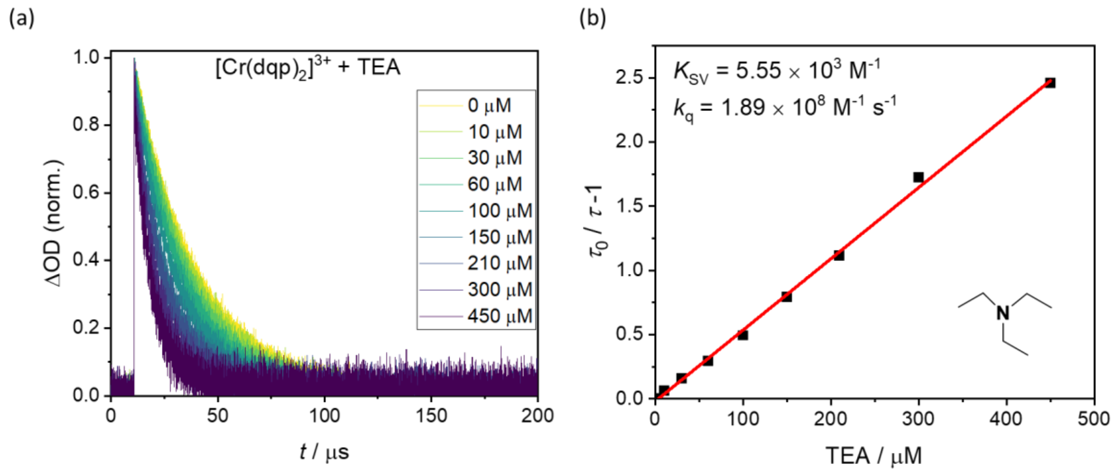

Supplementary Fig. 39: Stern-Volmer-type studies of $[Cr(dqp)_2]^{3+}$ (50 µM) in aerated CH_3CN at 20 °C with TEA: (a) normalized transient absorption decay of $[Cr(dqp)_2]^{3+}$ at 435 nm (excited state absorption signal) as a function of increasing concentration of TEA. Excitation occurred with a ~ 10 ns pulsed 425 nm laser (pulse energy ~ 10 mJ); mono-exponential fit of the decays yields lifetime τ; (b) linear Stern-Volmer plot based on the lifetime quenching derived from the decays in (a).

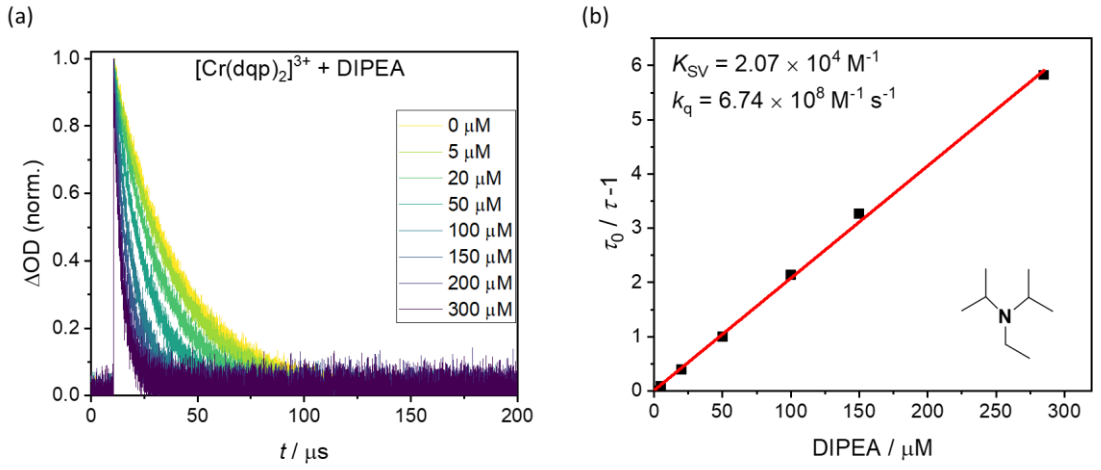

Supplementary Fig. 40: Stern-Volmer-type studies of $[Cr(dqp)_2]^{3+}$ (50 µM) in aerated CH_3CN at 20 °C with DIPEA: (a) normalized transient absorption decay of $[Cr(dqp)_2]^{3+}$ at 435 nm (excited state absorption signal) as a function of increasing concentration of DIPEA. Excitation occurred with a ~ 10 ns pulsed 425 nm laser (pulse energy ~ 10 mJ); mono-exponential fit of the decays yields lifetime τ; (b) linear Stern-Volmer plot based on the lifetime quenching derived from the decays in (a).

6. Determination of changes in extinction coefficients *Δε*

6.1 Δ*ε* of $[Ru(bpz)_3]^+$

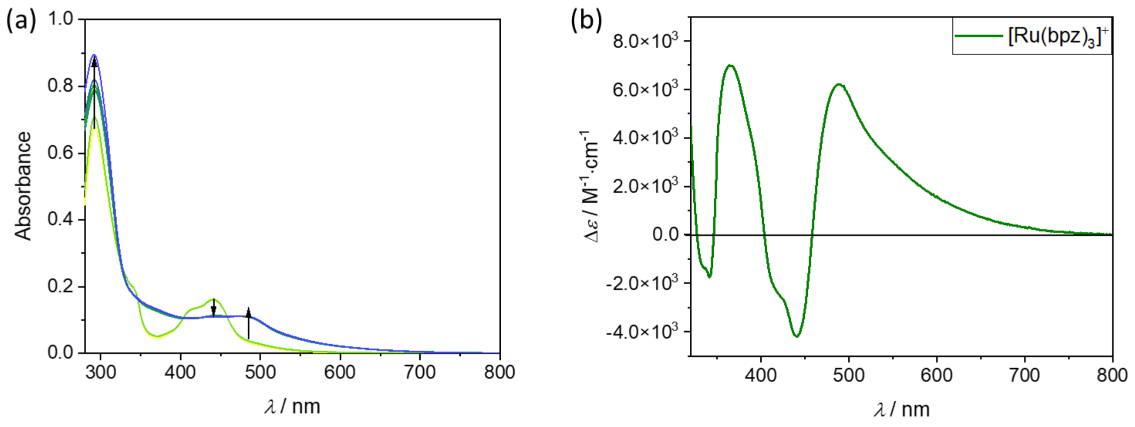

Supplementary Fig. 41: (a) UV-vis absorption spectrum of $[Ru(bpz)_3]^{2+}$ (13 µM) in the absence of triethylamine (TEA) in aerated CH_3CN at 20 °C (light green trace) and spectra obtained after photochemical reduction of $[Ru(bpz)_3]^{2+}$ by TEA (20 mM). Upon 418 nm pulsed laser irradiation of the solution containing TEA (pulse energy of ca. 10 mJ), changes in the absorption spectra were observed (black arrows) due to the formation of $[Ru(bpz)_3]^+$. Absorption spectra were recorded until no further changes were observed with continued laser irradiation (light green to dark blue trace), *i.e.* until $[Ru(bpz)_3]^{2+}$ was completely reduced to $[Ru(bpz)_3]^+$. The obtained final spectrum (dark blue) resembles that previously reported (even if the solvent was different).[17,18] (b) Changes in extinction coefficients Δ*ε* of $[Ru(bpz)_3]^+$ relative to $[Ru(bpz)_3]^{2+}$ in aerated CH_3CN, derived from the difference in absorption spectra obtained before and after photochemical reduction by TEA (light green and dark blue traces) in (a), using the known sample concentration. This procedure yields a Δ*ε* value of ~ 6000 M^{-1} cm^{-1} at the maximum of 490 nm in CH_3CN, which is lower than the previously reported value in water (~ 10000 M^{-1} cm^{-1}).[17]

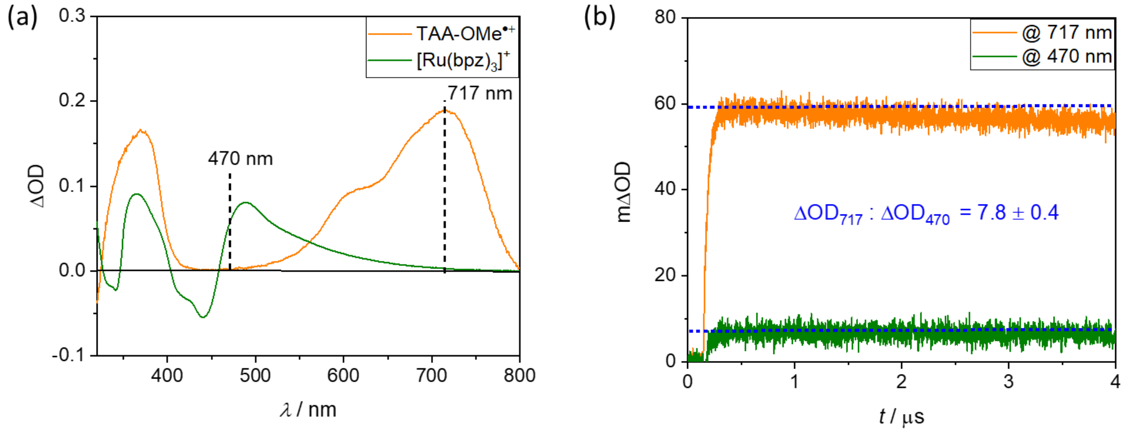

Supplementary Fig. 42: (a) Absorbance change associated with the (photochemical) formation of $[Ru(bpz)_3]^+$ from $[Ru(bpz)_3]^{2+}$ (green solid line, obtained by subtracting the yellow trace from the dark blue trace in Supplementary Fig. 41a) and the spectro-electrochemical absorption difference spectrum of the radical cation TAA-OMe$^{•+}$ (red solid line, from Supplementary Fig. 6). (b) Transient absorption decay kinetics measured at 717 nm (red line) and at 470 nm (green line) of $[Ru(bpz)_3]^{2+}$(13 µM) in the presence of TAA-OMe (2 mM) in aerated CH_3CN under pulsed laser excitation at 422 nm (pulse energy of ~ 8 mJ).

The change in molar extinction coefficient at 470 nm ($\Delta\varepsilon_{470}$) associated with the reduction of $[Ru(bpz)_3]^{2+}$ to $[Ru(bpz)_3]^+$ was determined independently with two different methods: I) direct photochemical reduction of the $[Ru(bpz)_3]^{2+}$ complex by triethylamine (TEA) under pulsed laser irradiation at 418 nm causes the spectral changes seen in **Error! Reference source not found.**a. After c

omplete reduction of the complex, $\Delta\varepsilon_{470}$ was calculated based on the known initial concentration of $[Ru(bpz)_3]^{2+}$ (**Error! Reference source not found.**b), yielding a $\Delta\varepsilon_{470}$ value of ~ 4300 M^{-1} cm^{-1}; II) TAA-O Me$^{•+}$ is optically essentially transparent at 470 nm, whereas $[Ru(bpz)_3]^{+}$ shows negligible extinction at 717 nm (**Error! Reference source not found.**a). Following selective excitation of $[Ru(bpz)_3]^{2+}$ (13 µM) at 422 nm in aerated CH_3CN containing TAA-OMe (2 mM), the TAA-OMe$^{•+}$ and $[Ru(bpz)_3]^{+}$ photoproducts are formed in 1:1 ratio and can be monitored independently at 717 and 470 nm. The obtained ratio of ΔOD values at 717 nm and 470 nm (7.8 ± 0.4, see **Error! Reference source not found.**b) r eflects the ratio of their respective extinction coefficient at the respective two observation wavelengths. Using the literature-known extinction coefficient of TAA-OMe$^{•+}$ at 717 nm in CH_3CN (32800 M^{-1} cm^{-1}),[19] $\Delta\varepsilon_{470}$ is determined to be 4200 ± 200 M^{-1} cm^{-1}. The agreement of $\Delta\varepsilon_{470}$ values obtained by the two independent methods validates the relative method (method II) for the determination of $\Delta\varepsilon$ values.

In **Error! Reference source not found.**b, the relevant ΔOD_{717} and ΔOD_{470} values were determined at t he maxima of the respective rise-and-decay curves. The initial rises reflect the formation of TAA-OMe$^{•+}$ and $[Ru(bpz)_3]^{+}$ by photoinduced electron transfer, whereas the ensuing (slower) decays reflect thermal reverse electron transfer to reinstate the TAA-OMe and $[Ru(bpz)_3]^{2+}$ starting materials. The same method has been applied to all following systems presented in the forthcoming subsections.

6.2 $\Delta\varepsilon$ of $[Cr(dqp)_2]^{2+}$

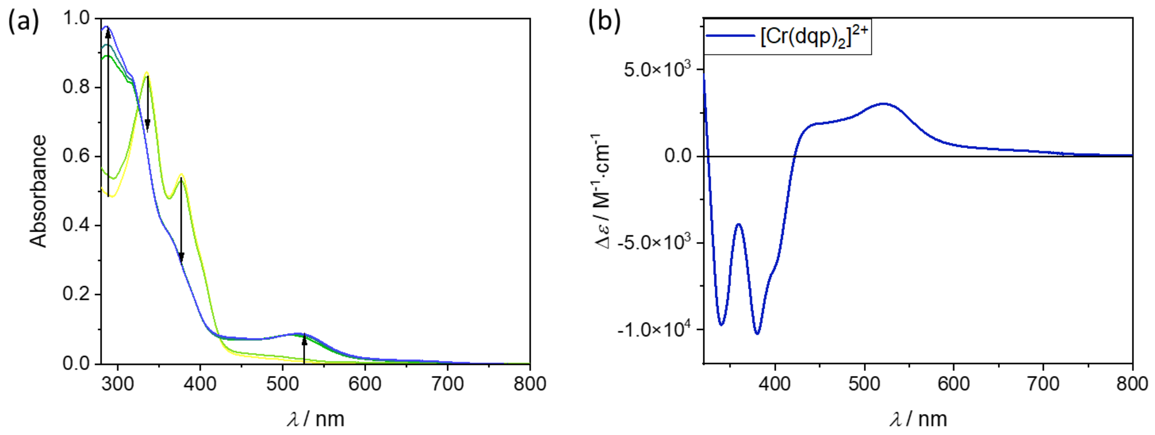

Supplementary Fig. 43*:* (a) UV-vis absorption spectrum of $[Cr(dqp)_2]^{3+}$ (30 µM) in the absence of triethylamine (TEA) in aerated CH_3CN at 20 °C (yellow trace) and spectra obtained after photochemical reduction of $[Cr(dqp)_2]^{3+}$ by TEA (10 mM). Upon 418 nm pulsed laser irradiation of this solution (pulse energy of ca. 10 mJ), changes in the absorption spectra were observed (black arrows) due to the formation of $[Cr(dqp)_2]^{2+}$. Absorption spectra were recorded until and no further changes were observed with continued laser irradiation (green to dark blue traces), i.e. until $[Cr(dqp)_2]^{3+}$ was completely reduced to $[Cr(dqp)_2]^{2+}$. (b) Changes in extinction coefficients $\Delta\varepsilon$ of $[Cr(dqp)_2]^{2+}$ relative to $[Cr(dqp)_2]^{3+}$ in aerated CH_3CN, derived from the difference in absorption spectra recorded before and after photochemical reduction by TEA (yellow and dark blue traces) in (a), using the known sample concentration. The obtained differential absorption spectrum resembles the UV-vis spectro-electrochemical absorption spectrum of $[Cr(dqp)_2]^{3+}$ previously reported in the literature.[6]

6.3 $\Delta\varepsilon$ of TAA-OMe•+

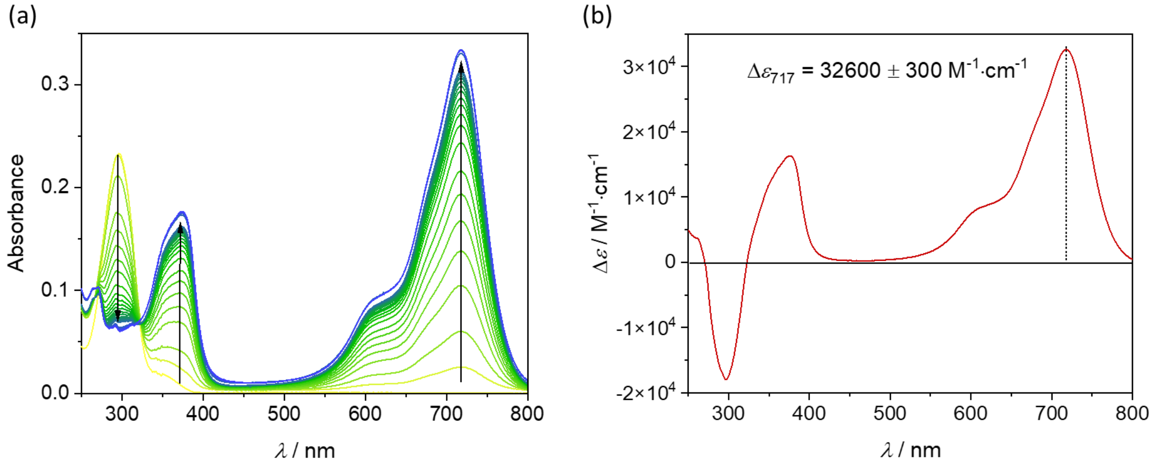

Supplementary Fig. 44: (a) UV-vis absorption spectra of TAA-OMe (30 µM) in aerated CH_3CN titrated by nitrosonium tetrafluoroborate ($NOBF_4$, concentration up to 90 µM). $NOBF_4$ has an oxidation potential of 1.25 V vs SCE in CH_3CN,[20] while the cyclic voltammogram of TAA-OMe exhibits the first oxidation peak at 0.58 V vs SCE in CH_3CN (Supplementary Fig. 1). Thus, oxidation of TAA-OMe to its radical cation (TAA-OMe$^{•+}$) by $NOBF_4$ is thermodynamically feasible in CH_3CN. Changes in absorption spectra were observed as a function of increasing amounts of added oxidant, due to the formation of chemically oxidized TAA-OMe$^{•+}$, which shows a characteristic absorption band centered at 717 nm in accordance with the literature.[19] Formation of TAA-OMe^{2+}, which features an absorption band at 520 nm in CH_3CN,[19] was not observed, suggesting that TAA-OMe$^{•+}$ is the sole oxidation product under our conditions. Absorption spectra were recorded until no further changes were observed, i.e. until all TAA-OMe was oxidized to TAA-OMe$^{•+}$. (b) Changes in molar extinction coefficients $\Delta\varepsilon$ of TAA-OMe$^{•+}$, derived from the absorption changes from (a) based on the known initial concentration of TAA-OMe. The characteristic absorption band with maximum at 717 nm was observed with $\Delta\varepsilon$ of 32600 ± 300 M^{-1} cm^{-1}, in agreement with the literature value of 32800 M^{-1} cm^{-1}.[19] The absorption features match the SEC absorption spectrum of the TAA-OMe$^{•+}$ in Supplementary Fig. 6.

6.4 $\Delta\varepsilon$ of TAA-PEG$_3^{\bullet+}$

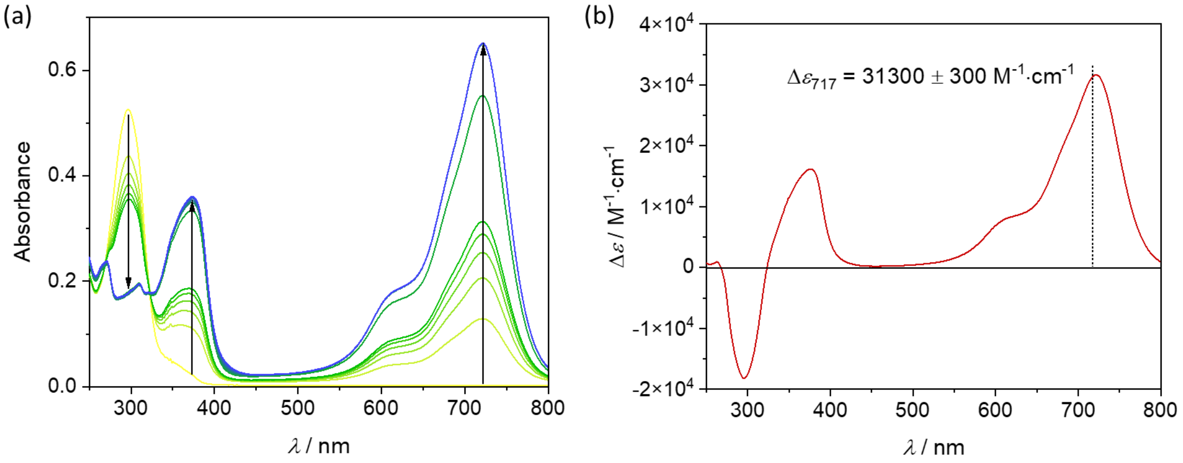

Supplementary Fig. 45: (a) UV-vis absorption spectra of TAA-PEG$_3$ (20 µM) in aerated CH_3CN titrated by nitrosonium tetrafluoroborate ($NOBF_4$, concentration up to 150 µM). $NOBF_4$ has an oxidation potential of 1.25 V vs SCE in CH_3CN,[20] while the cyclic voltammogram of TAA-PEG$_3$ exhibits the first oxidation peak at 0.68 V vs SCE in CH_3CN (Supplementary Fig. 2). Thus, oxidation of TAA-PEG$_3$ to its radical cation (TAA-PEG$_3^{\bullet+}$) by $NOBF_4$ is thermodynamically feasible in CH_3CN. Changes in absorption spectra occurred as a function of increasing amounts of added oxidant, due to the formation of chemically oxidized TAA-PEG$_3^{\bullet+}$, which shows a characteristic absorption band centered at 717 nm analogously to TAA-OMe$^{\bullet+}$. Formation of TAA-PEG$_3^{2+}$, which is supposed to feature an absorption band at 520 nm in CH_3CN analogously to TAA-OMe$^{\bullet+}$,[19] was not observed, suggesting that TAA-PEG$_3^{\bullet+}$ is the sole oxidation product under our conditions. Absorption spectra were recorded until no further changes were observed, i.e. until TAA-PEG$_3$ was fully oxidized to TAA-PEG$_3^{\bullet+}$. (b) Changes in molar extinction coefficients $\Delta\varepsilon$ of TAA-PEG$_3^{\bullet+}$, derived from the absorption changes from (a) based on the known initial concentration of TAA-PEG$_3$. The characteristic absorption band with maximum at 717 nm was observed with $\Delta\varepsilon$ of 31300 ± 300 M^{-1} cm^{-1}, and the absorption features match the SEC absorption spectrum of the TAA-PEG$_3^{\bullet+}$ in Supplementary Fig. 7.

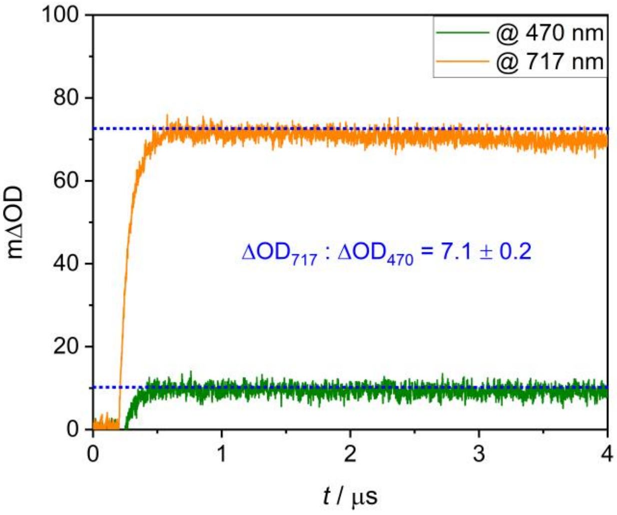

Supplementary Fig. 46: Transient absorption decay kinetics measured at 717 nm (orange line) and at 470 nm (green line) of $[Ru(bpz)_3]^{2+}$ (13 µM) in the presence of TAA-PEG$_3$ (2 mM) in aerated CH_3CN under pulsed laser excitation at 454 nm (pulse energy ~ 12 mJ).

The $\Delta\varepsilon$ value of TAA-PEG$_3^{\bullet+}$ at 717 nm was determined using two independent methods analogously as described above for $[Ru(bpz)_3]^+$: I) quantitative chemical oxidation of TAA-PEG$_3$ to TAA-PEG$_3^{\bullet+}$ with $NOBF_4$ yields $\Delta\varepsilon_{717}$ of 31300 ± 300 M^{-1} cm^{-1} (**Error! Reference source not found.**); II) TAA-PEG$_3^{\bullet+}$ is o

ptically transparent at 470 nm (**Error! Reference source not found.**b, **Error! Reference source not found.**), whereas $[Ru(bpz)_3]^+$ has negligible absorption at 717 nm (**Error! Reference source not found.**b). Following selective excitation of $[Ru(bpz)_3]^{2+}$ (13 µM) at 454 nm in aerated CH_3CN containing TAA-PEG$_3$ (2 mM), the TAA- PEG$_3^{\bullet+}$ and $[Ru(bpz)_3]^+$ photoproducts are formed in 1:1 ratio and can be monitored independently at 717 and 470 nm. The obtained ratio of ΔOD values at 717 nm and 470 nm reflects the ratio of their respective extinction coefficients (**Error! Reference source not found.**) at the r espective wavelengths. Using the measured change of extinction coefficient associated with the reduction of $[Ru(bpz)_3]^{2+}$ to $[Ru(bpz)_3]^+$ at 470 nm in CH_3CN ($\Delta\varepsilon_{470}$ = 4300 M^{-1} cm^{-1}) (**Error! Reference s ource not found.**b), $\Delta\varepsilon_{717}$ of TAA-PEG$_3$ is determined to be 30500 ± 900 M^{-1} cm^{-1}, in line with the $\Delta\varepsilon_{717}$ value obtained via oxidation with $NOBF_4$ (31300 ± 300 M^{-1} cm^{-1}). The agreement of $\Delta\varepsilon_{717}$ values obtained by the two independent methods validates again the relative method (method II) for determination of $\Delta\varepsilon$ values for the radical species. This analysis furthermore corroborates the $\Delta\varepsilon_{470}$ value obtained for $[Ru(bpz)_3]^+$.

6.5 $\Delta\varepsilon$ of TAA-PEG$_7^{\bullet+}$

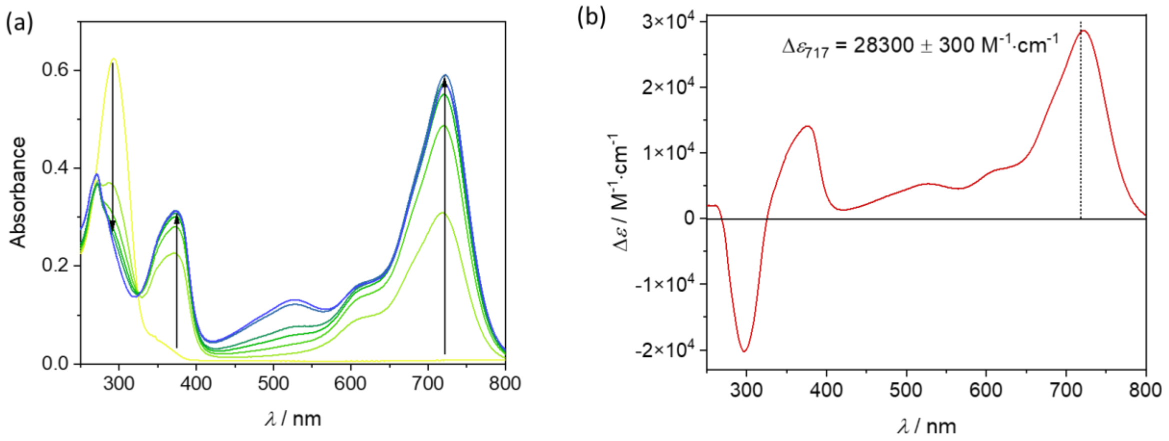

Supplementary Fig. 47*:* UV-vis absorption spectra of TAA-PEG$_7$ (20 µM) in aerated CH_3CN titrated by nitrosonium tetrafluoroborate ($NOBF_4$, concentration up to 150 µM). $NOBF_4$ has an oxidation potential of 1.25 V vs SCE in CH_3CN,[20] while the cyclic voltammogram of TAA-PEG$_7$ exhibits the first oxidation peak at 0.78 V vs SCE in CH_3CN (Supplementary Fig. 3). Thus, oxidation of TAA-PEG$_7$ to its radical cation (TAA-PEG$_7^{\bullet+}$) by $NOBF_4$ is thermodynamically feasible in CH_3CN. Changes in absorption spectra were as a function of increasing amounts of added oxidant, due to the formation of chemically oxidized TAA-PEG$_7^{\bullet+}$, which shows a characteristic absorption band centered at 717 nm analogously to TAA-OMe$^{\bullet+}$ and TAA-PEG$_3^{\bullet+}$. Formation of TAA-PEG$_7^{2+}$, which is supposed to feature an absorption band at 520 nm in CH_3CN analogously to TAA-OMe$^{\bullet+}$,[19] was not observed, suggesting that TAA-PEG$_7^{\bullet+}$ is the sole oxidation product under our conditions. Absorption spectra were recorded until no further changes were observed, i.e. until TAA-PEG$_7$ was fully oxidized to TAA-PEG$_7^{\bullet+}$. (b) Changes in molar extinction coefficients $\Delta\varepsilon$ of TAA-PEG$_7^{\bullet+}$, derived from the absorption changes from (a) based on the known initial concentration of TAA-PEG$_7$. The characteristic absorption band with maximum at 717 nm was observed with $\Delta\varepsilon$ of 28300 ± 300 M^{-1} cm^{-1}, and the absorption features match the SEC absorption spectrum of the TAA-PEG$_7^{\bullet+}$ in Supplementary Fig. 8.

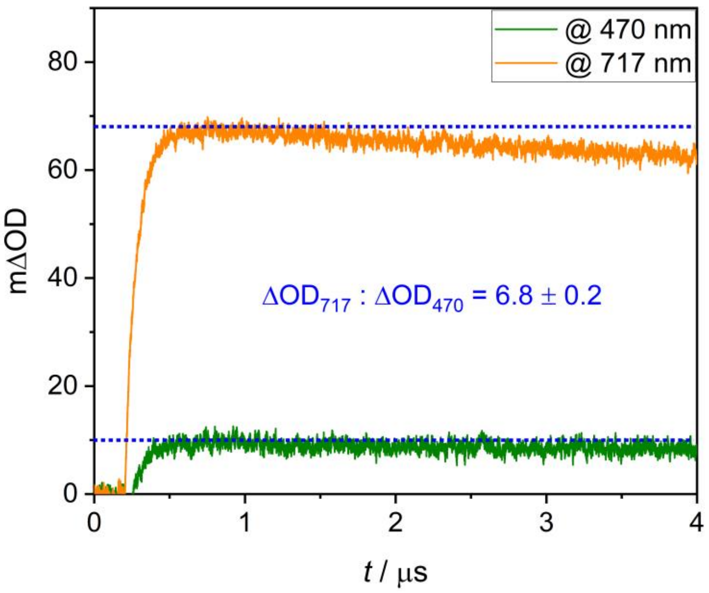

Supplementary Fig. 48: Transient absorption decay kinetics measured at 717 nm (orange line) and at 470 nm (green line) of $[Ru(bpz)_3]^{2+}$ (13 μM) in the presence of TAA-PEG_7 (2 mM) in aerated CH_3CN under pulsed laser excitation at 459 nm (pulse energy ~ 11 mJ).

The $\Delta\varepsilon$ value of TAA-$PEG_7^{\bullet+}$ at 717 nm was determined using two independent methods analogously as described above for $[Ru(bpz)_3]^+$ and TAA-$PEG_3^{\bullet+}$: I) quantitative chemical oxidation of TAA-PEG_7 to TAA-$PEG_7^{\bullet+}$ with $NOBF_4$ yields $\Delta\varepsilon_{717}$ of 28300 ± 300 M^{-1} cm^{-1} (Supplementary Fig. 47); II) TAA-$PEG_7^{\bullet+}$ is optically transparent at 470 nm (Supplementary Fig. 47b, Supplementary Fig. 8), whereas $[Ru(bpz)_3]^+$ has negligible absorption at 717 nm (Supplementary Fig. 41b). Following selective excitation of $[Ru(bpz)_3]^{2+}$ (13 μM) at 454 nm in aerated CH_3CN containing TAA-PEG_7 (2 mM), the TAA-$PEG_7^{\bullet+}$ and $[Ru(bpz)_3]^+$ photoproducts are formed in 1:1 ratio and can be monitored independently at 717 and 470 nm. The obtained ratio of ΔOD values at 717 nm and 470 nm reflects the ratio of their respective extinction coefficients (Supplementary Fig. 48) at the respective wavelengths. Using the measured change of extinction coefficient associated with the reduction of $[Ru(bpz)_3]^{2+}$ to $[Ru(bpz)_3]^+$ at 470 nm in CH_3CN ($\Delta\varepsilon_{470}$ = 4300 M^{-1} cm^{-1}) (Supplementary Fig. 41b), $\Delta\varepsilon_{717}$ of TAA-PEG_7 is determined to be 29200 ± 900 M^{-1} cm^{-1}, in line with the $\Delta\varepsilon_{717}$ value obtained via oxidation with $NOBF_4$ (28300 ± 300 M^{-1} cm^{-1}). The agreement of $\Delta\varepsilon_{717}$ values obtained by the two independent methods validates again the relative method (method II) for determination of $\Delta\varepsilon$ values for the radical species. This analysis furthermore corroborates the $\Delta\varepsilon_{470}$ value obtained for $[Ru(bpz)_3]^+$.

6.6 Δε of TAA-Cl$^{\bullet +}$

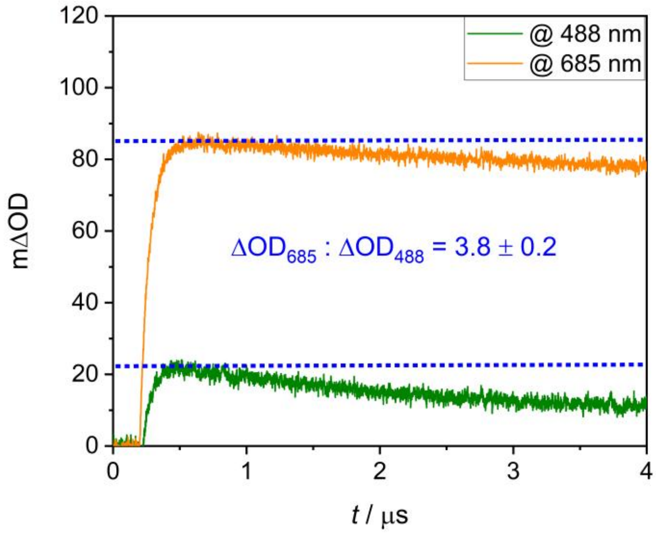

Supplementary Fig. 49: Transient absorption decay kinetics measured at 685 nm (orange line) and at 488 nm (green line) of $[Ru(bpz)_3]^{2+}$ (13 µM) in the presence of TAA-Cl (2 mM) in aerated CH_3CN under pulsed laser excitation at 450 nm (pulse energy ~ 13 mJ).

The Δε values for TAA-Cl$^{\bullet +}$ were determined with the relative method as validated above. TAA-Cl$^{\bullet +}$ has rare absorption at 488 nm according to its UV-vis SEC absorption spectrum (Supplementary Fig. 9), whereas $[Ru(bpz)_3]^{+}$ has negligible absorption at 685 nm (Supplementary Fig. 41b). Following selective excitation of $[Ru(bpz)_3]^{2+}$ (13 µM) at 450 nm in aerated CH_3CN containing TAA-Cl (2 mM), the TAA-Cl$^{\bullet +}$ and $[Ru(bpz)_3]^{+}$ photoproducts are formed in 1:1 ratio and can be monitored independently at 685 and 488 nm. The obtained ratio of ΔOD values at 685 nm and 488 nm reflects the ratio of their respective extinction coefficients (Supplementary Fig. 49) at the respective wavelengths. Using the measured change of extinction coefficient associated with the reduction of $[Ru(bpz)_3]^{2+}$ to $[Ru(bpz)_3]^{+}$ at 488 nm in CH_3CN ($\Delta\varepsilon_{488}$ = 6160 M^{-1} cm^{-1}) (Supplementary Fig. 41b), $\Delta\varepsilon_{685}$ of TAA-Cl is determined to be 23400 ± 1200 M^{-1} cm^{-1}, similar to the literature value (27000 M^{-1} cm^{-1}).[21]

6.7 $\Delta\varepsilon$ of TAA-Br$^{\bullet+}$

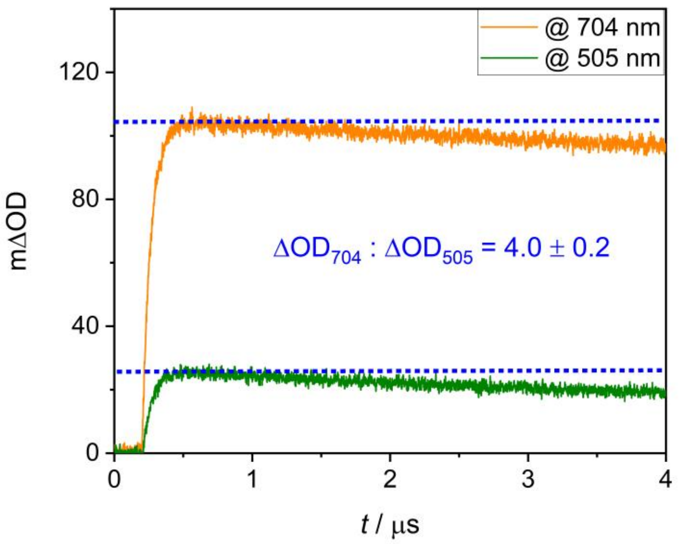

Supplementary Fig. 50: Transient absorption decay kinetics measured at 704 nm (orange line) and at 505 nm (green line) of $[Ru(bpz)_3]^{2+}$ (13 µM) in the presence of TAA-Br (2 mM) in aerated CH_3CN under pulsed laser excitation at 449 nm (pulse energy ~ 13 mJ).

The $\Delta\varepsilon$ values for TAA-Br$^{\bullet+}$ were determined with the relative method as validated above. TAA-Br$^{\bullet+}$ has an absorption band maximized at 704 nm and rare absorption in the range of 430 – 510 nm according to its UV-vis SEC absorption spectrum (Supplementary Fig. 10), whereas $[Ru(bpz)_3]^{+}$ has negligible absorption at 704 nm (Supplementary Fig. 41b). Following selective excitation of $[Ru(bpz)_3]^{2+}$ (13 µM) at 449 nm in aerated CH_3CN containing TAA-Br (2 mM), the TAA-Br$^{\bullet+}$ and $[Ru(bpz)_3]^{+}$ photoproducts are formed in 1:1 ratio and can be monitored independently at 704 and 505 nm. The obtained ratio of ΔOD values at 704 nm and 505 nm reflects the ratio of their respective extinction coefficients (Supplementary Fig. 50) at the respective wavelengths. Using the measured change of extinction coefficient associated with the reduction of $[Ru(bpz)_3]^{2+}$ to $[Ru(bpz)_3]^{+}$ at 505 nm in CH_3CN ($\Delta\varepsilon_{505}$ = 5300 M^{-1} cm^{-1}, Supplementary Fig. 41b), $\Delta\varepsilon_{704}$ of TAA-Br is determined to be 21200 ± 1100 M^{-1} cm^{-1}, which is found in between the literature values (17000 – 26000 M^{-1} cm^{-1}).[21,22]

6.8 $\Delta\varepsilon$ of TAA-I$^{•+}$

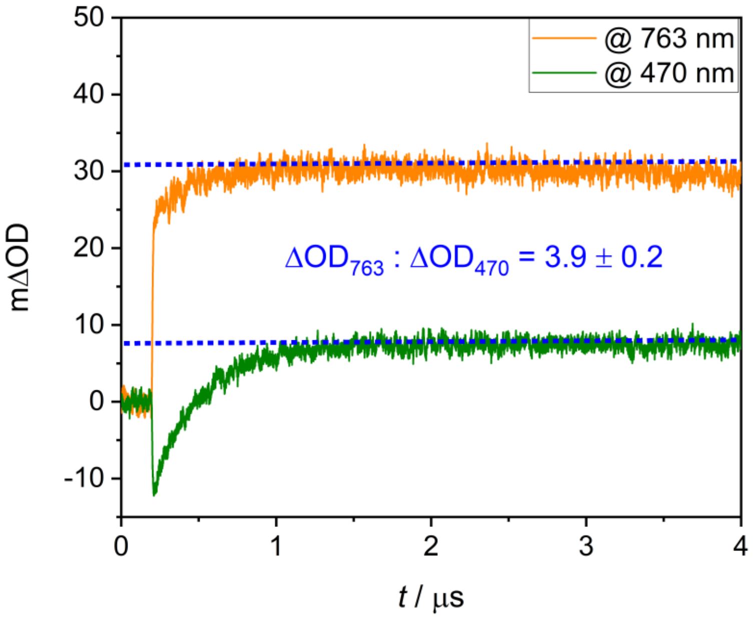

Supplementary Fig. 51: Transient absorption decay kinetics measured at 763 nm (orange line) and at 470 nm (green line) of $[Ru(bpz)_3]^{2+}$ (13 µM) in the presence of TAA-I (200 µM) in aerated CH_3CN under pulsed laser excitation at 452 nm (pulse energy ~ 13 mJ).

The $\Delta\varepsilon$ value for TAA-I$^{•+}$ were determined with the relative method as validated above. According to SEC absorption spectrum of TAA-I, its radical cation has an absorption band with a maximum at 763 nm and minor absorption in the range of 450 – 550 nm (Supplementary Fig. 11). $[Ru(bpz)_3]^+$ is optically transparent above 700 nm (Supplementary Fig. 40b). Following selective excitation of $[Ru(bpz)_3]^{2+}$ (13 µM) at 452 nm in aerated CH_3CN containing TAA-I (200 µM), the TAA-I$^{•+}$ and $[Ru(bpz)_3]^+$ photoproducts are formed in 1:1 ratio and can be monitored independently at 763 and 470 nm. The obtained ratio of ΔOD values at 763 nm and 470 nm reflects the ratio of their respective extinction coefficients (Supplementary Fig. 51) at the respective wavelengths. TAA-I has a relatively poor solubility in CH_3CN at room temperature. Given the resulting comparatively low concentration as a quencher (200 µM), photoinduced electron transfer from TAA-I to $[Ru(bpz)_3]^{2+}$ is slower than what was observed above with the other electron donors. This manifests in the observation of an initial negative signal in the green trace of Supplementary Fig. 50b, caused by the ground state bleach of photoexcited $[Ru(bpz)_3]^{2+}$ at the detection wavelength of 470 nm (Supplementary Fig. 15). After complete recovery of the ground state bleach, the $[Ru(bpz)_3]^+$ photoproduct shows a long-lived positive ΔOD signal, due to its characteristic absorption band centered at 488 nm (Supplementary Fig. 40b). Using the measured change in molar extinction coefficient of $[Ru(bpz)_3]^+$ at 470 nm in CH_3CN (~ 4300 M^{-1} cm^{-1}, Supplementary Fig. 40b), $\Delta\varepsilon_{763}$ of TAA-I$^{•+}$ is determined to be 16800 ± 900 M^{-1} cm^{-1}.

6.9 $\Delta\varepsilon$ of DMA$^{•+}$

According to the SEC absorption of DMA (Supplementary Fig. 12), its radical cation DMA$^{•+}$ has a characteristic absorption band with a maximum at 470 nm, in agreement with the literature.[23,24] The $\Delta\varepsilon_{470}$ value of 4500 ± 400 M^{-1} cm^{-1} has been previously determined.[25,26]

6.10 Δε of DMT$^{\bullet+}$

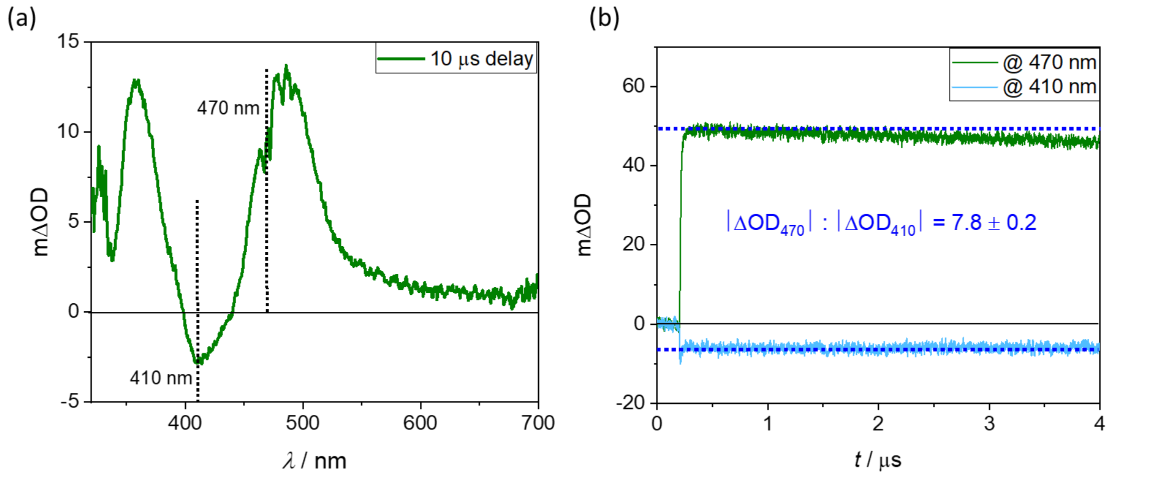

Supplementary Fig. 52: (a) Transient absorption spectrum (10 µs delay) and (b) transient absorption kinetics at 470 nm (green trace) and at 410 nm (bright blue trace) of $[Ru(bpz)_3]^{2+}$(13 µM) in the presence of DMT (10 mM) in aerated CH_3CN under pulsed laser excitation at 450 nm (pulse energy ~13 mJ). The spectrum in (a) was recorded with an integration time of 200 ns and a delay time of 10 µs after pulsed excitation, as indicated in the inset. Here, a time delay was applied to better visualize the fact that DMT$^{\cdot+}$ and $[Ru(bpz)_3]^+$ both absorb at 470 nm, whereas the bleach around 410 nm is relatively cleanly attributable to the formation of $[Ru(bpz)_3]^+$ from $[Ru(bpz)_3]^{2+}$ (Supplementary Fig. 41b).

Spectro-electrochemical experiments to monitor the radical cation of DMT in CH_3CN could not be accomplished successfully, presumably due to the formation of a dimer in the course of electrochemical oxidation, as reported in the literature.[16,27] According to previous studies, the radical cation of DMT shows an absorption band analogously to the DMA radical cation, with an absorption band maximum at ca. 470 nm and negligible absorption at 410 nm.[16] By comparison, $[Ru(bpz)_3]^+$ has a $\Delta\varepsilon_{470}$ value of 4300 M^{-1} cm^{-1} and for the bleach observable at ca. 410 nm, $\Delta\varepsilon_{410}$ value of -1700 M^{-1} cm^{-1} is determined for the formation of $[Ru(bpz)_3]^+$ from $[Ru(bpz)_3]^{2+}$ (Supplementary Fig. 41b). Consequently, in the transient absorption spectrum recorded with 10 µs delay after excitation of $[Ru(bpz)_3]^{2+}$ in the presence of excess DMT, $[Ru(bpz)_3]^+$ can be monitored cleanly at 410 nm (without contribution from DMT$^{\bullet+}$), whereas both of these two photoproducts contribute to the absorption transient signal at 470 nm. The change in extinction at that wavelength ($\Delta\varepsilon_{470,sum}$) is caused by the sum of both radical species (Supplementary Fig. 52a). The ΔOD ratio determined at 470 nm and 410 nm (7.8 ± 0.2) in the mixture indicates the ratio of $\Delta\varepsilon_{470,sum}$ (from both radical species) to $\Delta\varepsilon_{410}$ of $[Ru(bpz)_3]^+$ (Supplementary Fig. 52b). Based on the measured Δε values of $[Ru(bpz)_3]^+$ at 410 nm and 470 nm, the $\Delta\varepsilon_{470}$ value of DMT$^{\bullet+}$ was determined to be 9000 ± 300 M^{-1} cm^{-1}.

6.11 Δε of DMA-OMe$^{•+}$

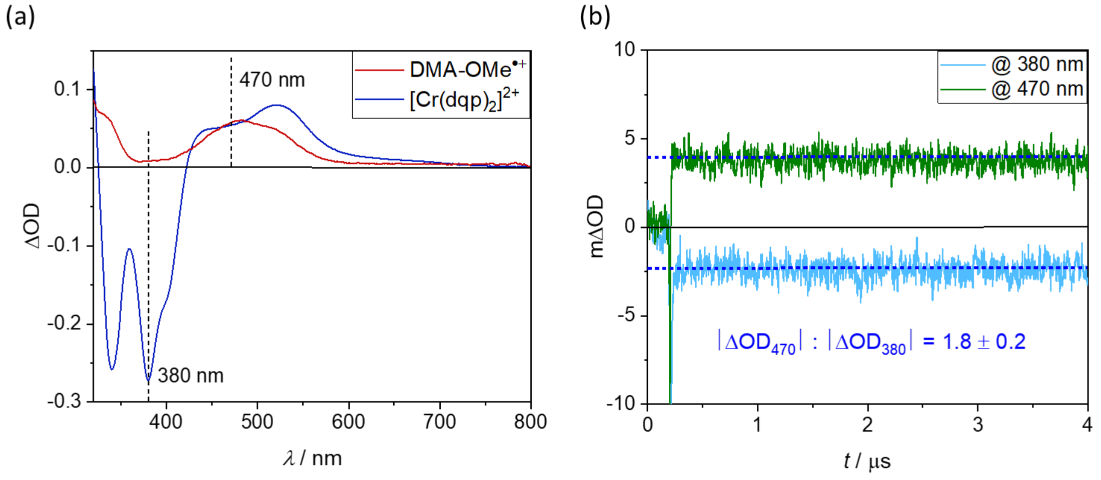

Supplementary Fig. 53: (a) Absorbance changes associated with the photochemical formation of $[Cr(dqp)_2]^{2+}$ from $[Cr(dqp)_2]^{3+}$ (dark blue solid line, from Supplementary Fig. 43) and the spectro-electrochemical absorption difference spectrum of DMA-OMe$^{•+}$ (red solid line) obtained following spectro-electrochemical oxidation of DMA-OMe (Supplementary Fig. 13). (b) Transient absorption decay kinetics measured at 380 nm (bright blue trace) and at 470 nm (green trace) of $[Cr(dqp)_2]^{3+}$(30 µM) in the presence of DMA-OMe (10 mM) in aerated CH_3CN under pulsed laser excitation at 419 nm (pulse energy of ~ 7 mJ).

According to the spectro-electrochemical data in Supplementary Fig. 13, the radical cation of DMA-OMe has a characteristic absorption band with a maximum at 480 nm and negligible absorption at 380 nm. Upon formation of $[Cr(dqp)_2]^{2+}$ from $[Cr(dqp)_2]^{3+}$, a strong bleach becomes observable at 380 nm with a $\Delta\varepsilon_{380}$ value of -10200 M^{-1} cm^{-1} (Supplementary Fig. 43b). Additional extinction at 470 nm with a $\Delta\varepsilon_{470}$ value of 2050 M^{-1} cm^{-1} is observed upon photochemical reduction of $[Cr(dqp)_2]^{3+}$ by TEA (Supplementary Fig. 43). Upon selective excitation of $[Cr(dqp)_2]^{3+}$ at 419 nm in the presence of excess DMA-OMe in aerated CH_3CN, a 1:1 mixture of $[Cr(dqp)_2]^{2+}$ and DMA-OMe$^{•+}$ is formed by photoinduced electron transfer. Given the spectral characteristics of these two photoproduct species, the observable change in optical density at 380 nm is caused by $[Cr(dqp)_2]^{2+}$ alone, whereas the observable ΔOD value at 470 nm is caused by contributions from both $[Cr(dqp)_2]^{2+}$ and DMA-OMe$^{•+}$ (Supplementary Fig. 53a). The experimentally determined ratio of absolute ΔOD values at 380 nm and 470 nm is 1.8 ± 0.2. Using the abovementioned known $\Delta\varepsilon_{380}$ value for $[Cr(dqp)_2]^{2+}$, the $\Delta\varepsilon_{470}$ value associated with the formation of $[Cr(dqp)_2]^{2+}$, and the $\Delta\varepsilon_{470,\ sum}$ value caused by both $[Cr(dqp)_2]^{2+}$ and DMA-OMe$^{•+}$ in the data of Supplementary Fig. 53b, a $\Delta\varepsilon_{470}$ value of 16300 ± 2000 M^{-1} cm^{-1} is obtained for DMA-OMe$^{•+}$.

7. Determination of cage escape quantum yields Φ_{CE}

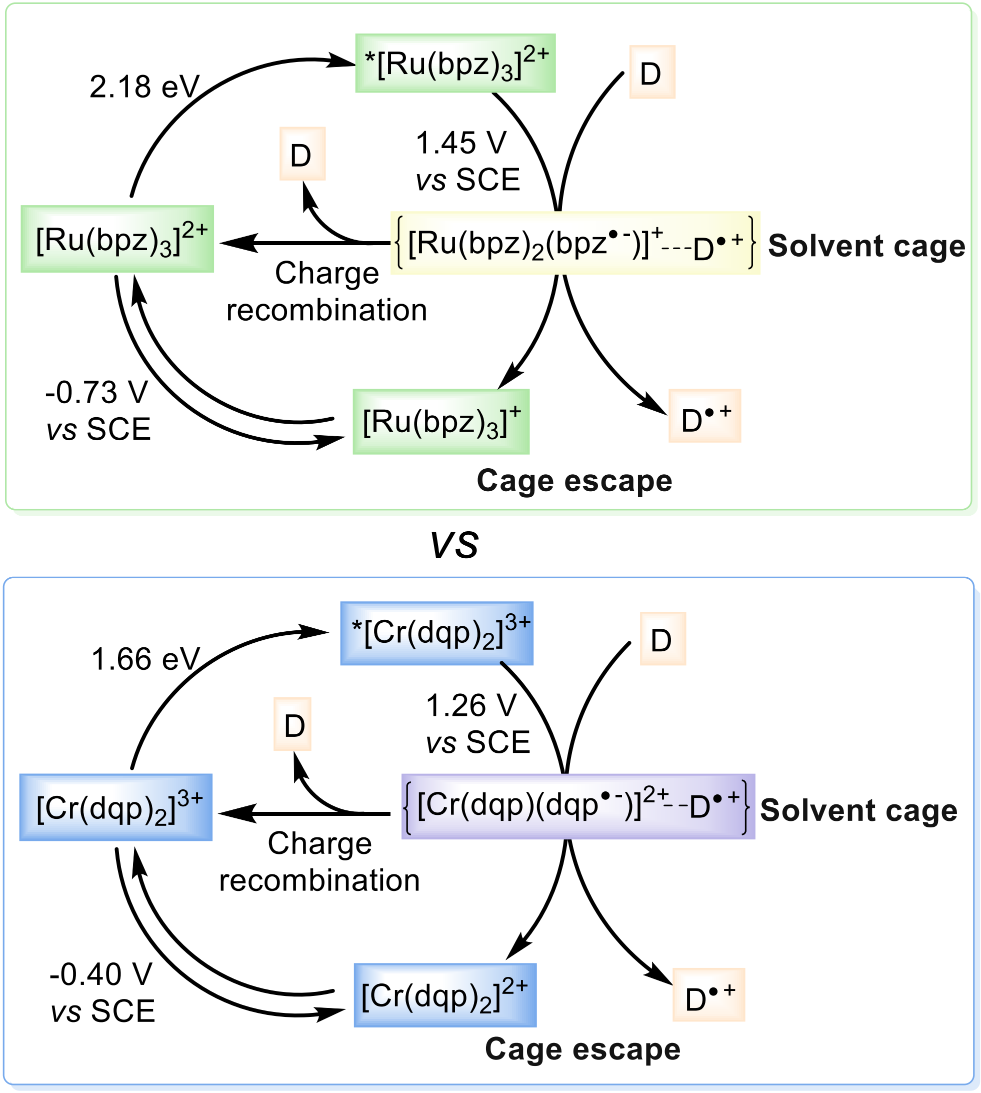

Supplementary Fig. 54: Schematic illustration of the photoredox catalytic cycles of $[Ru(bpz)_3]^{2+}$ (upper half) and $[Cr(dqp)_2]^{3+}$ (lower half) in the presence of a sacrificial electron donor (D). Photoexcitation promotes the complex to its photoactive excited state. In the presence of electron donor, a so-called encounter complex forms between the metal complex and the electron donor in a solvent cage. Within this solvent cage, photoinduced electron transfer leads to the caged photoproducts comprised of reduced metal complex and oxidized electron donor, a spin-correlated radical pair. Escape of these photoproducts from the solvent cage is then in competition with unproductive reverse electron transfer (charge recombination).

The cage escape quantum yields Φ_{CE} for the electron transfer photoproducts formed after excitation of $[Ru(bpz)_3]^{2+}$ or $[Cr(dqp)_2]^{3+}$ in the presence of various electron donors were determined with relative actinometry based on laser flash photolysis.[6,28,29] **Eq. 1** captures all relevant factors in compact form:

$$\Phi_{CE} = \frac{A_R}{A_S} \times \frac{\Delta OD_S}{\Delta OD_R} \times \frac{\Delta\varepsilon_R}{\Delta\varepsilon_S} \times \Phi_R \qquad \textbf{eq. 1}$$

Relative actinometry is based on comparative measurements of a sample (designated by the subscript "S" in the individual parameters included in **eq. 1**) and a reference solution (designated by the subscript "R" in **eq. 1**) with well-known properties, most notably the quantum yield Φ_R for the photochemical

formation of a given, easily detectable reference species.[28,29] In **eq. 1**, A_R and A_S stand for the absorbance of the reference and the sample at the (common) excitation wavelength, respectively. ΔOD_S and ΔOD_R represent the measured changes of optical densities of the sample and the reference solutions, respectively. $\Delta\varepsilon_S$ and $\Delta\varepsilon_R$ are the changes in extinction coefficients associated with photoproduct formation in the sample and the reference solution at the pertinent observation wavelength.

Throughout this work, a freshly prepared aqueous aerated solution of $[Ru(bpy)_3]Cl_2$ was used as the reference sample. Following visible-light irradiation, $[Ru(bpy)_3]^{2+}$ undergoes ultrafast and quantitative intersystem crossing and relaxation to a long-lived ^{3}MLCT excited state, which is readily detectable by its diagnostic ^{1}MLCT ground state absorption bleach centered around 455 nm. The change in molar extinction coefficient associated with the formation of ^{3}MLCT-excited $[Ru(bpy)_3]^{2+}$ at this wavelength is well-known and amounts to $\Delta\varepsilon_{455}$ = -10100 M^{-1} cm^{-1}.[30] Given the fact that intersystem crossing is quantitative, $\Phi_R = 1$ in this case. Based on prior studies of $[Cr(dqp)_2]^{3+}$ and closely related Cr^{III} complexes, intersystem crossing from the initially excited quartet states into the photoactive ^{2}E excited state is ultrafast.[31-34] The intersystem crossing is therefore commonly considered as quantitative in Cr^{III} polypyridine complexes.[35-37] Transient absorption measurements of the samples and the reference at their characteristic detection wavelengths give their ΔOD_S and ΔOD_R values. The absorbance values A_R and A_S at the excitation wavelength are accessible by simple UV-vis spectroscopy. The $\Delta\varepsilon_R$ value included in **eq. 1** corresponds to the $\Delta\varepsilon_{455}$ value of -10100 M^{-1} cm^{-1} quoted above. The $\Delta\varepsilon_S$ value in **eq. 1** corresponds to the $\Delta\varepsilon$ value determined in section 6 for the individual photoproducts. Thus, all parameters on the right-hand side of **eq. 1** are directly accessible, thereby allowing the determination of the cage escape quantum yields (Φ_{CE}) for electron transfer photoproducts.

In most cases, the reference and the sample concentrations were adjusted such as to result in the same absorbance at the excitation wavelength (A_R and A_S). In cases, in which an electron donor has non-negligible absorbance at the excitation wavelength, for example TAA-PEG_7 and THIQ, the absorbance of the electron donor was taken into account, and the used A_S value in those cases corresponds to the absorbance caused by the photosensitizer alone. The ΔOD_S and ΔOD_R values were obtained by measuring the decay kinetics of the sample and the reference under the strictly controlled identical conditions, with the same excitation energy. Most transient absorption kinetic traces exhibited a rise-and-decay behavior, in which the initial rise (typically at $t < 500$ ns) reflects the kinetics for photoinduced electron transfer from a given donor to the excited acceptor, whereas the subsequent decay reflects thermal reverse electron transfer to reinstate the starting materials.[38] The ΔOD_S and ΔOD_R values used for **eq. 1** were determined at the maxima reached in the course of this rise-and-decay behavior, because this reflects the point at which the photoproduct concentrations are maximal. Depending on what donor-acceptor combination is considered, this maximum occurs at different points in time, because different reaction free energies for electron transfer cause different reaction kinetics.

All measurements were performed using freshly prepared samples, and each sample was measured only for a time window of up to 10 minutes, to minimize systematic errors caused by the formation of unwanted photo-degradation products.

7.1 TAA-OMe

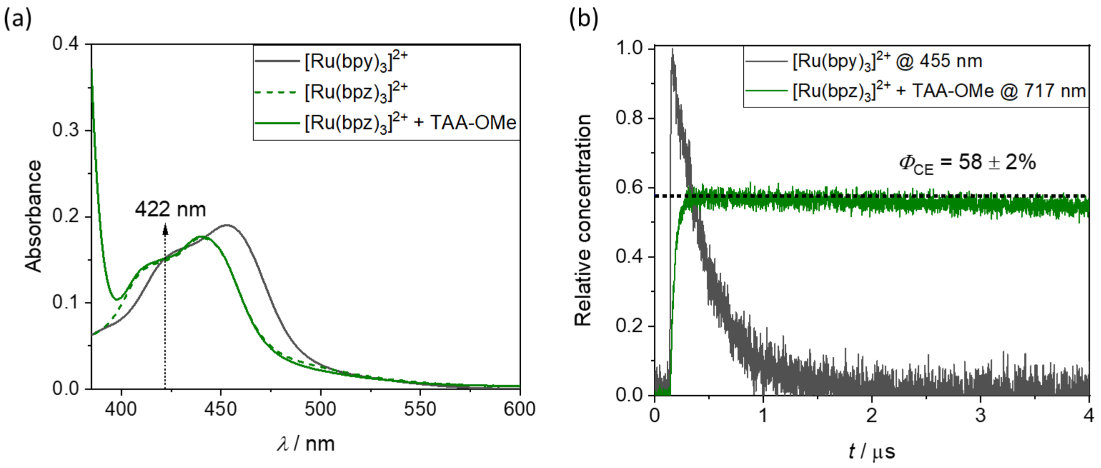

Supplementary Fig. 55: (a) Absorption spectra of the reference $[Ru(bpy)_3]^{2+}$ (12 µM in aerated H_2O, dark grey trace), neat $[Ru(bpz)_3]^{2+}$ (12 µM, green dotted trace), and the donor-acceptor pair $[Ru(bpz)_3]^{2+}$ (12 µM) / TAA-OMe (2 mM) in aerated CH_3CN at 293 K (green solid trace). All three solutions have identical absorbance at 422 nm. (b) Transient absorption decays of the reference $[Ru(bpy)_3]^{2+}$ at 455 nm (dark grey trace) and the $[Ru(bpz)_3]^{2+}$ / TAA-OMe donor-acceptor pair (green trace) from (a) at 717 nm. The relative concentrations of photoproducts (^{3}MLCT-excited $[Ru(bpy)_3]^{2+}$ at 455 nm and TAA-OMe$^{\bullet+}$ at 717 nm) were derived from the measured ΔOD_R and ΔOD_S values and their respective Δε values at the relevant observation wavelengths ($\Delta\varepsilon_{455} = \Delta\varepsilon_R$ = -10100 M^{-1} cm^{-1},[30] $\Delta\varepsilon_{717} = \Delta\varepsilon_S$ = 32600 ± 300 M^{-1} cm^{-1}). This analysis yields a Φ_{CE} value of 58 ± 2 % for the $[Ru(bpz)_3]^{2+}$ / TAA-OMe pair in CH_3CN. Based on the measured ΔOD_R and ΔOD_S values and their respective Δε values, the concentrations of ^{3}MLCT-excited $[Ru(bpy)_3]^{2+}$ and TAA-OMe$^{\bullet+}$ were determined to be 3.14 and 1.82 ± 0.06 µM, respectively. Excitation of both the reference and the sample occurred at 422 nm with a ns-pulsed laser (pulse energy ~ 9 mJ), $A_S = A_R$ in this case.

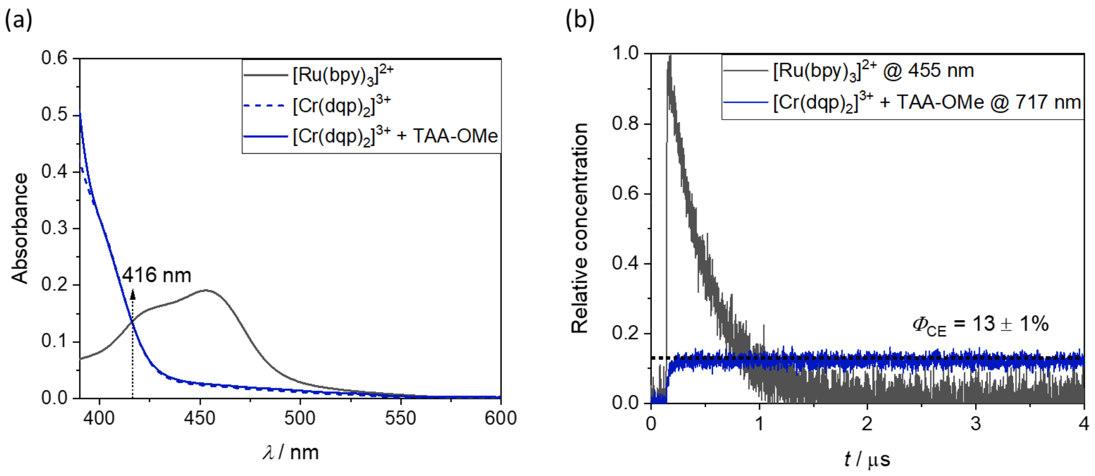

Supplementary Fig. 56: (a) Absorption spectra of the reference $[Ru(bpy)_3]^{2+}$ (12 µM in aerated H_2O, dark grey trace), neat $[Cr(dqp)_2]^{3+}$ (30 µM, dark blue dotted trace), and the donor-acceptor pair $[Cr(dqp)_2]^{3+}$ (30 µM) / TAA-OMe (2 mM) in aerated CH_3CN at 293 K (dark blue solid trace). All three solutions have identical absorbance at 416 nm. (b) Transient absorption decays of the reference $[Ru(bpy)_3]^{2+}$ at 455 nm (dark grey trace) and the $[Cr(dqp)_2]^{3+}$ / TAA-OMe donor-acceptor pair (dark blue trace) from (a) at 717 nm. The relative concentrations of photoproducts (^{3}MLCT-excited $[Ru(bpy)_3]^{2+}$ at 455 nm and TAA-OMe$^{\bullet+}$ at 717 nm) were derived from the measured ΔOD_R and ΔOD_S values and their respective Δε values at the relevant observation wavelengths ($\Delta\varepsilon_{455} = \Delta\varepsilon_R$ = -10100 M^{-1} cm^{-1},[30] $\Delta\varepsilon_{717} = \Delta\varepsilon_S$ = 32600 ± 300 M^{-1} cm^{-1}). This analysis yields a Φ_{CE} value of 13 ± 1 % for the $[Cr(dqp)_2]^{3+}$ / TAA-OMe pair in aerated CH_3CN. Based on the measured ΔOD_R and ΔOD_S values and their respective Δε values, the concentrations of ^{3}MLCT-excited $[Ru(bpy)_3]^{2+}$ and TAA-OMe$^{\bullet+}$ were determined to be 1.56 and 0.20 ± 0.02 µM, respectively. Excitation of both the reference and the sample occurred at 416 nm with a ns-pulsed laser (pulse energy ~ 8 mJ), $A_S = A_R$ in this case.

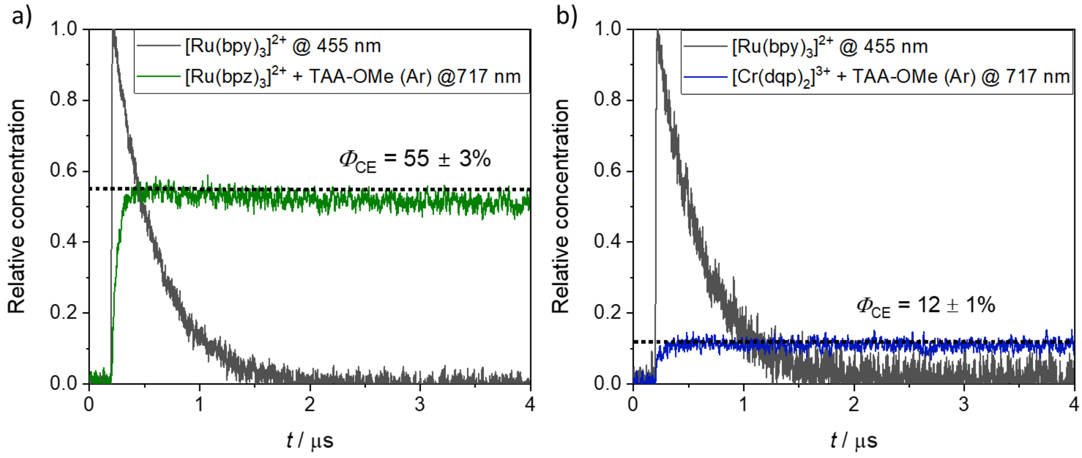

Supplementary Fig. 57: Transient absorption decays of the reference $[Ru(bpy)_3]^{2+}$ at 455 nm (dark grey trace) and a) the $[Ru(bpz)_3]^{2+}$ (12 µM) / TAA-OMe (2 mM) (green trace) and b) the $[Cr(dqp)_2]^{3+}$ (30 µM) / TAA-OMe (2 mM) (dark blue trace) donor-acceptor pairs in deaerated CH_3CN at 293 K at 717 nm. The relative concentrations of photoproducts (^{3}MLCT-excited $[Ru(bpy)_3]^{2+}$ at 455 nm and TAA-OMe$^{\bullet+}$ at 717 nm) were derived from the measured ΔOD_R and ΔOD_S values and their respective Δε values at the relevant observation wavelengths ($\Delta\varepsilon_{455} = \Delta\varepsilon_R$ = -10100 M^{-1} cm^{-1},[30] $\Delta\varepsilon_{717} = \Delta\varepsilon_S$ = 32600 ± 300 M^{-1} cm^{-1}). This analysis yields a Φ_{CE} value of 55 ± 3 % for the $[Ru(bpz)_3]^{2+}$ (12 µM) / TAA-OMe (2 mM) pair and 12 ± 1 % for the $[Cr(dqp)_2]^{3+}$ / TAA-OMe pair in deaerated CH_3CN, similar to the values (58 ± 2 % and 13 ± 1 %) obtained in aerated CH_3CN under the same conditions. Excitation of both the reference and the sample occurred at 416 nm with a ns-pulsed laser (pulse energy ~ 8 mJ), $A_S = A_R$ in this case.

In acetonitrile solutions of $[Ru(bpz)_3]^{2+}$ / TAA-OMe and $[Cr(dqp)_2]^{3+}$ / TAA-OMe donor-acceptor pairs, the presence of oxygen from air does not affect the cage escape quantum yield Φ_{CE}, as seen from comparison of the data presented in Supplementary Fig. 55b/56b (recorded from aerated solution) and in Supplementary Fig. 57, obtained from a deaerated sample.

7.2 TAA-PEG$_3$

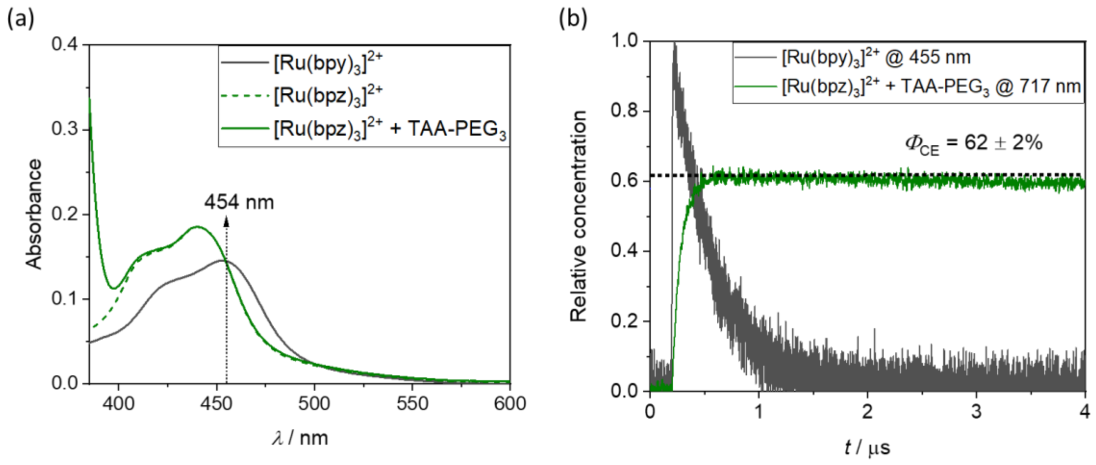

Supplementary Fig. 58: (a) Absorption spectra of the reference $[Ru(bpy)_3]^{2+}$ (12 µM in aerated H_2O, dark grey trace), neat $[Ru(bpz)_3]^{2+}$ (13 µM, green dotted trace), and the donor-acceptor pair $[Ru(bpz)_3]^{2+}$ (13 µM) / TAA-PEG$_3$ (2 mM) in aerated CH_3CN at 293 K (green solid trace). All three solutions have identical absorbance at 454 nm. (b) Transient absorption decays of the reference $[Ru(bpy)_3]^{2+}$ at 455 nm (dark grey trace) and the $[Ru(bpz)_3]^{2+}$ / TAA-PEG$_3$ donor-acceptor pair (green trace) from (a) at 717 nm. The relative concentrations of photoproducts (^{3}MLCT-excited $[Ru(bpy)_3]^{2+}$ at 455 nm and TAA- PEG$_3^{\bullet+}$ at 717 nm) were derived from the measured ΔOD_R and ΔOD_S values and their respective Δε values at the relevant observation

wavelengths ($\Delta\varepsilon_{455} = \Delta\varepsilon_R$ = -10100 M^{-1} cm^{-1},[30] $\Delta\varepsilon_{717} = \Delta\varepsilon_S$ = 31300 ± 300 M^{-1} cm^{-1}). This analysis yields a Φ_{CE} value of 62 ± 2 % for the $[Ru(bpz)_3]^{2+}$ / TAA-PEG$_3$ pair in aerated CH_3CN. Excitation of both the reference and the sample occurred at 454 nm with a ns-pulsed laser (pulse energy ~ 13 mJ), $A_S = A_R$ in this case.

In **Error! Reference source not found.**b, the increase in optical density at 717 nm (green trace) arising f rom the formation of the TAA-PEG$_3$ radical cation after pulsed laser excitation of $[Ru(bpz)_3]^{2+}$ is slower than with the TAA-OMe donor (Supplementary Fig. 55 b), indicating less rapid photoinduced electron transfer with TAA-PEG$_7$. This observation is in line with the rate constants determined for the quenching of ^{3}MLCT-excited $[Ru(bpz)_3]^{2+}$ by these two donors (10.27 × 10^9 $M^{-1}s^{-1}$ for TAA-OMe and 7.43 × 10^9 $M^{-1}s^{-1}$ for TAA-PEG$_3$, Supplementary Fig. 16b and Supplementary Fig. 17b). Similar phenomenon was also observed with the $[Cr(dqp)_2]^{3+}$ / TAA-PEG$_3$ donor-acceptor pair (Supplementary Fig. 59b, Supplementary Table 1).

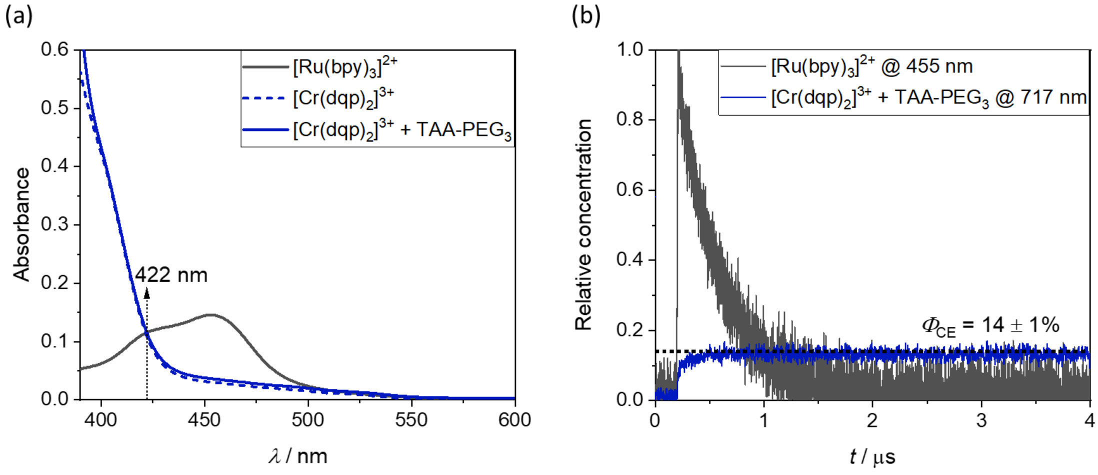

Supplementary Fig. 59: (a) Absorption spectra of the reference $[Ru(bpy)_3]^{2+}$ (12 µM in aerated H_2O, dark grey trace), neat $[Cr(dqp)_2]^{3+}$ (40 µM, dark blue dotted trace), and the donor-acceptor pair $[Cr(dqp)_2]^{3+}$ (40 µM) / TAA-PEG$_3$ (2 mM) in aerated CH_3CN at 293 K (dark blue solid trace). All three solutions have identical absorbance at 422nm. (b) Transient absorption decays of the reference $[Ru(bpy)_3]^{2+}$ at 455 nm (dark grey trace) and the $[Cr(dqp)_2]^{3+}$ / TAA-PEG$_3$ donor-acceptor pair (dark blue trace) from (a) at 717 nm. The relative concentrations of photoproducts (^{3}MLCT-excited $[Ru(bpy)_3]^{2+}$ at 455 nm and TAA-OMe$^{\bullet+}$ at 717 nm) were derived from the measured ΔOD_R and ΔOD_S values and their respective Δε values at the relevant observation wavelengths ($\Delta\varepsilon_{455} = \Delta\varepsilon_R$ = -10100 M^{-1} cm^{-1},[30] $\Delta\varepsilon_{717} = \Delta\varepsilon_S$ = 31300 ± 300 M^{-1} cm^{-1}). This analysis yields a Φ_{CE} value of 14 ± 1 % for the $[Cr(dqp)_2]^{3+}$ / TAA-PEG$_3$ pair in aerated CH_3CN. Excitation of both the reference and the sample occurred at 422 nm with a ns-pulsed laser (pulse energy ~ 9 mJ), $A_S = A_R$ in this case.

7.3 TAA-PEG$_7$

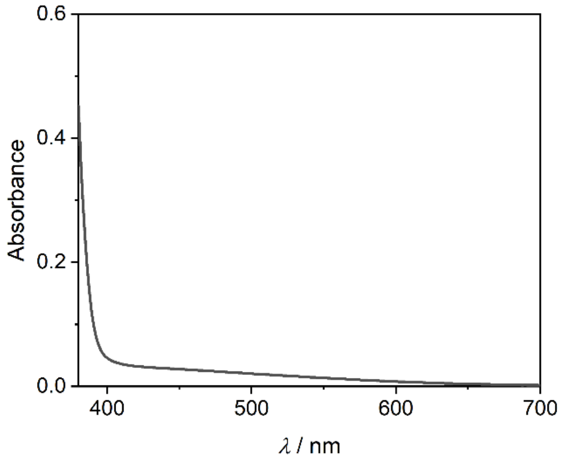

Supplementary Fig. 60: UV/vis absorption spectrum of TAA-PEG$_7$ (2 mM) in aerated CH_3CN at 293 K.

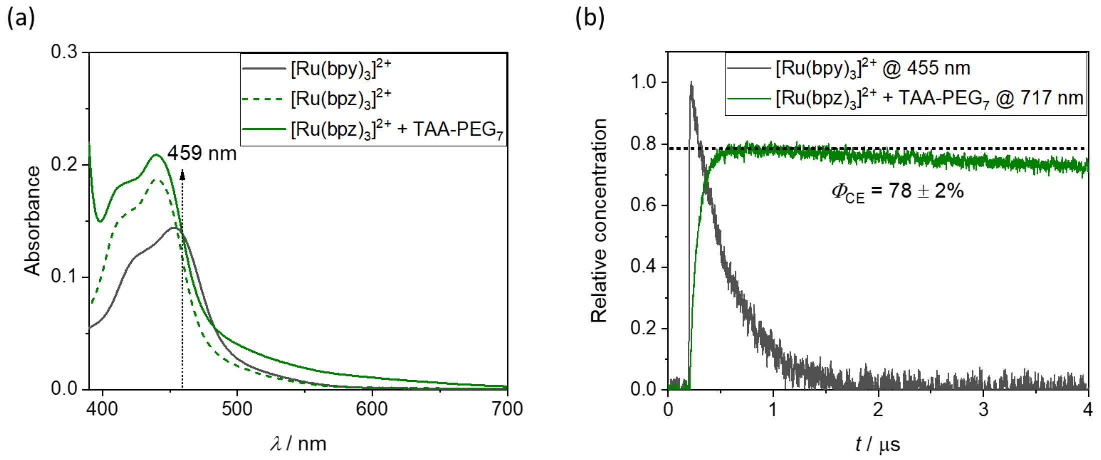

Supplementary Fig. 61: (a) Absorption spectra of the reference $[Ru(bpy)_3]^{2+}$ (12 µM in aerated H_2O, dark grey solid trace), neat $[Ru(bpz)_3]^{2+}$ (13 µM, green dotted trace), and the donor-acceptor pair $[Ru(bpz)_3]^{2+}$ (12 µM) / TAA-PEG$_7$ (2 mM) in aerated CH_3CN at 293 K (green solid trace). The differences in the absorption spectra of the neat $[Ru(bpz)_3]^{2+}$ solution and the $[Ru(bpz)_3]^{2+}$ sample containing 2 mM TAA-PEG$_7$ arise from the absorption of TAA-PEG$_7$ (Supplementary Fig. 60). Excitation of a neat solution of TAA-PEG$_7$ (2 mM) in CH_3CN at 450 nm does not yield any detectable emission, and we do not expect that direct absorbance of excitation light by TAA-PEG$_7$ will affect the photoinduced electron transfer studies performed here, other than diminishing the amount of light absorbed by $[Ru(bpy)_3]^{2+}$ by approximately 18% with respect to a $[Ru(bpy)_3]^{2+}$ solution of equal concentration without TAA-PEG$_7$. In this specific case here, the absorbance value of the neat $[Ru(bpz)_3]^{2+}$ solution at 459 nm (green dotted trace) was used as the *A*s value for determining the Φ_{CE} value with **eq. 1**. (b) Transient absorption decays of the reference $[Ru(bpy)_3]^{2+}$ at 455 nm (dark grey trace) and the $[Ru(bpz)_3]^{2+}$ / TAA-PEG$_7$ donor-acceptor pair (green trace) from (a) at 717 nm. The relative concentrations of photoproducts (^{3}MLCT-excited $[Ru(bpy)_3]^{2+}$ at 455 nm and TAA- PEG$_7^{\bullet+}$ at 717 nm) were derived from the measured ΔOD_R and ΔOD_S values and their respective Δε values at the relevant observation wavelengths ($\Delta\varepsilon_{455} = \Delta\varepsilon_R$ = -10100 M^{-1} cm^{-1},[30] $\Delta\varepsilon_{717} = \Delta\varepsilon_S$ = 28300 ± 300 M^{-1} cm^{-1}). This analysis yields a Φ_{CE} value of 78 ± 2 % for the $[Ru(bpz)_3]^{2+}$ / TAA-PEG$_7$ pair in aerated CH_3CN. Excitation of both the reference and the sample occurred at 459 nm with a ns-pulsed laser (pulse energy ~ 13 mJ).

Electron transfer between the TAA-PEG$_7$ and the $[Ru(bpz)_3]^{2+}$ is further slowed down relative to TAA-OMe and TAA-PEG$_3$, presumably due to the bulkier PEG$_7$ substituents. This is in line with the lower rate

constant of 6.79 × 10^9 $M^{-1}s^{-1}$ observed for quenching of the ^{3}MLCT excited state of $[Ru(bpz)_3]^{2+}$ by TAA-PEG_7 (Supplementary Fig. 18b) in comparison to TAA-OMe (10.27 × 10^9 $M^{-1}s^{-1}$) and TAA-PEG_3 (7.43 × 10^9 $M^{-1}s^{-1}$) as shown in Supplementary Fig. 16b and Supplementary Fig. 17b. Similar phenomenon was also observed with the $[Cr(dqp)_2]^{3+}$ / TAA-PEG_7 donor-acceptor pair (Supplementary Table 1).

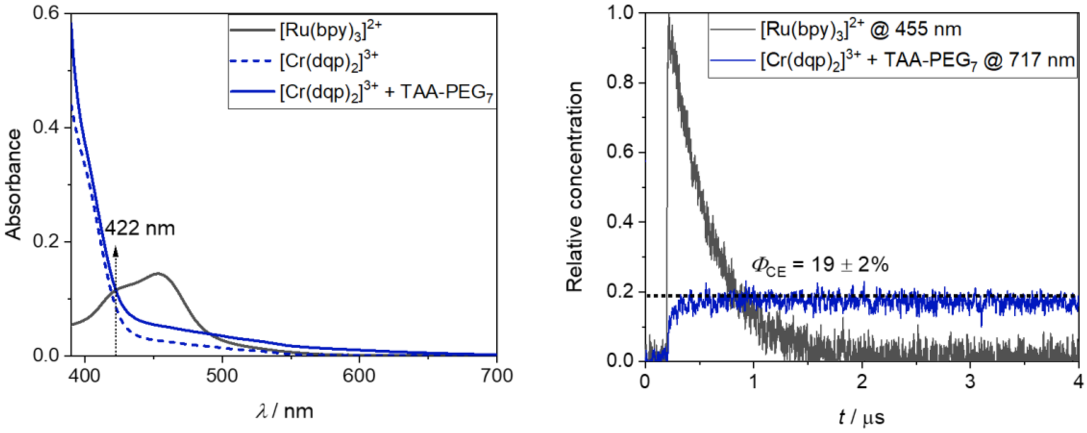

Supplementary Fig. 62*:* (a) Absorption spectra of the reference $[Ru(bpy)_3]^{2+}$ (12 µM in aerated H_2O, dark grey solid trace), neat $[Cr(dqp)_2]^{3+}$ (30 µM, dark blue dotted trace), and the donor-acceptor pair $[Cr(dqp)_2]^{3+}$ (30 µM) / TAA-PEG_7 (2 mM) in aerated CH_3CN at 293 K (dark blue solid trace). The differences in the absorption spectra of the neat $[Cr(dqp)_2]^{3+}$ solution and the $[Cr(dqp)_2]^{3+}$ sample containing 2 mM TAA-PEG_7 arise from the absorption of TAA-PEG_7 (Supplementary Fig. 60). Excitation of a neat solution of TAA-PEG_7 (2 mM) in CH_3CN at 420 nm does not yield any detectable emission, and we do not expect that direct absorbance of excitation light by TAA-PEG_7 will affect the photoinduced electron transfer studies performed here, other than diminishing the amount of light absorbed by $[Cr(dqp)_2]^{3+}$ by approximately 33% with respect to a $[Cr(dqp)_2]^{3+}$ solution of equal concentration without TAA-PEG_7. In this specific case here, the absorbance value of the neat $[Cr(dqp)_2]^{3+}$ solution at 422 nm (dark blue dotted trace) was used as the A_S value for determining the Φ_{CE} value with **eq. 1**. (b) Transient absorption decays of the reference $[Ru(bpy)_3]^{2+}$ at 455 nm (dark grey trace) and the $[Cr(dqp)_2]^{3+}$ / TAA-PEG_7 donor-acceptor pair (dark blue trace) from (a) at 717 nm. The relative concentrations of photoproducts (^{3}MLCT-excited $[Ru(bpy)_3]^{2+}$ at 455 nm and TAA- $PEG_7^{\bullet+}$ at 717 nm) were derived from the measured ΔOD_R and ΔOD_S values and their respective $\Delta\varepsilon$ values at the relevant observation wavelengths ($\Delta\varepsilon_{455} = \Delta\varepsilon_R$ = -10100 M^{-1} cm^{-1},[30] $\Delta\varepsilon_{717} = \Delta\varepsilon_S$ = 28300 ± 300 M^{-1} cm^{-1}). This analysis yields a Φ_{CE} value of 19 ± 2 % for the $[Cr(dqp)_2]^{3+}$ / TAA-PEG_7 pair in aerated CH_3CN. Excitation of both the reference and the sample occurred at 422 nm with a ns-pulsed laser (pulse energy ~ 13 mJ).

7.4 TAA-Cl

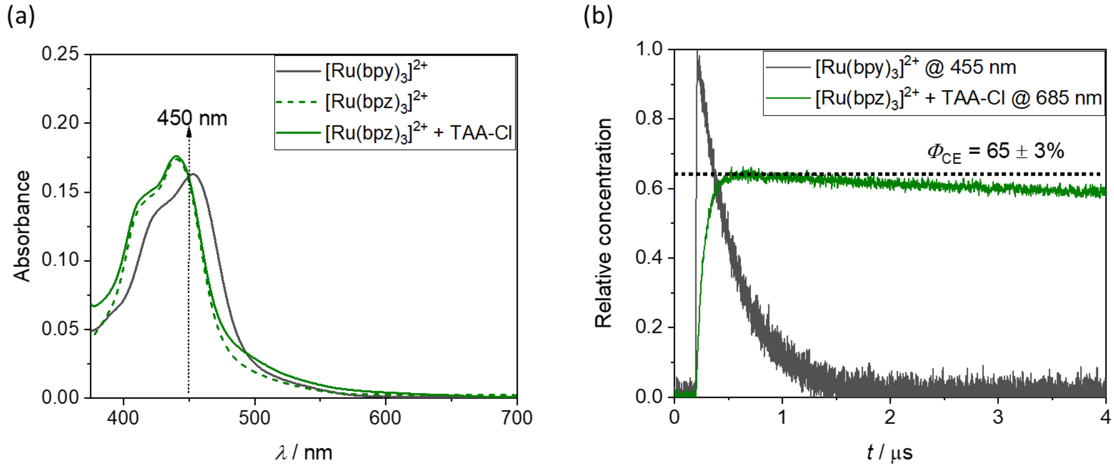

Supplementary Fig. 63: (a) Absorption spectra of the reference $[Ru(bpy)_3]^{2+}$ (14 µM in aerated H_2O, dark grey trace), neat $[Ru(bpz)_3]^{2+}$ (13 µM, green dotted trace), and the donor-acceptor pair $[Ru(bpz)_3]^{2+}$ (13 µM) / TAA-Cl (2 mM) in aerated CH_3CN at 293 K (green solid trace). All three solutions have identical absorbance at 450 nm. The absorption difference between 470 nm and 600 nm between the green traces is tentatively attributed to the formation of small amounts of $[Ru(bpy)_3]^+$ as a result of exposure to ambient light. (b) Transient absorption decays of the reference $[Ru(bpy)_3]^{2+}$ at 455 nm (dark grey trace) and the $[Ru(bpz)_3]^{2+}$ / TAA-Cl donor-acceptor pair (green trace) from (a) at 685 nm. The relative concentrations of photoproducts (^{3}MLCT-excited $[Ru(bpy)_3]^{2+}$ at 455 nm and TAA- $Cl^{\bullet+}$ at 685 nm) were derived from the measured ΔOD_R and ΔOD_S values and their respective Δε values at the relevant observation wavelengths ($\Delta\varepsilon_{455} = \Delta\varepsilon_R = -10100\ M^{-1}\ cm^{-1}$,[30] $\Delta\varepsilon_{685} = \Delta\varepsilon_S = 23400 \pm 1200\ M^{-1}\ cm^{-1}$). This analysis yields a Φ_{CE} value of 65 ± 3 % for the $[Ru(bpz)_3]^{2+}$ / TAA-Cl pair in aerated CH_3CN. Excitation of both the reference and the sample occurred at 450 nm with a ns-pulsed laser (pulse energy ~ 13 mJ), $A_S = A_R$ in this case.

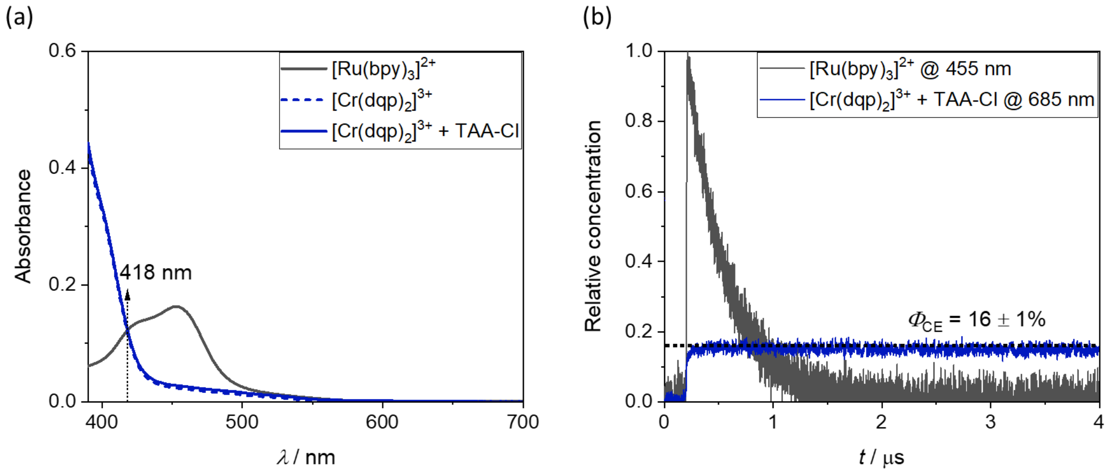

Supplementary Fig. 64: (a) Absorption spectra of the reference $[Ru(bpy)_3]^{2+}$ (14 µM in aerated H_2O, dark grey trace), neat $[Cr(dqp)_2]^{3+}$ (30 µM, dark blue dotted trace), and the donor-acceptor pair $[Cr(dqp)_2]^{3+}$ (30 µM) / TAA-Cl (2 mM) in aerated CH_3CN at 293 K (dark blue solid trace). All three solutions have identical absorbance at 418 nm. (b) Transient absorption decays of the reference $[Ru(bpy)_3]^{2+}$ at 455 nm (dark grey trace) and the $[Cr(dqp)_2]^{3+}$ / TAA-Cl donor-acceptor pair (dark blue trace) from (a) at 685 nm. The relative concentrations of photoproducts (^{3}MLCT-excited $[Ru(bpy)_3]^{2+}$ at 455 nm and TAA-$Cl^{\bullet+}$ at 685 nm) were derived from the measured ΔOD_R and ΔOD_S values and their respective Δε values at the relevant observation wavelengths ($\Delta\varepsilon_{455} = \Delta\varepsilon_R = -10100\ M^{-1}\ cm^{-1}$,[30] $\Delta\varepsilon_{685} = \Delta\varepsilon_S = 23400 \pm 1200\ M^{-1}\ cm^{-1}$). This analysis yields a Φ_{CE} value of 16 ± 1 % for the $[Cr(dqp)_2]^{3+}$ / TAA-Cl pair in aerated CH_3CN. Excitation of both the reference and the sample occurred at 418 nm with a ns-pulsed laser (pulse energy ~ 9 mJ), $A_S = A_R$ in this case.

7.5 TAA-Br

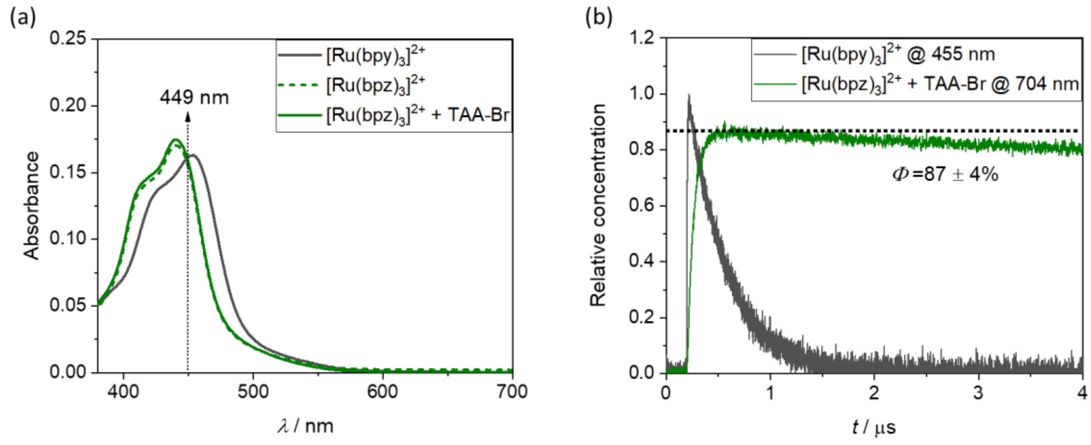

Supplementary Fig. 65: (a) Absorption spectra of the reference $[Ru(bpy)_3]^{2+}$ (14 µM in aerated H_2O, dark grey trace), neat $[Ru(bpz)_3]^{2+}$ (13 µM, green dotted trace), and the donor-acceptor pair $[Ru(bpz)_3]^{2+}$ (13 µM) / TAA-Br (2 mM) in aerated CH_3CN at 293 K (green solid trace). All three solutions have identical absorbance at 449 nm. (b) Transient absorption decays of the reference $[Ru(bpy)_3]^{2+}$ at 455 nm (dark grey trace) and the $[Ru(bpz)_3]^{2+}$ / TAA-Br donor-acceptor pair (green trace) from (a) at 704 nm. The relative concentrations of photoproducts (^{3}MLCT-excited $[Ru(bpy)_3]^{2+}$ at 455 nm and TAA-Br$^{•+}$ at 704 nm) were derived from the measured ΔOD_R and ΔOD_S values and their respective Δε values at the relevant observation wavelengths ($\Delta\varepsilon_{455} = \Delta\varepsilon_R$ = -10100 M^{-1} cm^{-1},[30] $\Delta\varepsilon_{704} = \Delta\varepsilon_S$ = 21200 ± 1100 M^{-1} cm^{-1}). This analysis yields a Φ_{CE} value of 87 ± 4 % for the $[Ru(bpz)_3]^{2+}$ / TAA-Br pair in aerated CH_3CN. Excitation of both the reference and the sample occurred at 449 nm with a ns-pulsed laser (pulse energy ~ 13 mJ), $A_S = A_R$ in this case.

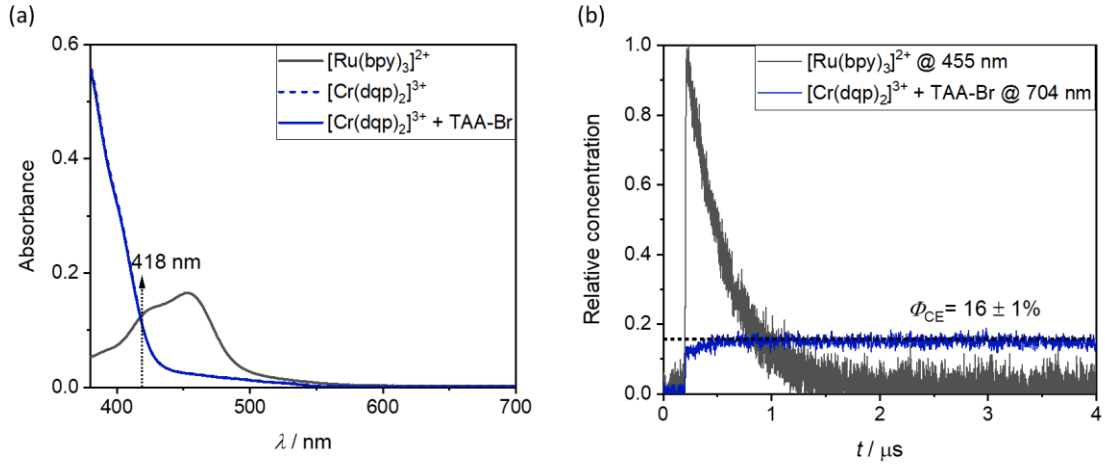

Supplementary Fig. 66: (a) Absorption spectra of the reference $[Ru(bpy)_3]^{2+}$ (14 µM in aerated H_2O, dark grey trace), neat $[Cr(dqp)_2]^{3+}$ (30 µM, dark blue dotted trace), and the donor-acceptor pair $[Cr(dqp)_2]^{3+}$ (30 µM) / TAA-Br (2 mM) in aerated CH_3CN at 293 K (dark blue solid trace). All three solutions have identical absorbance at 418 nm. (b) Transient absorption decays of the reference $[Ru(bpy)_3]^{2+}$ at 455 nm (dark grey trace) and the $[Cr(dqp)_2]^{3+}$ / TAA-Br donor-acceptor pair (dark blue trace) from (a) at 704 nm. The relative concentrations of photoproducts (^{3}MLCT-excited $[Ru(bpy)_3]^{2+}$ at 455 nm and TAA-Br$^{•+}$ at 704 nm) were derived from the measured ΔOD_R and ΔOD_S values and their respective Δε values at the relevant observation wavelengths ($\Delta\varepsilon_{455} = \Delta\varepsilon_R$ = -10100 M^{-1} cm^{-1},[30] $\Delta\varepsilon_{704} = \Delta\varepsilon_S$ = 21200 ± 1100 M^{-1} cm^{-1}). This analysis yields a Φ_{CE} value of 16 ± 1 % for the $[Cr(dqp)_2]^{3+}$ / TAA-Br pair in aerated CH_3CN. Excitation of both the reference and the sample occurred at 418 nm with a ns-pulsed laser (pulse energy ~ 9 mJ), $A_S = A_R$ in this case.

7.6 TAA-I

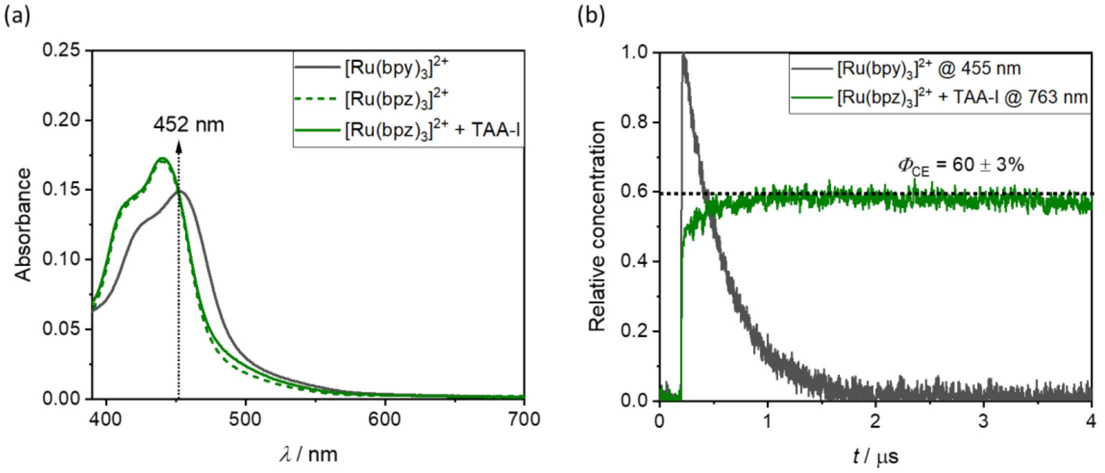

Supplementary Fig. 67: (a) Absorption spectra of the reference $[Ru(bpy)_3]^{2+}$ (14 µM in aerated H_2O, dark grey trace), neat $[Ru(bpz)_3]^{2+}$ (13 µM, green dotted trace), and the donor-acceptor pair $[Ru(bpz)_3]^{2+}$ (13 µM) / TAA-I (200 µM) in aerated CH_3CN at 293 K (green solid trace). All three solutions have identical absorbance at 452 nm, and the minor absorption difference around 500 nm between the neat $[Ru(bpz)_3]^{2+}$ solution and the solution containing the donor-acceptor pair likely arises from some small amount of $[Ru(bpz)_3]^{+}$ from $[Ru(bpz)_3]^{2+}$ via electron transfer with TAA-I induced by ambient light. (b) Transient absorption decays of the reference $[Ru(bpy)_3]^{2+}$ at 455 nm (dark grey trace) and the $[Ru(bpz)_3]^{2+}$ / TAA-I donor-acceptor pair (green trace) from (a) at 763 nm. The relative concentrations of photoproducts (^{3}MLCT-excited $[Ru(bpy)_3]^{2+}$ at 455 nm and TAA-$I^{\bullet+}$ at 763 nm) were derived from the measured ΔOD_R and ΔOD_S values and their respective Δε values at the relevant observation wavelengths ($\Delta\varepsilon_{455} = \Delta\varepsilon_R$ = -10100 M^{-1} cm^{-1},[30] $\Delta\varepsilon_{685} = \Delta\varepsilon_S$ = 16800 ± 900 M^{-1} cm^{-1}). This analysis yields a Φ_{CE} value of 60 ± 3 % for the $[Ru(bpz)_3]^{2+}$ / TAA-I pair in aerated CH_3CN. Excitation of both the reference and the sample occurred at 452 nm with a ns-pulsed laser (pulse energy ~ 13 mJ), $A_S = A_R$ in this case.

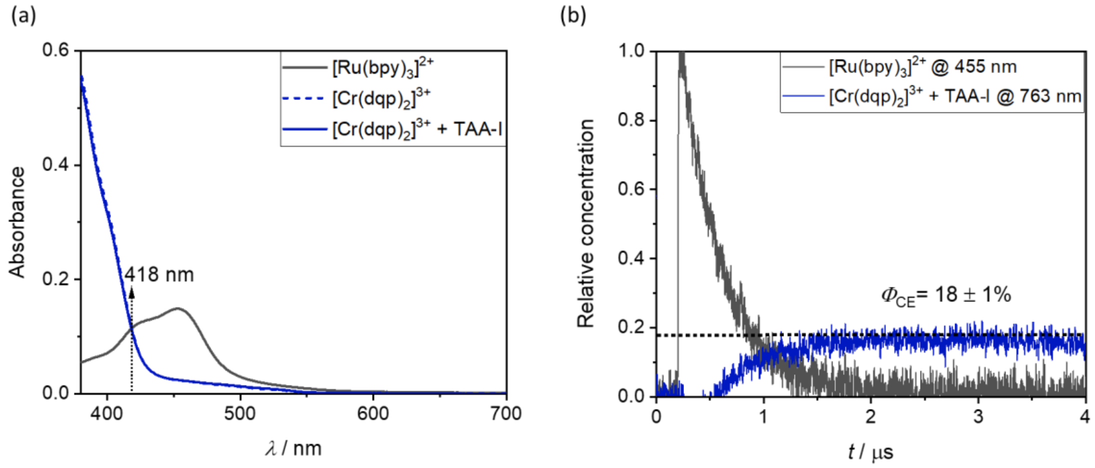

Supplementary Fig. 68: (a) Absorption spectra of the reference $[Ru(bpy)_3]^{2+}$ (14 µM in aerated H_2O, dark grey trace), neat $[Cr(dqp)_2]^{3+}$ (30 µM, dark blue dotted trace), and the donor-acceptor pair $[Cr(dqp)_2]^{3+}$ (30 µM) / TAA-I (200 µM) in aerated CH_3CN at 293 K (dark blue solid trace). All three solutions have identical absorbance at 418 nm. (b) Transient absorption decays of the reference $[Ru(bpy)_3]^{2+}$ at 455 nm (dark grey trace) and the $[Cr(dqp)_2]^{3+}$ / TAA-I donor-acceptor pair (dark blue trace) from (a) at 763 nm. The relative concentrations of photoproducts (^{3}MLCT-excited $[Ru(bpy)_3]^{2+}$ at 455 nm and TAA-$I^{\bullet+}$ at 763 nm) were derived from the measured ΔOD_R and ΔOD_S values and their respective $\Delta\varepsilon$ values at the relevant observation wavelengths ($\Delta\varepsilon_{455} = \Delta\varepsilon_R$ = -10100 M^{-1} cm^{-1},[30] $\Delta\varepsilon_{704} = \Delta\varepsilon_S$ = 16800 ± 900 M^{-1} cm^{-1}). This analysis yields a Φ_{CE} value of 18 ± 1 % for the $[Cr(dqp)_2]^{3+}$ / TAA-I pair in aerated CH_3CN. Excitation of both the reference and the sample occurred at 418 nm with a ns-pulsed laser (pulse energy ~ 9 mJ), $A_S = A_R$ in this case.

7.7 DMA

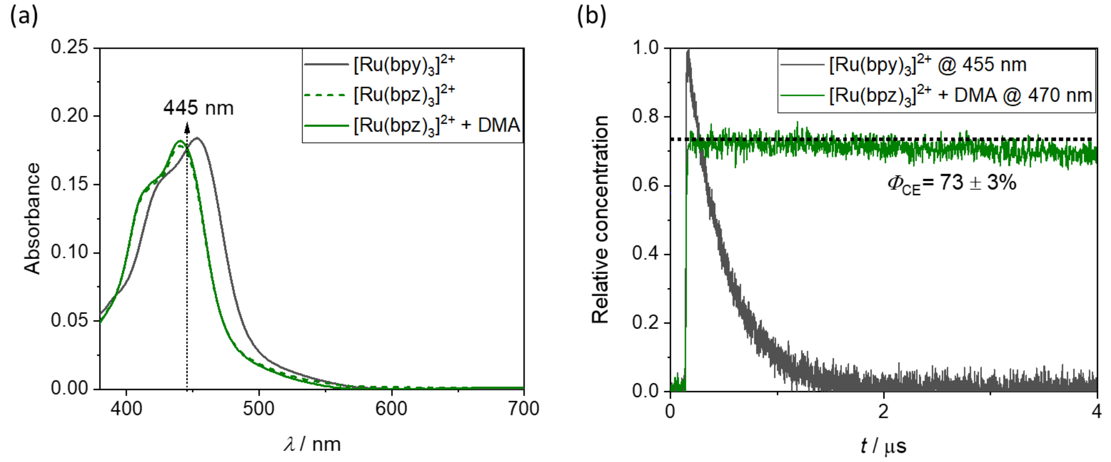

Supplementary Fig. 69: (a) Absorption spectra of the reference $[Ru(bpy)_3]^{2+}$ (16 µM in aerated H_2O, dark grey trace), neat $[Ru(bpz)_3]^{2+}$ (13 µM, green dotted trace), and the donor-acceptor pair $[Ru(bpz)_3]^{2+}$ (13 µM) / DMA (100 mM) in aerated CH_3CN at 293 K (green solid trace). All three solutions have identical absorbance at 449 nm. (b) Transient absorption decays of the reference $[Ru(bpy)_3]^{2+}$ at 455 nm (dark grey trace) and the donor-acceptor pair from (a) at 470 nm (green trace). The relative concentration of photoproducts (^{3}MLCT-excited $[Ru(bpy)_3]^{2+}$ at 455 nm and $DMA^{\bullet+}$ at 470 nm as well as $[Ru(bpz)_3]^{+}$ at 470 nm) were derived from the measured ΔOD_R and ΔOD_S values and their respective $\Delta\varepsilon$ values at the relevant observation wavelengths ($\Delta\varepsilon_{455} = \Delta\varepsilon_R$ = -10100 M^{-1} cm^{-1},[30] $\Delta\varepsilon_{470,sum} = \Delta\varepsilon_S = \Delta\varepsilon_{470}([Ru(bpz)_3]^{+}) + \Delta\varepsilon_{470}(DMA^{\bullet+})$ = 8800 ± 400 M^{-1} cm^{-1}). This analysis yields a Φ_{CE} value of 72 ± 3 % for the $[Ru(bpz)_3]^{2+}$ / DMA pair in CH_3CN. Excitation of both the reference and the sample occurred at 445 nm with a ns-pulsed laser (pulse energy ~ 12 mJ), $A_S = A_R$ in this case.

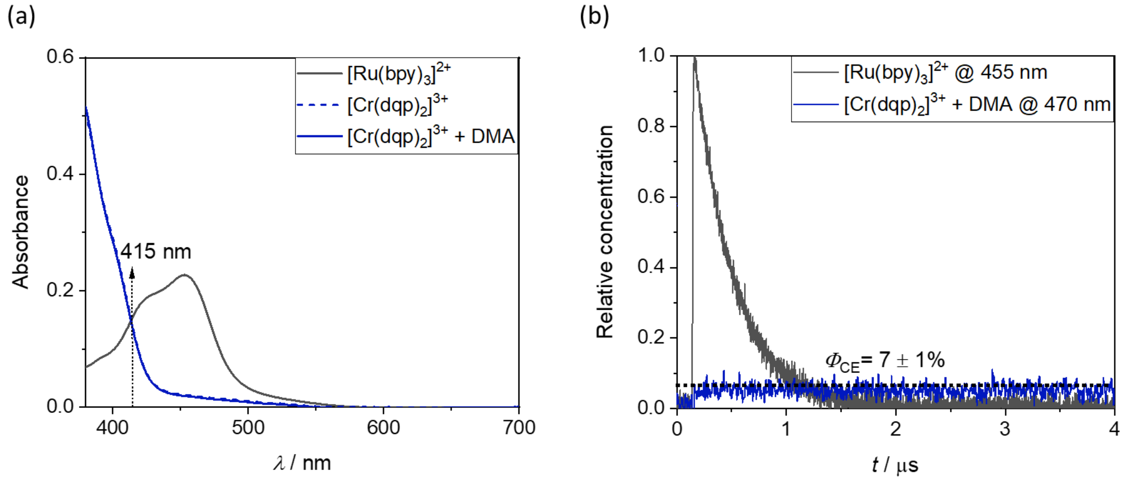

Supplementary Fig. 70: (a) Absorption spectra of the reference $[Ru(bpy)_3]^{2+}$ (18 µM in aerated H_2O, dark grey trace), neat $[Cr(dqp)_2]^{3+}$ (30 µM, dark blue dotted trace), and the donor-acceptor pair $[Cr(dqp)_2]^{3+}$ (30 µM) / DMA (100 mM) in aerated CH_3CN at 293 K (dark blue solid trace). All three solutions have identical absorbance at 415 nm. (b) Transient absorption decays of the reference $[Ru(bpy)_3]^{2+}$ at 455 nm (dark grey trace) and the $[Cr(dqp)_2]^{3+}$ / DMA donor-acceptor pair from (a) at 470 nm (dark blue trace). The relative concentration of photoproducts (^{3}MLCT-excited $[Ru(bpy)_3]^{2+}$ at 455 nm and $DMA^{\bullet+}$ at 470 nm as well as $[Cr(dqp)_2]^{2+}$ at 470 nm) were derived from the measured ΔOD_R and ΔOD_S values and their respective $\Delta\varepsilon$ values at the relevant observation wavelengths ($\Delta\varepsilon_{455} = \Delta\varepsilon_R$ = -10100 M^{-1} cm^{-1},[30] $\Delta\varepsilon_{470,sum} = \Delta\varepsilon_S = \Delta\varepsilon_{470}([Cr(dqp)_2]^{2+}) + \Delta\varepsilon_{470}(DMA^{\bullet+})$ = 6600 ± 400 M^{-1} cm^{-1}). This analysis yields a Φ_{CE} value of 7 ± 1 % for the $[Cr(dqp)_2]^{3+}$/ DMA pair in CH_3CN. Excitation of both the reference and the sample occurred at 415 nm with a ns-pulsed laser (pulse energy ~ 8 mJ), $A_S = A_R$ in this case.

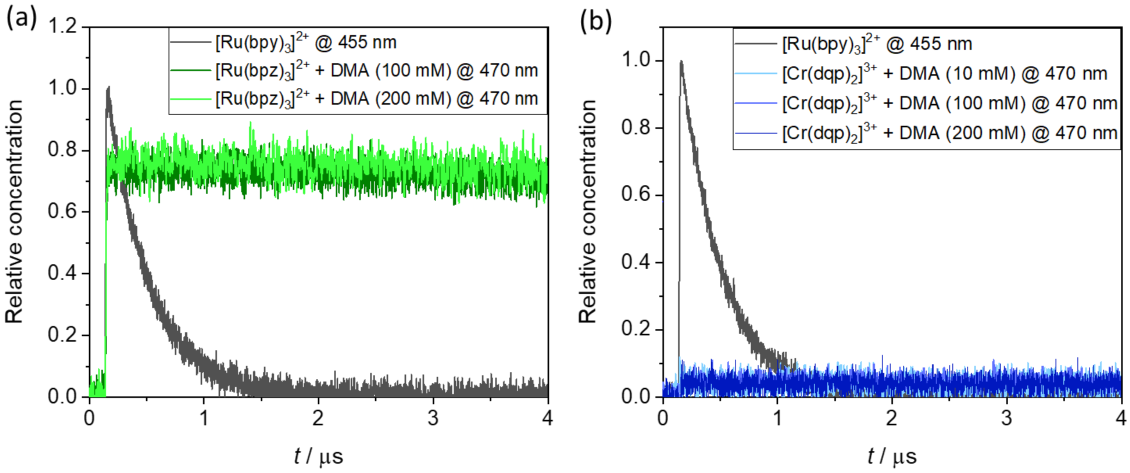

Supplementary Fig. 71: a) Transient absorption decays of the reference $[Ru(bpy)_3]^{2+}$ at 455 nm (16 µM in aerated H_2O, dark grey trace) and the donor-acceptor pair $[Ru(bpz)_3]^{2+}$ / DMA at 470 nm in aerated CH_3CN at 293 K, with the $[Ru(bpz)_3]^{2+}$ concentration of 13 µM and DMA of 100 mM (dark green, as in Supplementary Fig. 69b) and 200 mM (bright green). Excitation of both the reference and the sample occurred at 445 nm with a ns-pulsed laser (pulse energy ~ 12 mJ), $A_S = A_R$ in this case. b) Transient absorption decays of the reference $[Ru(bpy)_3]^{2+}$ at 455 nm (18 µM in aerated H_2O, dark grey trace) and the donor-acceptor pair $[Cr(dqp)_2]^{3+}$ / DMA at 470 nm in aerated CH_3CN at 293 K, with the $[Cr(dqp)_2]^{3+}$ concentration of 30 µM and 10 mM DMA (bright blue), 100 mM DMA (blue, as Supplementary Fig. 70b), and 200 mM DMA (dark blue). Excitation of both the reference and the sample occurred at 415 nm with a ns-pulsed laser (pulse energy ~ 8 mJ), $A_S = A_R$ in this case. No clear change in the cage escape quantum yield (relative concentration) was observed for both donor-acceptor pairs when the concentration of the DMA donors (in large excess to acceptor) was increased.

7.8 DMT

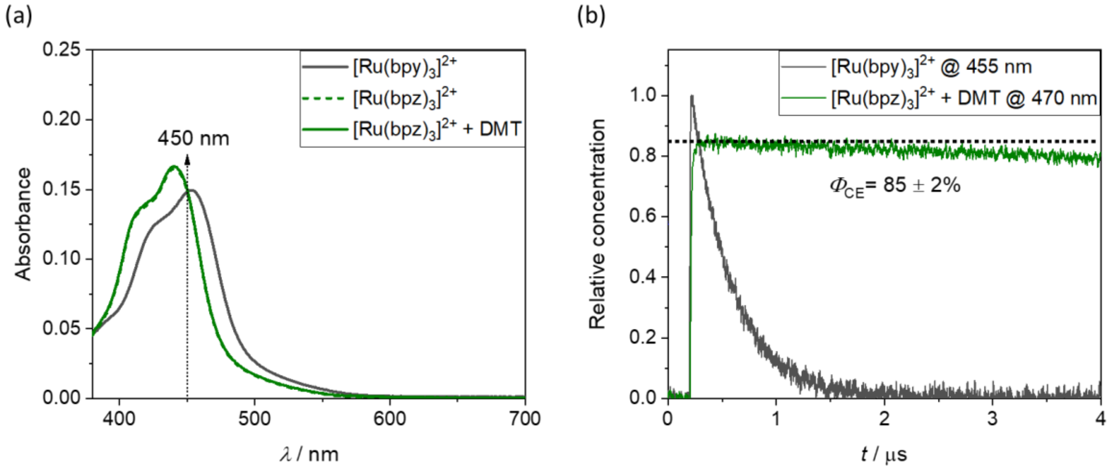

Supplementary Fig. 72: (a) Absorption spectra of the reference $[Ru(bpy)_3]^{2+}$ (13 µM in aerated H_2O, dark grey trace), neat $[Ru(bpz)_3]^{2+}$ (13 µM, green dotted trace), and the donor-acceptor pair $[Ru(bpz)_3]^{2+}$ (13 µM) / DMT (10 mM) in aerated CH_3CN at 293 K (green solid trace). All three solutions have identical absorbance at 450 nm. (b) Transient absorption decays of the reference $[Ru(bpy)_3]^{2+}$ at 455 nm (dark grey trace) and the $[Ru(bpz)_3]^{2+}$ / DMT donor-acceptor pair from (a) at 470 nm (green trace). The relative concentration of photoproducts (^{3}MLCT-excited $[Ru(bpy)_3]^{2+}$ at 455 nm and $DMT^{\bullet+}$ at 470 nm as well as $[Ru(bpz)_3]^{+}$ at 470 nm) were derived from the measured ΔOD_R and ΔOD_S values and their respective $\Delta\varepsilon$ values at the relevant observation wavelengths ($\Delta\varepsilon_{455} = \Delta\varepsilon_R = -10100\ M^{-1}\ cm^{-1}$,[30] $\Delta\varepsilon_{470,sum} = \Delta\varepsilon_S = \Delta\varepsilon_{470}([Ru(bpz)_3]^{+}) + \Delta\varepsilon_{470}(DMT^{\bullet+}) = 13800 \pm 300\ M^{-1}\ cm^{-1}$). This analysis yields a Φ_{CE} value of 85 ± 2 % for the $[Ru(bpz)_3]^{2+}$ / DMT pair in CH_3CN. Excitation of both the reference and the sample occurred at 450 nm with a ns-pulsed laser (pulse energy ~13 mJ), $A_S = A_R$ in this case.

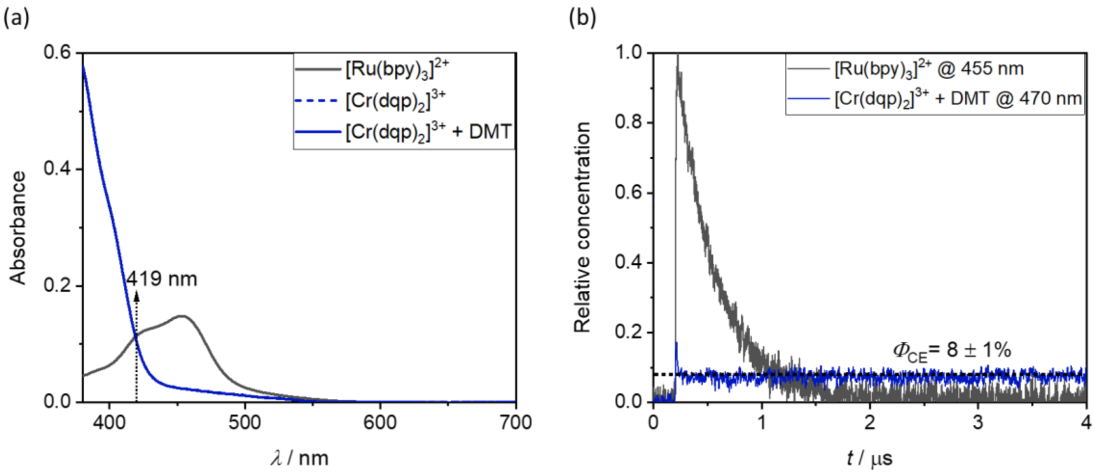

Supplementary Fig. 73: (a) Absorption spectra of the reference $[Ru(bpy)_3]^{2+}$ (13 µM in aerated H_2O, dark grey trace), neat $[Cr(dqp)_2]^{3+}$ (30 µM, dark blue dotted trace), and the donor-acceptor pair $[Cr(dqp)_2]^{3+}$ (30 µM) / DMT (10 mM) in aerated CH_3CN at 293 K (dark blue solid trace). All three solutions have identical absorbance at 419 nm. (b) Transient absorption decays of the reference $[Ru(bpy)_3]^{2+}$ at 455 nm (dark grey trace) and the $[Cr(dqp)_2]^{3+}$ / DMT donor-acceptor pair from (a) at 470 nm (dark blue trace). The relative concentration of photoproducts (^{3}MLCT-excited $[Ru(bpy)_3]^{2+}$ at 455 nm and $DMT^{\bullet+}$ at 470 nm as well as $[Cr(dqp)_2]^{2+}$ at 470 nm) were derived from the measured ΔOD_R and ΔOD_S values and their respective $\Delta\varepsilon$ values at the relevant observation wavelengths ($\Delta\varepsilon_{455} = \Delta\varepsilon_R$ = -10100 M^{-1} cm^{-1},[30] $\Delta\varepsilon_{470,sum} = \Delta\varepsilon_S = \Delta\varepsilon_{470}([Cr(dqp)_2]^{2+}) + \Delta\varepsilon_{470}(DMT^{\bullet+})$ = 11100 ± 300 M^{-1} cm^{-1}). This analysis yields a Φ_{CE} value of 8 ± 1 % for the $[Cr(dqp)_2]^{3+}$/ DMT pair in CH_3CN. Excitation of both the reference and the sample occurred at 419 nm with a ns-pulsed laser (pulse energy ~ 8 mJ), $A_S = A_R$ in this case.

7.9 DMA-OMe

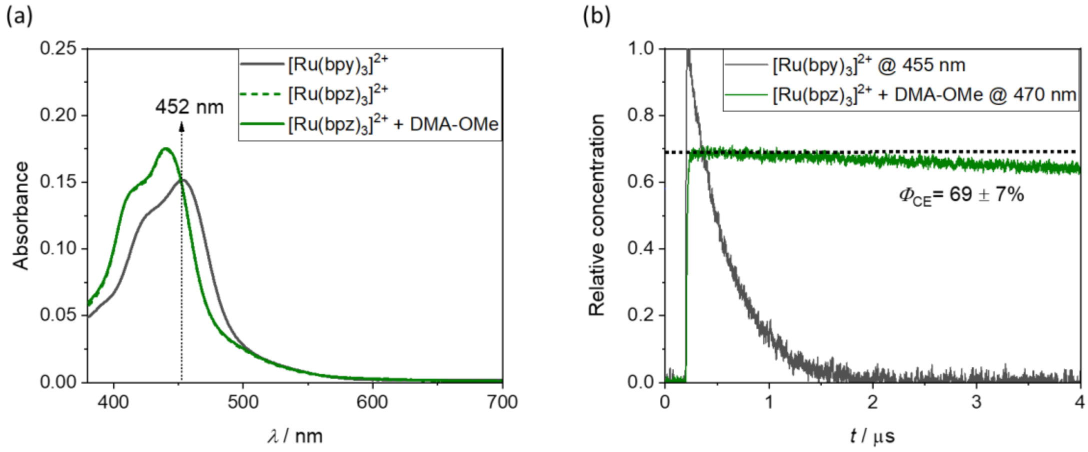

Supplementary Fig. 74: (a) Absorption spectra of the reference $[Ru(bpy)_3]^{2+}$ (13 µM in aerated H_2O, dark grey trace), neat $[Ru(bpz)_3]^{2+}$ (13 µM, green dotted trace), and the donor-acceptor pair $[Ru(bpz)_3]^{2+}$ (13 µM) / DMA-OMe (10 mM) in aerated CH_3CN at 293 K (green solid trace). All three solutions have identical absorbance at 452 nm. (b) Transient absorption decays of the reference $[Ru(bpy)_3]^{2+}$ at 455 nm (dark grey trace) and the $[Ru(bpz)_3]^{2+}$ / DMA-OMe donor-acceptor pair from (a) at 470 nm (green trace). The relative concentration of photoproducts (^{3}MLCT-excited $[Ru(bpy)_3]^{2+}$ at 455 nm and DMA-$OMe^{\bullet+}$ at 470 nm as well as $[Ru(bpz)_3]^{+}$ at 470 nm) were derived from the measured ΔOD_R and ΔOD_S values and their respective $\Delta\varepsilon$ values at the relevant observation wavelengths ($\Delta\varepsilon_{455} = \Delta\varepsilon_R$ = -10100 M^{-1} cm^{-1},[30] $\Delta\varepsilon_{470,sum} = \Delta\varepsilon_S = \Delta\varepsilon_{470}([Ru(bpz)_3]^{+}) + \Delta\varepsilon_{470}(DMA\text{-}OMe^{\bullet+})$ = 20600 ± 2000 M^{-1} cm^{-1}). This analysis yields a Φ_{CE} value of 69 ± 7 % for the $[Ru(bpz)_3]^{2+}$ / DMA-OMe pair in CH_3CN. Excitation of both the reference and the sample occurred at 452 nm with a ns-pulsed laser (pulse energy ~ 13 mJ), $A_S = A_R$ in this case.

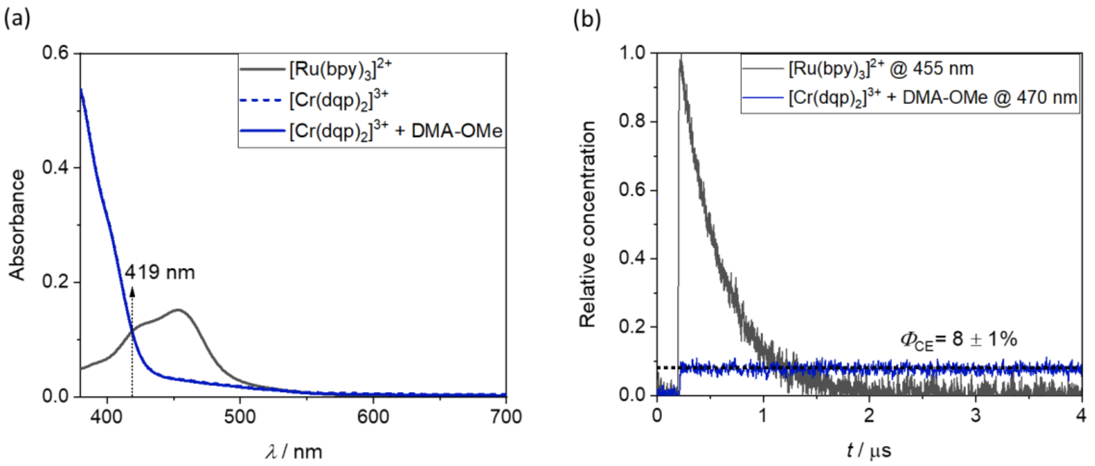

Supplementary Fig. 75: (a) Absorption spectra of the reference $[Ru(bpy)_3]^{2+}$ (13 µM in aerated H_2O, dark grey trace), neat $[Cr(dqp)_2]^{3+}$ (30 µM, dark blue dotted trace), and the donor-acceptor pair $[Cr(dqp)_2]^{3+}$ (30 µM) / DMA-OMe (10 mM) in aerated CH_3CN at 293 K (dark blue solid trace). All three solutions have identical absorbance at 419 nm. (b) Transient absorption decays of the reference $[Ru(bpy)_3]^{2+}$ at 455 nm (dark grey trace) and the $[Cr(dqp)_2]^{3+}$ / DMA-OMe donor-acceptor pair from (a) at 470 nm (dark blue trace). The relative concentration of photoproducts (^{3}MLCT-excited $[Ru(bpy)_3]^{2+}$ at 455 nm and DMA-OMe$^{•+}$ at 470 nm as well as $[Cr(dqp)_2]^{2+}$ at 470 nm) were derived from the measured ΔOD_R and ΔOD_S values and their respective $\Delta\varepsilon$ values at the relevant observation wavelengths ($\Delta\varepsilon_{455}$ = $\Delta\varepsilon_R$ = -10100 M^{-1} cm^{-1},[30] $\Delta\varepsilon_{470,sum}$ = $\Delta\varepsilon_S$ = $\Delta\varepsilon_{470}([Cr(dqp)_2]^{2+})$ + $\Delta\varepsilon_{470}$(DMA-OMe$^{•+}$) = 18300 ± 2000 M^{-1} cm^{-1}). This analysis yields a Φ_{CE} value of 8 ± 1 % for the $[Cr(dqp)_2]^{3+}$/ DMA-OMe pair in CH_3CN. Excitation of both the reference and the sample occurred at 419 nm with a ns-pulsed laser (pulse energy ~ 8 mJ), A_S = A_R in this case.

7.10 THIQ

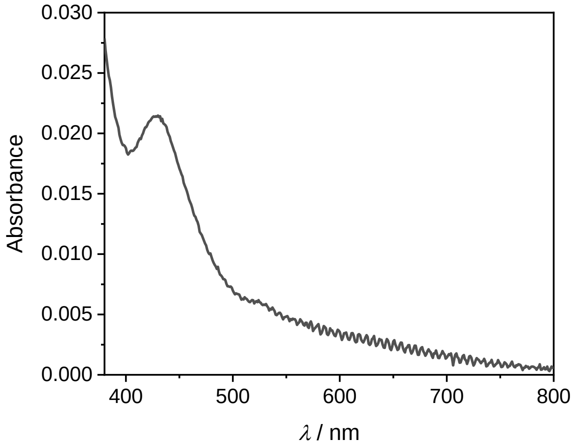

Supplementary Fig. 76: UV/vis absorption spectrum of THIQ (2 mM) in dry aerated CH_3CN at 293 K.

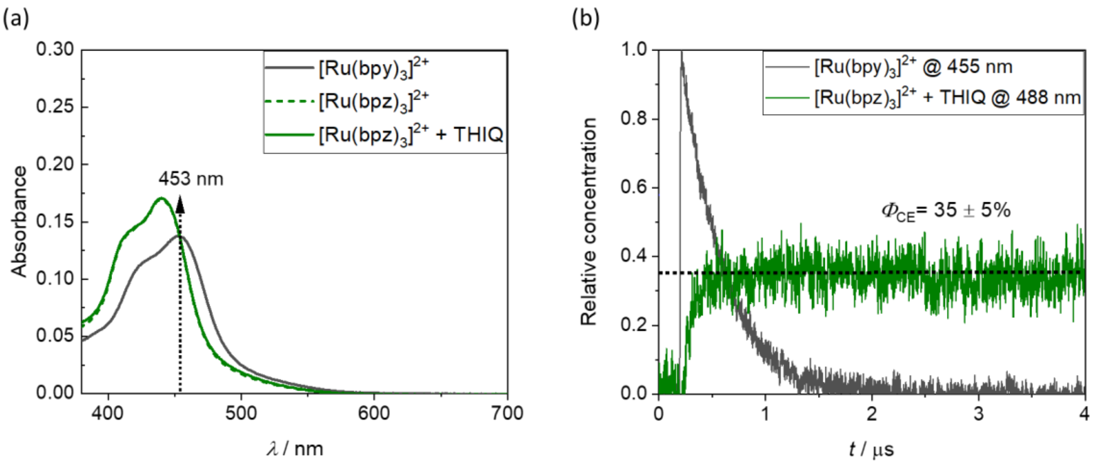

Supplementary Fig. 77: (a) Absorption spectra of the reference $[Ru(bpy)_3]^{2+}$ (12 µM in aerated H_2O, dark grey trace), neat $[Ru(bpz)_3]^{2+}$ (13 µM, green dotted trace), and the donor-acceptor pair $[Ru(bpz)_3]^{2+}$ (13 µM) / THIQ (2 mM) in aerated CH_3CN at 293 K (green solid trace). All three solutions have identical absorbance at 453 nm. The intrinsic absorption of THIQ at 453 nm is negligible. (b) Transient absorption decays of the reference $[Ru(bpy)_3]^{2+}$ at 455 nm (dark grey trace) and the $[Ru(bpz)_3]^{2+}$ / THIQ donor-acceptor pair (green trace) from (a) at 488 nm. According to the spectro-electrochemical absorption spectrum of THIQ (Supplementary Fig. 14), the $THIQ^{\bullet+}$ species is optically transparent above 450 nm. This permits the Φ_{CE} determination using the ΔOD_S value of the donor-acceptor pair at 488 nm, which arises solely from $[Ru(bpz)_3]^{+}$. The relative concentrations of photoproducts (^{3}MLCT-excited $[Ru(bpy)_3]^{2+}$ at 455 nm and $[Ru(bpz)_3]^{+}$ at 488 nm) were derived from the measured ΔOD_R and ΔOD_S values and their respective $\Delta\varepsilon$ values at the relevant observation wavelengths ($\Delta\varepsilon_{455} = \Delta\varepsilon_R$ = -10100 M^{-1} cm^{-1},[30] $\Delta\varepsilon_{488} = \Delta\varepsilon_S$ = 6200 M^{-1} cm^{-1}). This analysis yields a Φ_{CE} value of 35 ± 5 % for the $[Ru(bpz)_3]^{2+}$ / THIQ pair in CH_3CN. Excitation of both the reference and the sample occurred at 453 nm with a ns-pulsed laser (pulse energy ~ 13 mJ), $A_S = A_R$ in this case.

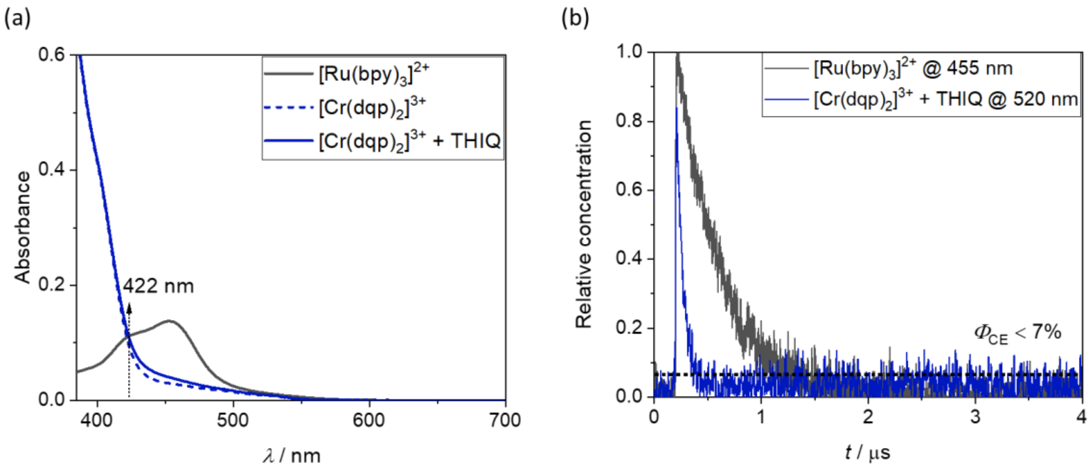

Supplementary Fig. 78: (a) Absorption spectra of the reference $[Ru(bpy)_3]^{2+}$ (12 µM in aerated H_2O, dark grey trace), neat $[Cr(dqp)_2]^{3+}$ (30 µM, dark blue dotted line), and the donor-acceptor pair $[Cr(dqp)_2]^{3+}$ (30 µM) / THIQ (2 mM) in aerated CH_3CN at 293 K (dark blue solid trace). All three solutions have identical absorbance at 422 nm. The intrinsic absorption of THIQ at 422 nm is negligible. (b) Transient absorption decays of the reference $[Ru(bpy)_3]^{2+}$ at 455 nm (dark grey trace) and the $[Cr(dqp)_2]^{3+}$ / THIQ donor-acceptor pair (dark blue trace) from (a) at 520 nm. The short-lived species in the dark blue trace corresponds to the residual of excited-state absorption of $[Cr(dqp)_2]^{3+}$. According to the spectro-electrochemical absorption spectrum of THIQ (Supplementary Fig. 14), the $THIQ^{\bullet+}$ species is optically transparent above 450 nm. This permits the Φ_{CE} determination using the ΔOD_S value of the donor-acceptor pair at 520 nm, which arises solely from $[Cr(dqp)_2]^{2+}$. The relative concentrations of photoproducts (^{3}MLCT-excited $[Ru(bpy)_3]^{2+}$ at 455 nm and $[Cr(dqp)_2]^{2+}$ at 520 nm) were derived from the measured ΔOD_R and ΔOD_S values and their respective $\Delta\varepsilon$ values at the relevant observation wavelengths ($\Delta\varepsilon_{455} = \Delta\varepsilon_R$ = -10100 M^{-1} cm^{-1},[30] $\Delta\varepsilon_{520} = \Delta\varepsilon_S$ = 3000 M^{-1} cm^{-1}). Due to the high signal to noise ratio and the low $\Delta\varepsilon_{520}$ ($[Cr(dqp)_2]^{2+}$), a Φ_{CE} below 7 % was estimated for the $[Cr(dqp)_2]^{3+}$ / THIQ pair in aerated CH_3CN. Excitation of both the reference and the sample occurred at 422 nm with a ns-pulsed laser (pulse energy ~ 9 mJ), $A_S = A_R$ in this case.

7.11 TEA

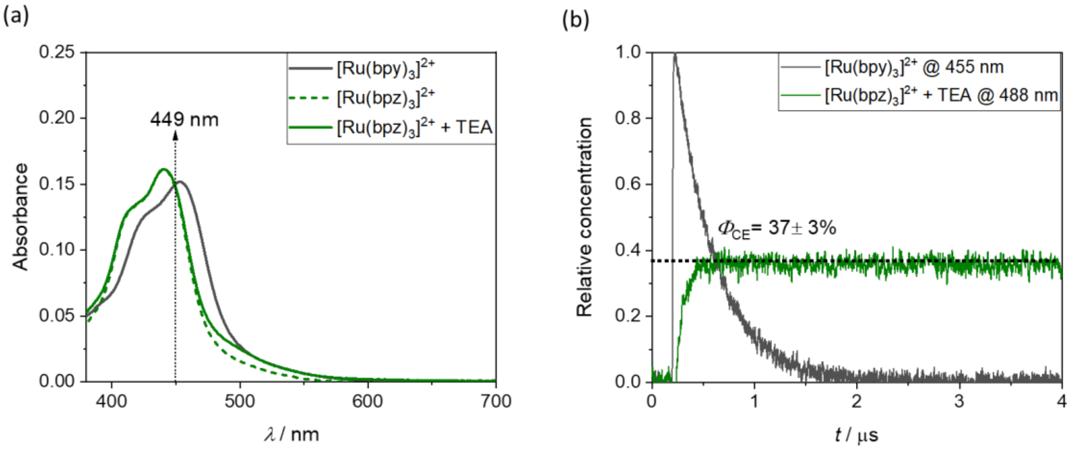

Supplementary Fig. 79: (a) Absorption spectra of the reference $[Ru(bpy)_3]^{2+}$ (12 μM in aerated H_2O, dark grey trace), neat $[Ru(bpz)_3]^{2+}$ (13 μM, green dotted trace), and the donor-acceptor pair $[Ru(bpz)_3]^{2+}$ (13 μM) / TEA (10 mM) in aerated CH_3CN at 293 K (green solid trace). All three solutions have identical absorbance at 449 nm. The absorption difference between 450 nm and 600 nm between the green traces is tentatively attributed to the formation of small amounts of $[Ru(bpy)_3]^{+}$ as a result of exposure to ambient light. (b) Transient absorption decays of the reference $[Ru(bpy)_3]^{2+}$ at 455 nm (dark grey trace) and the $[Ru(bpz)_3]^{2+}$ / TEA donor-acceptor pair (green trace) from (a) at 488 nm. The optical transparence of $TEA^{\bullet +}$ in the visible regime permits the Φ_{CE} determination using the ΔOD_S value of the donor-acceptor pair at 488 nm, which arises solely from $[Ru(bpz)_3]^{+}$. The relative concentrations of photoproducts (^{3}MLCT-excited $[Ru(bpy)_3]^{2+}$ at 455 nm and $[Ru(bpz)_3]^{+}$ at 488 nm) were derived from the measured ΔOD_R and ΔOD_S values and their respective $\Delta\varepsilon$ values at the relevant observation wavelengths ($\Delta\varepsilon_{455} = \Delta\varepsilon_R$ = -10100 M^{-1} cm^{-1},[30] $\Delta\varepsilon_{488} = \Delta\varepsilon_S$ = 6200 M^{-1} cm^{-1}). This analysis yields a Φ_{CE} value of 37 ± 3 % for the $[Ru(bpz)_3]^{2+}$ / TEA pair in CH_3CN. Excitation of both the reference and the sample occurred at 449 nm with a ns-pulsed laser (pulse energy ~ 13 mJ), $A_S = A_R$ in this case.

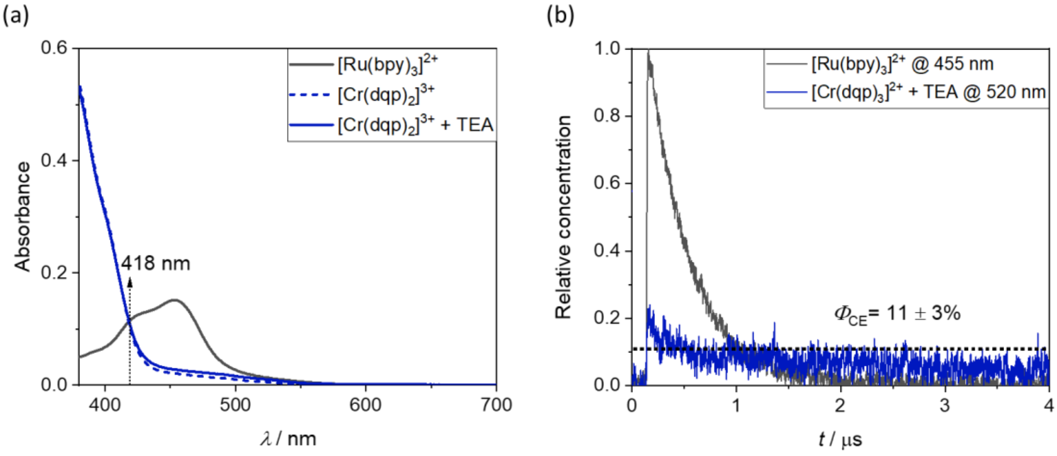

Supplementary Fig. 80: (a) Absorption spectra of the reference $[Ru(bpy)_3]^{2+}$ (12 μM in aerated H_2O, dark grey trace), neat $[Cr(dqp)_2]^{3+}$ (30 μM, dark blue dotted line), and the donor-acceptor pair $[Cr(dqp)_2]^{3+}$ (30 μM) / TEA (40 mM) in aerated CH_3CN at 293 K (dark blue solid trace). All three solutions have identical absorbance at 418 nm. The absorption difference between 420 nm and 550 nm between the blue traces is tentatively attributed to the formation of small amounts of $[Cr(dqp)_2]^{2+}$ as a result of exposure to ambient light. (b) Transient absorption decays of the reference $[Ru(bpy)_3]^{2+}$ at 455 nm (dark grey trace) and the $[Cr(dqp)_2]^{3+}$ / TEA donor-acceptor pair (dark blue trace) from (a) at 520 nm. The optical transparence of $TEA^{\bullet +}$ in the visible regime permits the Φ_{CE} determination using the ΔOD_S value of the donor-acceptor pair at 520 nm, which arises solely from $[Cr(dqp)_2]^{2+}$. The initial decay at 520 nm up to 1 μs (dark blue trace) is assigned to the residual excited state absorption of $[Cr(dqp)_2]^{3+}$, owing to slow photoinduced electron transfer from TEA to $[Cr(dqp)_2]^{3+}$.The relative concentrations of photoproducts (^{3}MLCT-excited $[Ru(bpy)_3]^{2+}$ at 455 nm and $[Cr(dqp)_2]^{2+}$ at 520 nm) were derived from the measured ΔOD_R and ΔOD_S values and their respective $\Delta\varepsilon$ values at the relevant observation wavelengths ($\Delta\varepsilon_{455} = \Delta\varepsilon_R$ = -10100 M^{-1} cm^{-1},[30] $\Delta\varepsilon_{520} = \Delta\varepsilon_S$ = 3000 M^{-1} cm^{-1}). This analysis yields a Φ_{CE} value of 11 ± 3 % for the $[Cr(dqp)_2]^{3+}$/ TEA pair in CH_3CN. Excitation of both the reference and the sample occurred at 418 nm with a ns-pulsed laser (pulse energy ~ 9 mJ), $A_S = A_R$ in this case.

7.12 DIPEA

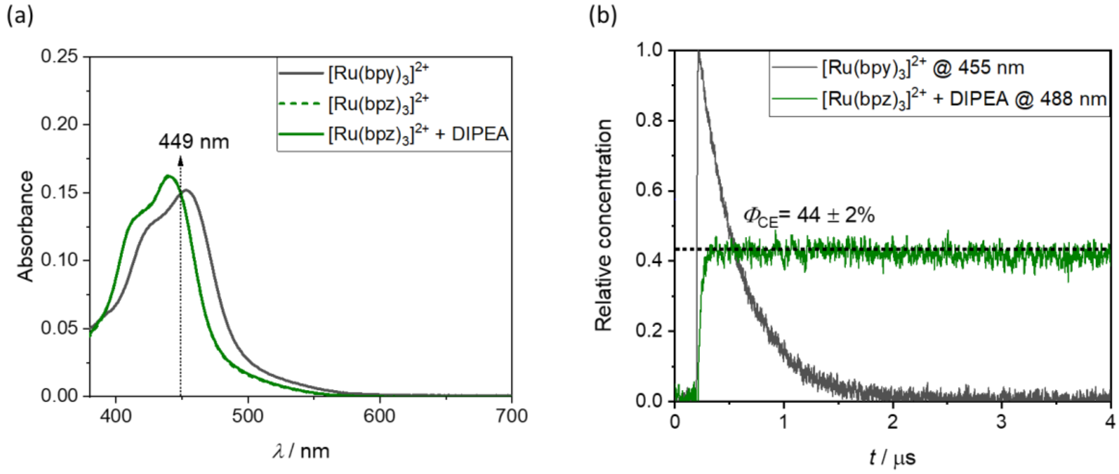

Supplementary Fig. 81: (a) Absorption spectra of the reference $[Ru(bpy)_3]^{2+}$ (12 µM in aerated H_2O, dark grey trace), neat $[Ru(bpz)_3]^{2+}$ (13 µM, green dotted trace), and the donor-acceptor pair $[Ru(bpz)_3]^{2+}$ (13 µM) / DIPEA (10 mM) in aerated CH_3CN at 293 K (green solid trace). All three solutions have identical absorbance at 449 nm. (b) Transient absorption decays of the reference $[Ru(bpy)_3]^{2+}$ at 455 nm (dark grey trace) and the $[Ru(bpz)_3]^{2+}$ / DIPEA donor-acceptor pair (green trace) from (a) at 488 nm. The optical transparence of $DIPEA^{•+}$ in the visible regime permits the Φ_{CE} determination using the ΔOD_S value of the donor-acceptor pair at 488 nm, which arises solely from $[Ru(bpz)_3]^{+}$. The relative concentrations of photoproducts (^{3}MLCT-excited $[Ru(bpy)_3]^{2+}$ at 455 nm and $[Ru(bpz)_3]^{+}$ at 488 nm) were derived from the measured ΔOD_R and ΔOD_S values and their respective $\Delta\varepsilon$ values at the relevant observation wavelengths ($\Delta\varepsilon_{455} = \Delta\varepsilon_R$ = -10100 M^{-1} cm^{-1},[30] $\Delta\varepsilon_{488} = \Delta\varepsilon_S$ = 6200 M^{-1} cm^{-1}). This analysis yields a Φ_{CE} value of 44 ± 2 % for the $[Ru(bpz)_3]^{2+}$ / DIPEA pair in CH_3CN. Excitation of both the reference and the sample occurred at 449 nm with a ns-pulsed laser (pulse energy ~ 13 mJ), $A_S = A_R$ in this case.

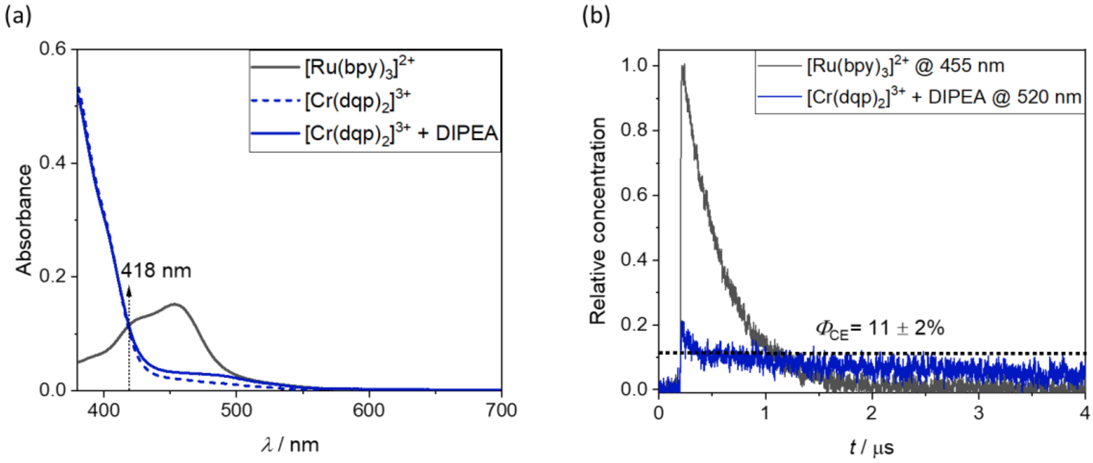

Supplementary Fig. 82: (a) Absorption spectra of the reference $[Ru(bpy)_3]^{2+}$ (13 µM in aerated H_2O, dark grey trace), neat $[Cr(dqp)_2]^{3+}$ (30 µM, dark blue dotted line), and the donor-acceptor pair $[Cr(dqp)_2]^{3+}$ (30 µM) / DIPEA (10 mM) in aerated CH_3CN at 293 K (dark blue solid trace). All three solutions have identical absorbance at 418 nm. The absorption difference between 420 nm and 550 nm between the blue traces is tentatively attributed to the formation of small amounts of $[Cr(dqp)_2]^{2+}$ as a result of exposure to ambient light. (b) Transient absorption decays of the reference $[Ru(bpy)_3]^{2+}$ at 455 nm (dark grey trace) and the $[Cr(dqp)_2]^{3+}$ / DIPEA donor-acceptor pair (dark blue trace) from (a) at 520 nm. The optical transparence of $DIPEA^{•+}$ in the visible regime permits the Φ_{CE} determination using the ΔOD_S value of the donor-acceptor pair at 520 nm, which arises solely from $[Cr(dqp)_2]^{2+}$. The initial decay at 520 nm up to 1 µs (dark blue trace) is attributed to the residual excited state absorption of $[Cr(dqp)_2]^{3+}$, owing to slow photoinduced electron transfer from DIPEA to $[Cr(dqp)_2]^{3+}$. The relative concentrations of photoproducts (^{3}MLCT-excited $[Ru(bpy)_3]^{2+}$ at 455 nm and $[Cr(dqp)_2]^{2+}$ at 520 nm) were derived from the measured ΔOD_R and ΔOD_S values and their respective $\Delta\varepsilon$ values at the relevant observation wavelengths ($\Delta\varepsilon_{455}$ = $\Delta\varepsilon_R$ = -10100 M^{-1} cm^{-1},[30] $\Delta\varepsilon_{520} = \Delta\varepsilon_S$ = 3000 M^{-1} cm^{-1}). This analysis yields a Φ_{CE} value of 11 ± 2 % for the $[Cr(dqp)_2]^{3+}$/ DIPEA pair in aerated CH_3CN. Excitation of both the reference and the sample occurred at 418 nm with a ns-pulsed laser (pulse energy ~ 9 mJ), $A_S = A_R$ in this case.

7.13 Summary

Supplementary Table 1: Summary of cage escape quantum yields Φ_{CE}, rate constants for photoinduced electron transfer, and redox potentials of the electron donors and the acceptors. The experimental errors associated with the reported Φ_{CE} values arise mainly from the uncertainties associated with the determination of the change in molar extinction coefficient $\Delta\varepsilon$ (**eq. 1**) upon donor oxidation and acceptor reduction (section 6). The relative errors are similar for both $[Ru(bpz)_3]^{2+}$ and $[Cr(dqp)_2]^{3+}$.

Electron donor	**TAA-OMe**	**TAA-PEG$_3$**	**TAA-PEG$_7$**	**TAA-Cl**	**TAA-Br**	**TAA-I**	**DMA**	**DMT**	**DMA-OMe**	**THIQ**	**TEA**	**DIPEA**
Redox potential / V vs SCE	0.58	0.68	0.78	1.04[39]	1.10[40]	1.19	0.81[41]	0.72[41]	0.33[41]	0.87	0.96[41]	0.90[41]
	**$[Ru(bpz)_3][PF_6]_2$**: $^*[Ru(bpz)_3]^{2+}$/$[Ru(bpz)_3]^{+}$: 1.45 V vs SCE; $[Ru(bpz)_3]^{2+}$/$[Ru(bpz)_3]^{+}$: -0.73 V vs SCE[6]											
Φ_{CE} / %	58 ± 2	62 ± 2	78 ± 2	65 ± 3	87 ± 4	60 ± 3	73 ± 3	85 ± 2	69 ± 7	35 ± 5	37 ± 3	44 ± 2
k_q/10^9 M^{-1} s^{-1}	10.27	7.43	6.79	4.31	5.40	10.66	9.65	12.14	10.93	6.36	1.06	4.70
ΔG_{ET} / eV	-0.87	-0.77	-0.67	-0.41	-0.35	-0.26	-0.64	-0.73	-1.12	-0.58	-0.49	-0.55
ΔG_{rET} /eV	-1.31	-1.41	-1.51	-1.77	-1.83	-1.92	-1.54	-1.45	-1.06	-1.60	-1.69	-1.63
	**$[Cr(dqp)_2][PF_6]_3$**: $^*[Cr(dqp)_2]^{3+}$/$[Cr(dqp)_2]^{2+}$: 1.26 V vs SCE; $[Cr(dqp)_2]^{3+}$/$[Cr(dqp)_2]^{2+}$: -0.40 V vs SCE[6]											
Φ_{CE} / %	13 ± 1	14 ± 1	19± 2	16 ± 1	16 ± 1	18 ± 1	7 ± 1	8 ± 1	8 ± 1	<7	11 ± 3	11 ± 2
k_q/10^9 M^{-1} s^{-1}	6.71	3.82	3.64	1.68	2.13	7.46	7.92	9.81	11.15	3.48	0.19	0.67
ΔG_{ET} / eV	-0.68	-0.58	-0.48	-0.22	-0.16	-0.07	-0.45	-0.54	-0.93	-0.39	-0.30	-0.36
ΔG_{rET} /eV	-0.98	-1.08	-1.18	-1.44	-1.50	-1.59	-1.21	-1.12	-0.73	-1.27	-1.36	-1.30

Cage escape yields can be affected by many different factors, including driving forces and reorganization energy associated with the electron transfer step,[42,43] spin states and heavy atom effects,[14,44-48] solvent polarity and viscosity,[14,48-51] size effects,[52] ionic strength and ion-pairing effects,[44,50,51,53] electrostatic interactions between the radical species,[54] and temperature.[50,51,55] These factors and their influences on cage escape are summarized in Supplementary Table 2. For all electron donors explored here, the Φ_{CE} values obtained with $[Ru(bpz)_3]^{2+}$ are substantially higher than those obtained with $[Cr(dqp)_2]^{3+}$. The Φ_{CE} values furthermore vary between different electron donors for any given complex, but these differences are smaller than those between $[Ru(bpz)_3]^{2+}$ and $[Cr(dqp)_2]^{3+}$ (Supplementary Table 1). As all the Φ_{CE} values were determined at 293 K in aerated acetonitrile without any additives, effects of solvent, temperature, and ion-pairing cannot account for the experimentally observable differences between the $[Ru(bpz)_3]^{2+}$ and $[Cr(dqp)_2]^{3+}$ complexes. We therefore discuss the following potentially relevant factors that could affect the cage escape quantum yields in the systems investigated here:

(1) The driving forces for photoinduced electron transfer (ΔG_{ET}) from the donor to the photoexcited metal complex and for (thermal) reverse electron transfer (ΔG_{rET}) from the reduced metal complex to the oxidized donor (subsection 7.13.1);

(2) The intrinsic difference between the electronic structures of $[Ru(bpz)_3]^{2+}$ and $[Cr(dqp)_2]^{3+}$and their one-electron reduced forms, and the relevance of the spin selection rule (subsection 7.13.2);

(3) Heavy atom effects (subsection 7.13.3);

(4) Size effect of the donors and solvent viscosity (subsection 7.13.4);

(5) The ionic strength (subsection 7.13.5);

(6) Electrostatic effects between the caged radical species (subsection 7.13.6).

Supplemtary Table 2: Summary of factors affecting cage escape quantum yields and the relevant discussion subsections.

Factors	Influences on Φ_{CE}	Exemplary references	Subsections
Driving forces	U-shaped dependence of Φ_{CE} on driving forces found in some selected cases, but not in others.	42	7.13.1
Reorganization energy of the electron donor / acceptor	Small reorganization energy can favor reverse ET and low Φ_{CE}	43	
Spin states and heavy atom effects	Heavy atoms effects enhance spin-orbit coupling and can lower Φ_{CE}	14,44-48	7.13.2, 7.13.3
Solvent polarity	Low polarity often leads to high Φ_{CE}	14,50,51	-
Solvent viscosity	Low viscosity can lead to high Φ_{CE}	48,49	7.13.4
Size effects	Larger molecule sizes tend to give higher Φ_{CE}	52	
Ionic strength	Low ionic strength can lead to high Φ_{CE}	44,50,51,53	7.13.5
Electrostatic interactions between the radical species	Strong electrostatic repulsion can lead to high Φ_{CE}	54	7.13.6
Electrolytes (ion pairing effects)	Electrolyte additives lower Φ_{CE}	53	-
Temperature	Higher temperature can lead to higher Φ_{CE}	50,51,55	-

7.13.1 Driving-force effects

All the employed electron donors have oxidation potentials below 1.2 V vs SCE (Supporting Information Section 3, Supplementary Fig. 1-S5, Supplementary Table 1). For any given electron donor, the driving force for photoinduced electron transfer (ΔG_{ET}, **eq. 2**) is higher by 0.19 eV with $[Ru(bpz)_3]^{2+}$ in comparison to $[Cr(dqp)_2]^{3+}$. The reason for this is that the excited-state reduction potential of the Ru^{II} complex is higher by 0.19 V vs SCE relative to the Cr^{III} complex ($*[Ru(bpz)_3]^{2+}$ / $[Ru(bpz)_3]^{+}$: 1.45 V vs SCE, $*[Cr(dqp)_2]^{3+}$ / $[Cr(dqp)_2]^{2+}$: 1.26 V vs SCE, Supplementary Table 1).[6] For these exergonic reactions, variation of the electron donors leads to the same k_q vs. ΔG_{ET} dependence for both $[Ru(bpz)_3]^{2+}$ and $[Cr(dqp)_2]^{3+}$ following the Rehm-Weller trend (Figure 2c in the main paper).[6,56,57] Recently, we already

investigated the driving-force dependence for the same two complexes over an even greater range of ΔG_{ET} (using additional, weaker electron donors), which revealed a clear Rehm-Weller trend and the same k_q vs. ΔG_{ET} dependence with $[Ru(bpz)_3]^{2+}$ and $[Cr(dqp)_2]^{3+}$.[6] For a given complex, there is no correlation between k_q and Φ_{CE} values. For the $[Cr(dqp)_2]^{3+}$ / DMA-based donor couples, higher k_q values but lower Φ_{CE} values were observed in comparison to $[Cr(dqp)_2]^{3+}$ / TAA-based donor couples. For some electron donors, e.g. DMA-OMe, similar k_q values were obtained with both $[Ru(bpz)_3]^{2+}$ and $[Cr(dqp)_2]^{3+}$, but the Φ_{CE} value for $[Ru(bpz)_3]^{2+}$ exceeds that for $[Cr(dqp)_2]^{3+}$ nine-fold. These findings suggest that the driving force and the reorganization energy for photoinduced electron transfer, which determine the rate constant k_q, are not principally responsible for the differences in cage-escape quantum yields between these two complexes.

Thermal reverse electron transfer from the reduced acceptor to the oxidized donor between the caged photoproducts is generally associated with a larger driving force (ΔG_{rET}, **eq. 3**) for $[Ru(bpz)_3]^{2+}$ compared to $[Cr(dqp)_2]^{3+}$. This is because the reduction potential is -0.33 V vs SCE lower for the ruthenium complex relative to the chromium complex ($[Ru(bpz)_3]^{2+}$ / $[Ru(bpz)_3]^{+}$: -0.73 V vs SCE, $[Cr(dqp)_2]^{3+}$ / $[Cr(dqp)_2]^{2+}$: -0.40 V vs SCE, Supplementary Table 1). No clear correlation was found between the obtained cage escape quantum yields and the driving forces ΔG_{ET} or ΔG_{rET}. These observations are in agreement with previous studies of reductive excited-state quenching of $[Ru(bpy)_3]^{2+}$ by amine-based donors.[58]

$$\Delta G_{ET} = e \times [E^0(D^{\bullet+}/D) - E^0(^*A/A^{\bullet-})] \qquad \textbf{eq. 2}$$

$$\Delta G_{rET} = e \times [E^0(A/A^{\bullet-}) - E^0(D^{\bullet+}/D)] \qquad \textbf{eq. 3}$$

In equations S2 and S3, ΔG_{ET} is the driving force for photoinduced electron transfer from the organic donor (D) to the excited metal complex (*A). ΔG_{rET} stands for the driving force for thermal reverse electron transfer from the one-electron reduced forms of $[Ru(bpz)_3]^{2+}$ and $[Cr(dqp)_2]^{3+}$ ($A^{\bullet-}$) back to the one-electron oxidized form of the organic donor ($D^{\bullet+}$). $E^0(D^{\bullet+}/D)$ is the oxidation potential of the electron donor, while $E^0(^*A/A^{\bullet-})$ and $E^0(A/A^{\bullet-})$ stand for the reduction potentials of the acceptors in their photoactive excited state and their electronic ground-state, respectively. The pre-factor e is the elementary charge.

The analysis above reveals that for any given electron donor, ΔG_{rET} is 0.33 eV more negative for the Ru^{II} complex in comparison to the Cr^{III} complex. In principle, it therefore seems possible that thermal reverse electron transfer between caged radical pairs is generally deeper in the Marcus inverted region with $[Ru(bpz)_3]^{2+}$ than with $[Cr(dqp)_2]^{3+}$. Assuming that the inverted driving-force effect would indeed manifest in a caged radical pair, one might therefore expect that the systematic cage escape differences between $[Ru(bpz)_3]^{2+}$ and $[Cr(dqp)_2]^{3+}$ have their physical origin in different in-cage reverse electron transfer rates (k_{rET}). Based on semi-classical Marcus theory, the ratio of rates for in-cage reverse electron transfer can be expressed as given in **eq. 4**, where H_{AB} is the electronic coupling matrix element between the oxidized electron donor and the reduced metal complex, and λ is the reorganization energy. Both of these parameters are unknown, but reorganization energies in the range of 0.7 – 1.6 eV seem plausible and are considered in the following. For H_{AB}, ratios between $[Ru(bpz)_3]^{2+}$ and $[Cr(dqp)_2]^{3+}$ ($H_{AB}(Ru)$ / $H_{AB}(Cr)$) in the range from 0.3 to 1.6 are considered in the forthcoming analysis.

$$\frac{k_{\mathrm{rET.calc.}}(\mathrm{Ru})}{k_{\mathrm{rET,calc.}}(\mathrm{Cr})} = \frac{[H_{\mathrm{AB}}(\mathrm{Ru})]^2 \cdot \exp\left(-\frac{(\Delta G_{\mathrm{rET}}(\mathrm{Ru})+\lambda)^2}{4\lambda k_{\mathrm{B}}\mathrm{T}}\right)}{[H_{\mathrm{AB}}(\mathrm{Cr})]^2 \cdot \exp\left(-\frac{(\Delta G_{\mathrm{rET}}(\mathrm{Cr})+\lambda)^2}{4\lambda k_{\mathrm{B}}\mathrm{T}}\right)}$$ **eq. 4**

The cage escape quantum yield (Φ_{CE}) for both complexes is governed by the competition between the cage escape rate (k_{CE}) and in-cage reverse electron transfer (k_{rET}), as shown in **eq. 5** and **eq. 6**.

$$\phi_{\mathrm{CE}}(\mathrm{Ru}) = \frac{k_{\mathrm{CE}}(\mathrm{Ru})}{k_{\mathrm{CE}}(\mathrm{Ru})+k_{\mathrm{rET}}(\mathrm{Ru})}$$ **eq. 5**

$$\phi_{\mathrm{CE}}(\mathrm{Cr}) = \frac{k_{\mathrm{CE}}(\mathrm{Cr})}{k_{\mathrm{CE}}(\mathrm{Cr})+k_{\mathrm{rET}}(\mathrm{Cr})}$$ **eq. 6**

Assuming equal cage escape rates for the Ru and the Cr complex (k_{CE}(Ru) = k_{CE}(Cr)), the ratio of rates for in-cage reverse electron transfer can be formulated as **eq. 7**.

$$\frac{k_{\mathrm{rET,exp.}}(\mathrm{Ru})}{k_{\mathrm{rET,exp.}}(\mathrm{Cr})} = \frac{\frac{1}{\phi_{\mathrm{CE}}(\mathrm{Ru})}-1}{\frac{1}{\phi_{\mathrm{CE}}(\mathrm{Cr})}-1}$$ **eq. 7**

When the H_{AB}(Ru) / H_{AB}(Cr) ratios are allowed to vary between 0.3 to 1.6 in 0.1 increments, the corresponding reorganization energies λ can be calculated based on **eq. 4** and **eq. 7** using the experimentally determined cage escape quantum yields Φ_{CE}(Ru) and Φ_{CE}(Cr). This analysis allows to screen what reorganization energies can account for the experimentally observed differences in the cage escape quantum yields for the Ru^{II} and Cr^{III} complexes at these given H_{AB} ratios. The obtained λ values are summarized in Supplementary Table 3 and displayed in Supplementary Fig. 83a as a function of the H_{AB} ratio. We find that for any given H_{AB}(Ru) / H_{AB}(Cr) ratio between 0.3 and 1.6, a sizeable fraction (79-86%) of all calculated λ values fall within a range of ±15% relative to the median reorganization energy obtained for a given donor (red marked values in Supplementary Table 3). In the [H_{AB}(Ru) / H_{AB}(Cr)] / λ matrix spanned by Supplementary Table 3, 135 out of 168 data points fulfill this criterion, which shows that a sizeable fraction of the considered combinations can account for the difference in cage escape quantum yields between Ru^{II} and Cr^{III} for each electron donor.

Supplementary Table 3: Summary of the calculated reorganization energy λ values using eq. 4 with given H_{AB}(Ru) / H_{AB}(Cr) ratios (0.3 to 1.6) and experimentally determined k_{rET}(Ru) / k_{rET}(Cr) values derived from eq. 7. Values highlighted in red deviate by 15% or less from the median reorganization energy for any given donor.

**H_{AB}(Ru) / H_{AB}(Cr)**	0.3	0.4	0.5	0.6	0.7	0.8	0.9	1	1.1	1.2	1.3	1.4	1.5	1.6	
Electron donor	**Reorganization energy *λ* / eV**														**Median *λ* / eV**
**TAA-OMe**	1.18	1.08	1.01	0.97	0.93	0.90	0.88	0.85	0.84	0.82	0.81	0.79	0.78	0.77	0.86
**TAA-PEG3**	1.26	1.16	1.09	1.04	1.00	0.97	0.94	0.92	0.90	0.88	0.87	0.86	0.84	0.83	0.93
**TAA-PEG7**	1.28	1.18	1.12	1.07	1.03	1.00	0.97	0.95	0.93	0.91	0.90	0.89	0.87	0.86	0.96
**TAA-Cl**	1.64	1.50	1.41	1.35	1.30	1.25	1.22	1.19	1.17	1.14	1.12	1.11	1.09	1.08	1.20
**TAA-Br**	1.42	1.32	1.25	1.20	1.16	1.13	1.10	1.08	1.06	1.04	1.02	1.01	1.00	0.99	1.09
**TAA-I**	1.90	1.73	1.62	1.54	1.48	1.43	1.39	1.36	1.33	1.30	1.28	1.26	1.24	1.22	1.37
**DMA**	1.17	1.08	1.03	0.99	0.96	0.93	0.91	0.89	0.87	0.86	0.84	0.83	0.82	0.81	0.90
**DMT**	1.01	0.95	0.90	0.87	0.84	0.82	0.80	0.78	0.77	0.76	0.75	0.74	0.73	0.72	0.79
**DMA-OMe**	0.79	0.74	0.70	0.67	0.65	0.63	0.61	0.60	0.59	0.58	0.57	0.56	0.55	0.55	0.60
**THIQ**	1.54	1.41	1.32	1.25	1.20	1.16	1.13	1.10	1.08	1.06	1.04	1.02	1.01	0.99	1.12
**TEA**	1.75	1.59	1.49	1.41	1.35	1.30	1.26	1.23	1.20	1.18	1.16	1.14	1.12	1.10	1.25
**DIPEA**	1.60	1.46	1.37	1.30	1.25	1.21	1.17	1.14	1.12	1.09	1.07	1.06	1.04	1.03	1.16
**Median *λ* / eV**	1.35	1.25	1.18	1.13	1.09	1.06	1.04	1.01	0.99	0.98	0.96	0.95	0.94	0.92	

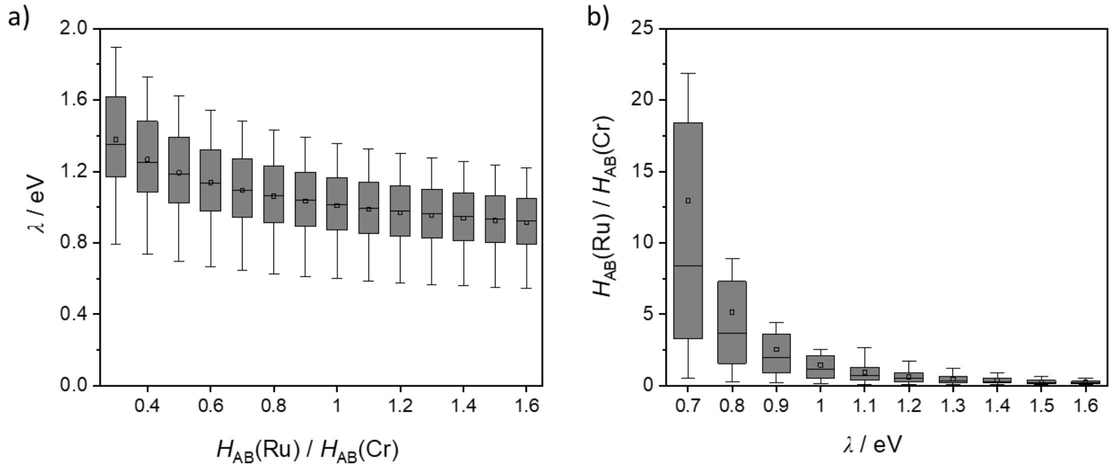

Supplementary Fig. 83*:* Screening of electron transfer parameters to account for the experimentally observed differences between the cage escape quantum yields of $[Ru(bpz)_3]^{2+}$ and $[Cr(dqp)_2]^{3+}$, as determined by equations S4 – S7. a) Reorganization energy values at given ratios of electronic couplings for Ru^{II} and Cr^{III} complexes able to produce $k_{rET,calc.}$(Ru) / $k_{rET,calc.}$(Cr) ratios (eq. 7) in line with the experimental ratios $k_{rET,calc.}$(Ru) / $k_{rET,calc.}$(Cr) derived from eq. 4 (Supplementary Table 3); b) Electronic coupling ratios at a given reorganization energy value able to produce $k_{rET,calc.}$(Ru) / $k_{rET,calc.}$(Cr) ratios (eq. 7) in line with the experimental ratios $k_{rET,calc.}$(Ru) / $k_{rET,calc.}$(Cr) derived from eq. 4. The data size n for each distribution is 12 in both a) and b) (for twelve applied donors). The grey boxes contain the middle 50% of the data, the so-called interquartile range (IQR). The whisker boundaries are drawn at at the most extreme data point that is no more than 1.5 times IQR from the edge of the box. The horizontal black lines within the grey boxes mark the median values, and the open squares within the grey boxes represent the average values.

Analogous calculations can be performed by solving for the H_{AB}(Ru) / H_{AB}(Cr) ratio by considering a range of λ values between 0.7 and 1.6 eV in 0.1 eV increments (Supplementary Fig. 83b). This analysis provides insight into what H_{AB}(Ru) / H_{AB}(Cr) ratios can account for the experimentally observed differences in the cage escape quantum yield values for the Ru^{II} and the Cr^{III} complexes at a given reorganization energy. Broader screening of suitable combinations of H_{AB}(Ru) / H_{AB}(Cr) ratios and λ values that reproduce the experimentally derived $k_{rET,calc.}$(Ru) / $k_{rET,calc.}$(Cr) ratios was subsequently performed. Specifically, all 2184 possibilities emerging from the combination of H_{AB}(Ru) / H_{AB}(Cr) ratios between 0.3 and 1.6 (in 0.1 increments) and λ values ranging from 0.4 to 1.6 eV (in 0.1 eV increments) were considered. Each combination yields a $k_{rET,calc.}$(Ru) / $k_{rET,calc}$(Cr) value according to **eq. 4**, and these values are compared with the experimentally determined $k_{rET,exp.}$(Ru) / $k_{rET,exp.}$(Cr) values derived from the experimentally obtained Φ_{CE} values for both complexes (Supplementary Fig. 84, grey squares). Among these 2184 $k_{rET,calc.}$(Ru) / $k_{rET,calc}$(Cr) values, 63 are able to reproduce the experimental results within a deviation of ±15% (Supplementary Fig. 84, black circles). The calculated $k_{rET,calc}$(Ru) / $k_{rET,calc}$(Cr) ratios and the corresponding H_{AB} ratios as well as the reorganization energies of these 63 combinations are listed in Supplementary Table 4. The full set of all 2184 combinations is displayed in Supplementary Table 5.

Supplementary Table 4: Summary of the $k_{rET,calc.}$(Ru) / $k_{rET,calc}$(Cr) ratios matching the experimental $k_{rET,exp.}$(Ru) / $k_{rET,exp}$(Cr) ratios with 15% deviation or less. 63 (out of 2184) combinations of H_{AB}(Ru) / H_{AB}(Cr) ratios (0.3 to 1.6, in 0.1 increments) and reorganization energies λ (0.4 to 1.6 eV, in 0.1 eV increments) fulfill this condition. The $k_{rET,calc.}$(Ru) / $k_{rET,calc}$(Cr) ratios were obtained using eq. 4, and the $k_{rET,exp.}$(Ru) / $k_{rET,exp}$(Cr) ratios were obtained from eq. 7 and the experimentally determined cage escape quantum yields. The complete set of all 2184 combinations is given in Supplemetary Table 5.

	**$k_{rET,calc.}$(Ru) / $k_{rET,calc.}$(Cr)**														**$k_{rET,exp.}$(Ru) / $k_{rET,exp.}$(Cr)**
**TAA-OMe**	0.121	0.122	0.097			0.108					0.101	0.117			0.108
H_{AB}(Ru) / H_{AB}(Cr) ; λ / eV	0.3; 1.2	0.4; 1.1	0.5; 1.0			0.8; 0.9					1.3; 0.8	1.4; 0.8			
**TAA-PEG$_3$**			0.106		0.099				0.099						0.100
H_{AB}(Ru) / H_{AB}(Cr) ; λ / eV			0.5; 1.1		0.7; 1.0				1.1; 0.9						
**TAA-PEG$_7$**	0.072	0.073	0.058			0.067				0.057	0.067				0.066
H_{AB}(Ru) / H_{AB}(Cr) ; λ / eV	0.3; 1.3	0.4; 1.2	0.5: 1.1			0.8; 1.0				1.2; 0.9	1.3; 0.9				
**TAA-Cl**	0.088	0.101	0.096		0.106		0.089	0.110				0.098	0.112		0.103
H_{AB}(Ru) / H_{AB}(Cr) ; λ / eV	0.3; 1.6	0.4; 1.5	0.5; 1.4		0.7; 1.3		0.9; 1.2	1.0; 1.2				1.4; 1.1	1.5; 1.1		
**TAA-Br**	0.026	0.026		0.029			0.028					0.025	0.029		0.028
H_{AB}(Ru) / H_{AB}(Cr) ; λ / eV	0.3; 1.4	0.4; 1.3		0.6; 1.2			0.9; 1.1					1.4; 1.0	1.5; 1.0		
**TAA-I**			0.132		0.161		0.154			0.146				0.125	0.146
H_{AB}(Ru) / H_{AB}(Cr) ; λ / eV			0.5; 1.6		0.7; 1.5		0.9; 1.4			1.2; 1.3				1.6; 1.2	
**DMA**		0.031		0.031			0.026	0.032							0.028
H_{AB}(Ru) / H_{AB}(Cr) ; λ / eV		0.4; 1.1		0.6; 1.0			0.9; 0.9	1.0; 0.9							
**DMT**	0.014		0.015				0.015								0.015
H_{AB}(Ru) / H_{AB}(Cr) ; λ / eV	0.3; 1.0		0.5; 1.0				0.9; 0.8								
**DMA-OMe**	0.041		0.040					0.040							0.039
H_{AB}(Ru) / H_{AB}(Cr) ; λ / eV	0.3; 0.8		0.5; 0.7					1.0; 0.6							
**THIQ**	0.119	0.136	0.127		0.136			0.137					0.131	0.149	0.140
H_{AB}(Ru) / H_{AB}(Cr) ; λ / eV	0.3; 1.5	0.4; 1.4	0.5; 1.3		0.7; 1.2			1.0; 1.1					1.5; 1.0	1.6; 1.0	
**TEA**		0.217	0.224	0.201		0.207			0.206				0.180	0.205	0.210
H_{AB}(Ru) / H_{AB}(Cr) ; λ / eV		0.4; 1.6	0.5; 1.5	0.6; 1.4		0.8; 1.3			1.1; 1.2				1.5; 1.1	1.6; 1.1	
**DIPEA**	0.156			0.157		0.151			0.138	0.165					0.157
H_{AB}(Ru) / H_{AB}(Cr) ; λ / eV	0.3; 1.6			0.6; 1.3		0.8; 1.2			1.1; 1.1	1.2; 1.1					

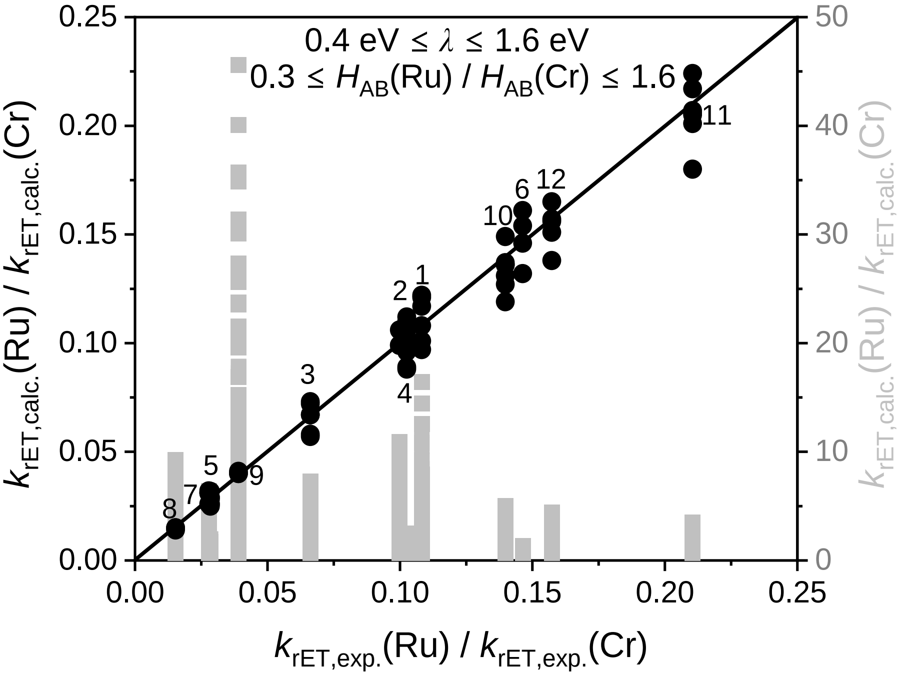

Supplementary Fig. 84: Correlation between calculated and experimental ratios of rate constants for reverse in-cage electron transfer. Reorganization energies (λ) ranging from 0.4 to 1.6 eV (in 0.1 eV increments) were considered, along with electronic coupling ratios H_{AB}(Ru) / H_{AB}(Cr) ranging from 0.3 to 1.6 (in 0.1 increments). The black circles correspond to the 63 combinations leading to good match (with 15% deviation or less) between $k_{rET,calc.}$(Ru) / $k_{rET,calc.}$(Cr) and $k_{rET,calc.}$(Ru) / $k_{rET,calc.}$(Cr) ratios as listed in Supplementary Table 4; these data points are plotted against the left horizontal axis. The grey squares, plotted against the right y-axis, correspond to all considered combinations of H_{AB}(Ru) / H_{AB}(Cr) and λ values, as listed in Supplementary Table 5 (2184 combinations in total – 63 combinations providing a match within 15% deviation or less).

Supplementary Table 5: Summary of the $k_{rET,calc.}$(Ru) / $k_{rET,calc}$(Cr) values using eq. 4 with given reorganization energies λ varying from 0.4 eV to 1.6 eV (in 0.1 eV increments) and the H_{AB}(Ru) / H_{AB}(Cr) ratio varying from 0.3 to 1.6 (in 0.1 increments), giving a total of 2184 combinations.

H_{AB}(Ru) / H_{AB}(Cr)		0.3	0.3	0.3	0.3	0.3	0.3		0.3	0.3	0.3	0.3	0.3	0.3	0.3
λ / eV		0.4	0.5	0.6	0.7	0.8	0.9		1	1.1	1.2	1.3	1.4	1.5	1.6
Donors	k_{rET}, exp. (Ru) / k_{rET}, exp.(Cr)		**k_{rET}, calc. (Ru) / k_{rET}, calc.(Cr)**												
**TAA-OMe**	0.108	0.000	0.000	0.000	0.001	0.005	0.015		0.035	0.069	0.121	0.196	0.296	0.423	0.577
**TAA-PEG$_3$**	0.100	0.000	0.000	0.000	0.001	0.002	0.007		0.018	0.038	0.070	0.119	0.186	0.273	0.384
**TAA-PEG$_7$**	0.066	0.000	0.000	0.000	0.000	0.001	0.004		0.009	0.021	0.041	0.072	0.116	0.177	0.255
**TAA-Cl**	0.103	0.000	0.000	0.000	0.000	0.000	0.001		0.002	0.004	0.010	0.019	0.035	0.057	0.088
**TAA-Br**	0.028	0.000	0.000	0.000	0.000	0.000	0.000		0.001	0.003	0.007	0.014	0.026	0.044	0.069
**TAA-I**	0.146	0.000	0.000	0.000	0.000	0.000	0.000		0.001	0.002	0.004	0.009	0.017	0.030	0.048
**DMA**	0.028	0.000	0.000	0.000	0.000	0.001	0.003		0.008	0.018	0.035	0.062	0.101	0.155	0.226
**DMT**	0.015	0.000	0.000	0.000	0.000	0.002	0.005		0.014	0.030	0.057	0.097	0.154	0.230	0.326
**DMA-OMe**	0.039	0.000	0.001	0.004	0.015	0.041	0.093		0.179	0.304	0.474	0.689	0.951	1.256	1.602
**THIQ**	0.140	0.000	0.000	0.000	0.000	0.001	0.002		0.005	0.012	0.025	0.046	0.076	0.119	0.177
**TEA**	0.210	0.000	0.000	0.000	0.000	0.000	0.001		0.003	0.007	0.015	0.029	0.050	0.081	0.122
**DIPEA**	0.157	0.000	0.000	0.000	0.000	0.000	0.001		0.004	0.010	0.021	0.039	0.066	0.105	0.156

H_{AB}(Ru) / H_{AB}(Cr)		0.4	0.4	0.4	0.4	0.4	0.4		0.4	0.4	0.4	0.4	0.4	0.4	0.4
λ / eV		0.4	0.5	0.6	0.7	0.8	0.9		1	1.1	1.2	1.3	1.4	1.5	1.6
	k_{rET}, exp. (Ru) / k_{rET}, exp.(Cr)		**k_{rET}, calc. (Ru) / k_{rET}, calc.(Cr)**												
**TAA-OMe**	0.108	0.000	0.000	0.000	0.003	0.010	0.027		0.062	0.122	0.216	0.349	0.526	0.751	1.026
**TAA-PEG$_3$**	0.100	0.000	0.000	0.000	0.001	0.004	0.013		0.032	0.068	0.125	0.211	0.330	0.486	0.682
**TAA-PEG$_7$**	0.066	0.000	0.000	0.000	0.000	0.002	0.006		0.017	0.037	0.073	0.128	0.207	0.314	0.453
**TAA-Cl**	0.103	0.000	0.000	0.000	0.000	0.000	0.001		0.003	0.008	0.018	0.035	0.061	0.101	0.157
**TAA-Br**	0.028	0.000	0.000	0.000	0.000	0.000	0.001		0.002	0.006	0.013	0.026	0.046	0.078	0.123
**TAA-I**	0.146	0.000	0.000	0.000	0.000	0.000	0.000		0.001	0.003	0.008	0.016	0.031	0.053	0.085
**DMA**	0.028	0.000	0.000	0.000	0.000	0.001	0.005		0.014	0.031	0.062	0.110	0.180	0.276	0.401
**DMT**	0.015	0.000	0.000	0.000	0.001	0.003	0.010		0.025	0.053	0.101	0.173	0.274	0.408	0.579
**DMA-OMe**	0.039	0.000	0.001	0.006	0.026	0.074	0.166		0.318	0.541	0.842	1.225	1.690	2.233	2.849
**THIQ**	0.140	0.000	0.000	0.000	0.000	0.001	0.003		0.009	0.022	0.044	0.081	0.136	0.212	0.314
**TEA**	0.210	0.000	0.000	0.000	0.000	0.000	0.002		0.005	0.013	0.027	0.052	0.089	0.143	0.217
**DIPEA**	0.157	0.000	0.000	0.000	0.000	0.001	0.003		0.008	0.018	0.038	0.070	0.118	0.186	0.278
H_{AB}(Ru) / H_{AB}(Cr)		0.5	0.5	0.5	0.5	0.5	0.5		0.5	0.5	0.5	0.5	0.5	0.5	0.5
λ / eV		0.4	0.5	0.6	0.7	0.8	0.9		1	1.1	1.2	1.3	1.4	1.5	1.6
	k_{rET}, exp. (Ru) / k_{rET}, exp.(Cr)		**k_{rET}, calc. (Ru) / k_{rET}, calc.(Cr)**												
**TAA-OMe**	0.108	0.000	0.000	0.001	0.004	0.015	0.042		0.097	0.191	0.337	0.545	0.822	1.174	1.603
**TAA-PEG$_3$**	0.100	0.000	0.000	0.000	0.002	0.007	0.020		0.050	0.106	0.196	0.330	0.515	0.759	1.066
**TAA-PEG$_7$**	0.066	0.000	0.000	0.000	0.001	0.003	0.010		0.026	0.058	0.114	0.199	0.323	0.491	0.708
**TAA-Cl**	0.103	0.000	0.000	0.000	0.000	0.000	0.001		0.005	0.012	0.028	0.054	0.096	0.158	0.245
**TAA-Br**	0.028	0.000	0.000	0.000	0.000	0.000	0.001		0.003	0.009	0.020	0.040	0.073	0.122	0.192
**TAA-I**	0.146	0.000	0.000	0.000	0.000	0.000	0.001		0.002	0.005	0.012	0.025	0.048	0.082	0.133
**DMA**	0.028	0.000	0.000	0.000	0.000	0.002	0.008		0.022	0.049	0.096	0.171	0.281	0.431	0.627
**DMT**	0.015	0.000	0.000	0.000	0.001	0.005	0.015		0.039	0.083	0.157	0.270	0.428	0.638	0.905
**DMA-OMe**	0.039	0.000	0.001	0.010	0.040	0.115	0.259		0.497	0.845	1.316	1.915	2.640	3.489	4.451
**THIQ**	0.140	0.000	0.000	0.000	0.000	0.001	0.005		0.015	0.034	0.070	0.127	0.212	0.332	0.490
**TEA**	0.210	0.000	0.000	0.000	0.000	0.001	0.003		0.008	0.020	0.043	0.081	0.139	0.224	0.340
**DIPEA**	0.157	0.000	0.000	0.000	0.000	0.001	0.004		0.012	0.029	0.059	0.109	0.185	0.291	0.434
H_{AB}(Ru) / H_{AB}(Cr)		0.6	0.6	0.6	0.6	0.6	0.6		0.6	0.6	0.6	0.6	0.6	0.6	0.6
λ / eV		0.4	0.5	0.6	0.7	0.8	0.9		1	1.1	1.2	1.3	1.4	1.5	1.6
	k_{rET}, exp. (Ru) / k_{rET}, exp.(Cr)		**k_{rET}, calc. (Ru) / k_{rET}, calc.(Cr)**												
**TAA-OMe**	0.108	0.000	0.000	0.001	0.006	0.021	0.061		0.140	0.276	0.486	0.785	1.184	1.690	2.309
**TAA-PEG$_3$**	0.100	0.000	0.000	0.000	0.002	0.009	0.029		0.073	0.152	0.282	0.475	0.742	1.093	1.535
**TAA-PEG$_7$**	0.066	0.000	0.000	0.000	0.001	0.004	0.014		0.038	0.084	0.163	0.287	0.465	0.707	1.020

**TAA-Cl**	0.103	0.000	0.000	0.000	0.000	0.001	0.002		0.007	0.018	0.040	0.078	0.138	0.228	0.353
**TAA-Br**	0.028	0.000	0.000	0.000	0.000	0.000	0.001		0.005	0.013	0.029	0.057	0.104	0.175	0.276
**TAA-I**	0.146	0.000	0.000	0.000	0.000	0.000	0.001		0.003	0.007	0.018	0.037	0.069	0.119	0.191
**DMA**	0.028	0.000	0.000	0.000	0.001	0.003	0.011		0.031	0.070	0.139	0.247	0.405	0.621	0.902
**DMT**	0.015	0.000	0.000	0.000	0.002	0.007	0.022		0.056	0.120	0.227	0.388	0.616	0.919	1.303
**DMA-OMe**	0.039	0.000	0.002	0.014	0.058	0.166	0.373		0.715	1.217	1.895	2.757	3.802	5.023	6.410
**THIQ**	0.140	0.000	0.000	0.000	0.000	0.002	0.007		0.021	0.049	0.100	0.183	0.306	0.478	0.706
**TEA**	0.210	0.000	0.000	0.000	0.000	0.001	0.004		0.012	0.029	0.061	0.116	0.201	0.323	0.489
**DIPEA**	0.157	0.000	0.000	0.000	0.000	0.002	0.006		0.017	0.041	0.085	0.157	0.266	0.419	0.625
H_{AB}(Ru) / H_{AB}(Cr)		0.7	0.7	0.7	0.7	0.7	0.7		0.7	0.7	0.7	0.7	0.7	0.7	0.7
λ / eV		0.4	0.5	0.6	0.7	0.8	0.9		1	1.1	1.2	1.3	1.4	1.5	1.6
	k_{rET}, exp. (Ru) / k_{rET}, exp.(Cr)		**k_{rET}, calc. (Ru) / k_{rET}, calc.(Cr)**												
**TAA-OMe**	0.108	0.000	0.000	0.001	0.008	0.029	0.083		0.190	0.375	0.661	1.068	1.611	2.301	3.143
**TAA-PEG$_3$**	0.100	0.000	0.000	0.000	0.003	0.013	0.040		0.099	0.207	0.384	0.646	1.010	1.488	2.089
**TAA-PEG$_7$**	0.066	0.000	0.000	0.000	0.001	0.006	0.019		0.051	0.114	0.222	0.391	0.633	0.963	1.388
**TAA-Cl**	0.103	0.000	0.000	0.000	0.000	0.001	0.003		0.009	0.024	0.054	0.106	0.188	0.310	0.480
**TAA-Br**	0.028	0.000	0.000	0.000	0.000	0.000	0.002		0.006	0.017	0.039	0.078	0.142	0.239	0.376
**TAA-I**	0.146	0.000	0.000	0.000	0.000	0.000	0.001		0.004	0.010	0.024	0.050	0.093	0.161	0.260
**DMA**	0.028	0.000	0.000	0.000	0.001	0.004	0.016		0.042	0.096	0.189	0.336	0.551	0.845	1.228
**DMT**	0.015	0.000	0.000	0.000	0.002	0.009	0.030		0.076	0.163	0.308	0.528	0.838	1.250	1.774
**DMA-OMe**	0.039	0.000	0.003	0.020	0.079	0.226	0.508		0.973	1.656	2.580	3.753	5.175	6.838	8.724
**THIQ**	0.140	0.000	0.000	0.000	0.001	0.003	0.010		0.029	0.067	0.136	0.249	0.416	0.650	0.961
**TEA**	0.210	0.000	0.000	0.000	0.000	0.001	0.005		0.016	0.039	0.083	0.158	0.273	0.439	0.666
**DIPEA**	0.157	0.000	0.000	0.000	0.000	0.002	0.008		0.023	0.056	0.116	0.214	0.362	0.571	0.850
H_{AB}(Ru) / H_{AB}(Cr)		0.8	0.8	0.8	0.8	0.8	0.8		0.8	0.8	0.8	0.8	0.8	0.8	0.8
λ / eV		0.4	0.5	0.6	0.7	0.8	0.9		1	1.1	1.2	1.3	1.4	1.5	1.6
	k_{rET}, exp. (Ru) / k_{rET}, exp.(Cr)		**k_{rET}, calc. (Ru) / k_{rET}, calc.(Cr)**												
**TAA-OMe**	0.108	0.000	0.000	0.002	0.010	0.038	0.108		0.248	0.490	0.863	1.395	2.104	3.005	4.105
**TAA-PEG$_3$**	0.100	0.000	0.000	0.001	0.004	0.017	0.052		0.129	0.270	0.501	0.844	1.319	1.944	2.728
**TAA-PEG$_7$**	0.066	0.000	0.000	0.000	0.002	0.007	0.025		0.067	0.149	0.291	0.510	0.827	1.257	1.813
**TAA-Cl**	0.103	0.000	0.000	0.000	0.000	0.001	0.004		0.012	0.032	0.071	0.138	0.246	0.405	0.627
**TAA-Br**	0.028	0.000	0.000	0.000	0.000	0.001	0.002		0.008	0.022	0.051	0.102	0.186	0.312	0.491
**TAA-I**	0.146	0.000	0.000	0.000	0.000	0.000	0.001		0.005	0.013	0.031	0.065	0.122	0.211	0.340
**DMA**	0.028	0.000	0.000	0.000	0.001	0.006	0.020		0.055	0.125	0.247	0.439	0.719	1.103	1.604
**DMT**	0.015	0.000	0.000	0.000	0.003	0.012	0.039		0.099	0.213	0.403	0.690	1.095	1.633	2.317
**DMA-OMe**	0.039	0.000	0.004	0.026	0.104	0.295	0.664		1.271	2.163	3.369	4.902	6.760	8.931	11.395
**THIQ**	0.140	0.000	0.000	0.000	0.001	0.004	0.013		0.037	0.087	0.178	0.325	0.544	0.850	1.256

**TEA**	0.210	0.000	0.000	0.000	0.000	0.002	0.007		0.021	0.051	0.109	0.207	0.357	0.574	0.869
**DIPEA**	0.157	0.000	0.000	0.000	0.001	0.003	0.011		0.031	0.073	0.151	0.279	0.473	0.745	1.111
H_{AB}(Ru) / H_{AB}(Cr)		0.9	0.9	0.9	0.9	0.9	0.9		0.9	0.9	0.9	0.9	0.9	0.9	0.9
λ / eV		0.4	0.5	0.6	0.7	0.8	0.9		1	1.1	1.2	1.3	1.4	1.5	1.6
	k_{rET}, exp. (Ru) / k_{rET}, exp.(Cr)		**k_{rET}, calc. (Ru) / k_{rET}, calc.(Cr)**												
**TAA-OMe**	0.108	0.000	0.000	0.002	0.013	0.048	0.137		0.314	0.620	1.093	1.766	2.663	3.803	5.195
**TAA-PEG$_3$**	0.100	0.000	0.000	0.001	0.005	0.021	0.066		0.163	0.342	0.634	1.068	1.670	2.460	3.453
**TAA-PEG$_7$**	0.066	0.000	0.000	0.000	0.002	0.009	0.032		0.085	0.189	0.368	0.646	1.047	1.591	2.295
**TAA-Cl**	0.103	0.000	0.000	0.000	0.000	0.001	0.005		0.016	0.040	0.089	0.175	0.311	0.513	0.794
**TAA-Br**	0.028	0.000	0.000	0.000	0.000	0.001	0.003		0.010	0.028	0.064	0.129	0.235	0.395	0.621
**TAA-I**	0.146	0.000	0.000	0.000	0.000	0.000	0.002		0.006	0.017	0.039	0.082	0.154	0.267	0.430
**DMA**	0.028	0.000	0.000	0.000	0.001	0.007	0.026		0.070	0.158	0.312	0.556	0.910	1.396	2.030
**DMT**	0.015	0.000	0.000	0.000	0.003	0.015	0.049		0.126	0.270	0.510	0.873	1.386	2.067	2.932
**DMA-OMe**	0.039	0.000	0.005	0.033	0.131	0.373	0.840		1.609	2.738	4.264	6.204	8.555	11.303	14.422
**THIQ**	0.140	0.000	0.000	0.000	0.001	0.005	0.017		0.047	0.111	0.225	0.411	0.688	1.075	1.589
**TEA**	0.210	0.000	0.000	0.000	0.000	0.002	0.009		0.026	0.065	0.138	0.261	0.452	0.726	1.100
**DIPEA**	0.157	0.000	0.000	0.000	0.001	0.004	0.013		0.039	0.093	0.191	0.353	0.598	0.943	1.406
H_{AB}(Ru) / H_{AB}(Cr)		1	1	1	1	1	1		1	1	1	1	1	1	1
λ / eV		0.4	0.5	0.6	0.7	0.8	0.9		1	1.1	1.2	1.3	1.4	1.5	1.6
	k_{rET}, exp. (Ru) / k_{rET}, exp.(Cr)		**k_{rET}, calc. (Ru) / k_{rET}, calc.(Cr)**												
**TAA-OMe**	0.108	0.000	0.000	0.003	0.016	0.060	0.169		0.388	0.765	1.349	2.180	3.288	4.696	6.413
**TAA-PEG$_3$**	0.100	0.000	0.000	0.001	0.006	0.026	0.082		0.202	0.423	0.783	1.318	2.062	3.037	4.263
**TAA-PEG$_7$**	0.066	0.000	0.000	0.000	0.002	0.012	0.040		0.105	0.233	0.454	0.798	1.293	1.965	2.833
**TAA-Cl**	0.103	0.000	0.000	0.000	0.000	0.001	0.006		0.019	0.050	0.110	0.216	0.384	0.633	0.980
**TAA-Br**	0.028	0.000	0.000	0.000	0.000	0.001	0.004		0.013	0.035	0.079	0.160	0.290	0.487	0.767
**TAA-I**	0.146	0.000	0.000	0.000	0.000	0.000	0.002		0.007	0.020	0.049	0.102	0.191	0.329	0.531
**DMA**	0.028	0.000	0.000	0.000	0.002	0.009	0.032		0.086	0.195	0.386	0.686	1.124	1.724	2.507
**DMT**	0.015	0.000	0.000	0.001	0.004	0.019	0.061		0.155	0.333	0.629	1.078	1.711	2.551	3.620
**DMA-OMe**	0.039	0.000	0.006	0.040	0.162	0.460	1.037		1.986	3.380	5.264	7.659	10.562	13.954	17.805
**THIQ**	0.140	0.000	0.000	0.000	0.001	0.006	0.021		0.058	0.137	0.278	0.507	0.849	1.327	1.962
**TEA**	0.210	0.000	0.000	0.000	0.000	0.003	0.011		0.032	0.080	0.170	0.323	0.558	0.897	1.358
**DIPEA**	0.157	0.000	0.000	0.000	0.001	0.004	0.017		0.048	0.114	0.236	0.436	0.738	1.165	1.736
H_{AB}(Ru) / H_{AB}(Cr)		1.1	1.1	1.1	1.1	1.1	1.1		1.1	1.1	1.1	1.1	1.1	1.1	1.1
λ / eV		0.4	0.5	0.6	0.7	0.8	0.9		1	1.1	1.2	1.3	1.4	1.5	1.6
	k_{rET}, exp. (Ru) / k_{rET}, exp.(Cr)		**k_{rET}, calc. (Ru) / k_{rET}, calc.(Cr)**												
**TAA-OMe**	0.108	0.000	0.000	0.003	0.019	0.072	0.204		0.469	0.926	1.633	2.637	3.979	5.682	7.760

**TAA-PEG$_3$**	0.100	0.000	0.000	0.001	0.007	0.032	0.099		0.244	0.511	0.947	1.595	2.495	3.675	5.158
**TAA-PEG$_7$**	0.066	0.000	0.000	0.000	0.003	0.014	0.048		0.127	0.282	0.549	0.965	1.564	2.377	3.429
**TAA-Cl**	0.103	0.000	0.000	0.000	0.000	0.002	0.007		0.023	0.060	0.133	0.261	0.465	0.766	1.186
**TAA-Br**	0.028	0.000	0.000	0.000	0.000	0.001	0.005		0.016	0.042	0.096	0.193	0.351	0.590	0.928
**TAA-I**	0.146	0.000	0.000	0.000	0.000	0.000	0.002		0.009	0.025	0.059	0.123	0.231	0.398	0.642
**DMA**	0.028	0.000	0.000	0.000	0.002	0.011	0.038		0.104	0.236	0.467	0.830	1.360	2.086	3.033
**DMT**	0.015	0.000	0.000	0.001	0.005	0.023	0.074		0.188	0.403	0.762	1.305	2.070	3.087	4.381
**DMA-OMe**	0.039	0.000	0.007	0.049	0.196	0.557	1.255		2.403	4.090	6.370	9.268	12.780	16.885	21.544
**THIQ**	0.140	0.000	0.000	0.000	0.001	0.007	0.025		0.071	0.165	0.337	0.614	1.028	1.606	2.374
**TEA**	0.210	0.000	0.000	0.000	0.001	0.003	0.013		0.039	0.097	0.206	0.390	0.675	1.085	1.644
**DIPEA**	0.157	0.000	0.000	0.000	0.001	0.005	0.020		0.058	0.138	0.286	0.528	0.893	1.409	2.100
H_{AB}(Ru) / H_{AB}(Cr)		1.2	1.2	1.2	1.2	1.2	1.2		1.2	1.2	1.2	1.2	1.2	1.2	1.2
λ / eV		0.4	0.5	0.6	0.7	0.8	0.9		1	1.1	1.2	1.3	1.4	1.5	1.6
	k_{rET}, exp. (Ru) / k_{rET}, exp.(Cr)		**k_{rET}, calc. (Ru) / k_{rET}, calc.(Cr)**												
**TAA-OMe**	0.108	0.000	0.000	0.004	0.023	0.086	0.243		0.558	1.102	1.943	3.139	4.735	6.762	9.235
**TAA-PEG$_3$**	0.100	0.000	0.000	0.001	0.009	0.038	0.118		0.290	0.608	1.127	1.899	2.969	4.374	6.139
**TAA-PEG$_7$**	0.066	0.000	0.000	0.000	0.003	0.017	0.057		0.151	0.336	0.654	1.148	1.861	2.829	4.080
**TAA-Cl**	0.103	0.000	0.000	0.000	0.000	0.002	0.009		0.028	0.072	0.159	0.311	0.553	0.911	1.411
**TAA-Br**	0.028	0.000	0.000	0.000	0.000	0.001	0.006		0.019	0.050	0.114	0.230	0.418	0.702	1.104
**TAA-I**	0.146	0.000	0.000	0.000	0.000	0.001	0.003		0.010	0.029	0.070	0.146	0.275	0.474	0.765
**DMA**	0.028	0.000	0.000	0.000	0.003	0.013	0.046		0.124	0.281	0.555	0.988	1.618	2.482	3.610
**DMT**	0.015	0.000	0.000	0.001	0.006	0.027	0.088		0.224	0.480	0.906	1.553	2.463	3.674	5.213
**DMA-OMe**	0.039	0.000	0.008	0.058	0.233	0.663	1.493		2.860	4.867	7.581	11.029	15.209	20.094	25.639
**THIQ**	0.140	0.000	0.000	0.000	0.002	0.008	0.030		0.084	0.197	0.400	0.731	1.223	1.911	2.825
**TEA**	0.210	0.000	0.000	0.000	0.001	0.004	0.015		0.047	0.115	0.245	0.465	0.803	1.291	1.956
**DIPEA**	0.157	0.000	0.000	0.000	0.001	0.006	0.024		0.069	0.165	0.340	0.628	1.063	1.677	2.499
H_{AB}(Ru) / H_{AB}(Cr)		1.3	1.3	1.3	1.3	1.3	1.3		1.3	1.3	1.3	1.3	1.3	1.3	1.3
λ / eV		0.4	0.5	0.6	0.7	0.8	0.9		1	1.1	1.2	1.3	1.4	1.5	1.6
	k_{rET}, exp. (Ru) / k_{rET}, exp.(Cr)		**k_{rET}, calc. (Ru) / k_{rET}, calc.(Cr)**												
**TAA-OMe**	0.108	0.000	0.000	0.004	0.027	0.101	0.285		0.655	1.294	2.280	3.684	5.557	7.935	10.839
**TAA-PEG$_3$**	0.100	0.000	0.000	0.002	0.010	0.045	0.138		0.341	0.714	1.323	2.228	3.484	5.133	7.204
**TAA-PEG$_7$**	0.066	0.000	0.000	0.001	0.004	0.020	0.067		0.177	0.394	0.767	1.348	2.185	3.320	4.789
**TAA-Cl**	0.103	0.000	0.000	0.000	0.000	0.002	0.010		0.032	0.084	0.186	0.365	0.649	1.070	1.656
**TAA-Br**	0.028	0.000	0.000	0.000	0.000	0.001	0.007		0.022	0.059	0.134	0.270	0.491	0.824	1.296
**TAA-I**	0.146	0.000	0.000	0.000	0.000	0.001	0.003		0.012	0.035	0.082	0.172	0.322	0.556	0.897
**DMA**	0.028	0.000	0.000	0.000	0.003	0.015	0.054		0.146	0.330	0.652	1.159	1.899	2.913	4.236

**DMT**	0.015	0.000	0.000	0.001	0.007	0.032	0.103		0.262	0.563	1.064	1.822	2.891	4.312	6.118
**DMA-OMe**	0.039	0.001	0.010	0.068	0.274	0.778	1.752		3.357	5.712	8.897	12.944	17.850	23.582	30.090
**THIQ**	0.140	0.000	0.000	0.000	0.002	0.009	0.035		0.098	0.231	0.470	0.857	1.435	2.243	3.316
**TEA**	0.210	0.000	0.000	0.000	0.001	0.005	0.018		0.055	0.135	0.288	0.545	0.943	1.516	2.296
**DIPEA**	0.157	0.000	0.000	0.000	0.001	0.007	0.028		0.081	0.193	0.399	0.737	1.248	1.968	2.933
H_{AB}(Ru) / H_{AB}(Cr)		1.4	1.4	1.4	1.4	1.4	1.4		1.4	1.4	1.4	1.4	1.4	1.4	1.4
λ / eV		0.4	0.5	0.6	0.7	0.8	0.9		1	1.1	1.2	1.3	1.4	1.5	1.6
	k_{rET}, exp. (Ru) / k_{rET}, exp.(Cr)		**k_{rET}, calc. (Ru) / k_{rET}, calc.(Cr)**												
**TAA-OMe**	0.108	0.000	0.000	0.005	0.031	0.117	0.331		0.760	1.500	2.644	4.272	6.445	9.203	12.570
**TAA-PEG_3**	0.100	0.000	0.000	0.002	0.012	0.052	0.160		0.395	0.828	1.534	2.584	4.041	5.953	8.355
**TAA-PEG_7**	0.066	0.000	0.000	0.001	0.005	0.023	0.077		0.206	0.457	0.890	1.563	2.534	3.851	5.554
**TAA-Cl**	0.103	0.000	0.000	0.000	0.000	0.003	0.012		0.038	0.098	0.216	0.423	0.753	1.240	1.920
**TAA-Br**	0.028	0.000	0.000	0.000	0.000	0.002	0.008		0.025	0.068	0.156	0.313	0.569	0.955	1.503
**TAA-I**	0.146	0.000	0.000	0.000	0.000	0.001	0.004		0.014	0.040	0.095	0.199	0.374	0.645	1.041
**DMA**	0.028	0.000	0.000	0.000	0.004	0.018	0.062		0.169	0.383	0.756	1.344	2.203	3.379	4.913
**DMT**	0.015	0.000	0.000	0.001	0.008	0.037	0.120		0.304	0.653	1.234	2.114	3.353	5.001	7.096
**DMA-OMe**	0.039	0.001	0.011	0.079	0.317	0.902	2.032		3.893	6.625	10.318	15.012	20.701	27.350	34.898
**THIQ**	0.140	0.000	0.000	0.000	0.002	0.011	0.040		0.114	0.268	0.545	0.994	1.665	2.602	3.845
**TEA**	0.210	0.000	0.000	0.000	0.001	0.005	0.021		0.063	0.157	0.334	0.632	1.094	1.758	2.663
**DIPEA**	0.157	0.000	0.000	0.000	0.002	0.009	0.032		0.094	0.224	0.463	0.855	1.447	2.283	3.402
H_{AB}(Ru) / H_{AB}(Cr)		1.5	1.5	1.5	1.5	1.5	1.5		1.5	1.5	1.5	1.5	1.5	1.5	1.5
λ / eV		0.4	0.5	0.6	0.7	0.8	0.9		1	1.1	1.2	1.3	1.4	1.5	1.6
	k_{rET}, exp. (Ru) / k_{rET}, exp.(Cr)		**k_{rET}, calc. (Ru) / k_{rET}, calc.(Cr)**												
**TAA-OMe**	0.108	0.000	0.000	0.006	0.035	0.134	0.380		0.872	1.722	3.036	4.904	7.398	10.565	14.430
**TAA-PEG_3**	0.100	0.000	0.000	0.002	0.014	0.059	0.184		0.454	0.951	1.761	2.967	4.639	6.834	9.592
**TAA-PEG_7**	0.066	0.000	0.000	0.001	0.005	0.026	0.089		0.236	0.525	1.022	1.794	2.909	4.420	6.375
**TAA-Cl**	0.103	0.000	0.000	0.000	0.000	0.003	0.013		0.043	0.112	0.248	0.486	0.864	1.424	2.205
**TAA-Br**	0.028	0.000	0.000	0.000	0.000	0.002	0.009		0.029	0.078	0.179	0.359	0.653	1.096	1.725
**TAA-I**	0.146	0.000	0.000	0.000	0.000	0.001	0.005		0.016	0.046	0.110	0.228	0.429	0.741	1.195
**DMA**	0.028	0.000	0.000	0.000	0.004	0.021	0.071		0.194	0.439	0.868	1.543	2.529	3.879	5.640
**DMT**	0.015	0.000	0.000	0.001	0.010	0.043	0.137		0.349	0.750	1.416	2.426	3.849	5.741	8.146
**DMA-OMe**	0.039	0.001	0.013	0.091	0.364	1.036	2.333		4.469	7.605	11.845	17.233	23.764	31.397	40.061
**THIQ**	0.140	0.000	0.000	0.000	0.002	0.013	0.046		0.131	0.308	0.626	1.141	1.911	2.987	4.414
**TEA**	0.210	0.000	0.000	0.000	0.001	0.006	0.024		0.073	0.180	0.383	0.726	1.255	2.018	3.056
**DIPEA**	0.157	0.000	0.000	0.000	0.002	0.010	0.037		0.108	0.257	0.531	0.982	1.661	2.621	3.905
H_{AB}(Ru) / H_{AB}(Cr)		1.6	1.6	1.6	1.6	1.6	1.6		1.6	1.6	1.6	1.6	1.6	1.6	1.6
λ / eV		0.4	0.5	0.6	0.7	0.8	0.9		1	1.1	1.2	1.3	1.4	1.5	1.6

	k_{rET}, exp. (Ru) / k_{rET}, exp.(Cr)		**k_{rET}, calc. (Ru) / k_{rET}, calc.(Cr)**												
**TAA-OMe**	0.108	0.000	0.001	0.007	0.040	0.153	0.432		0.992	1.959	3.454	5.580	8.417	12.021	16.418
**TAA-PEG$_3$**	0.100	0.000	0.000	0.002	0.016	0.068	0.209		0.516	1.082	2.004	3.375	5.278	7.775	10.913
**TAA-PEG$_7$**	0.066	0.000	0.000	0.001	0.006	0.030	0.101		0.269	0.597	1.162	2.042	3.309	5.029	7.254
**TAA-Cl**	0.103	0.000	0.000	0.000	0.001	0.004	0.015		0.049	0.127	0.282	0.553	0.983	1.620	2.508
**TAA-Br**	0.028	0.000	0.000	0.000	0.000	0.002	0.010		0.033	0.089	0.203	0.409	0.743	1.248	1.963
**TAA-I**	0.146	0.000	0.000	0.000	0.000	0.001	0.005		0.018	0.052	0.125	0.260	0.488	0.843	1.359
**DMA**	0.028	0.000	0.000	0.001	0.005	0.023	0.081		0.221	0.500	0.987	1.756	2.877	4.413	6.417
**DMT**	0.015	0.000	0.000	0.001	0.011	0.049	0.156		0.398	0.853	1.611	2.760	4.379	6.532	9.268
**DMA-OMe**	0.039	0.001	0.015	0.103	0.415	1.178	2.655		5.084	8.653	13.477	19.607	27.039	35.723	45.581
**THIQ**	0.140	0.000	0.000	0.000	0.003	0.014	0.053		0.149	0.350	0.712	1.299	2.174	3.398	5.022
**TEA**	0.210	0.000	0.000	0.000	0.001	0.007	0.027		0.083	0.205	0.436	0.826	1.428	2.296	3.478
**DIPEA**	0.157	0.000	0.000	0.000	0.002	0.011	0.042		0.123	0.293	0.605	1.117	1.890	2.982	4.443

7.13.2 Spin effects

Considering the electron spin difference between the photoexcited ^{3}MLCT state of $[Ru(bpz)_3]^{2+}$ (Supplementary Fig. 85, left) and the $^2T_1/^2E$ states of $[Cr(dqp)_2]^{3+}$ (Supplementary Fig. 86, left), spin effects might play a non-negligible role and could affect the Φ_{CE} values. Photoinduced electron transfer from any of the investigated donors to ^{3}MLCT-excited $[Ru(bpz)_3]^{2+}$ can result in four different radical pairs as illustrated in the middle of Supplementary Fig. 85. Among them, the formation of radical pairs ① and ② is spin-allowed, because the total spin of the formed oxidized electron donor ($D^{\bullet+}$) and reduced complex $[Ru(bpz)_3]^{+}$ (S = 1) remains unchanged ($\Delta S = 0$) with respect to the initially present ^{3}MLCT excited state of $[Ru(bpz)_3]^{2+}$ ($S_{Ru} = 1$) along with the closed-shell electron donor ($S_D = 0$) (Supplementary Fig. 85, left part). Conversely, thermal reverse electron transfer of the radical pairs ① and ② is spin-forbidden, because the two respective spin-correlated radical pairs comprised of $[Ru(bpz)_3]^{+}$ and $D^{\bullet+}$ (Supplementary Fig. 85, middle) have an overall spin state of S = 1, whereas the product comprised of $[Ru(bpz)_3]^{2+}$ and D has an overall spin state of S = 0 (Supplementary Fig. 85, right). Consequently, (unwanted) in-cage thermal charge recombination is disfavored in these two cases. In contrast, the formation of radical pairs ③ and ④ is spin-forbidden due to the change in total spin from S = 1 of the initial state (Supplementary Fig. 85, left) to S = 0 of the radical pairs (Supplementary Fig. 85, middle). In-cage thermal reverse electron transfer is spin-allowed for radical pairs ③ and ④ (Supplementary Fig. 85, right). Consequently, two (out of four) pathways (upper half of Supplementary Fig. 85), forming radical pairs ① and ② promote high cage escape yields: The initial photoinduced electron transfer is spin-allowed in both cases, and the in-cage thermal charge recombination is spin-forbidden. The combination of these two factors seems helpful for efficient cage escape in the case of $[Ru(bpz)_3]^{2+}$.

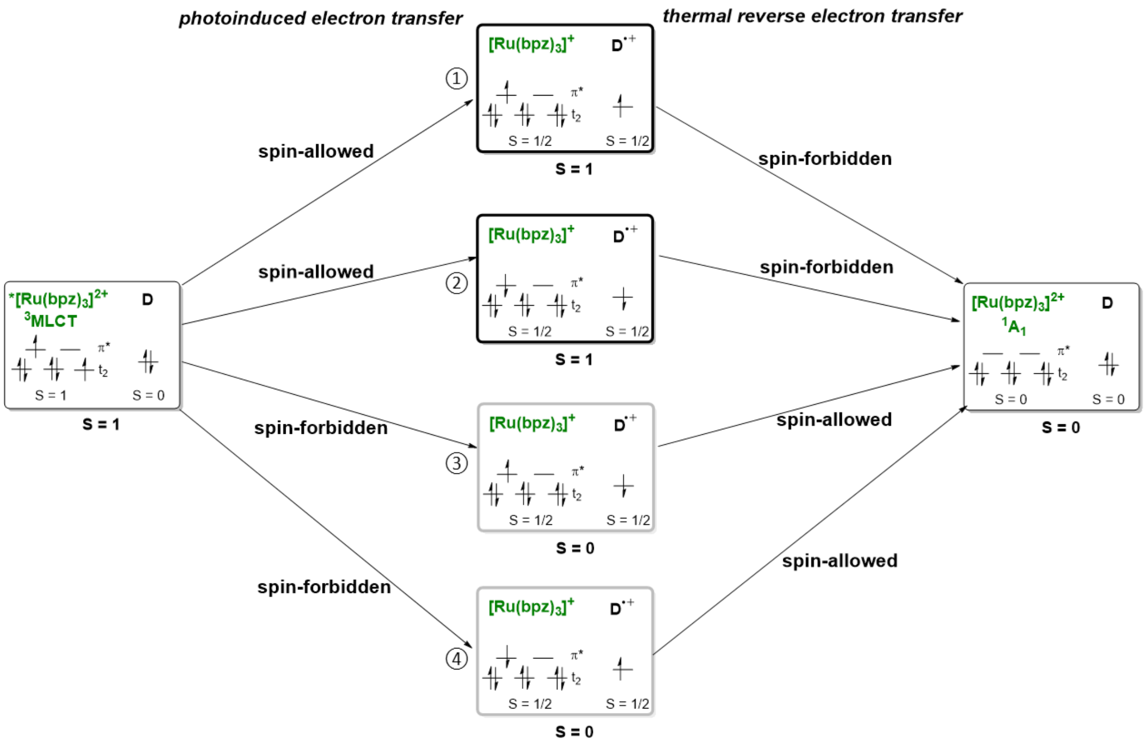

Supplementary Fig. 85: Simplistic consideration of spin selection rules associated with photoinduced electron transfer and thermal reverse electron transfer of a donor-acceptor pair consisting of excited $[Ru(bpz)_3]^{2+}$ and an electron donor (D) in a solvent cage. The two uppermost pathways promote high cage-escape for $[Ru(bpz)_3]^{2+}$, because the initial photoinduced electron transfer step is spin-allowed, whereas (unwanted) in-cage reverse electron transfer is spin-forbidden. The focus here is on selected microstates to illustrate a qualitative point, because it is difficult to represent all relevant microstates graphically in a compact manner. One shortcoming from this simplistic picture is that a third spin-allowed pathway is not shown in the upper half, because a third present microstate for the overall triplet state has not been included. The relevant radical pair states have been discussed previously in the literature.[59,60] Similarly, not all spin-forbidden pathways are graphically represented in the lower half.

Our finding of high cage-escape quantum yields for essentially all reactions with $[Ru(bpz)_3]^{2+}$ is in line with early reports of reductive excited-state quenching of $[Ru(bpy)_3]^{2+}$ by amine-based electron donors or phenols, which found cage escape with yields near unity,[50,58] and reductive quenching of $[Ru(bpz)_3]^{2+}$ by the dianionic form of ethylenediaminetetraacetic acid (EDTA) and triethanolamine (TEOA), which resulted in Φ_{CE} values above 70%.[61,62] These high cage-escape yields obtained for reductive ^{3}MLCT excited-state quenching of Ru^{II} complexes stand in strong contrast to the cage escape yields reported for the same compound class in oxidative quenching reactions. For instance, for photoinduced electron transfer from $[Ru(bpy)_3]^{2+}$ to methyl viologens (MV^{2+}), the observed cage escape quantum yields are substantially lower (42% at most).[46,48,53] The lower Φ_{CE} values obtained from this oxidative excited-state quenching process in comparison to the reductive quenching was previously attributed to the difference in the electronic structure of the quenching products.[46] In the one-electron reduced ruthenium complexes formed upon reductive excited-state quenching, the additional unpaired electron is located in a ligand-based π* orbital, whereas the metal-centered $4(t_{2g})$ orbitals are fully occupied. It has been argued previously that in this $4(t_{2g})^6\ \pi^{*1}$ electron configuration, spin interconversion can hardly occur, due to the strongly limited orbital basis.[46] Consequently, in-cage reverse electron transfer in radical pairs ① and ② of Supplementary Fig. 85 is strongly spin-forbidden, favoring high cage escape quantum yields.

For Cr^{III} compounds, the situation is different than for Ru^{II} complexes, because their photoexcitation commonly populates two doublet excited states which are in thermal equilibrium, namely the ^{2}E state and the $^2T_1$ state (Supplementary Fig. 86, left part), as known for example from ruby, or $[Cr(ddpd)_2]^{3+}$ (ddpd=*N*,*N*′-dimethyl-*N*,*N*′-dipyridin-2-ylpyridine-2,6-diamine), and many other Cr^{III} compounds.[34,63,64]

Previous work on $[Cr(^tbpy)_3]^{3+}$ (^{t}bpy = 4,4'-di-*tert*-butyl-2,2'-bipyridine),[65] in which one-electron reduction is ligand-based (analogously as in the $[Cr(dqp)_2]^{3+}$ complex investigated here),[6,34] indicated that the unpaired electron of ($bpy^{\bullet-}$) in $[Cr(^tbpy)_3]^{2+}$ is antiferromagnetically coupled to the three unpaired spins of the chromium(III) ion with an S_{Cr} = 3/2 ground state (Supplementary Fig. 86, middle part, radical pairs ① and ②). The same overall spin state (S = 1) was also found for one-electron reduced $[Cr(tpy)_2]^{2+}$ (tpy = 2,2':6',2"-terpyridine), in which the reduction is also ligand-based.[66] This has important implications for the expectable radical pairs resulting from photoinduced electron transfer with $[Cr(dqp)_2]^{3+}$, as discussed in the following.

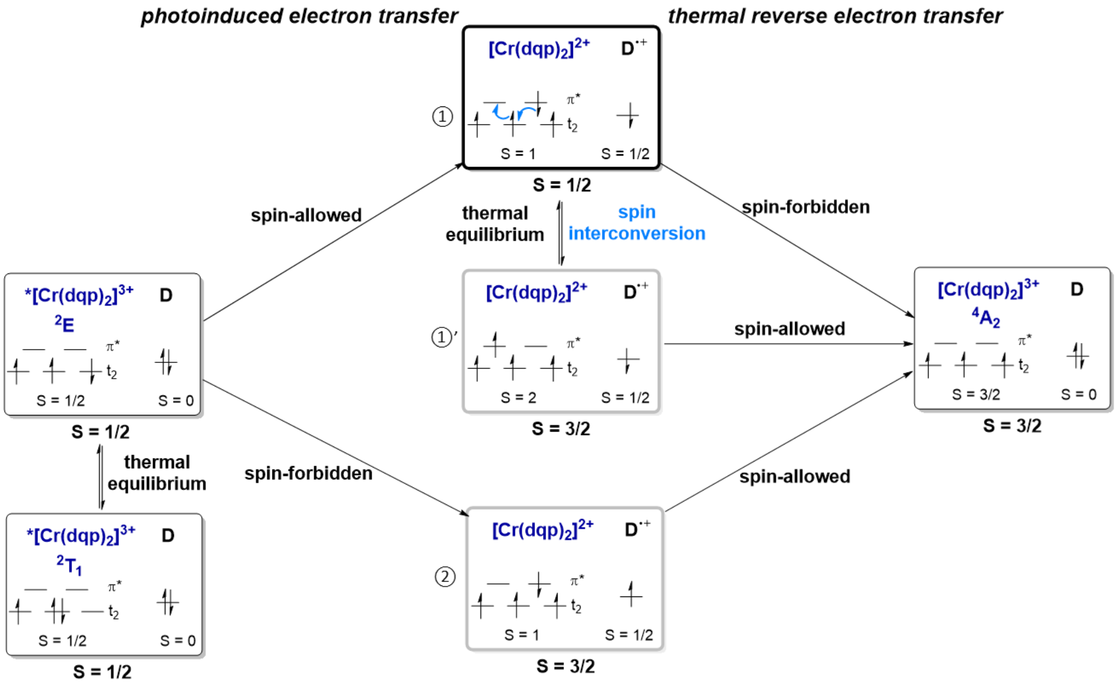

Supplementary Fig. 86: Consideration of spin selection rules associated with photoinduced electron transfer and thermal reverse electron transfer of a donor-acceptor pair consisting of excited $[Cr(dqp)_2]^{3+}$ and an electron donor (D) in a solvent cage. For the uppermost radical pair ①, photoinduced electron transfer is spin-allowed, whereas (unwanted) in-cage reverse electron transfer is spin-forbidden. From radical pair ①, spin interconversion to radical pair ①' (middle) is presumed to be possible following the pathway emphasized by the light blue arrows; this pathway relies on half-filled t_{2g} orbitals (see text below for details). The resulting radical pair ①' (middle) has a total spin of 3/2, leading to spin-allowed in-cage reverse electron transfer to the ground state species (right). The pathway from ① to ①' can account for the low cage escape yields obtained with $[Cr(dqp)_2]^{3+}$. Formation of radical pair ② is spin-forbidden, making this pathway invalid for efficient photoproduct formation.

The abovementioned expectable antiferromagnetic coupling between the ligand-centered electron on the one hand and the three metal-based electrons on the other hand limits the anticipated spin states of the radical pairs comprised of $[Cr(dqp)_2]^{2+}$ and $D^{\bullet+}$. Specifically, only two total spin states of S = 1/2 and S = 3/2, as illustrated in the middle part of Supplementary Fig. 86 (radical pairs ① and ②), are then expected. From the ^2E excited state of $[Cr(dqp)_2]^{3+}$, the formation of radical pair ① (S = 1/2) is spin-allowed, because the total spin remains unchanged (ΔS = 0) with respect to the initially present ^2E-excited state of $[Cr(dqp)_2]^{3+}$ (S_{Cr} = 1/2) along with the closed-shell electron donor (S_D = 0) (Supplementary Fig. 86, left part). Thermal reverse electron transfer from radical pair ① to the $^4A_2$ ground state $[Cr(dqp)_2]^{3+}$ and closed-shell electron donor with a total spin of S = 3/2 is then spin-forbidden (Supplementary Fig. 86, right part). The formation of radical pair ② is spin-forbidden due to the total spin change from S = 1/2 of the initial state to S = 3/2, making this pathway invalid for photoproduct formation. The pathway via radical pair ① is therefore in principle expectable to promote high cage escape yields. However, both the ligand-based π*orbital and the metal-centered t_{2g} orbitals in radical pair ① are only half filled, providing orbital spaces for spin interconversion, as noted

earlier (see above).[46] This might open a pathway for interconversion of radical pair ① into radical pair ①′ (vertical arrows in the middle of Supplementary Fig. 86): In radical pair ①, one of the metal-centered electrons can interconvert to an empty π* orbital, while the electron initially located in another π* orbital can spin flip to the emptied t_{2g} orbital, as indicated with the light blue arrows. This latter step involves a simultaneous change in orbital type (from ligand-based π* to metal-centered t_{2g}) and in spin, conceptually resembling the interconversion between $^1\pi$-π^* and ^{3}n-π^* excited states in carbonyl compounds, which is allowed according to El Sayed's rule.[67,68] The overall interconversion between ① and ①′ relies on t_{2g} orbitals which are not completely filled, hence is inapplicable to the $[Ru(bpz)_3]^{2+}$ complex discussed above. From the resulting radical pair ①′ involving the $[Cr(dqp)_2]^{3+}$ complex, in-cage reverse electron transfer to the ground state species (right) is spin-allowed. Consequently, this pathway is expected to promote in-cage reverse electron transfer and low cage escape quantum yields, as found experimentally for $[Cr(dqp)_2]^{3+}$ in comparison to $[Ru(bpz)_3]^{2+}$. The formation of radical pair ② in Supplementary Fig. 86 is spin-forbidden, making this pathway unimportant for product formation.

The exact magnitude of the antiferromagnetic coupling between the ligand-based electron and the three t_{2g}-electrons on Cr^{III} in $[Cr(dqp)_2]^{2+}$ and in $[Cr(^t bpy)_3]^{2+}$ is not known. However, an exchange coupling constant (J) of -477 cm^{-1} has been reported for the antiferromagnetic coupling between the three $(^t bpy)^{\bullet-}$ radicals and the central Cr^{III} ion in $[Cr(^t bpy)_3]^0$.[65] If the strength of the antiferromagnetic coupling between the single $(dpq)^{\bullet-}$ radical and the central Cr^{III} ion is of similar magnitude (a few hundred wavenumbers), then a substantial thermal population of microstate ①′ seems expectable at room temperature.

To summarize this discussion of spin effects, we note that the fact that the t_{2g}-orbitals in Cr^{III} are only half-filled whereas in Ru^{II} they are completely filled is a key difference. As noted earlier, the completely filled t_{2g} subshell of Ru^{II} does not permit for spin interconversion, due to the strongly limited orbital basis.[46] By contrast, the half-filled t_{2g} subshell of Cr^{III} allows for such spin interconversion, which can make in-cage reverse electron transfer spin-allowed, causing a lower cage-escape yield. The relevant S = 1/2 and S=3/2 total spin states seem thermally within reach of each other.[65]

7.13.3 Heavy atom effects

The possible involvement of a heavy-atom effect on the cage escape quantum yields Φ_{CE} was investigated by exploring the Cl-, Br- and I-substituted variants of triarylamine electron donors. The halide heavy atoms clearly accelerate the initial photoinduced electron transfer,[47] as observed with the increasing k_q values obtained with TAA-Cl, TAA-Br and TAA-I (Supplementary Table 1), despite decreasing driving-force ΔG_{ET} along this series. For $[Ru(bpz)_3]^{2+}$, a higher Φ_{CE} value was obtained when going from TAA-Cl to TAA-Br, whereas similar Φ_{CE} values were obtained with TAA-Cl and TAA-I. Heavy atoms can cause a relaxation of the spin selection rule, thereby facilitating intersystem crossing between radical pairs and making individual electron transfer processes less spin-forbidden,[69] for instance thermal reverse electron transfer in the two pathways in the upper half of Supplementary Fig. 85. This could promote in-cage recombination and might explain the decrease of Φ_{CE} when going from TAA-Br to TAA-I in the case of $[Ru(bpz)_3]^{2+}$. The increase of the Φ_{CE} value when going from the $[Ru(bpz)_3]^{2+}$/TAA-Cl pair to the $[Ru(bpz)_3]^{2+}$/TAA-Br pair counteracts this expectation and is possibly attributable to the steric hindrance caused by the larger Br atoms. In the case of $[Cr(dqp)_2]^{3+}$, the observable differences between Φ_{CE} obtained with the TAA-Cl, TAA-Br and TAA-I electron donors (Supplementary Table 1) are within experimental uncertainty. Heavy atom effects seem negligible in this case.

7.13.4 Size and viscosity effects

By introducing sterically demanding polyethylene glycol (PEG) chains at the *p*-positions of our triarylamine based electron donor to obtain bulkier analogs of TAA-OMe, resulting in TAA-PEG_3 and TAA-PEG_7, we aimed to investigate the influence of donor size on cage escape. The experimentally determined Φ_{CE} values tend to increase with increasing donor size due to the longer PEG chains, both with $[Ru(bpz)_3]^{2+}$ and $[Cr(dqp)_2]^{3+}$. This observation could be due to the combined effects of an enhanced steric hindrance of the donors, leading to better distancing of the donor-acceptor pair, thus promoting cage escape of the radical species,[46,52] and the increased viscosity of the solutions due to the PEG chains.[45] The decrease of ΔG_{rET} (Supplementary Table 1) is due to the increasing oxidation potential of these electron donors with PEG chains. In principle, the PEG substituents are not expected to affect the redox potentials,[70] yet our data in Supplementary Table 1 indicate that this is a non-negligible effect. Lower rate constants k_q for photoinduced electron transfer were obtained with increasing PEG chain length, possibly due to steric hindrance and a decreased driving force ΔG_{ET}. It seems plausible that the hydrophilic nature of the PEG units can contribute to slow down photoinduced electron transfer between the relatively hydrophobic donor-acceptor species,[71] and the enhanced viscosity of the solutions caused by the PEG chains might furthermore affect the rate of photoinduced electron transfer.[45] Additionally, the Φ_{CE} values obtained with electron donors comprising aromatic rings, i.e. triarylamine- and dimethylaniline-based donors, are higher than those obtained with aliphatic donors such as TEA and DIPEA. A similar observation was made for the reductive excited-state quenching of cyanoanthracene by aromatic hydrocarbon donors, where donors containing three or two aromatic rings led to higher Φ_{CE} values than donors with only one aromatic ring.[52]

7.13.5 Ionic strength

The ionic strength of solutions containing $[Cr(dqp)_2][PF_6]_3$ (30 µM) is expected to be higher than of those solutions containing $[Ru(bpz)_3][PF_6]_2$ (12 µM). In principle, higher ionic strength can be expected to yield lower Φ_{CE} values,[44,50,51,53] in line with our observations here. However, at micromolar concentrations, this effect is likely not dominant.

7.13.6 Electrostatic repulsion

The electrostatic repulsion between $[Ru(bpz)_3]^+$ and the oxidized radical cation in the solvent cage is expected to be weaker than the repulsion between $[Cr(dqp)_2]^{2+}$ and $D^{\bullet+}$. Consequently, differences in electrostatic repulsion between the spin-correlated radical pair in the solvent cage cannot account for the lower cage escape quantum yields observed with the Cr^{III} complex in comparison to the Ru^{II} complex.

8. Photocatalytic reactions

The photocatalytic behavior of $[Ru(bpz)_3]^{2+}$ and $[Cr(dqp)_2]^{3+}$ was compared in three different photochemical reactions (details given below). The reaction conditions with both photocatalysts were strictly controlled to be exactly the same: i) the concentrations of the photocatalysts were adjusted such as to ensure the same absorbance at the irradiation wavelength of 415 nm emitted by an LED equipped with a 400 nm long pass filter; the emission profile of that irradiation setup features a relatively narrow emission band, as shown in Supplementary Fig. 87; ii) reaction mixtures that were investigated in comparative fashion with $[Ru(bpz)_3]^{2+}$ and $[Cr(dqp)_2]^{3+}$ differed only in the photocatalyst,

i.e., substrates, electron donors, and the internal reference were prepared previously in common stock solutions, to ensure that both reaction mixtures have the same concentrations; iii) NMR tubes with different photocatalysts were put next to each other in a stirred water bath with the same distance (13 cm) to the 415 nm LED and were irradiated in parallel, as illustrated in Supplementary Fig. 88. The beam at the sample position had a round shape with a diameter of approximately 3 cm.

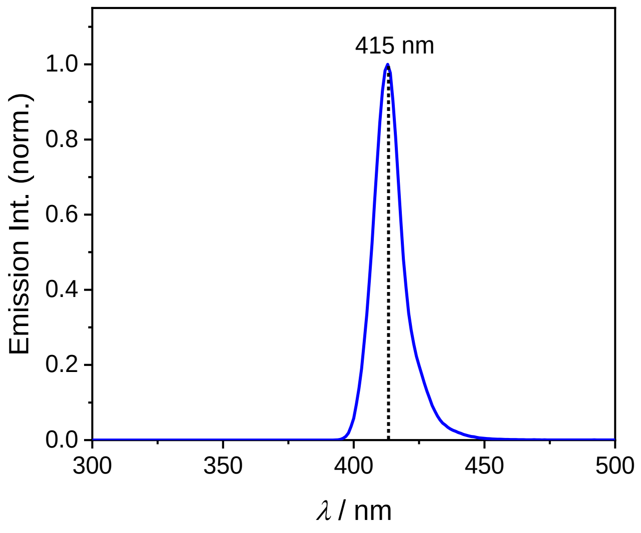

Supplementary Fig. 87: Normalized irradiation profile of the 415 nm LED (7 W output) equipped with a 400 nm long pass filter.

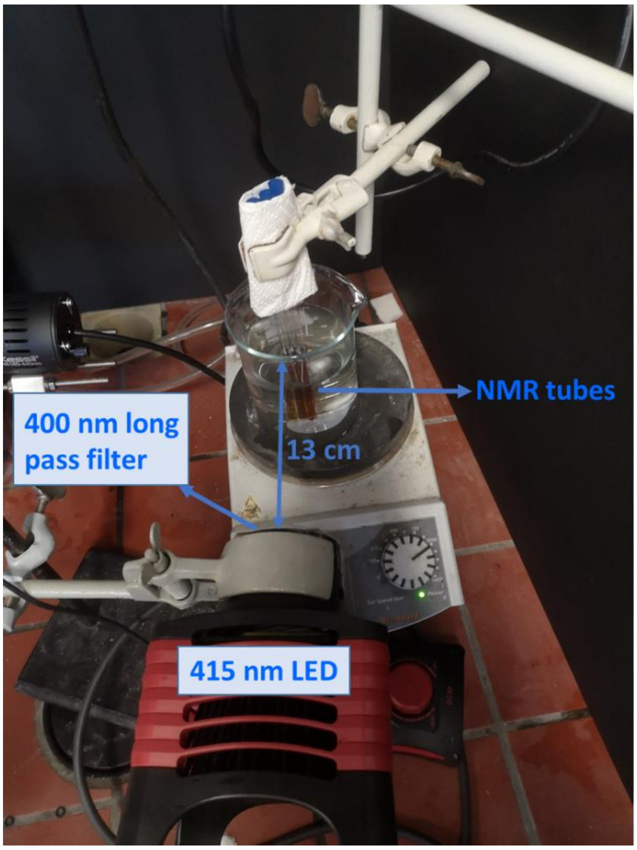

Supplementary Fig. 88: Irradiation setup for photochemical reactions.

8.1 Photocatalytic aerobic hydroxylation of arylboronic acid

4-Methoxylphenylboronic acid (50 mM, 1.0 eq.), *N*,*N*'-diisopropylethylamine (DIPEA, 250 mM, 5.0 eq.), and phenyltrimethylsilane (50 mM, 1.0 eq.) or hexamethylcyclotrisiloxane (10 mM, 0.2 eq.) as the internal standard were dissolved in air-saturated CD_3CN/D_2O (4/1, v/v) as a stock solution. Photocatalysts (PC) $[Cr(dqp)_2][PF_6]_3$ (348 µg, 500 µM, 1.0 mol%) and $[Ru(bpz)_3][PF_6]_2$ (104 µg, 200 µM, 0.4 mol%) were each dissolved separately in 600 µL of the stock solution. After measuring the UV/vis absorption spectra of the two reaction mixtures to ensure that their absorbance at 415 nm is identical, the solutions were transferred to NMR tubes. Followed by ^{1}H-NMR measurements of the freshly prepared samples, the tubes were irradiated at room temperature with a 415 nm LED (7.0 W) equipped with a 400 nm long pass filter. The reaction mechanism for the aerobic hydroxylation of 4-methoxylphenylboronic acid as suggested previously in literature is shown in Supplementary Fig. 89.[72-74]

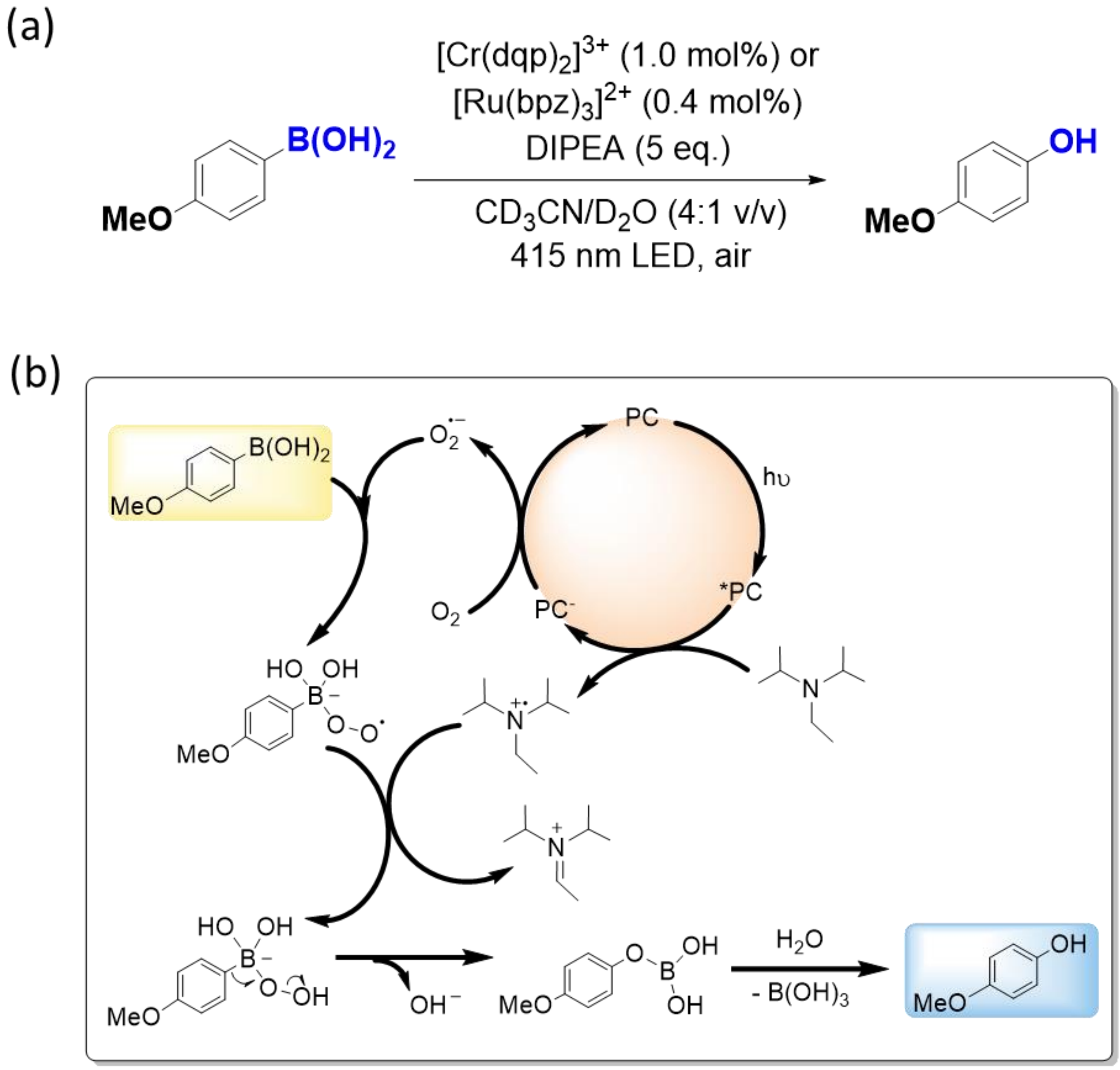

Supplementary Fig. 89: (a) Photocatalytic aerobic hydroxylation of 4-methoxylphenylboronic acid. The reaction mixture included $[Cr(dqp)_2]^{3+}$ (1 mol%) or $[Ru(bpz)_3]^{2+}$ (0.4 mol%) as the photocatalyst (PC), 4-methoxylphenylboronic acid (50 mM), internal reference phenyltrimethylsilane (50 mM, 1.0 eq.) or hexamethylcyclotrisiloxane (10 mM, 0.2 eq.), and *N*,*N*-diisopropylethylamine (DIPEA, 250 mM, 5 eq.) in air-saturated CD_3CN/D_2O (4/1, v/v). Irradiation occurred with a 415 nm LED (max. output 7.0 W) at room temperature, equipped with a 400 nm long pass filter. (b) Plausible reaction mechanism as proposed previously for this reaction type with other photocatalysts.[72-74] The $[Cr(dqp)_2]^{3+}$ or $[Ru(bpz)_3]^{2+}$ photocatalyst concentrations were adjusted such as to ensure the absorption of equal amounts of light at 415 nm.

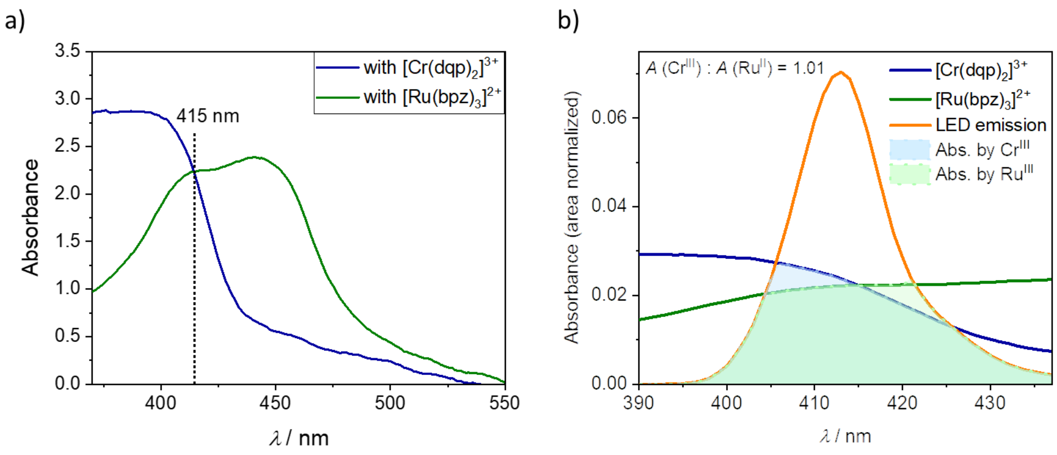

Supplementary Fig. 90: a) UV/vis absorption spectra of the reaction mixtures containing 4-methoxylphenylboronic acid (50 mM, 1.0 eq.), *N,N'*-diisopropylethylamine (DIPEA, 250 mM, 5.0 eq.), hexamethylcyclotrisiloxane as the reference (10 mM, 0.2 eq.), and $[Cr(dqp)_2]^{3+}$ (500 µM, 1.0 mol%, blue solid line) or $[Ru(bpz)_3]^{2+}$ (200 µM, 0.4 mol%, green solid line) in CD_3CN/D_2O (4/1, v/v). At 415 nm both solutions have identical absorbance. b) Area-normalized absorption spectra of the LED emission profile (orange solid line) and the two solutions from a) in the range of 390 - 437 nm. The integral of the LED emission was normalized to 1 (area under orange solid line) in this wavelength range. The absorption spectra of $[Ru(bpz)_3]^{2+}$ (green line) and $[Cr(dqp)_2]^{3+}$ (blue line) in the same wavelength range were equally normalized to yield an integral of 1.0. The resulting spectral overlap integrals between the LED emission and the $[Ru(bpz)_3]^{2+}$ and $[Cr(dqp)_2]^{3+}$ absorption profiles differ only by 1%. This indicates that both photocatalysts absorb nearly the same amount of light under the photoredox catalysis conditions.

Under 7.0 W LED irradiation

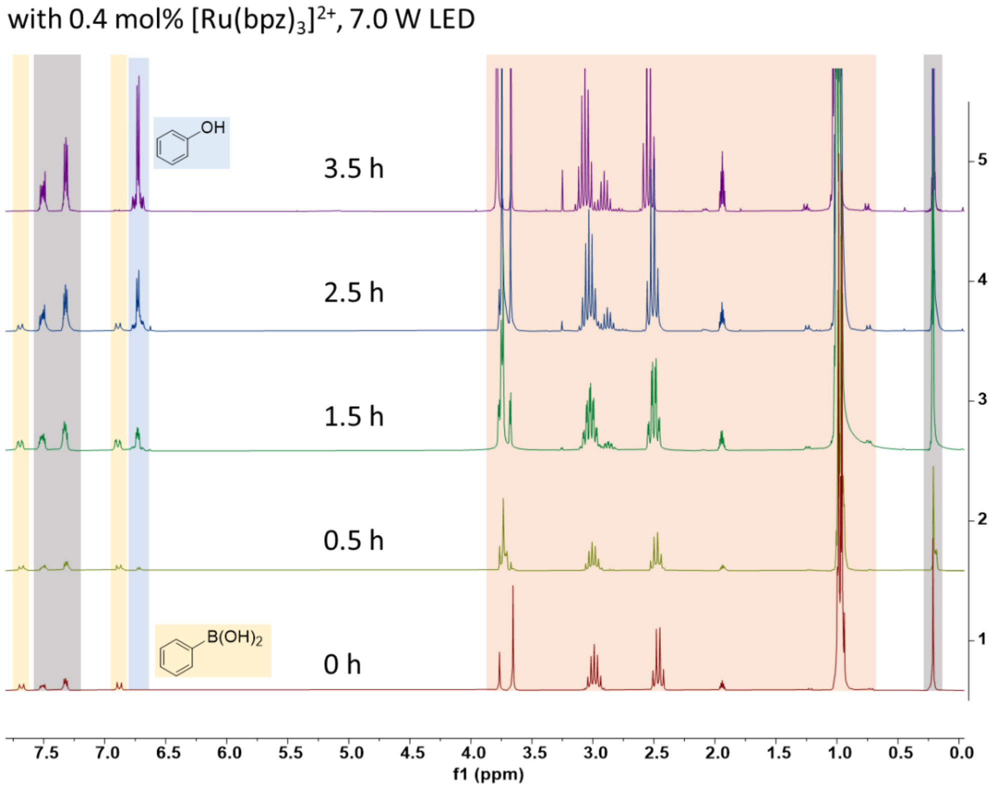

Supplementary Fig. 91: ^{1}H-NMR spectra of a solution of 50 mM 4-methoxylphenylboronic acid, 5 eq. N,N'-diisopropylethylamine (DIPEA), 0.4 mol% $[Ru(bpz)_3]^{2+}$, and 1 eq. phenyltrimethylsilane as internal reference in air-saturated CD_3CN/D_2O (4/1, v/v) at room temperature under 415 nm LED (7.0 W, equipped with a 400 nm long pass filter) irradiation. The individual spectra were recorded after the indicated irradiation times. The characteristic NMR resonances of the 4-methoxylphenylboronic acid starting material are marked by the yellow shaded areas, the signals marked in orange are assigned to DIPEA and solvent signals, the reference is marked in grey, and the 4-methoxylphenol product is marked by the blue area. For determination of the product yield, the integral of the proton signals at 0.2 ppm from the trimethylsilane group

of phenyltrimethylsilane was used as the internal reference. Product formation was verified by comparison to the ^{1}H-NMR spectra of the same photochemical reaction previously published by us (see Supplementary Fig. 120).[6]

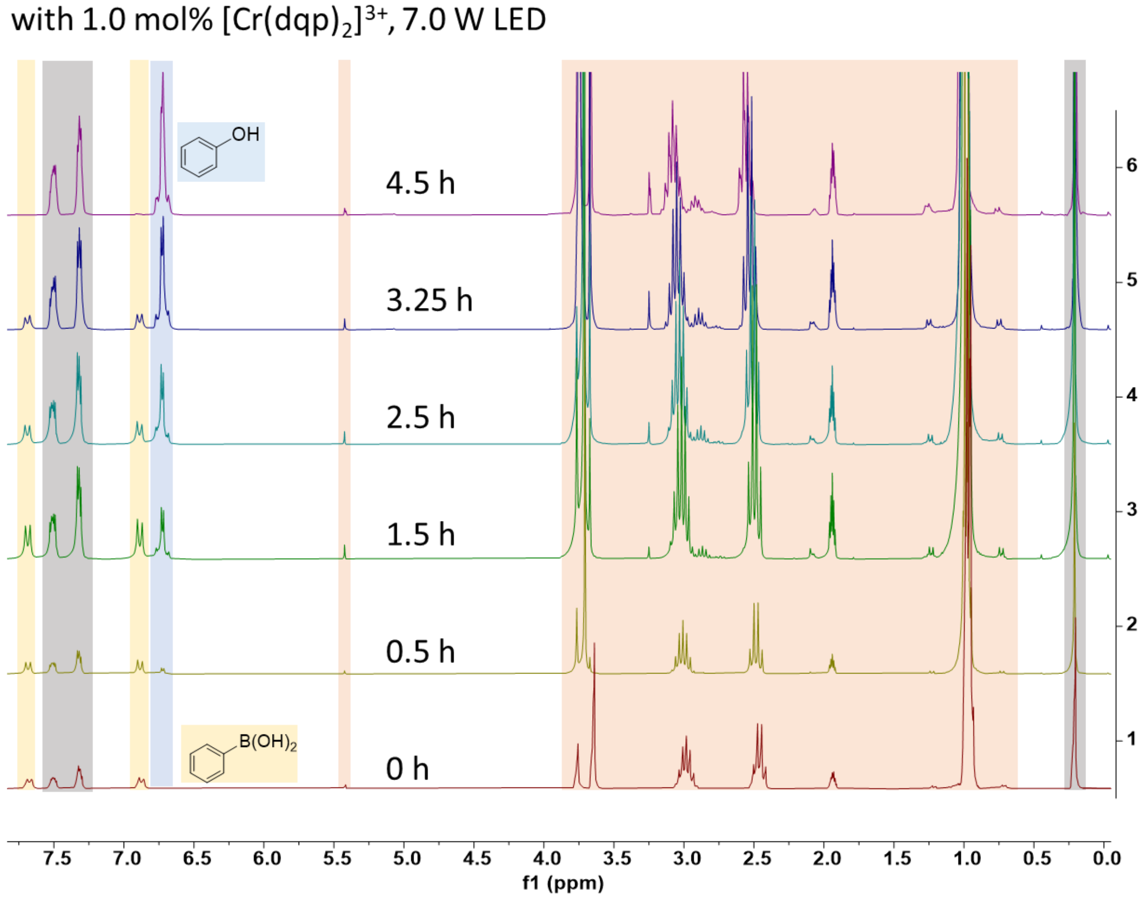

Supplementary Fig. 92*:* ^{1}H-NMR spectra of a solution of 50 mM 4-methoxylphenylboronic acid, 5 eq. *N,N'*-diisopropylethylamine (DIPEA), 1.0 mol% $[Cr(dqp)_2]^{3+}$, and 1 eq. phenyltrimethylsilane as internal reference in air-saturated CD_3CN/D_2O (4/1, v/v) at room temperature under 415 nm LED (7.0 W, equipped with a 400 nm long pass filter) irradiation. The individual spectra were recorded after the indicated irradiation times. The characteristic NMR resonances of the 4-methoxylphenylboronic acid starting material are marked by the yellow shaded areas, the signals marked in orange are assigned to DIPEA and solvent signals, the reference is marked in grey, and the 4-methoxylphenol product is marked by the blue area. For determination of the product yield, the integral of the proton signals at 0.2 ppm from the trimethylsilane group of phenyltrimethylsilane was used as the internal reference. Product formation was verified by comparison to the ^{1}H-NMR spectra of the same photochemical reaction previously published by us (see Supplementary Fig. 120).[6]

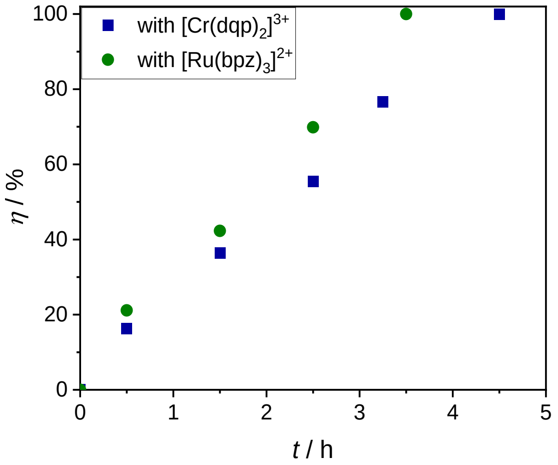

Supplementary Fig. 93: 4-Methoxylphenol product yields (η) resulting from the photocatalytic aerobic hydroxylation of 4-methoxylphenylboronic acid catalyzed by $[Cr(dqp)_2]^{3+}$ (1.0 mol%, blue squares) and $[Ru(bpz)_3]^{2+}$ (0.4 mol%, green circles) as a function of irradiation time (415 nm LED, 7.0 W output, equipped with a 400 nm long pass filter).

With the maximal output power of the 415 nm LED (7.0 W), the 4-methoxylphenol product yields η reached near 100% within 3.5 h and 4.5 h of irradiation with both the $[Ru(bpz)_3]^{2+}$ and $[Cr(dqp)_2]^{3+}$ photocatalysts, respectively (Supplementary Fig. 91 and Supplementary Fig. 92). Under these high-power irradiation conditions, only a slightly faster reaction rate is observed with $[Ru(bpz)_3]^{2+}$ in comparison to $[Cr(dqp)_2]^{3+}$ (Supplementary Fig. 93). A more significant difference might be expected based on the roughly 4-fold higher cage escape quantum yield of the $[Ru(bpz)_3]^{2+}$ / DIPEA pair compared to the $[Cr(dqp)_2]^{3+}$/DIPEA pair (see section 7.12). Under the conditions of intense irradiation, photodecomposition of $[Ru(bpz)_3]^{2+}$ could likely occur as previously observed,[75] potentially slowing down the reaction rate with the Ru^{II} complex over time.

Under 230 mW LED irradiation

To minimize photodegradation, the power of the 415 nm LED was turned down to 230 mW, yielding a significantly weak power density of 73 mW cm^{-2} at the sample position. Under this irradiation condition, the reaction kinetics were studied again.

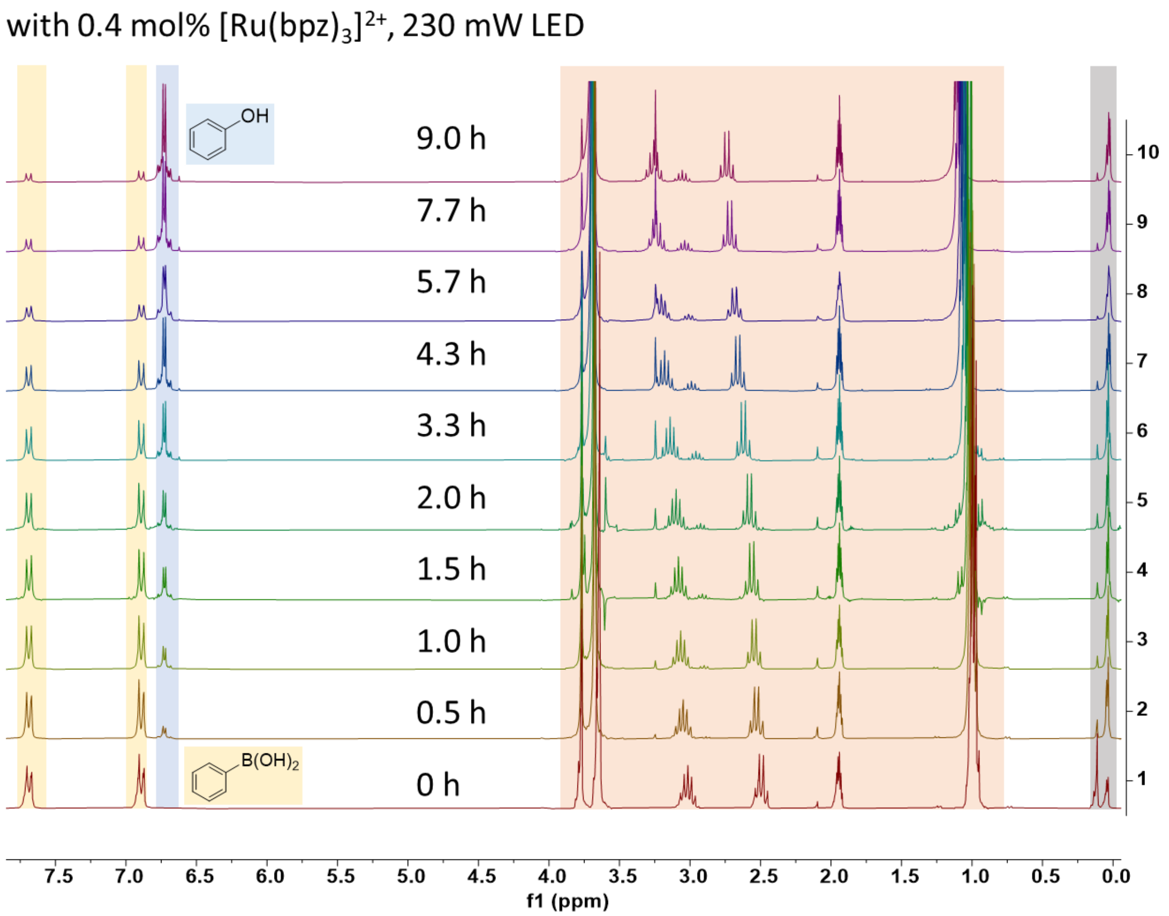

Supplementary Fig. 94: ^{1}H-NMR spectra of a solution of 50 mM 4-methoxylphenylboronic acid, 5 eq. N,N'-diisopropylethylamine (DIPEA), 0.4 mol% $[Ru(bpz)_3]^{2+}$, and 0.2 eq. hexamethylcyclotrisiloxane as internal reference in air-saturated CD_3CN/D_2O (4/1, v/v) at room temperature under 415 nm LED (230 mW, equipped with a 400 nm long pass filter) irradiation. The individual spectra were recorded after the indicated irradiation times. The characteristic NMR resonances of the 4-methoxylphenylboronic acid starting material are marked by the yellow shaded areas, the signals marked in orange are assigned to DIPEA and solvent signals, the reference is marked in grey, and the 4-methoxylphenol product is marked by the blue area. For determination of the product yield, the integral of the proton signals at 0.2 ppm from the trimethylsilane group of phenyltrimethylsilane was used as the internal reference. Product formation was verified by comparison to the ^{1}H-NMR spectra of the same photochemical reaction previously published by us (see Supplementary Fig. 120).[6]

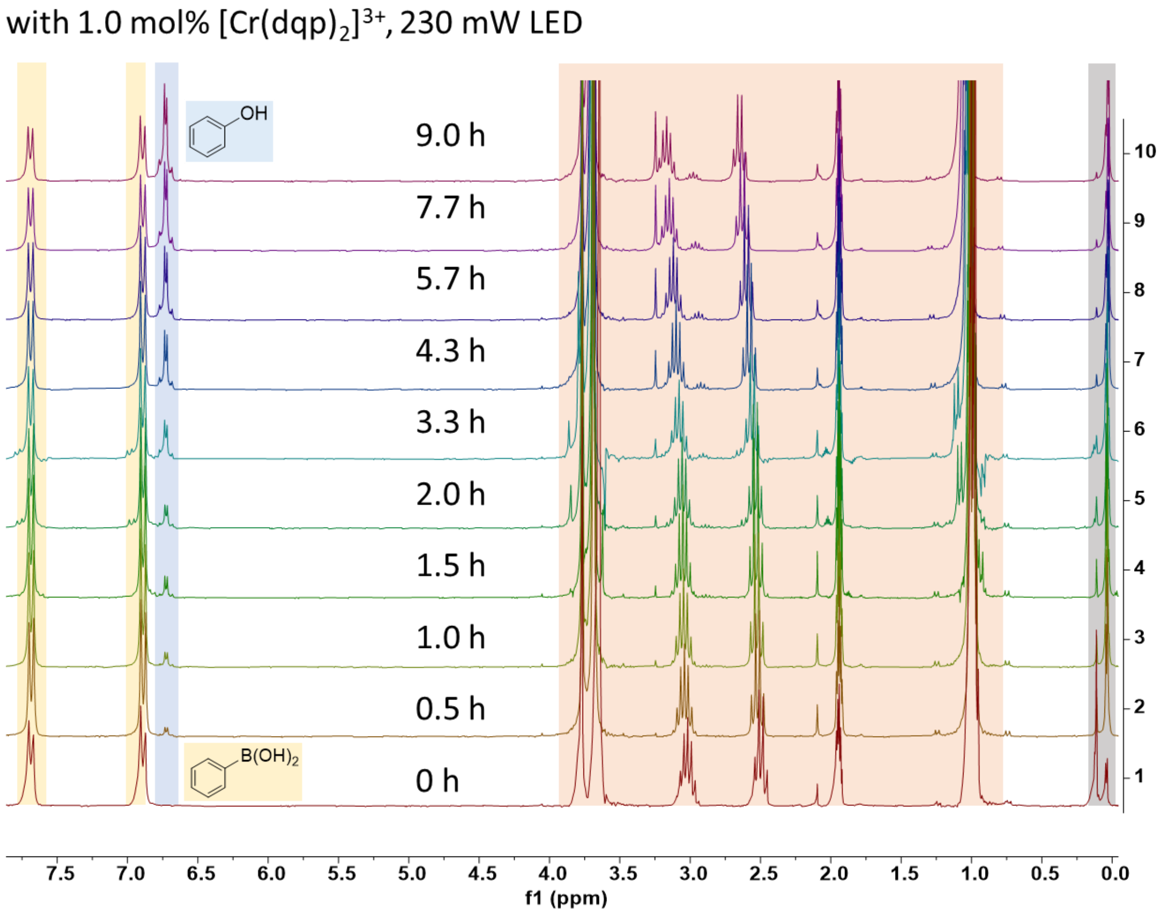

Supplementary Fig. 95: ^{1}H-NMR spectra of a solution of 50 mM 4-methoxylphenylboronic acid, 5 eq. *N*,*N'*-diisopropylethylamine (DIPEA), 1.0 mol% $[Cr(dqp)_2]^{3+}$, and 0.2 eq. hexamethylcyclotrisiloxane as internal reference in air-saturated CD_3CN/D_2O (4/1, v/v) at room temperature under 415 nm LED (230 mW, equipped with a 400 nm long pass filter) irradiation. The individual spectra were recorded after the indicated irradiation times. The characteristic NMR resonances of the 4-methoxylphenylboronic acid starting material are marked by the yellow shaded areas, the signals marked in orange are assigned to DIPEA and solvent signals, the reference is marked in grey, and the 4-methoxylphenol product is marked by the blue area. For determination of the product yield, the integral of the proton signals at 0.2 ppm from the trimethylsilane group of phenyltrimethylsilane was used as the internal reference. Product formation was verified by comparison to the ^{1}H-NMR spectra of the same photochemical reaction previously published by us (see Supplementary Fig. 120).[6]

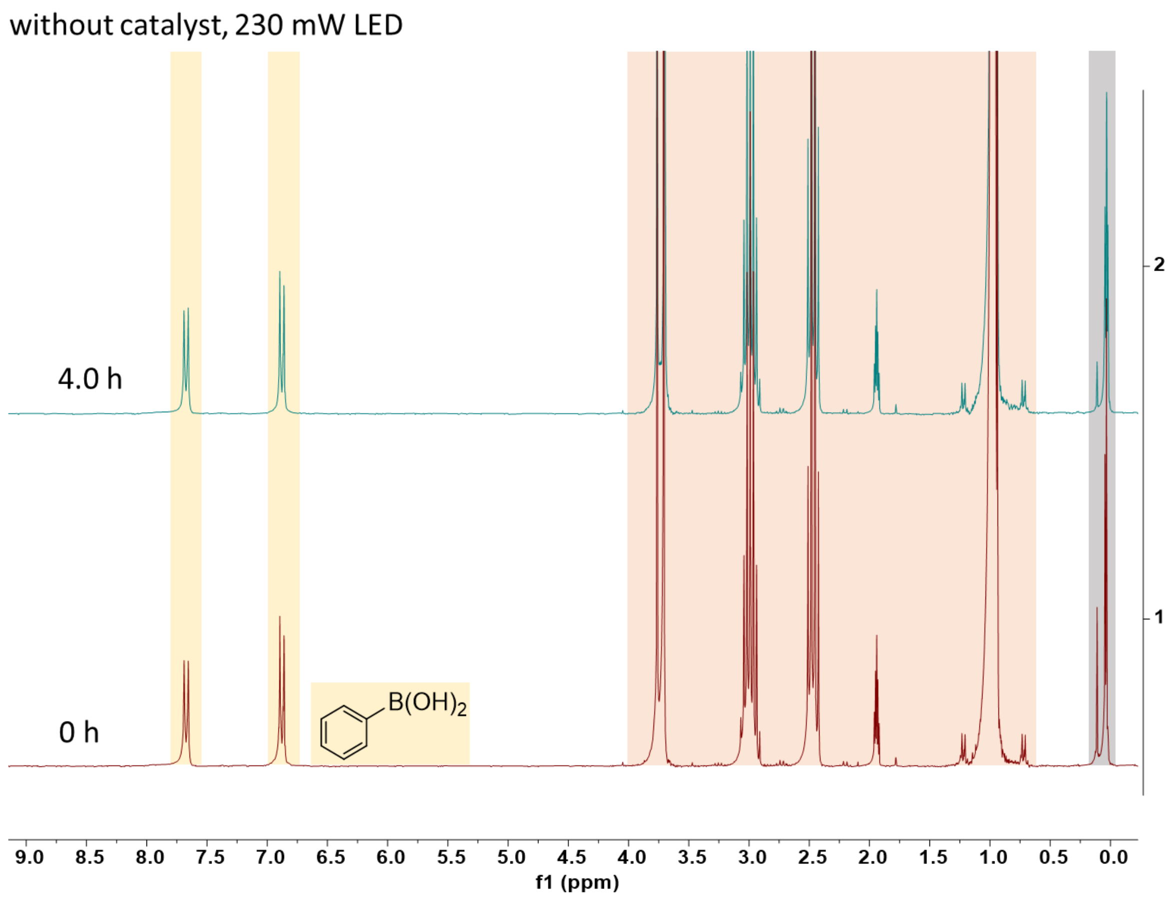

Supplementary Fig. 96: Control experiment performed in the absence of any photosensitizer: ^{1}H-NMR spectra of a solution of 50 mM 4-methoxylphenylboronic acid, 5 eq. N,N'-diisopropylethylamine (DIPEA), and 0.2 eq. hexamethylcyclotrisiloxane as internal reference in oxygen saturated CD_3CN/D_2O (4/1, v/v) under 415 nm LED (230 mW) irradiation at room temperature. The individual spectra were recorded after the indicated irradiation times. The characteristic NMR signals of the 4-methoxylphenylboronic acid starting material are marked by the yellow shaded areas, the resonances marked in orange are assigned to DIPEA and solvent signals, the hexamethylcyclotrisiloxane reference is marked in grey. After four hours irradiation, no new signals were observed.

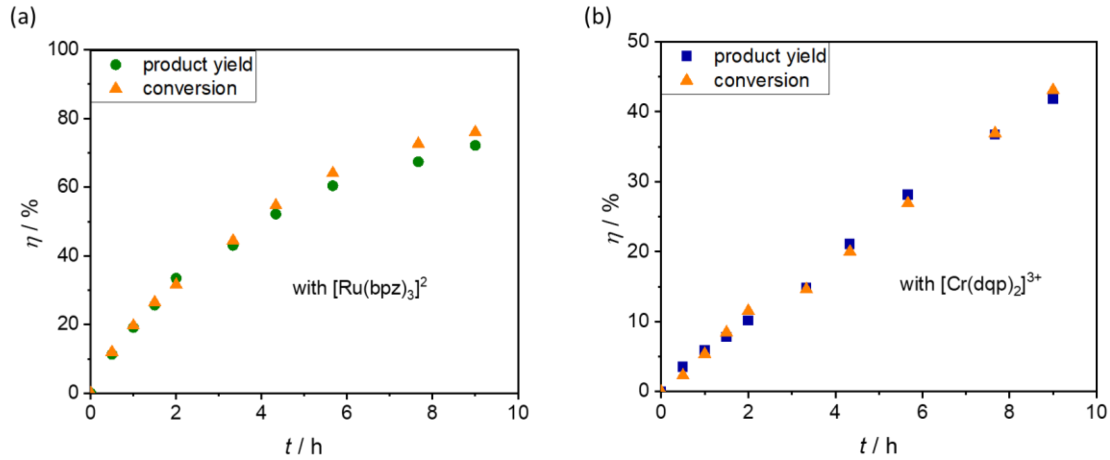

Supplementary Fig. 97: 4-Methoxylphenol product yields and conversion resulting from the photocatalytic aerobic hydroxylation of 4-methoxylphenylboronic acid catalyzed by (a) $[Ru(bpz)_3]^{2+}$ (0.4 mol%, product yields in green circles and conversion in orange triangles, derived from Supplementary Fig. 94) and (b) $[Cr(dqp)_2]^{3+}$ (1.0 mol%, product yields in blue squares and conversion in orange triangles, Supplementary Fig. 95) as a function of irradiation time (415 nm LED, 230 mW). In both cases, the product yields of 4-methoxylphenol are similar to the conversion of the 4-methoxylphenylboronic acid over the entire irradiation time, indicating that no substantial amounts of side products are formed under these conditions.

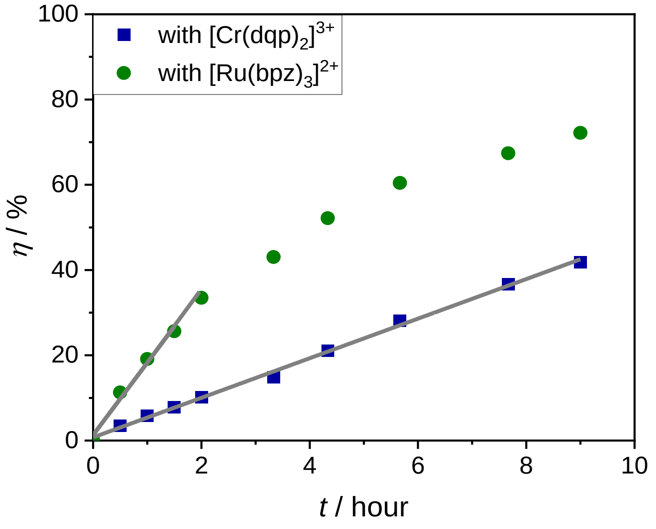

Supplementary Fig. 98: 4-Methoxylphenol product yields of the photocatalytic aerobic hydroxylation of 4-methoxylphenylboronic acid catalyzed by $[Cr(dqp)_2]^{3+}$ (1.0 mol%, blue squares) and $[Ru(bpz)_3]^{2+}$ (0.4 mol%, green circles) as a function of irradiation time (415 nm LED, 230 mW). Linear fits (grey solid lines) of the product yields as a function of time in the initial irradiation periods provide the initial reaction rates. These initial reaction rates (848 ± 77 mM h^{-1} for $[Ru(bpz)_3]^{2+}$ and 232 ± 4 mM h^{-1} for $[Cr(dqp)_2]^{3+}$) exhibit a ratio of $v(Ru^{II}) : v(Cr^{III})$ = 3.7 ± 0.3, approaching the cage escape quantum yields ratio $\Phi_{CE}(Ru^{II}) : \Phi_{CE}(Cr^{III})$ = 4.0 ± 0.7 obtained for the $[Ru(bpz)_3]^{2+}$ / DIPEA and $[Cr(dqp)_2]^{3+}$ / DIPEA donor-acceptor couples, Supplementary Table 1.

In the absence of photocatalyst, no reaction occurred under blue light irradiation, as shown in Supplementary Fig. 96. In the presence of photocatalyst, i.e., $[Cr(dqp)_2]^{3+}$ or $[Ru(bpz)_3]^{2+}$, the desired phenol product was formed upon light irradiation. Following the reaction kinetics with ^{1}H NMR measurements, the product yields of 4-methoxylphenol are similar to the conversion of 4-methoxylphenylboronic both with $[Cr(dqp)_2]^{3+}$ and $[Ru(bpz)_3]^{2+}$ as photocatalyst (Supplementary Fig. 97), indicating that unwanted side products are not formed in significant quantities in the course of these photoreactions. The initial reaction rate was determined to be 232 ± 4 mM h^{-1} and 848 ± 77 mM h^{-1} for $[Cr(dqp)_2]^{3+}$ or $[Ru(bpz)_3]^{2+}$, respectively (Supplementary Fig. 98). This corresponds to an initial reaction rate ratio of $v(Ru^{II}) : v(Cr^{III})$ = 3.7 ± 0.3, in good agreement with the cage escape quantum yield ratio of $\Phi_{CE}(Ru^{II}) : \Phi_{CE}(Cr^{III})$ = 4.0 ± 0.7 determined for the $[Ru(bpz)_3]^{2+}$ / DIPEA and $[Cr(dqp)_2]^{3+}$ / DIPEA donor-acceptor couples (Supplementary Table 1). Based on the electron transfer rate constants k_q in Supplementary Table 1 and the intrinsic lifetimes τ_0 of 565 ns for $[Ru(bpz)_3]^{2+}$ and 30 µs for $[Cr(dqp)_2]^{3+}$ in aerated acetonitrile at room temperature, the efficiency of photoinduced electron transfer Φ_{ET} in the presence of 0.25 M DIPEA can be estimated. The respective Φ_{ET} values are 99.85% for $[Ru(bpz)_3]^{2+}$ and 99.98% for $[Cr(dqp)_2]^{3+}$. Thus, photoinduced electron transfer is quantitative in both cases and therefore is unlikely to be a performance-limiting factor for the overall photoredox reaction.

The photoreactions above were performed in CD_3CN/D_2O 4:1 mixture, whereas the laser spectroscopic cage-escape studies were conducted in neat acetonitrile. Based on initial solvent screening studies, we expect that the cage escape quantum yield for the CD_3CN/D_2O 4:1 mixture is within experimental accuracy the same as for neat acetonitrile.

8.2 Photocatalytic reductive debromination of 2-bromoacetophenone

2-Bromoacetophenone (10 mM, 1.0 eq.), *N,N*-dimethyl-*p*-toluidine (DMT, 100 mM, 10.0 eq.), and hexamethylcyclotrisiloxane (2 mM, 0.2 eq.) as the internal standard were dissolved in CD_3CN as a stock

solution. This mixture was purged with Ar for 15 min. Photocatalysts (PC) $[Cr(dqp)_2]^{3+}$ (139 µg, 200 µM, 2.0 mol%) and $[Ru(bpz)_3]^{2+}$ (42 µg, 80 µM, 0.8 mol%) were dissolved in 600 µL of the degassed stock solution, respectively, and sealed in NMR tubes under argon protection with a balloon. After initial ^{1}H-NMR measurements of the freshly prepared samples, the NMR tubes were irradiated at room temperature with a 415 nm LED (230 mW) equipped with a 400 nm long pass filter. A plausible mechanism, as previously proposed for this reaction, is shown in Supplementary Fig. 99b.[76-78]

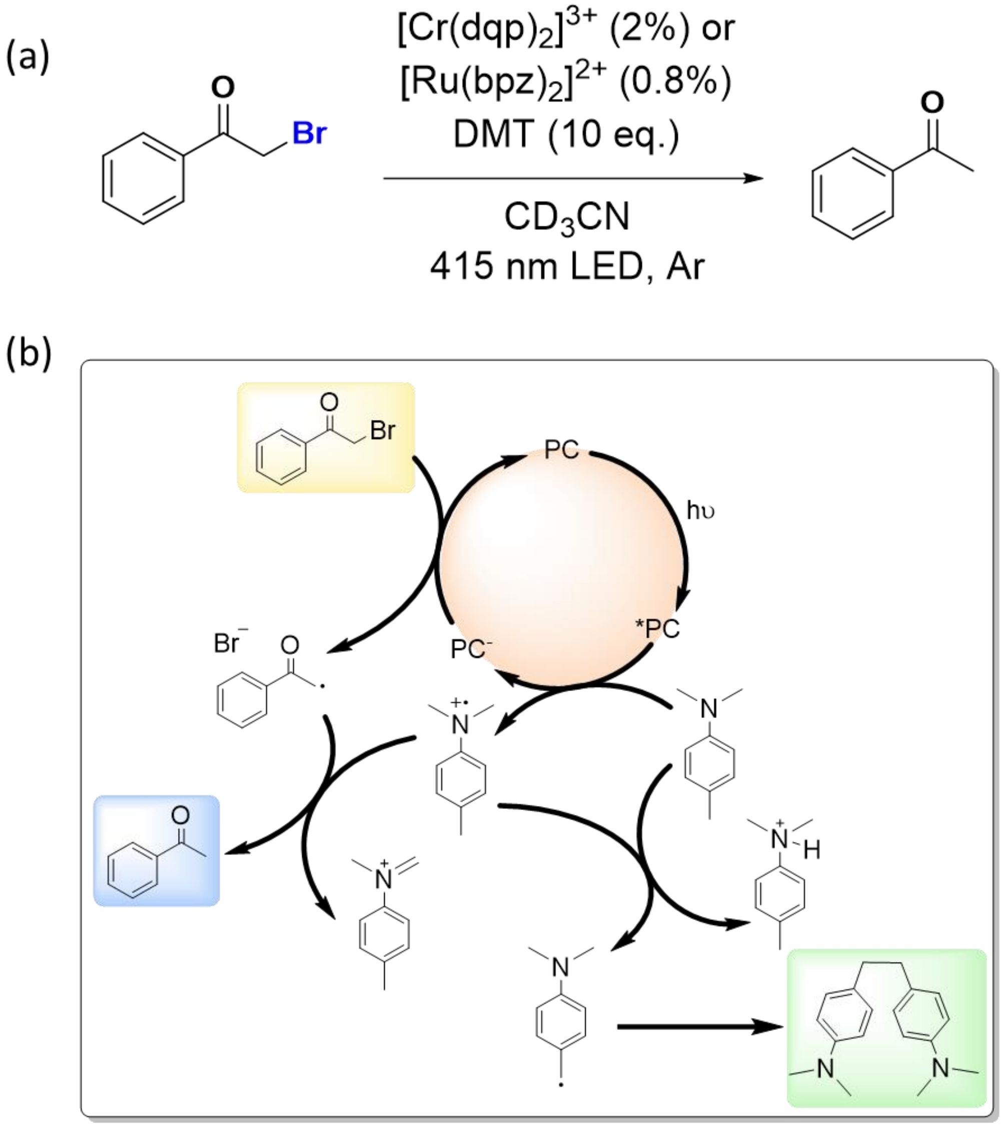

Supplementary Fig. 99: (a) Photocatalytic reductive debromination of 2-bromoacetophenone. The reaction mixture includes $[Cr(dqp)_2]^{3+}$ (2 mol%) or $[Ru(bpz)_3]^{2+}$ (0.8 mol%) as the photocatalyst (PC), 2-bromoacetophenone (10 mM), internal reference hexamethylcyclotrisiloxane (2 mM, 0.2 eq.), and *N*,*N*-dimethyl-*p*-toluidine (DMT, 100 mM, 10 eq.) in Ar-saturated CD_3CN. Irradiation occurred with a 415 nm LED irradiation (230 mW) at room temperature. (b) Plausible reaction mechanism as proposed previously for different photocatalysts,[76-78] here furthermore including a previously proposed pathway for the dimerization of DMT.[16] Photocatalyst (PC) indicates here $[Cr(dqp)_2]^{3+}$ or $[Ru(bpz)_3]^{2+}$.

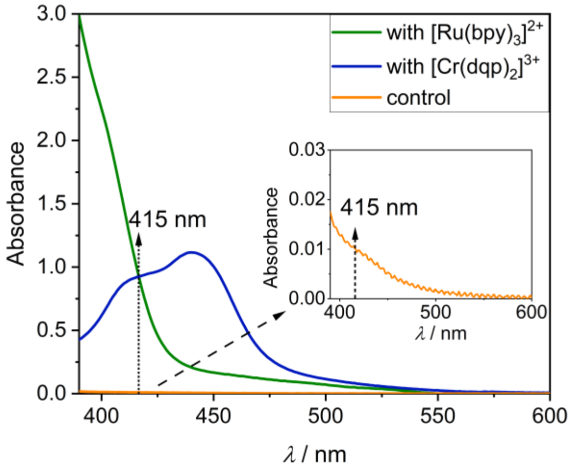

Supplementary Fig. 100: UV/vis absorption spectra of the reaction mixtures containing 2-bromoacetophenone (10 mM, 1.0 eq.), N,N-dimethyl-*p*-toluidin (DMT, 100 mM, 10.0 eq.), hexamethylcyclotrisiloxane (2 mM, 0.2 eq.) as the reference, and $[Cr(dqp)_2]^{3+}$ (200 µM, 2.0 mol%, blue solid line) or $[Ru(bpz)_3]^{2+}$ (80 µM, 0.8 mol%, green solid line), and in the absence of any photocatalyst (orange solid line) in air-saturated CD_3CN at room temperature, measured in 10 mm quartz cuvettes. At the irradiation wavelength of 415 nm, the two reaction mixtures containing Ru^{II} and Cr^{III} photocatalysts have identical absorbance. At this wavelength, the absorbance of 10 mM 2-bromoacetophenone accounts for ~ 1% of the total absorbance of the mixtures containing photocatalyst. Direct absorption of 415-nm light by 2-bromoacetophenone is therefore very limited when either 200 µM (2.0 mol%) $[Cr(dqp)_2]^{3+}$ or 80 µM (0.8 mol%) $[Ru(bpz)_3]^{2+}$ are present.

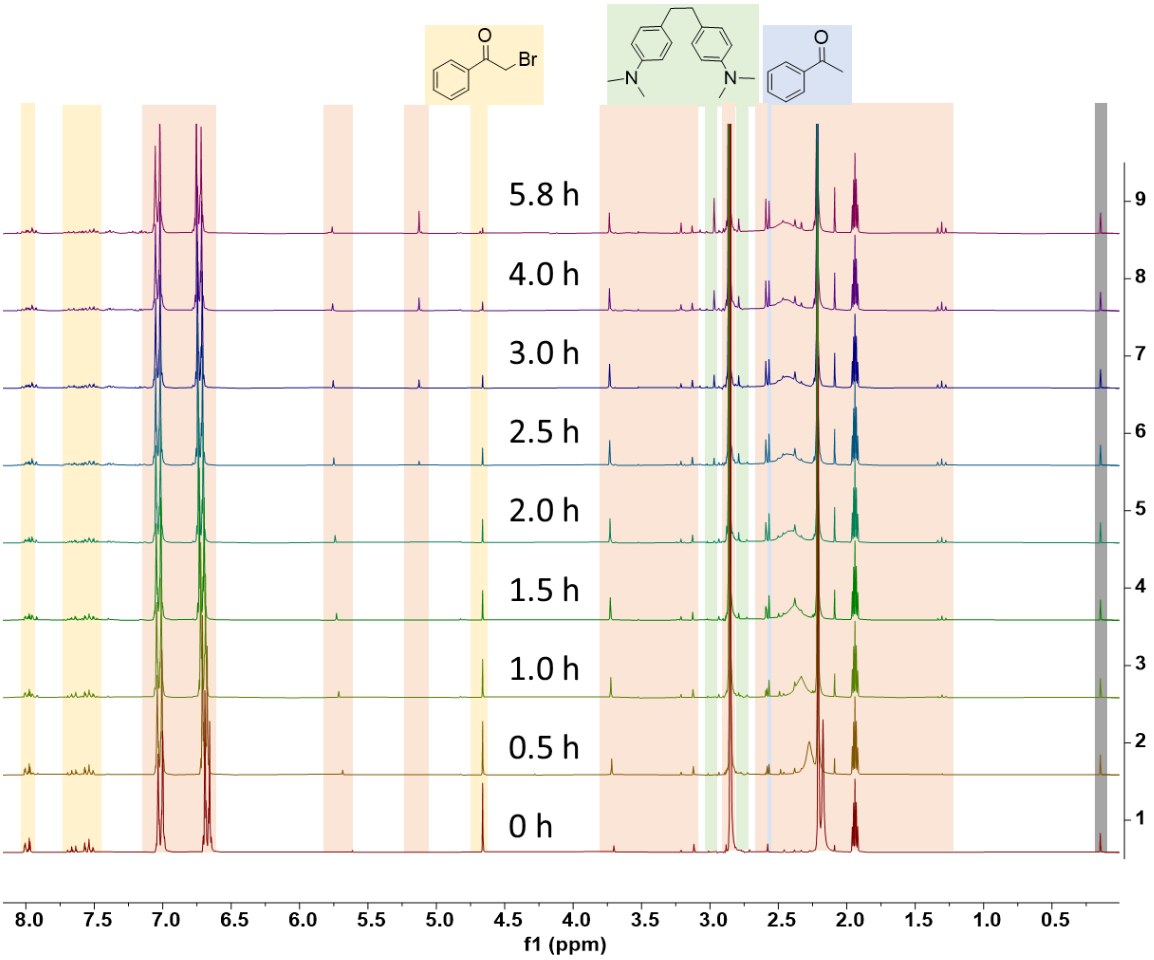

Supplementary Fig. 101: ^{1}H NMR spectra of the reaction mixture containing 2-bromoacetophenone (10 mM, 1.0 eq.), *N*,*N*-dimethyl-*p*-toluidine (DMT, 100 mM, 10.0 eq.), hexamethylcyclotrisiloxane (2 mM, 0.2 eq.) as the reference, and $[Ru(bpz)_3]^{2+}$ (80 µM, 0.8 mol%) in degassed CD_3CN. Resonances caused by the 2-bromoacetophenone starting material are marked by the

yellow shaded areas, the resonances marked in orange are assigned to DMT and solvent signals, the reference signals are marked in grey, the signals in the green marked area are attributable to the DMT dimer (1,2-bis(4-*N*,*N*-dimethylaminophenyl)ethane) according to the literature,[79] and the signals caused by the obtained acetophenone product are marked by the blue areas. For determination of the product yield, the integral of the proton signals at 0.2 ppm from the hexamethylsilane group of hexamethylcyclotrisiloxane was used as the internal reference. Product formation was verified by comparison to the ^{1}H-NMR spectrum of acetophenone in CD_3CN (Supplementary Fig. 121), in line with the literature.[80]

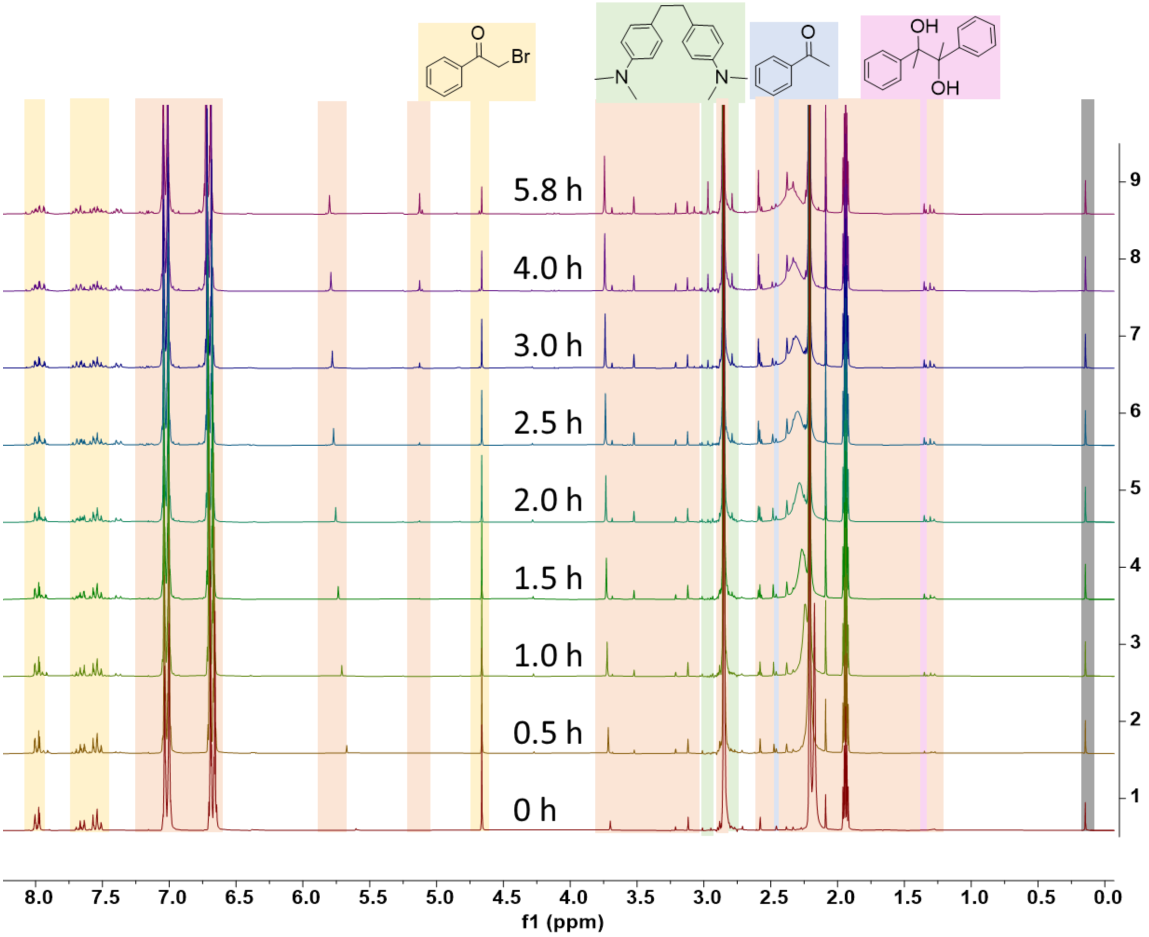

Supplementary Fig. 102: ^{1}H NMR spectra of the reaction mixture containing 2-bromoacetophenone (10 mM, 1.0 eq.), *N*,*N*-dimethyl-*p*-toluidine (DMT, 100 mM, 10.0 eq.), hexamethylcyclotrisiloxane (2 mM, 0.2 eq.) as the reference, and $[Cr(dqp)_2]^{3+}$ (200 µM, 2.0 mol%) in degassed CD_3CN. Resonances caused by the 2-bromoacetophenone starting material are marked by the yellow shaded areas, the resonances marked in orange are assigned to DMT and solvent signals, the reference signals are marked in grey, the signals in the green marked area are attributable to the DMT dimer (1,2-bis(4-*N*,*N*-dimethylaminophenyl)ethane) according to the literature,[79] the signals marked in the pink area are attributed to the pinacol coupling product (2,3-diphenyl-2,3-butanediol) as reported in the literature,[81] and the signals caused by the obtained acetophenone product are marked by the blue areas. For determination of the product yield, the integral of the proton signals at 0.2 ppm from the hexamethylsilane group of hexamethylcyclotrisiloxane was used as the internal reference. Product formation was verified by comparison to the ^{1}H-NMR spectrum of acetophenone in CD_3CN (Supplementary Fig. 121).[80]

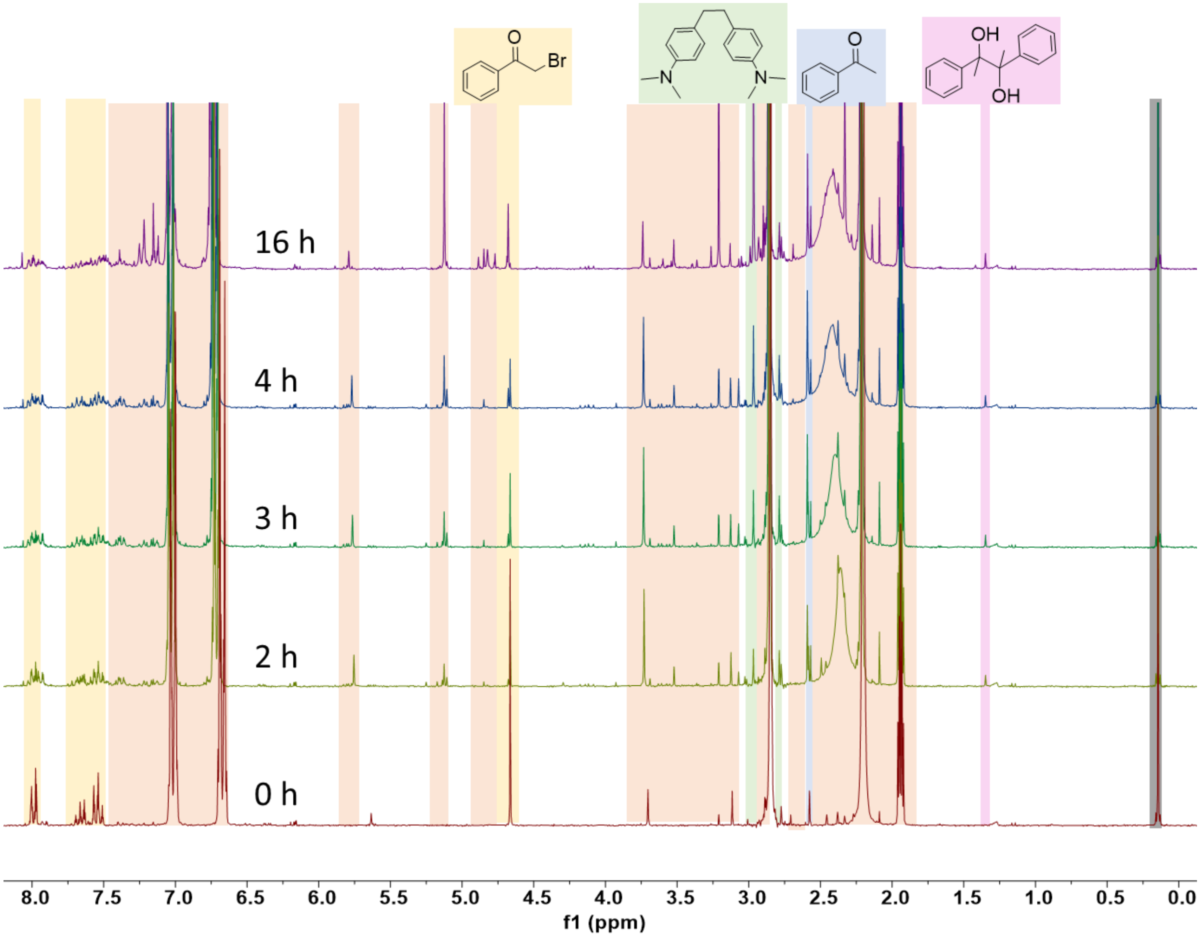

Supplementary Fig. 103*:* Control experiment performed in the absence of any photosensitizer: ^{1}H NMR spectra of the reaction mixture containing 2-bromoacetophenone (10 mM, 1.0 eq.), *N*,*N*-dimethyl-*p*-toluidine (DMT, 100 mM, 10.0 eq.), hexamethylcyclotrisiloxane (2 mM, 0.2 eq.) as the reference, in the absence of photocatalyst in degassed CD_3CN. Resonances caused by the 2-bromoacetophenone starting material are marked by the yellow shaded areas, the resonances marked in orange are assigned to DMT and solvent signals, the reference signals are marked in grey, the signals in the green marked area are attributable to the DMT dimer (1,2-bis(4-*N*,*N*-dimethylaminophenyl)ethane) according to the literature,[79] the signals marked in the pink area are attributed to the pinacol coupling product (2,3-diphenyl-2,3-butanediol) as reported in the literature,[81] and the signals caused by the obtained acetophenone product are marked by the blue areas. For determination of the product yield, the integral of the proton signals at 0.2 ppm from the hexamethylsilane group of hexamethylcyclotrisiloxane was used as the internal reference. Product formation was verified by comparison to the ^{1}H-NMR spectrum of acetophenone in CD_3CN (Supplementary Fig. 121).[80]

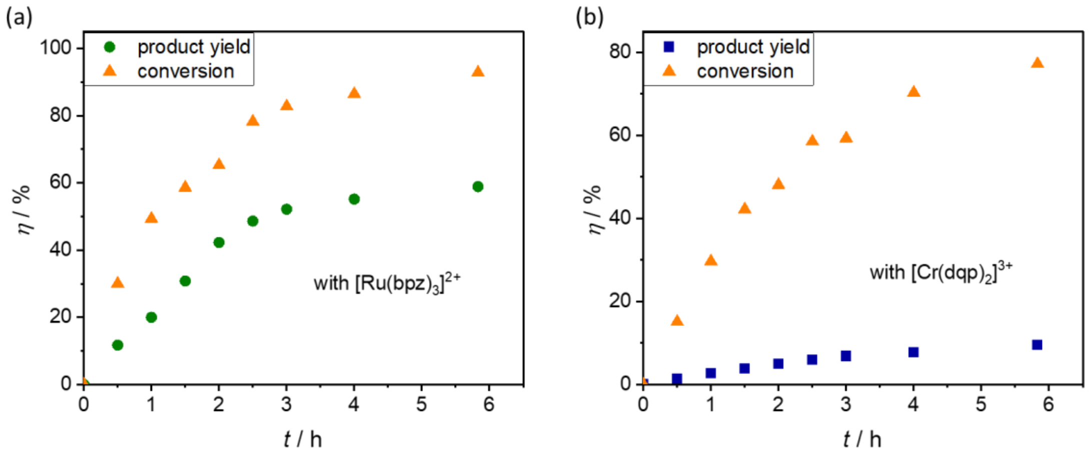

Supplementary Fig. 104: Acetophenone product yields and conversions of the photocatalytic reductive debromination of 2-bromoacetophenone catalyzed by (a) $[Ru(bpz)_3]^{2+}$ (0.8 mol%, product yields in green circles and conversions in orange

triangles, derived from Supplementary Fig. 101) and (b) $[Cr(dqp)_2]^{3+}$ (2.0 mol%, product yields in dark blue squares and conversions in orange triangles, derived from Supplementary Fig. 102) as a function of irradiation time (415 nm LED, 230 mW). In both cases, the product yields of acetophenone are substantially lower than the conversions of 2-bromoacetophenone during the irradiation, indicating that side reactions occur under these conditions (see discussion further below).

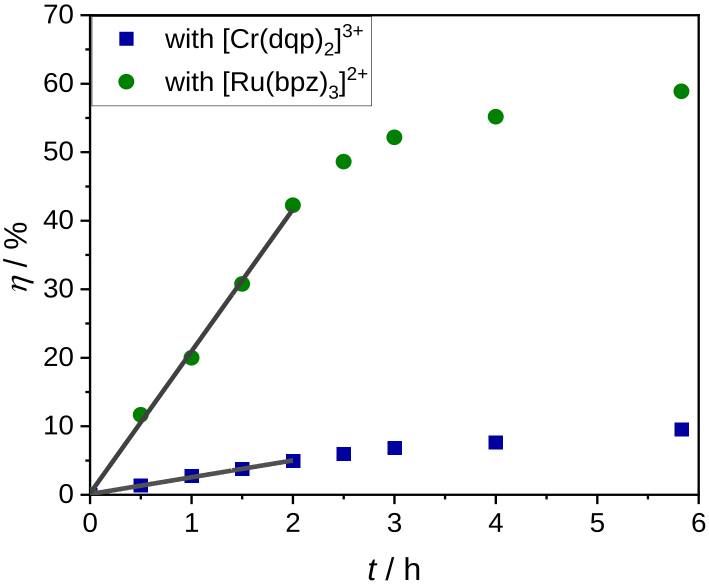

Supplementary Fig. 105: 2-Bromoacetophenone product yields of the photocatalytic reductive debromination catalyzed by $[Cr(dqp)_2]^{3+}$ (1.0 mol%, blue squares) and $[Ru(bpz)_3]^{2+}$ (0.4 mol%, green circles) as a function of irradiation time (415 nm LED, 230 mW). Linear fits (grey solid lines) of the product yields as a function of time in the initial irradiation period provide the initial product formation rates. These initial product formation rates (2.07 ± 0.06 mM h^{-1} for $[Ru(bpz)_3]^{2+}$ and 0.24 ± 0.01 mM for $[Cr(dqp)_2]^{3+}$) exhibit a ratio of $v(Ru^{II}) : v(Cr^{III})$ = 8.4 ± 0.5, approaching the cage escape quantum yields ratio $\Phi_{CE}(Ru^{II}) : \Phi_{CE}(Cr^{III})$ = 10.6 ± 1.3 obtained for the $[Ru(bpz)_3]^{2+}$ / DMT and $[Cr(dqp)_2]^{3+}$ / DMT donor-acceptor couples, Supplementary Table 1.

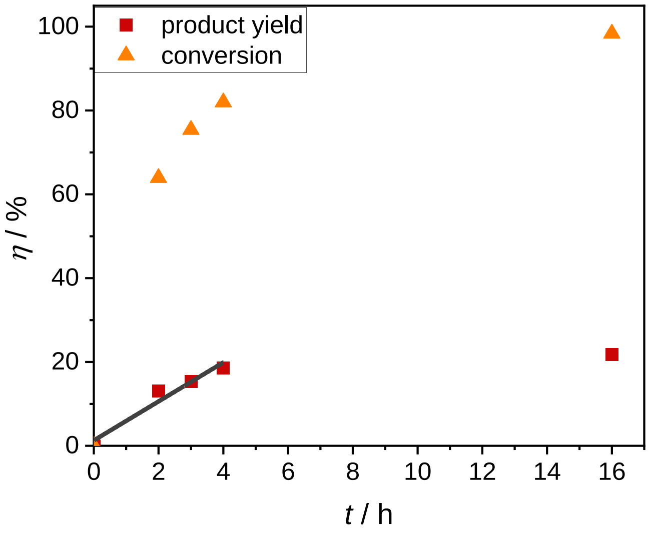

Supplementary Fig. 106: Product yield of acetophenone (red squares) and conversions of 2-bromoacetophenone (orange triangles) for the debromination of 2-bromoacetophenone performed in the absence of photocatalyst under blue light irradiation (415 nm LED, 230 mW) as a function of irradiation time. Based on the known initial substrate concentration (10 mM), a linear fit (grey solid line) to the observable product yield as a function of the initial reaction time (first 4 hours) provides an averaged product formation rate of = 0.47 ± 0.08 mM h^{-1}.

Debromination of α-bromoketones can be typically achieved by photoredox catalysis.[82-86] In the absence of photocatalyst, this reaction can occur according to a recent study, where the homolysis of the C–Br bond upon light irradiation (390 nm LED and 427 nm LED) was proposed as the mechanism, with solvent as the photoreductant.[87] One Einstein of 400-nm photons correspond to 71.5 kcal/mol, slightly exceeding the C–Br bond dissociation energy of 2-bromoacetophenone (65 ~ 68 kcal/mol),[88,89] making the reaction thermodynamically viable under these conditions. Indeed, in the absence of photocatalyst, the formation of acetophenone was observed, likely due to the very weak but detectable absorbance at wavelengths above 400 nm (Supplementary Fig. 100, inset).

In our experiments, the substrate conversion is clearly higher than the acetophenone product yields in all cases (Supplementary Fig. 104, Supplementary Fig. 106), indicating that side products other than acetophenone are formed. Considering the formed radical species upon homolysis of the C-Br bond and the presence of DMT and solvent,[87] various side reactions seem possible. In the presence of $[Ru(bpz)_3]^{2+}$ or $[Cr(dqp)_2]^{3+}$, lower product yields than substrate conversions were also observed, where the difference is more prominent for $[Cr(dqp)_2]^{3+}$ (Supplementary Fig. 104b), indicating the formation of substantial amounts of side products also when a photocatalyst is used. Pinacol coupling product[90] was observed when using $[Cr(dqp)_2]^{3+}$ as the catalyst (η = 9% after 6 hours irradiation, Supplementary Fig. 102) and in the absence of photocatalyst (η = 5% after 16 hours irradiation, Supplementary Fig. 103), but was not formed in a significant quantity with $[Ru(bpz)_3]^{2+}$ as the catalyst (Supplementary Fig. 101). Thus it seems that the pinacol coupling observed in the presence of $[Cr(dqp)_2]^{3+}$ can act as an additional reaction pathway for consuming 2-bromoacetophenone and/or the formed acetophenone, leading to a larger difference between the product yield and substrate conversion for $[Cr(dqp)_2]^{3+}$ than with $[Ru(bpz)_3]^{2+}$. Additionally, we also observed that the formed DMT radicals react with each other to form DMT dimers (Supplementary Fig. 101-S103), and it seem plausible that these DMT radical can also react with any acetophenone-derived radicals to give various side products, which could not be identified in the ^1H NMR spectra of the mixtures due to many overlapping resonances. The formation of all these side products, which are formed in multiple reaction pathways and to different extents in the cases of $[Ru(bpz)_3]^{2+}$ and $[Cr(dqp)_2]^{3+}$, makes the comparison of the kinetics for substrate conversion with $[Ru(bpz)_3]^{2+}$ and $[Cr(dqp)_2]^{3+}$ very challenging. Consequently, here we decided to focus on the kinetics of product formation.

In the comparative study of the photocatalytic debromination of 2-bromoacetophenone, the initial product formation rate was determined to be 2.07 ± 0.06 mM h^{-1} and 0.24 ± 0.01 mM h^{-1} for the $[Ru(bpz)_3]^{2+}$ and $[Cr(dqp)_2]^{3+}$ catalysts, respectively. The formation rate of acetophenone with $[Cr(dqp)_2]^{3+}$ present is thus even lower than the rate in the absence of any photocatalyst (0.47 ± 0.08 mM h^{-1}, Supplementary Fig. 106). Possibly, the presence of photocatalyst suppresses the background reaction, because the absorbance of the substrate at 415 nm amounts to only 1% of the overall absorbance in the presence of catalyst (Supplementary Fig. 100). In other words, 99% of the 415-nm light is absorbed by the photocatalyst, which largely (but not completely) suppresses the photoreaction that occurs in the absence of photocatalysts. Another fact that could account for this low product formation rate in the presence of $[Cr(dqp)_2]^{3+}$ is the very low cage escape quantum yield of (Φ_{CE} = 8 ± 1%) with DMT. Compared to $[Cr(dqp)_2]^{3+}$, the presence of $[Ru(bpz)_3]^{2+}$ accelerates the acetophenone formation by a factor of 8.4 ± 0.5, in good agreement with the cage escape quantum yield ratio of $\Phi_{CE}(Ru^{II}) : \Phi_{CE}(Cr^{III})$ = 10.6 ± 1.3 determined for the $[Ru(bpz)_3]^{2+}$ / DMT and $[Cr(dqp)_2]^{3+}$ / DMT donor-acceptor couples (Supplementary Table 1). Based on the electron transfer rate constants k_q in Supplementary Table 1 and the intrinsic lifetimes τ_0 of 565 ns for $[Ru(bpz)_3]^{2+}$ and 30 µs for $[Cr(dqp)_2]^{3+}$ in aerated acetonitrile at room temperature, the efficiency of photoinduced electron transfer Φ_{ET} in the presence of 0.1 M DMT can be estimated. The respective Φ_{ET} values are 99.85% for

$[Ru(bpz)_3]^{2+}$ and 100% for $[Cr(dqp)_2]^{3+}$. Thus, photoinduced electron transfer is quantitative in both cases and therefore is unlikely to be a performance-limiting factor for the overall photoredox reaction.

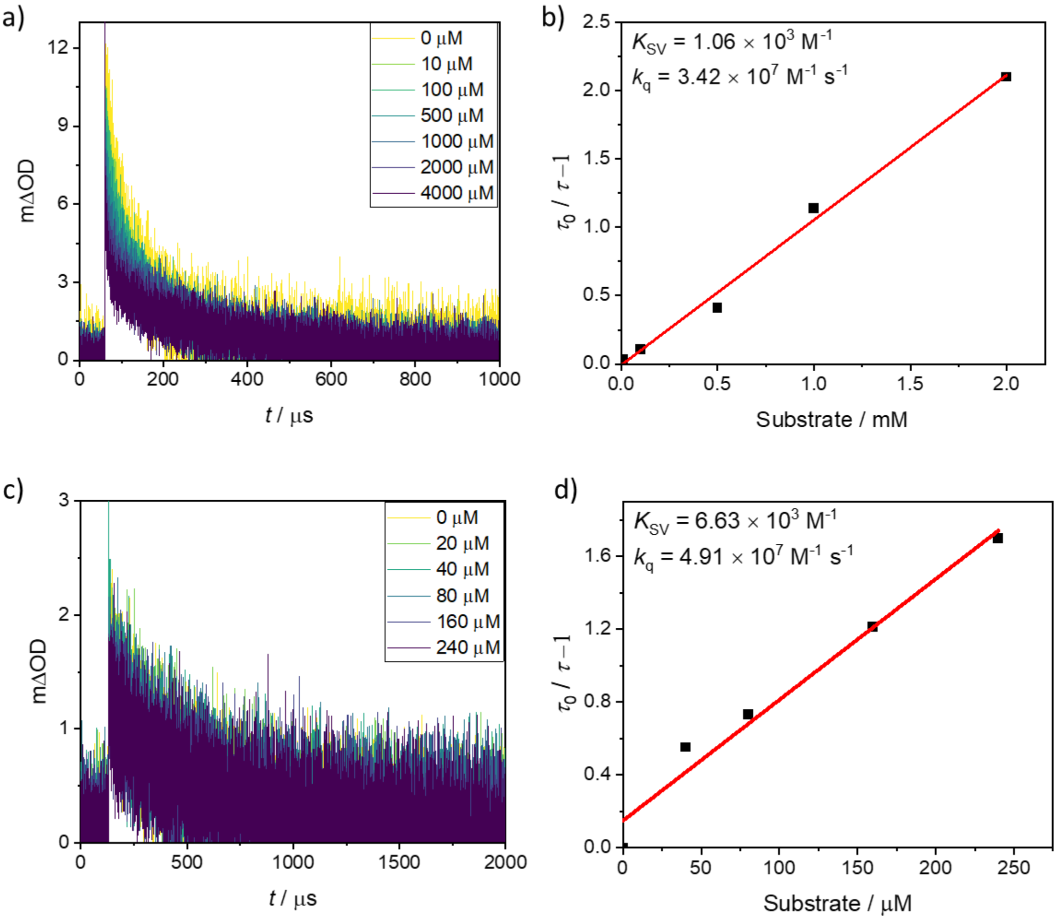

Supplementary Fig. 107: Stern-Volmer-type quenching studies of the one-electron reduced photocatalysts a)-b) $[Ru(bpz)_3]^+$ and c)-d) $[Cr(dqp)_2]^{2+}$ in deaerated CH_3CN at 20 °C with the substrate 2-bromoacetophenone; the concentration ratios of catalysts to DMT were kept the same as in the photochemical reactions (Supplementary Fig. 99a). a) Transient absorption decays of the $[Ru(bpz)_3]^{2+}$ (20 μM) / DMT (25 mM) couple at 414 nm (bleach signal caused by the formation of $[Ru(bpz)_3]^+$, without strong spectral contribution of $DMT^{•+}$, resulting from electron transfer as shown in Supplementary Fig. 52; signals multiplied by a factor of -1) with increasing concentration of the substrate under excitation with a ~10 ns pulsed 415 nm laser (pulse energy ~6 mJ); bi-exponential fits of the decays yield a short lifetime τ_1 and a long lifetime τ_2 (detailed discussion follows below). (b) Linear Stern-Volmer plot based on the short lifetime τ_1 derived from the decays in (a). c) Transient absorption decays of the $[Cr(dqp)_2]^{3+}$ (50 μM) / DMT (25 mM) couple at 380 nm (bleach signal caused by the formation of $[Cr(dqp)_2]^{2+}$ (Supplementary Fig. 43), without strong spectral contribution of $DMT^{•+}$, resulting from electron transfer; signals multiplied by a factor of -1) with increasing concentration of the substrate under excitation with a ~10 ns pulsed 415 nm laser (pulse energy ~6 mJ). Due to the very low cage escape quantum yield of the $[Cr(dqp)_2]^{3+}$ / DMT pair (Φ_{CE} = 8 ± 1%, ten times lower than the $[Ru(bpz)_3]^{2+}$ / DMT pair, Supplementary Table 1), the signal to noise ratio of the transient absorption signals of the photoproducts upon electron transfer is very low. Bi-exponential fits of the decays yield a short lifetime τ_1 and a long lifetime τ_2 (detailed discussion see below). d) Linear Stern-Volmer plot based on the short lifetime τ_1 derived from the decays in (c).

Supplementary Table 6: Summary of bi-exponential fitting results of transient absorption decays of the $[Ru(bpz)_3]^{2+}$ / DMT couple at 414 nm (Supplementary Fig. 107a) and the $[Cr(dqp)_2]^{3+}$ / DMT couple at 380 nm (Supplementary Fig. 107c) in the absence and in the presence of various concentrations of the substrate 2-bromoacetophenone. The low signal to noise ratio obtained for the $[Cr(dqp)_2]^{3+}$ / DMT in this experiment (Supplementary Fig. 107c) does not allow for equally good fitting results as in the case of the $[Ru(bpz)_3]^{2+}$ / DMT couple.

Substrate / µM	τ_1	A_1	τ_2	A_2
	$[Ru(bpz)_3]^{2+}$ / DMT, transient absorption decay at 414 nm			
0	31	0.51	149	0.49
10	30	0.53	170	0.47
100	28	0.50	184	0.50
500	23	0.41	183	0.59
1000	15	0.46	204	0.54
2000	10	0.52	190	0.48
	$[Cr(dqp)_2]^{3+}$ / DMT, transient absorption decay at 380 nm			
0	135	0.37	536	0.63
40	87	0.25	545	0.75
80	78	0.12	355	0.88
160	61	0.18	467	0.82
240	50	0.14	500	0.86

Quenching studies of the one-electron reduced catalysts with the substrate provided a closer insight into the kinetics of this reaction step (Supplementary Fig. 107). For the $[Ru(bpz)_3]^{2+}$ / DMT couple, the transient absorption signal recorded at 414 nm arises almost exclusively from $[Ru(bpz)_3]^{+}$ formed upon photoinduced electron transfer (Supplementary Fig. 52), whereas $DMT^{•+}$ has comparatively little absorption at that wavelength (analogously to DMA-$OMe^{•+}$ in Supplementary Fig. 13). In the absence of the substrate, the transient absorption signal at 414 nm does not decay in single-exponential manner because the bimolecular diffusion-controlled back electron transfer with $DMT^{•+}$ follows a second-order reaction. In the presence of increasing amount of substrate, electron transfer from $[Ru(bpz)_3]^{+}$ to the substrate can occur, and the transient absorption decays at 414 nm become faster (Supplementary Fig. 107a). The changes in decay kinetics in the absence and in the presence of substrate at variable concentrations are thus a consequence of the reaction between $[Ru(bpz)_3]^{+}$ and the substrate, which follows a pseudo-first order reaction rate law with excess substrate. Given that the reactions of $[Ru(bpz)_3]^{+}$ with $DMT^{•+}$ and with the substrate occur in a competing manner and follow different rate laws (second order vs pseudo-first order), the analysis of the resulting decay kinetics is not straightforward. It seemed plausible to use a bi-exponential fitting procedure, which yielded a short-lived decay component (τ_1) that was markedly dependent on the concentration of the substrate, whereas the longer-lived decay component (τ_2) showed no obvious trend with increasing solvent concentration (Supplementary Table 6). Consequently, the decrease in τ_1 with increasing substrate concentration was attributed to the reaction of $[Ru(bpz)_3]^{+}$ with the substrate, whereas the long-lived component (τ_2) is likely dominated by the reaction with $DMT^{•+}$. A linear Stern-Volmer type plot of τ_1 can therefore be used to estimate the kinetics of the reaction between $Ru(bpz)_3]^{+}$ and the substrate (Supplementary Fig. 107b), leading to a rate constant of $3.42 \times 10^{7}\ M^{-1}\ s^{-1}$.

Analogous analysis was done for the transient absorption decays at 380 nm obtained for the $[Cr(dqp)_2]^{3+}$ / DMT couple (Supplementary Table 6), giving a rate constant of $4.91 \times 10^{7}\ M^{-1}\ s^{-1}$ for the reaction of $[Cr(dqp)_2]^{2+}$ with the substrate (Supplementary Fig. 107c/d). Initial attempts to use identical substrate concentrations for both $[Ru(bpz)_3]^{2+}$ and $[Cr(dqp)_2]^{3+}$ photosensitizers gave even lower signal to noise ratios for the Cr^{III} complex, which we tentatively attribute to the formation of photochemical products, for example the radical anion of acetophenone. The disappearance of the one-electron reduced Cr complex is monitored by a bleach at 380 nm, which does not give a very strong signal in

the first place (largely owed to low cage escape quantum yield), and where the acetophenone radical anion and related species absorb.[91] To limit the concentration of such photoproducts formed, a lower substrate concentration than with the Ru photosensitizer seemed meaningful. The one-electron reduced form of the Ru^{II} photosensitizer is monitored by an easier-to-detect signal at 450 nm (largely owed to the substantially higher cage escape quantum yield), and it seems that photoproduct absorption interferes less strongly in this case. The decays monitored for Cr^{III} at 380 nm are therefore likely a complicated function of different processes involving at least (i) reaction of one-electron reduced Cr^{III} complex with the substrate, (ii) reverse electron transfer between one-electron reduced Cr^{III} complex with DMT radical cation, and (iii) decay of the abovementioned acetophenone radical anion and related absorbing species. The bi-exponential fits are therefore a simplistic approach, as mentioned further above, and the meaning of the weighting factors A_1 and A_2 values should not be over-interpreted.

The similar rate constants obtained with $[Ru(bpz)_3]^{2+}$ and $[Cr(dqp)_2]^{3+}$ suggest that the elementary step of onward electron transfer from the reduced photosensitizers to the substrate is not responsible for the observable difference in overall product formation rates between Ru^{II} and Cr^{III} complexes in the photocatalysis experiments, at least within the limits of this experiment, dictated largely by the experimental uncertainty associated with the Cr^{III} experiments.

8.3 Photocatalytic Aza-Henry reaction

2-Phenyl-1,2,3,4-tetrahydroisoquinoline (THIQ, 50 mM, 1.0 eq.), nitromethane (250 mM, 5.0 eq.), and hexamethylcyclotrisiloxane (2 mM, 0.2 eq.) as the internal standard were dissolved in air-saturated CD_3CN as a stock solution. The photocatalysts (PC) $[Cr(dqp)_2]^{3+}$ (345 µg, 500 µM, 1.0 mol%) and $[Ru(bpz)_3]^{2+}$ (104 µg, 200 µM, 0.4 mol%) were dissolved in 600 µL of the stock solution, respectively. After ^{1}H-NMR measurements of the freshly prepared samples, the NMR tubes were irradiated at room temperature with a 415 nm LED (230 mW) equipped with a 400 nm long pass filter. A plausible reaction mechanism, similar as proposed previously, is shown in Supplementary Fig. 108b/c.[92,93]

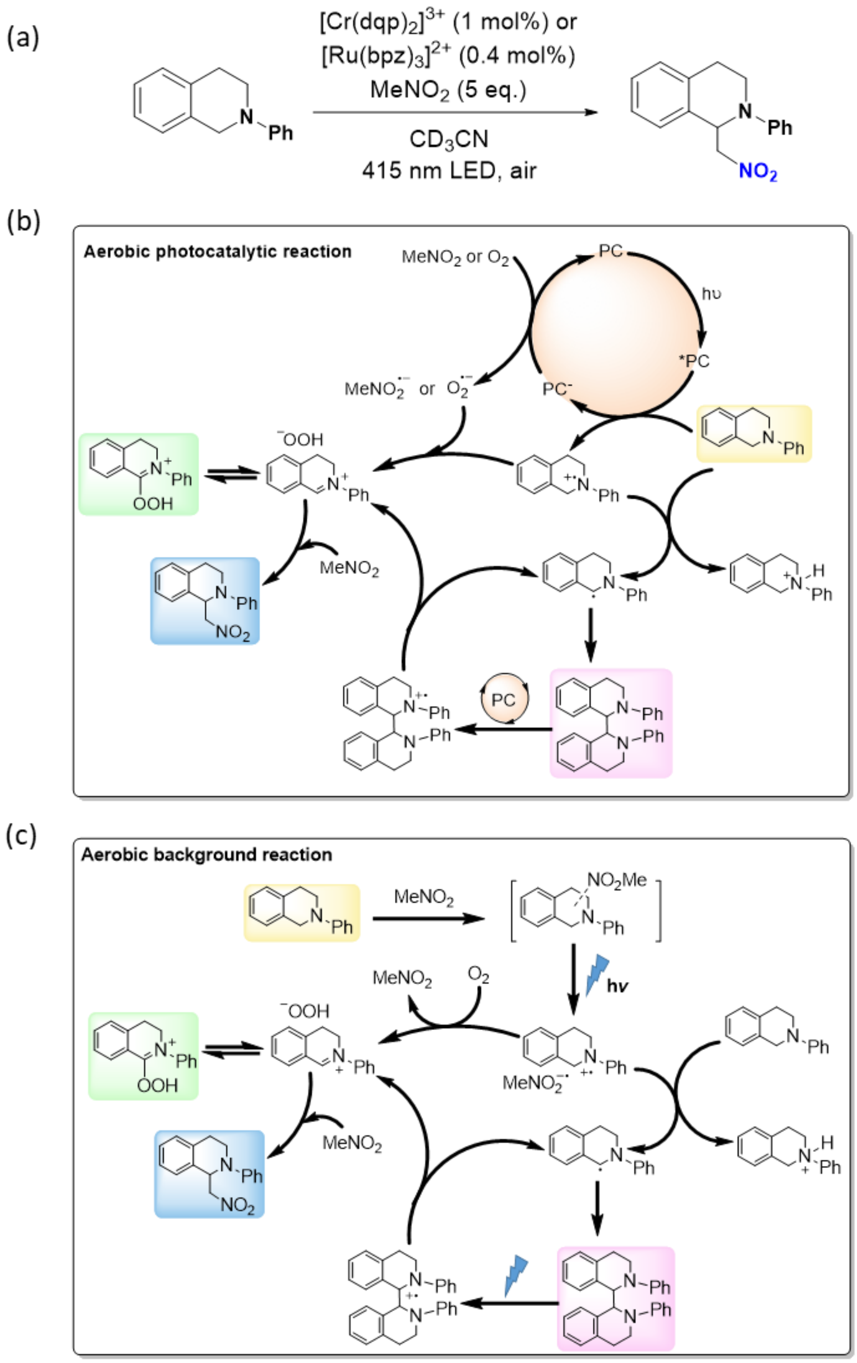

Supplementary Fig. 108: (a) Photocatalytic Aza-Henry reaction. 2-Phenyl-1,2,3,4-tetrahydroisoquinoline (THIQ, 50 mM, 1.0 eq.), nitromethane ($MeNO_2$, 250 mM, 5.0 eq.), and hexamethylcyclotrisiloxane (10 mM, 0.2 eq.) as the internal standard were dissolved in air-saturated CD_3CN. Either $[Cr(dqp)_2]^{3+}$ (345 µg, 500 µM, 1.0 mol%) or $[Ru(bpz)_3]^{2+}$ (104 µg, 200 µM, 0.4 mol%) was used as the photocatalyst (PC). Irradiation occurred with a 415 nm LED (230 mW) at room temperature. (b) Photocatalytic Aza-Henry reaction mechanism as proposed previously for different photocatalysts.[92,93] (c) Background reaction mechanism under blue light irradiation in the absence of PC derived from a previous investigation.[93]

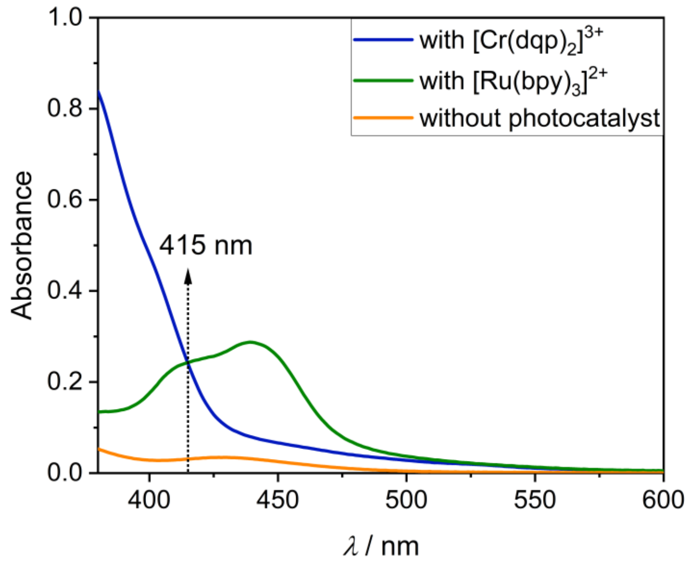

Supplementary Fig. 109: UV/vis absorption spectra of the reaction mixtures containing 2-phenyl-1,2,3,4-tetrahydroisoquinoline (THIQ, 50 mM, 1.0 eq.), nitromethane (250 mM, 5.0 eq.), hexamethylcyclotrisiloxane (10 mM, 0.2 eq.) in the presence of $[Cr(dqp)_2]^{3+}$ (500 µM, 1.0 mol%, blue solid line) or $[Ru(bpz)_3]^{2+}$ (200 µM, 0.4 mol%, green solid line), and in the absence of any photocatalyst (orange solid line) in air-saturated CD_3CN at room temperature, measured in 1 mm quartz cuvettes. At the irradiation wavelength of 415 nm, the two reaction mixtures containing Ru^{II} and Cr^{III} photocatalysts have identical absorbance. At this wavelength, the absorbance of 50 mM THIQ accounts for ~ 13% of the total absorbance of the mixtures containing photocatalyst.

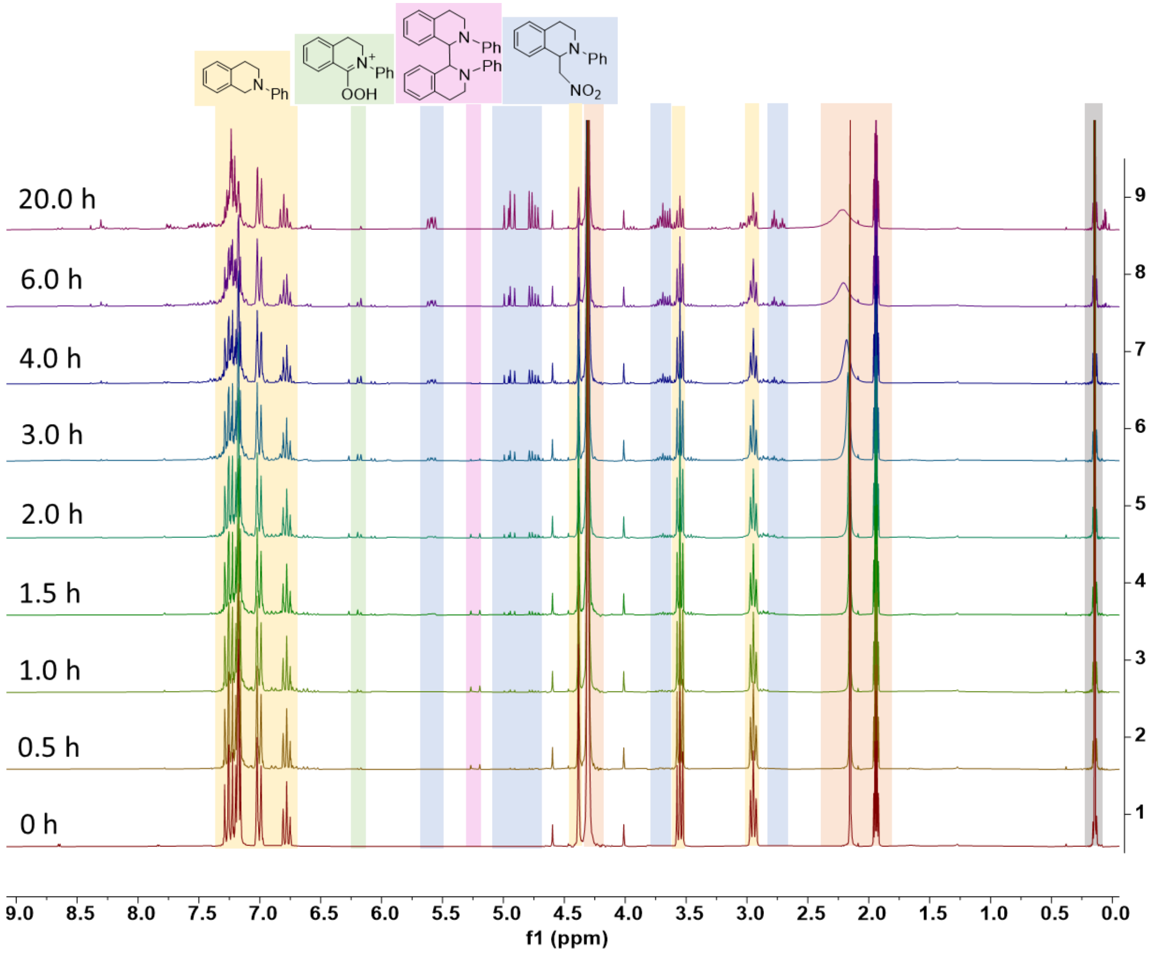

Supplementary Fig. 110: ^{1}H NMR spectra of the reacting mixture containing 2-phenyl-1,2,3,4-tetrahydroisoquinoline (THIQ, 50 mM, 1.0 eq.), nitromethane ($MeNO_2$, 250 mM, 5.0 eq.), and hexamethylcyclotrisiloxane (2 mM, 0.2 eq.) as the internal reference, and $[Ru(bpz)_3]^{2+}$ (200 µM, 0.4 mol%) in air-saturated CD_3CN. Resonances due to the THIQ starting material are marked by the yellow shaded areas, the signals marked in orange are assigned to nitromethane and solvent signals, the reference signals are marked in grey, the signals marked in pink and green are assigned to the THIQ dimer and the THIQ hydroperoxide (THIQ-OOH) according to the literature,[93] and the resonances attributable to the THIQ-CH_2NO_2 product are marked by the blue areas. For the determination of the product yield, the integral of the proton resonances at 0.2 ppm caused by the hexamethylsilane group of hexamethylcyclotrisiloxane was used as the internal reference. Product formation was verified by comparison to the ^{1}H-NMR spectrum of the product in CD_3CN, as reported in the literature.[93]

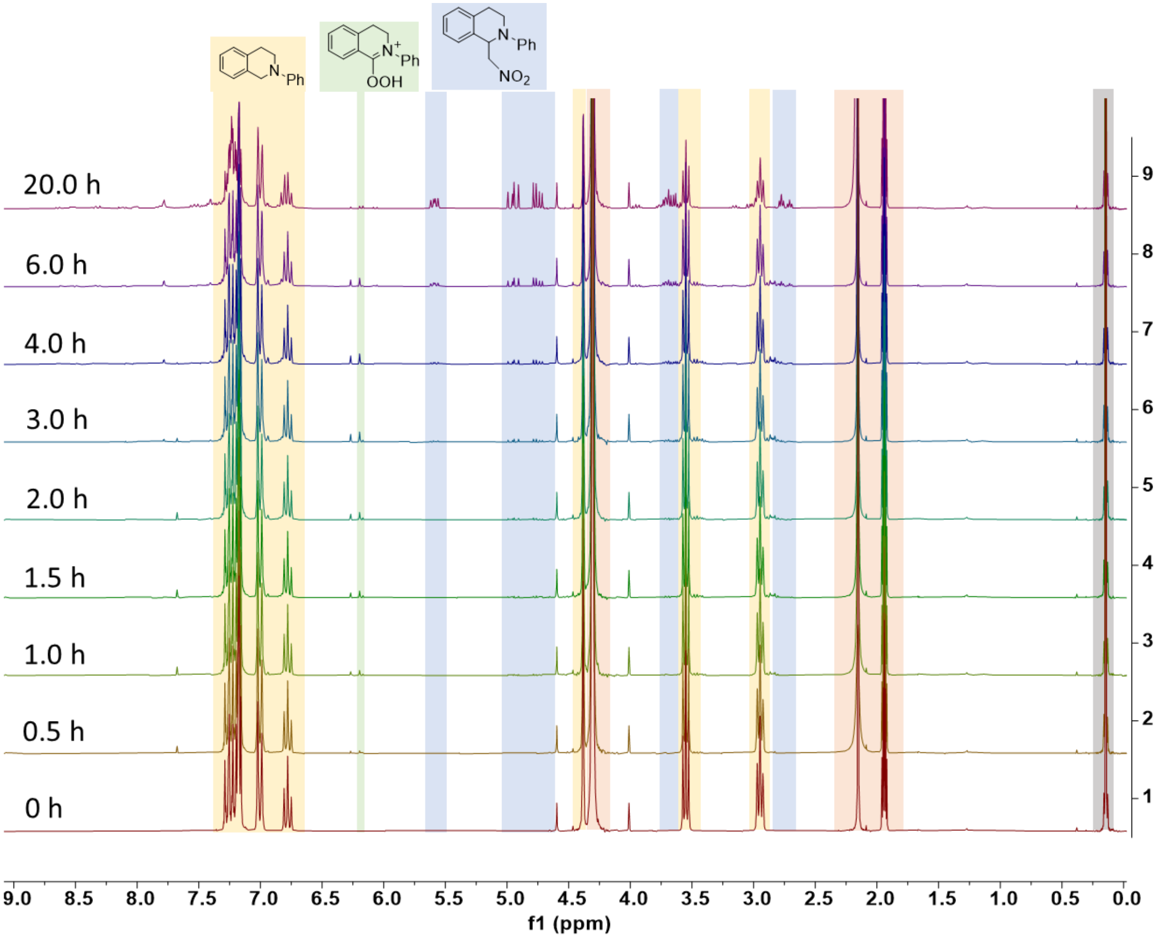

Supplementary Fig. 111: ^{1}H NMR spectra of the reacting mixture containing 2-phenyl-1,2,3,4-tetrahydroisoquinoline (THIQ, 50 mM, 1.0 eq.), nitromethane ($MeNO_2$, 250 mM, 5.0 eq.), and hexamethylcyclotrisiloxane (2 mM, 0.2 eq.) as the internal reference, and $[Cr(dqp)_2]^{3+}$ (500 µM, 1.0 mol%) in air-saturated CD_3CN. Resonances due to the THIQ starting material are marked by the yellow shaded areas, the signals marked in orange are assigned to nitromethane and solvent signals, the reference signals are marked in grey, the signals marked in pink and green are assigned to the THIQ dimer and the THIQ hydroperoxide (THIQ-OOH) according to the literature,[93] and the resonances attributable to the THIQ-CH_2NO_2 product are marked by the blue areas. For the determination of the product yield, the integral of the proton resonances at 0.2 ppm caused by the hexamethylsilane group of hexamethylcyclotrisiloxane was used as the internal reference. Product formation was verified by comparison to the ^{1}H-NMR spectrum of the product in CD_3CN, as reported in the literature.[93]

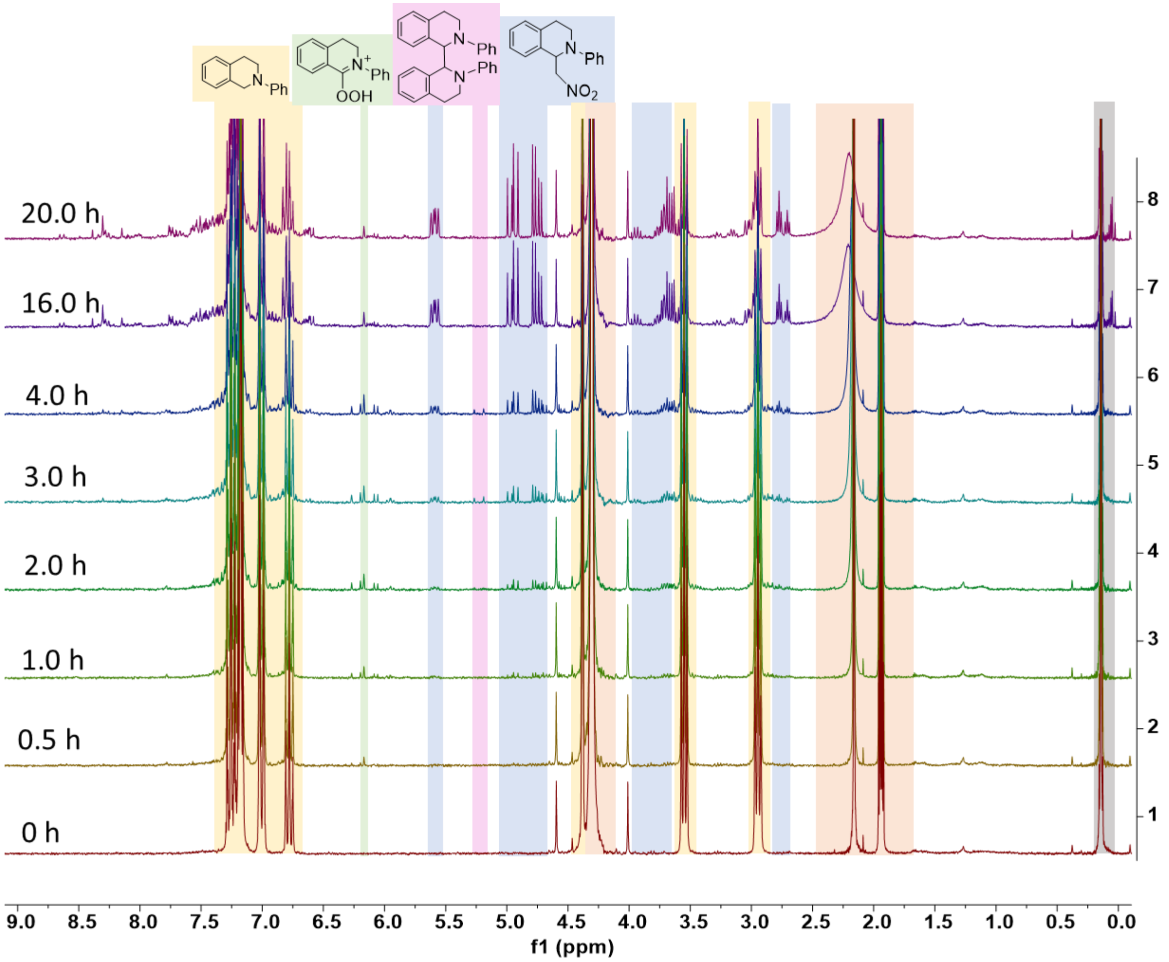

Supplementary Fig. 112: Control experiment performed in the absence of any photosensitizer: ^{1}H NMR spectra of the reacting mixture containing 2-phenyl-1,2,3,4-tetrahydroisoquinoline (THIQ, 50 mM, 1.0 eq.), nitromethane (250 mM, 5.0 eq.), and hexamethylcyclotrisiloxane (2 mM, 0.2 eq.) as the internal reference, in the absence of photocatalyst. Resonances due to the THIQ starting material are marked by the yellow shaded areas, the signals marked in orange are assigned to nitromethane and solvent signals, the reference signals are marked in grey, the signals marked in pink and green are assigned to the THIQ dimer and the THIQ hydroperoxide (THIQ-OOH) according to the literature,[93] and the resonances attributable to the THIQ-CH_2NO_2 product are marked by the blue areas. For the determination of the product yield, the integral of the proton resonances at 0.2 ppm caused by the hexamethylsilane group of hexamethylcyclotrisiloxane was used as the internal reference. Product formation was verified by comparison to the ^{1}H-NMR spectrum of the product in CD_3CN, as reported in the literature.[93]

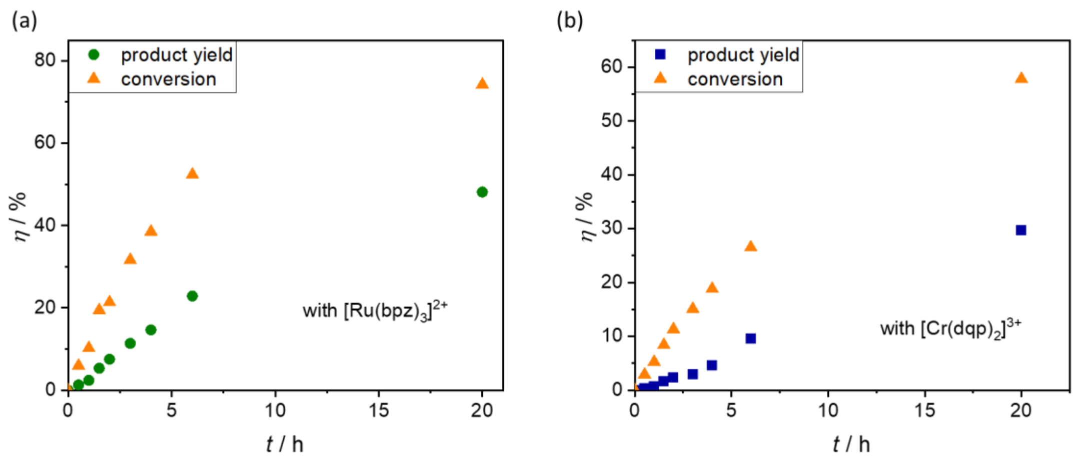

Supplementary Fig. 113: Product yields and conversions for the photocatalytic Aza-Henry reaction catalyzed by (a) $[Ru(bpz)_3]^{2+}$ (0.4 mol%, product yields in green circles and conversions in orange triangles, derived from Supplementary Fig. 110) and (b) $[Cr(dqp)_2]^{3+}$ (1.0 mol%, product yields in dark blue squares and conversions in orange triangles, derived from Supplementary Fig. 111) as a function of irradiation time (415 nm LED, 230 mW). In both cases, the product yields are substantially lower than the conversion of the THIQ substrate, indicating that significant amounts of side products are formed under these conditions (see discussion further below).

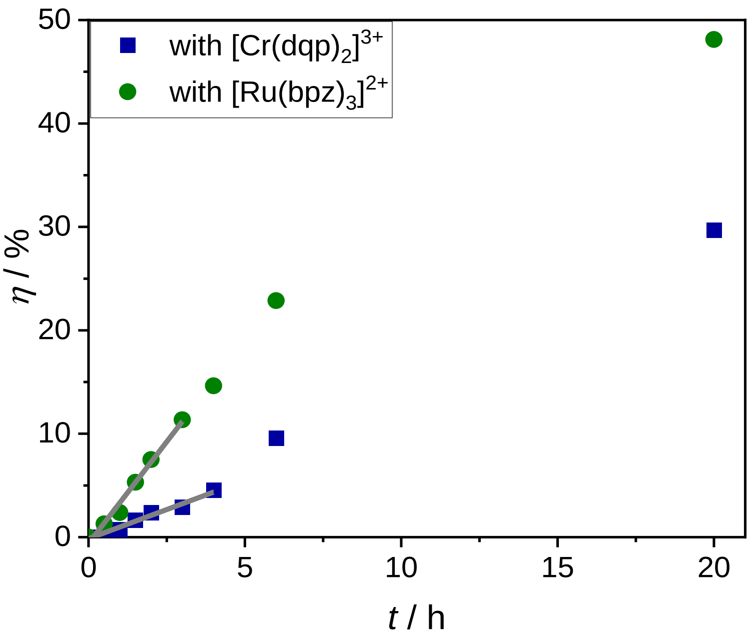

Supplementary Fig. 114: Product yields for the photocatalytic Aza-Henry reaction catalyzed by $[Cr(dqp)_2]^{3+}$ (1.0 mol%, dark blue squares) and $[Ru(bpz)_3]^{2+}$ (0.4 mol%, green circles) as a function of irradiation time (415 nm LED, 230 mW). Linear fits (grey solid lines) to the observable product yields as a function of time yield product formation rates of 1.97 ± 0.12 mM h^{-1} and 0.56 ± 0.04 mM h^{-1} for $[Ru(bpz)_3]^{2+}$ and $[Cr(dqp)_2]^{3+}$, respectively, corresponding to a rate ratio of 3.5 ± 0.3. For the reaction catalyzed by $[Ru(bpz)_3]^{2+}$, the additional reaction pathway of forming the THIQ dimer in the first four hours of irradiation can potentially affect the product formation rate (Supplementary Fig. 110) (see discussion below).

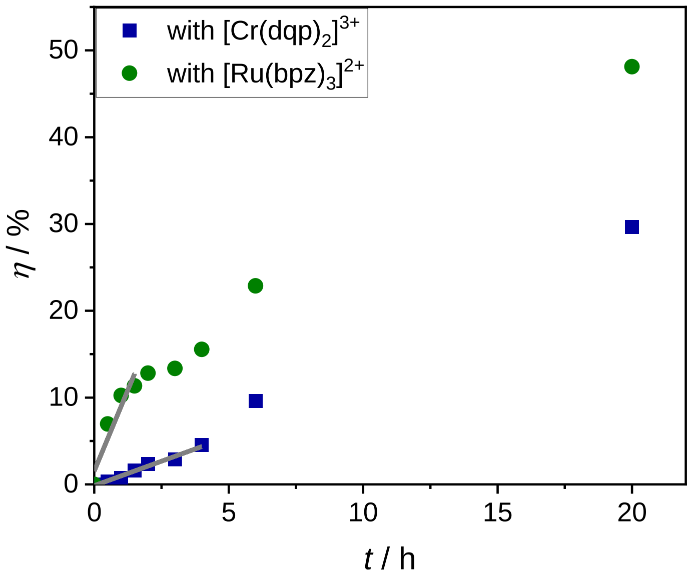

Supplementary Fig. 115: Summed yields of the THIQ-CH_2NO_2 main product and the THIQ dimer side product formed in the photocatalytic Aza-Henry reaction catalyzed by $[Cr(dqp)_2]^{3+}$ (1.0 mol%, dark blue squares) and $[Ru(bpz)_3]^{2+}$ (0.4 mol%, green circles) as a function of irradiation time (415 nm LED, 230 mW). Linear fits (grey solid lines) to the experimentally determined yields as a function of irradiation time give reaction rates of 3.71 ± 0.93 mM h^{-1} and 0.56 ± 0.04 mM h^{-1} for $[Ru(bpz)_3]^{2+}$ and $[Cr(dqp)_2]^{3+}$, respectively, corresponding to a rate ratio of 6.6 ± 1.7. For the reaction catalyzed by $[Ru(bpz)_3]^{2+}$, the additional reaction pathway of forming the THIQ dimer, observed mainly in the first four hours of irradiation (Supplementary Fig. 110) affects the overall product formation rate (see discussion below).

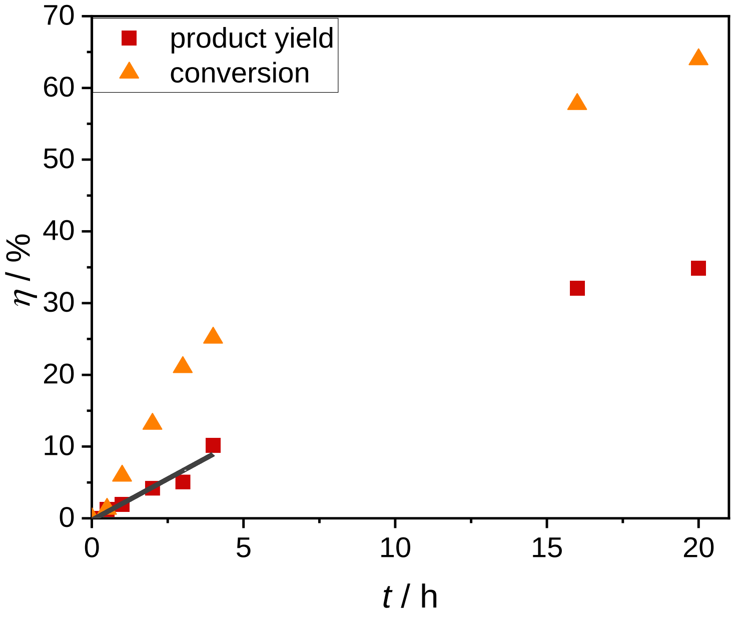

Supplementary Fig. 116: Product yields for the Aza-Henry reaction (red squares) and the conversions of THIQ (orange triangles) performed in the absence of photocatalyst under blue light irradiation (415 nm LED, 230 mW) as a function of irradiation time. Based on the known initial substrate concentration (50 mM), a linear fit (grey solid lines) to the observable product yields as a function of irradiation time provides a product formation rate of 1.15 ± 0.15 mM h^{-1}.

A detailed mechanistic study of the photocatalyzed Aza-Henry reaction and light-driven background reactions has been reported earlier.[93] In the absence of photocatalyst, THIQ can indeed react with

nitromethane under blue light irradiation, forming the desired product (Supplementary Fig. 108c). Our own investigations of the reaction kinetics in the absence of photocatalyst with ^{1}H NMR spectroscopy indicate the formation of the THIQ-OOH product (Supplementary Fig. 112, green areas), in line with the abovementioned previous mechanistic study.[93] Additionally, we observed the characteristic NMR resonances of the THIQ dimer after 3 hours of irradiation (Supplementary Fig. 112, pink area), which disappear again at even longer irradiation times. It seems that blue light can also be absorbed directly by the THIQ dimer, yielding a THIQ dimer radical cation, which can further decompose to an iminium cation and a THIQ α-amino alkyl radical. Compared to the conversion of THIQ, the product yields are substantially lower (Supplementary Fig. 116). The formation of the THIQ dimer and THIQ-OOH (Supplementary Fig. 112) can likely account for this behavior. This phenomenon was also observed in the presence of $[Ru(bpz)_3]^{2+}$ or $[Cr(dqp)_2]^{3+}$ catalyst (Supplementary Fig. 113), where side products are marked in the respective ^{1}H NMR spectra (Supplementary Fig. 110 and Supplementary Fig. 111). Hereby, we continue to focus on the kinetics of product formation instead of substrate conversion due to the formation of various side products, similarly to the photocatalytic debromination reaction discussed above.

Following the reaction kinetics as a function of time results in an initial product formation rate of 1.15 ± 0.15 mM h^{-1} for the formation of the Aza-Henry photoproduct in the absence of any photocatalyst under the conditions applied here. For the comparative study of the photocatalytic Aza-Henry reaction, the product formation rate was determined to be 1.97 ± 0.12 mM h^{-1} and 0.56 ± 0.04 mM h^{-1} for the $[Ru(bpz)_3]^{2+}$ and $[Cr(dqp)_2]^{3+}$ catalysts, respectively. Notably, the initial rate for product formation with $[Cr(dqp)_2]^{3+}$ catalyst is lower than the background rate determined in its absence. Evidently, $[Cr(dqp)_2]^{3+}$ is not accelerating in the photoreaction, but instead decelerates the reaction, likely due to dominant absorption of the 415-nm excitation light by $[Cr(dqp)_2]^{3+}$ (Supplementary Fig. 109) and the very low cage escape quantum yield of (Φ_{CE} < 7%) with THIQ. In comparison, the presence of $[Ru(bpz)_3]^{2+}$ accelerates the reaction by a factor of 3.5 ± 0.3 with respect to the reaction performed in the presence of $[Cr(dqp)_2]^{3+}$. This factor is somewhat lower than the cage escape quantum yield ratio of $\Phi_{CE}(Ru^{II}) : \Phi_{CE}(Cr^{III}) > 5.0$ determined for the $[Ru(bpz)_3]^{2+}$ / THIQ and $[Cr(dqp)_2]^{3+}$ / THIQ donor-acceptor couples (Supplementary Table 1). In the presence of $[Ru(bpz)_3]^{2+}$, the additional reaction pathway of forming the THIQ dimer (Supplementary Fig. 110), which was not observed in the case of $[Cr(dqp)_2]^{3+}$, might lead to a somewhat lowered Aza-Henry product formation rate with $[Ru(bpz)_3]^{2+}$. This could potentially account for the observation that the product formation rate ratio (~3.5) is lower than the cage escape quantum yield ratio (> 5.0). Indeed, with $[Ru(bpz)_3]^{2+}$ the summed reaction rates for forming the anticipated main product and the THIQ dimer side product exceeds the reaction rate with $[Cr(dqp)_2]^{3+}$ by a factor of 6.6 ± 1.7 (Supplementary Fig. 115), in line with the cage escape quantum yield ratio of $\Phi_{CE}(Ru^{II}) : \Phi_{CE}(Cr^{III}) > 5.0$. Similar to the other two reactions, near quantitative electron transfer efficiencies Φ_{ET} of 99.45% and 99.90% were obtained for $[Ru(bpz)_3]^{2+}$ and $[Cr(dqp)_2]^{3+}$, respectively, with 10 mM THIQ in the reaction mixture. Therefore, photoinduced electron transfer is unlikely to be a performance-limiting factor for the overall photoredox reaction.

9. Determination of the quantum yields Φ_P for photoproduct formation

The quantum yields for the formation of the photoproducts Φ_P of the individual reactions were determined using the following equation **eq. 8**:[94]

$$\Phi_P = \frac{N_P}{N_{abs}} \qquad \textbf{eq. 8}$$

where N_P is the number of formed product molecules per second and N_{abs} indicates the number of photons absorbed per second.

From the number of formed product molecules per second N_P, the product yield η can be derived from **eq. 9**, in which t is the reaction time, $c[S]_0$ is the initial concentration of the substrate at the beginning of the reaction, V is the volume of the sample (0.6 mL), and N_A is Avogadro's constant.

$$N_P = \frac{\eta \times c[S]_0 \times V \times N_A}{t} \qquad \textbf{eq. 9}$$

For determination of the number of the absorbed photons by the sample, the 415 nm illumination power density at the sample position, the light reflections caused by the glass beaker for water cooling bath and the NMR tube glass, and the absorbance of the samples need to be considered (Supplementary Fig. 88). Absorption by the water bath at 415 nm is negligible.[95] The power of the used 415 nm LED equipped with a 400 nm long pass filter at the sample position was measured with a power meter (COHERENT, Field MaxII-TOP Laser Power and Energy Meter). The size and shape of the light beam at the sample position was determined with a laser beam profiler (Newport, BM-USB3-SP932U). The power density was calculated to be 73 mW cm^{-2}. The irradiation area on the NMR tube was measured to be 1.48 cm^2. Light reflections on the glass surfaces cause ca. 20% energy losses as determined with the power meter. Thus, the power irradiated on the samples was calculated to be 86 mW, corresponding to an irradiation energy of 5.38×10^{17} eV per second. This irradiation energy per unit time was then converted to the number of photons per second, assuming that the irradiation light consists exclusively of photons with 415 nm wavelength (see Supplementary Fig. 87 for the effective spectral emission profile of the used irradiation source), in which case each photon has the energy of 2.99 eV. This approximation yields a number of $\sim 1.80 \times 10^{17}$ photons per second, which reach the sample solutions. The absorbance of the samples for photocatalytic reactions were calculated according to the Beer–Lambert law, using the molar extinction coefficients of the photocatalysts at 415 nm ($\Delta\varepsilon_{415}$ = 11000 $M^{-1}cm^{-1}$ for $[Ru(bpz)_3]^{2+}$ and 4400 $M^{-1}cm^{-1}$ for $[Cr(dqp)_2]^{3+}$ in deuterated acetonitrile), their respective concentrations, and the averaged optical path length (3.6 mm) through the sample. Using the obtained absorbance values, the fraction of the transmitted photon flux to the irradiated photon flux can be calculated,[96] yielding the fraction of absorbed photon flux. Consequently, the photon flux N_{abs} absorbed by the photocatalysts can be calculated.

For photocatalytic reactions containing 500 µM $[Cr(dqp)_2]^{3+}$ or 200 µM $[Ru(bpz)_3]^{2+}$, the number of absorbed photons per second N_{abs} was estimated to be 1.51×10^{17}. For the reaction mixtures containing 200 µM $[Cr(dqp)_2]^{3+}$ or 80 µM $[Ru(bpz)_3]^{2+}$, an N_{abs} value of 0.93×10^{17} was determined. The uncertainty of this determination is estimated to 10%.

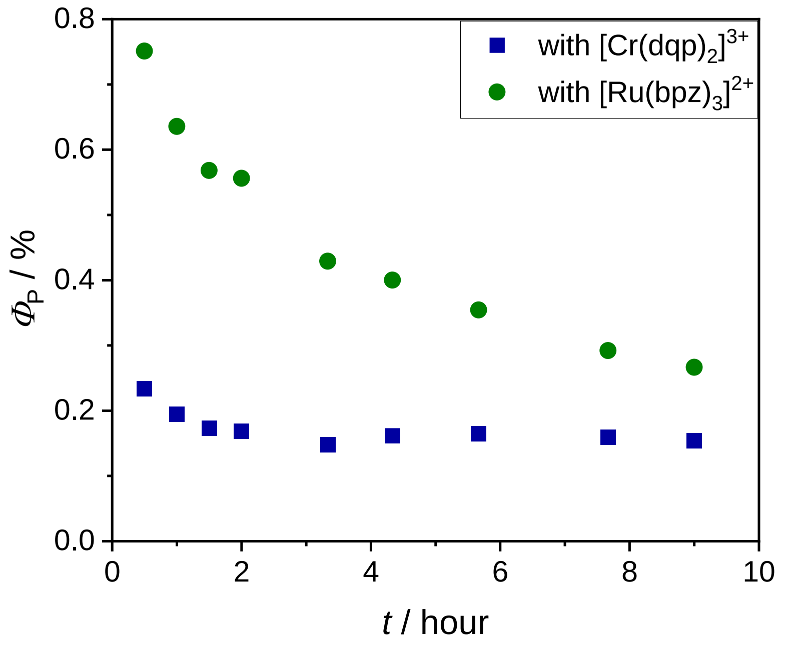

Supplementary Fig. 117: Quantum yield Φ_P for 4-methoxylphenol product formation in the aerobic hydroxylation of 4-methoxylphenylboronic acid catalyzed by $[Cr(dqp)_2]^{3+}$ (blue squares) and $[Ru(bpz)_3]^{2+}$ (green circles) as a function of irradiation time. Irradiation occurred with an LED at 415 nm providing a power density of 73 mW cm^{-2} at the sample position. The Φ_P values were determined on the basis of the ^{1}H-NMR data in Figures S94 and Supplementary Fig. 95. With increasing irradiation time, the Φ_P values lower, likely due to the combined effects of less and less substrate remaining and degradation of the photocatalysts.

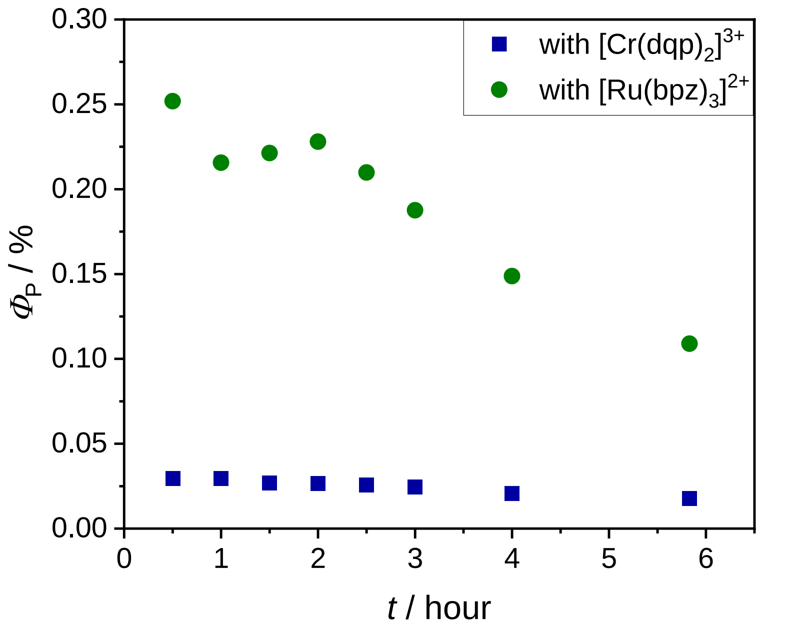

Supplementary Fig. 118*:* Quantum yield Φ_P for photocatalytic reductive debromination of 2-bromoacetophenone by $[Cr(dqp)_2]^{3+}$ (blue squares) and $[Ru(bpz)_3]^{2+}$ (green circles) as a function of irradiation time. Irradiation occurred with an LED at 415 nm providing a power density of 73 mW cm^{-2} at the sample position. The Φ_P values were determined on the basis of the ^{1}H-NMR data in Supplementary Fig. 101 and Supplementary Fig. 102. With increasing irradiation time, the Φ_P values lower, likely due to the combined effects of less and less substrate remaining and degradation of the photocatalysts.

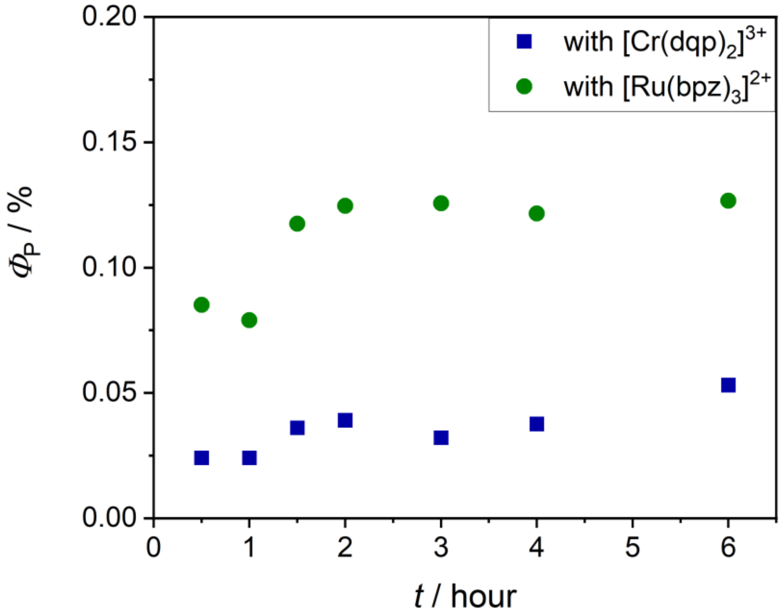

Supplementary Fig. 119: Quantum yield Φ_P for the photocatalytic Aza-Henry reaction catalyzed by $[Cr(dqp)_2]^{3+}$ (blue squares) and $[Ru(bpz)_3]^{2+}$ (green circles) as a function of irradiation time. Irradiation occurred with an LED at 415 nm providing a power density of 73 mW cm^{-2} at the sample position. The Φ_P values were determined on the basis of the ^{1}H-NMR data in Supplementary Fig. 110 and Supplementary Fig. 111. During the applied irradiation period, substrate consumption has no obvious effect on the determined Φ_P values. This can possibly be attributed to the formation of the THIQ-dimer and its subsequent decomposition into a THIQ radical and an iminium cation (Supplementary Fig. 108b, Supplementary Fig. 110), which can be further converted to the desired product. This reaction channel can potentially contribute substantially after extended irradiation periods.

At a given irradiation time, the Φ_P values obtained with $[Ru(bpz)_3]^{2+}$ are generally higher than those with $[Cr(dqp)_2]^{3+}$ for all three explored photocatalytic reactions. These differences, particularly within the initial irradiation hours, reflect the ratios of their initial reaction rates and cage escape quantum yields Φ_{CE}. These quantitative correlations between the cage escape quantum yields Φ_{CE}, the initial product formation rate, and the quantum yields Φ_P for photoproduct formation suggest that Φ_{CE} plays a decisive role for the overall reaction rates and efficiencies. Prolonged irradiation led to decreasing Φ_P values in some cases (Supplementary Fig. 117 and Supplementary Fig. 118), possibly due to substrate consumption and some photocatalyst degradation. Alternative reaction pathways, such as the formation of THIQ-dimer, its subsequent decomposition, and eventually conversion to product, might also affect the overall product formation quantum yields Φ_P over time (Supplementary Fig. 119).

10. NMR and mass spectra

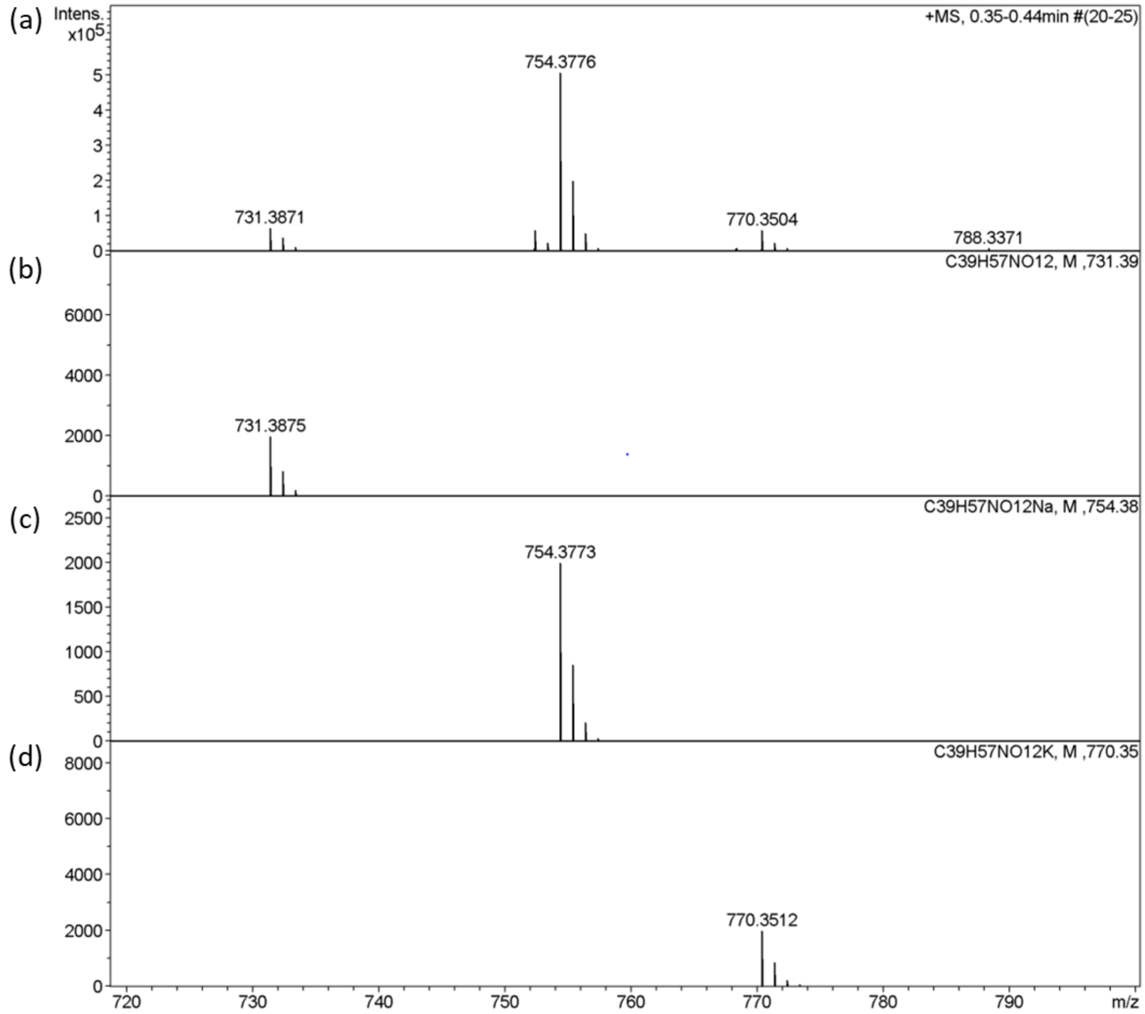

Supplementary Fig. 120: (a) ESI-HRMS (positive ions) mass spectrum of TAA-PEG$_3$ and simulated mass spectra of (b) TAA-PEG$_3$, (c) [TAA-PEG$_3$ + Na]$^+$ and (d) [TAA-PEG$_3$ + K]$^+$.

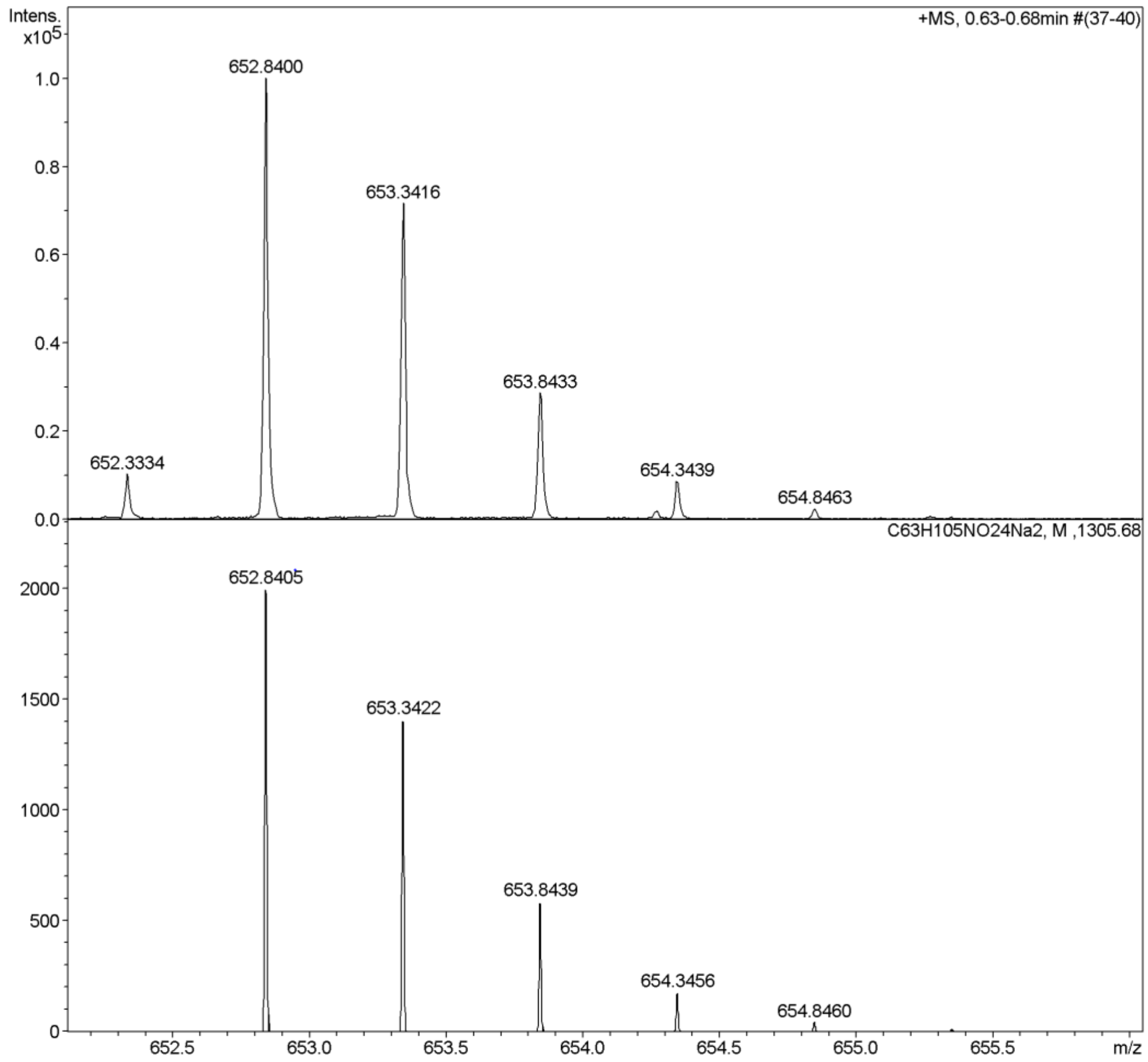

Supplementary Fig. 121: Top: ESI-HRMS (positive ions) mass spectrum of TAA-PEG$_7$. Bottom: simulated mass spectrum of [TAA-PEG$_7$ + 2Na]$^{2+}$.

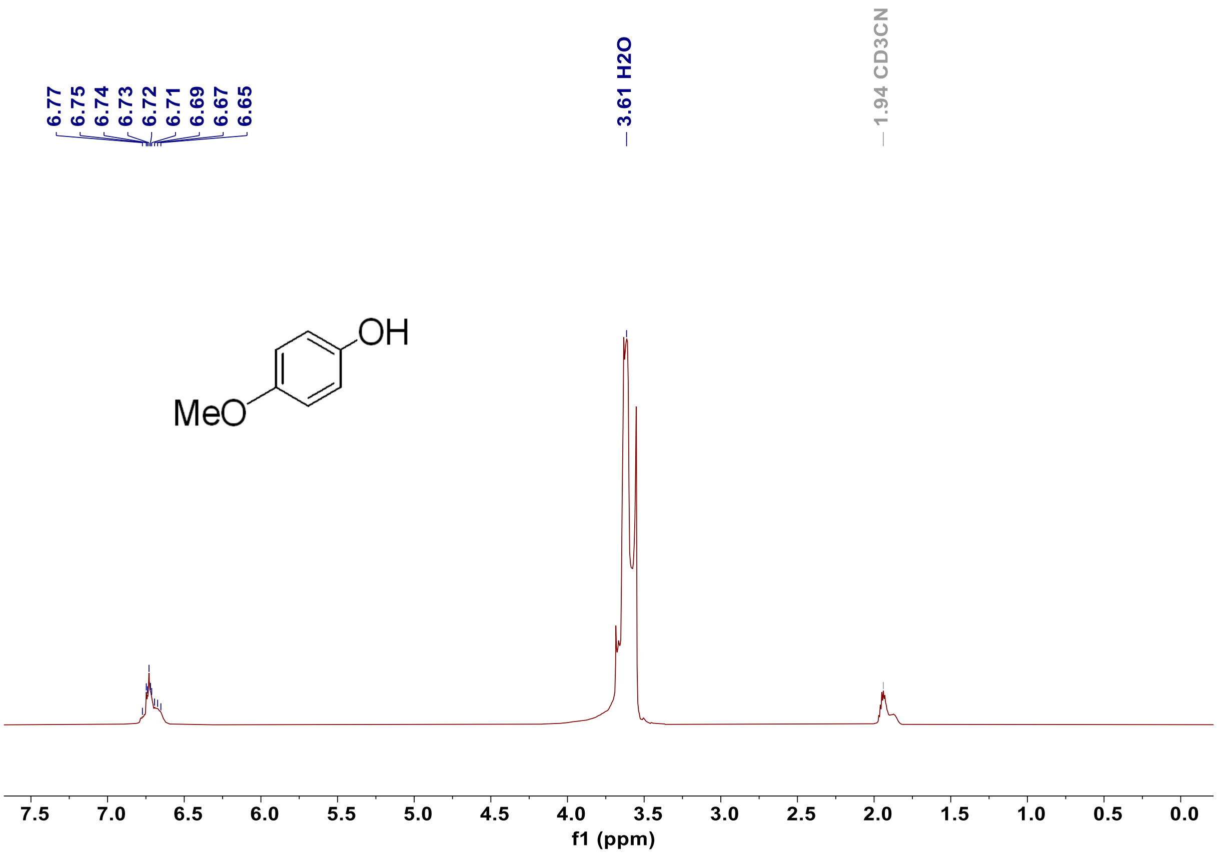

Supplementary Fig. 122: ^{1}H NMR spectrum of 4-methoxyphenol in CD_3CN/D_2O (4/1, v/v).

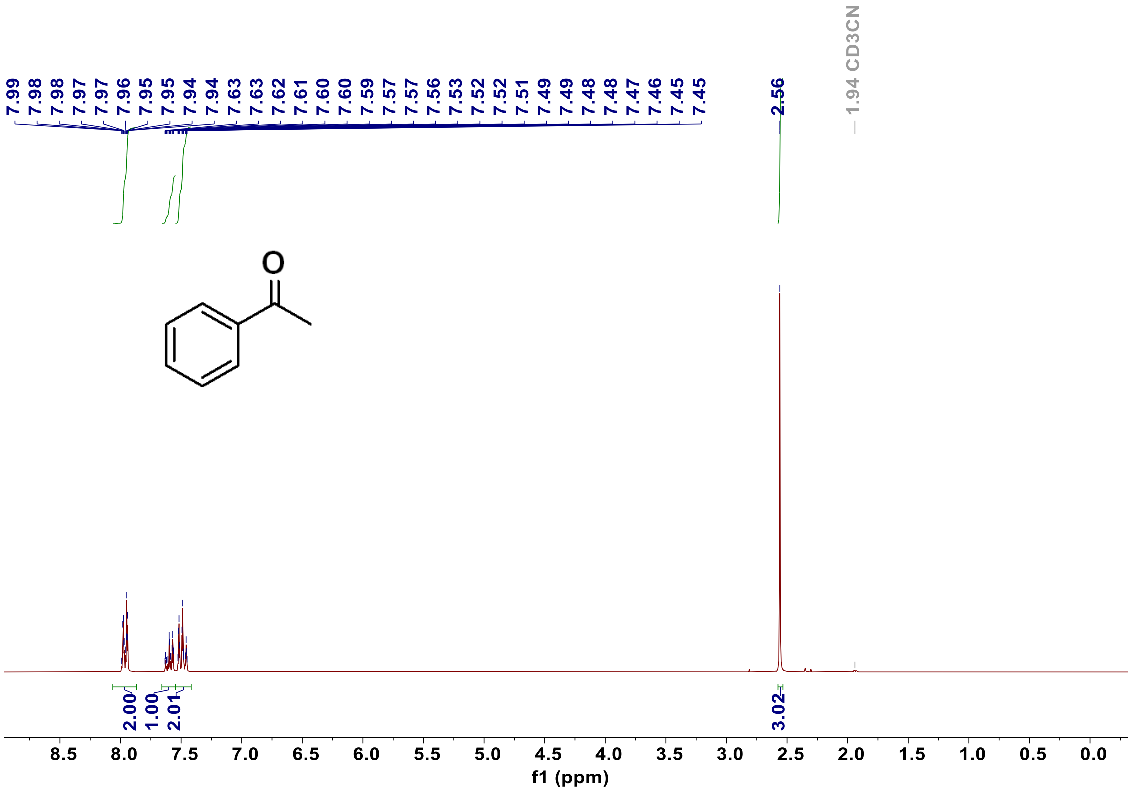

Supplementary Fig. 123: ^{1}H NMR spectrum of acetophenone in CD_3CN.

TAA-OH

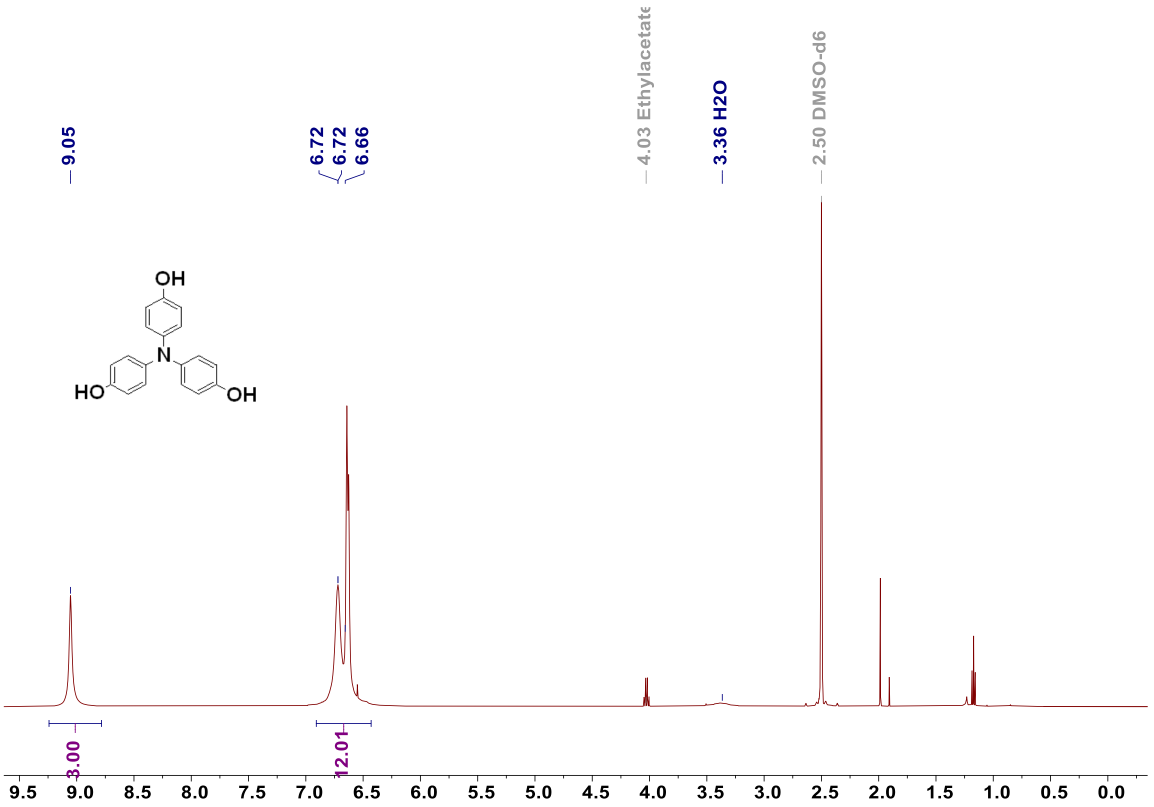

Supplementary Fig. 124: ^{1}H NMR spectrum of TAA-OH in DMSO-d6.

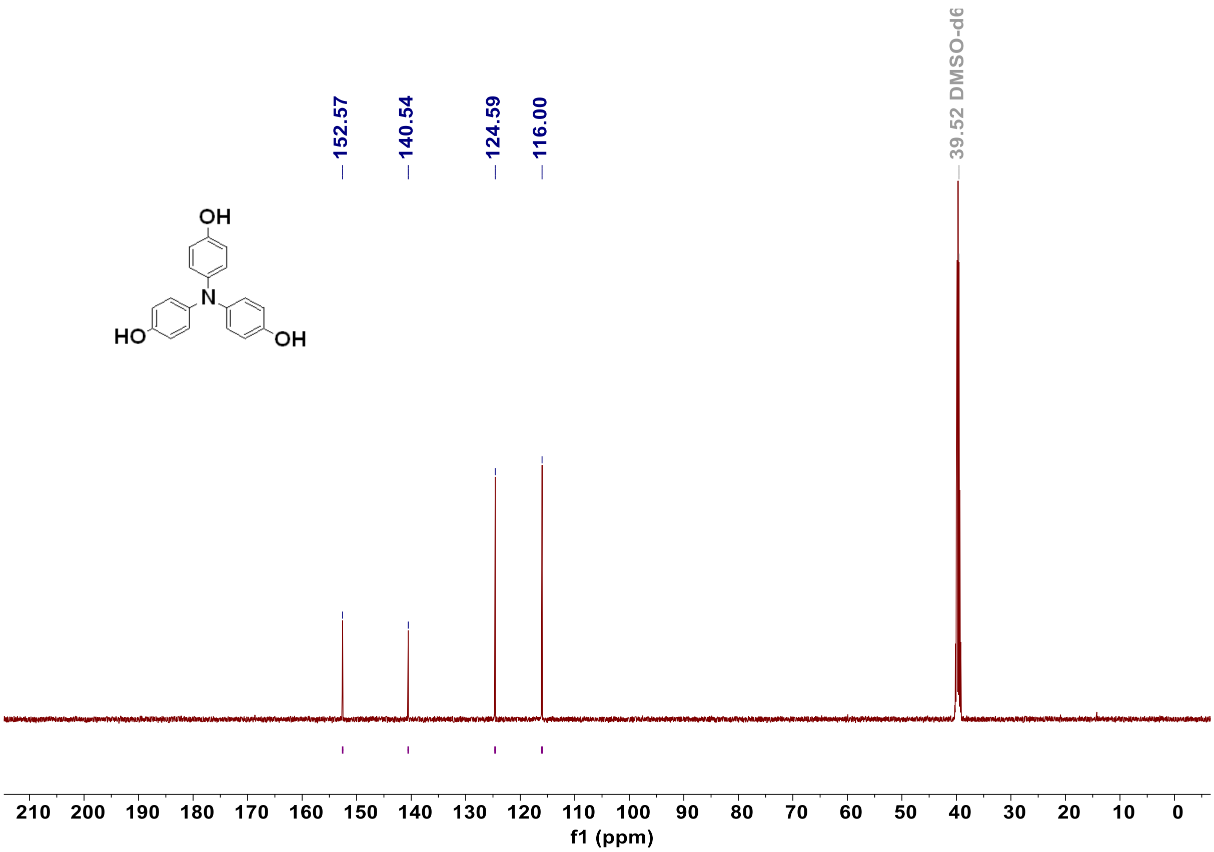

Supplementary Fig. 125: ^{13}C NMR spectrum of TAA-OH in DMSO-d6.

TAA-PEG$_3$

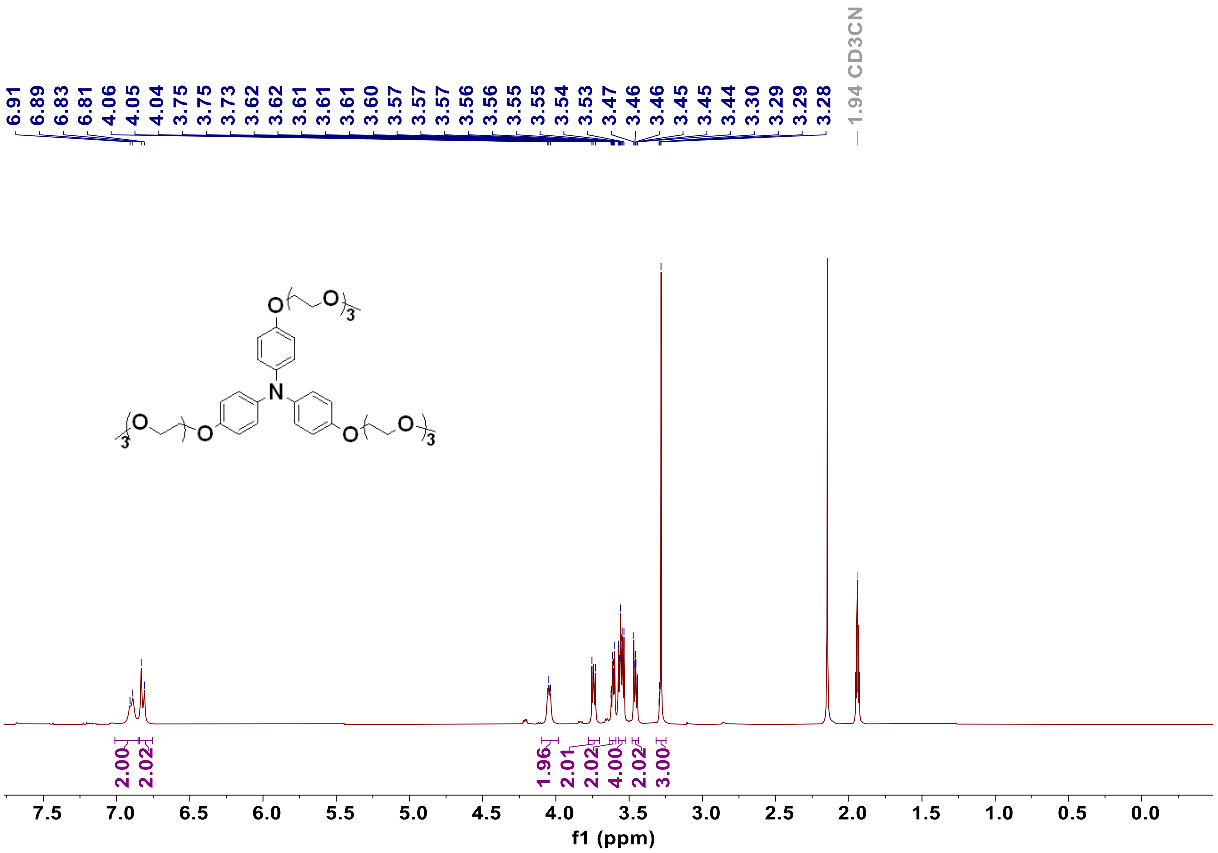

Supplementary Fig. 126: ^{1}H NMR spectrum of TAA-PEG$_3$ in CD_3CN.

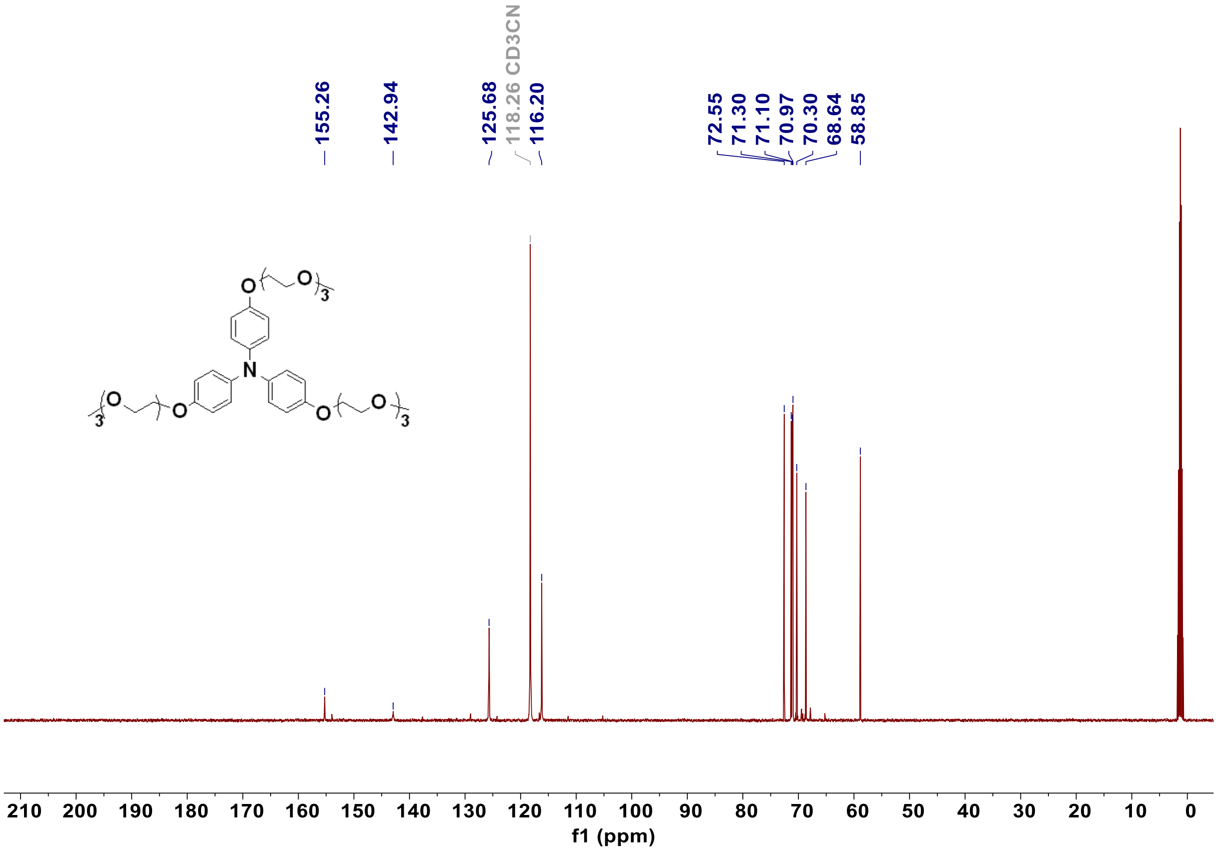

Supplementary Fig. 127: ^{13}C NMR spectrum of TAA-PEG$_3$ in CD_3CN.

PEG$_7$-OH

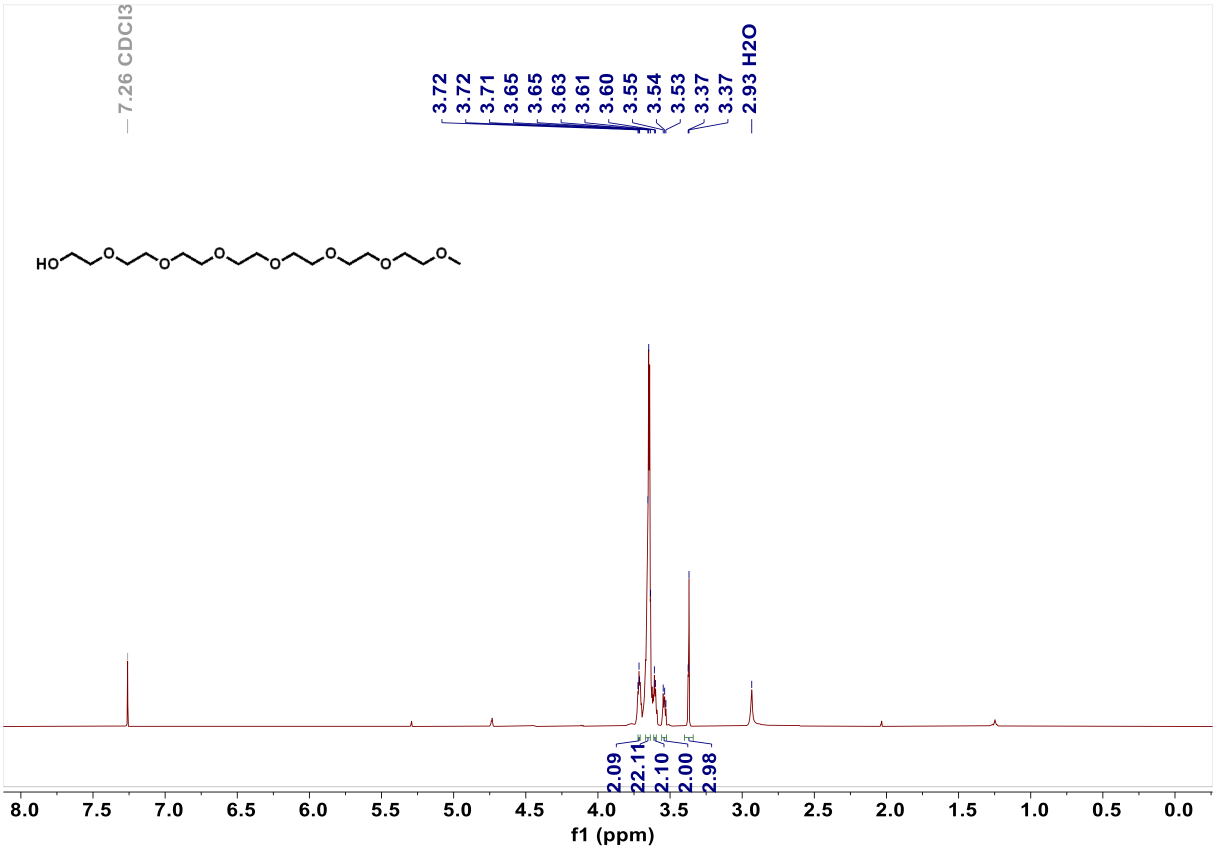

Supplementary Fig. 128: ^{1}H NMR spectrum of PEG$_7$-OH in $CDCl_3$.

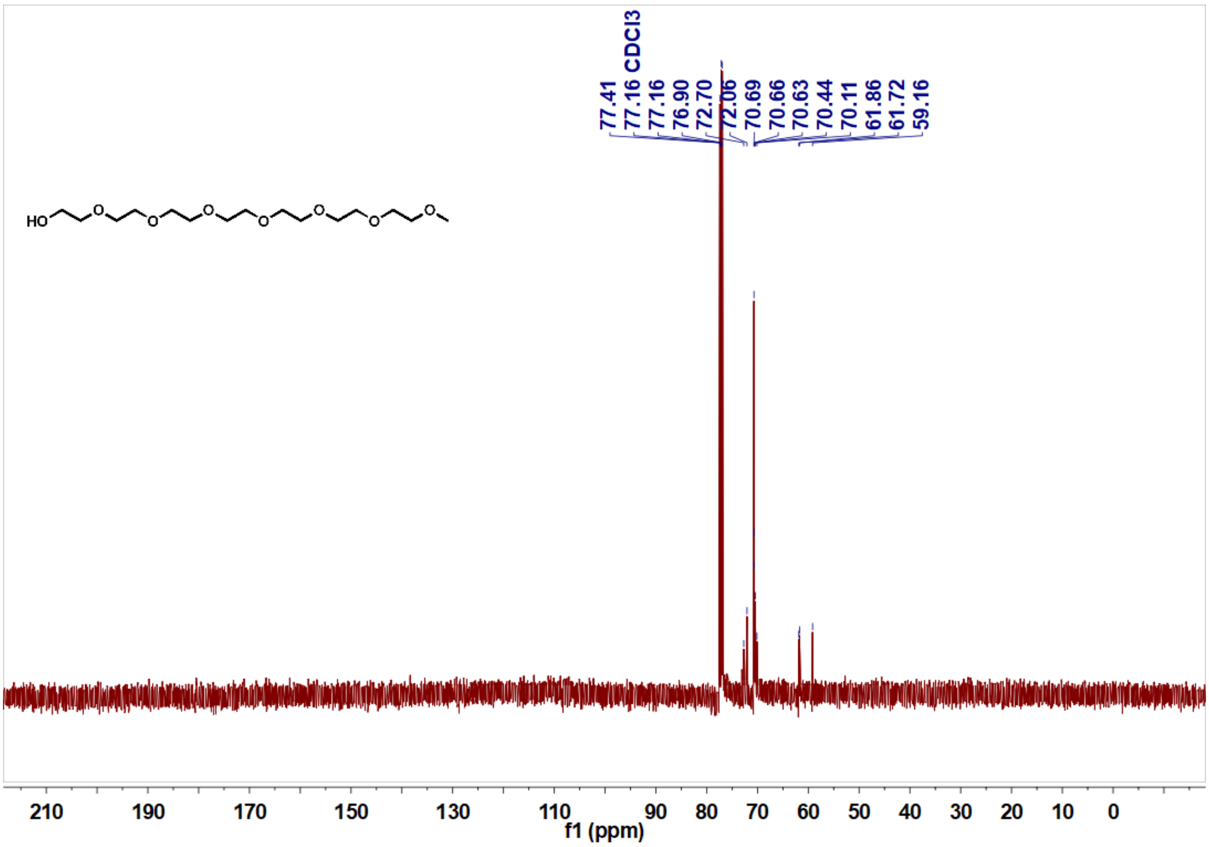

Supplementary Fig. 129: ^{13}C NMR spectrum of PEG$_7$-OH in $CDCl_3$.

PEG$_7$-Br

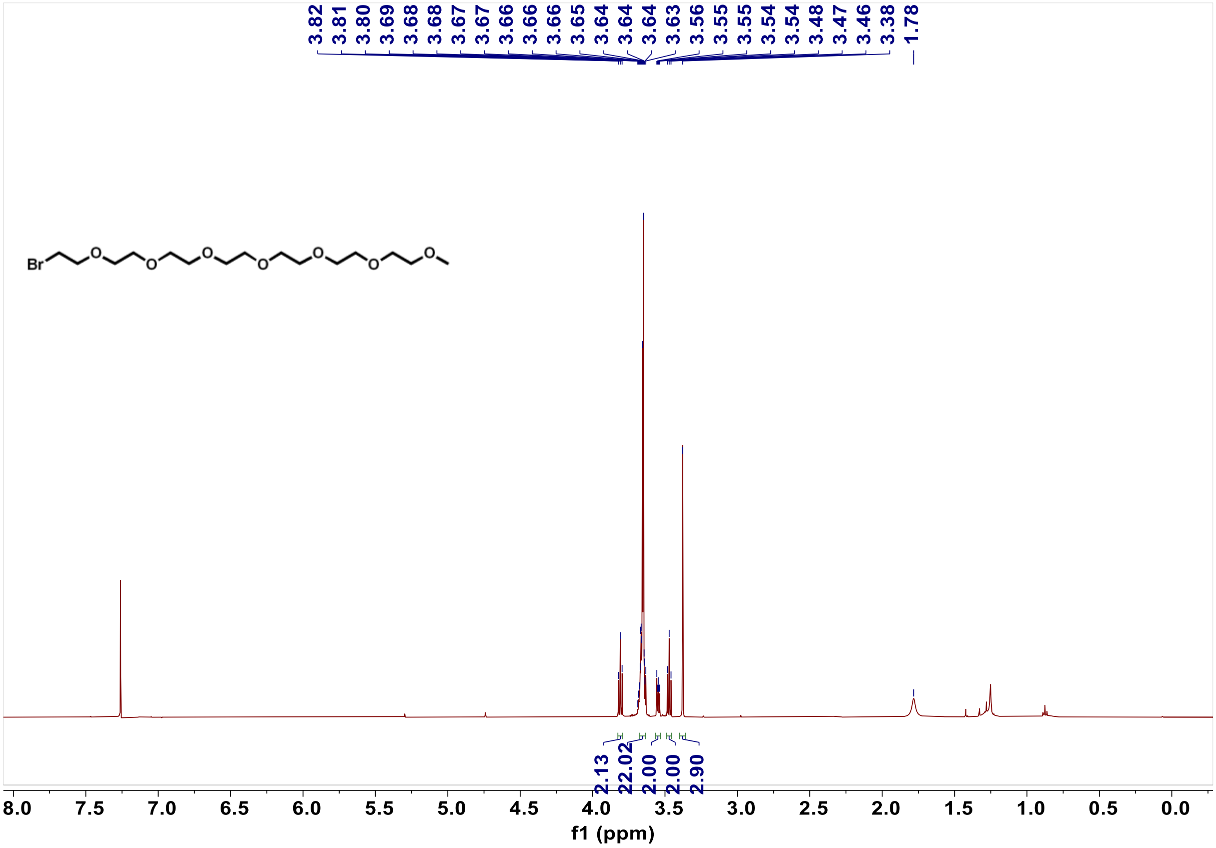

Supplementary Fig. 130: ^{1}H NMR spectrum of PEG$_7$-Br in $CDCl_3$.

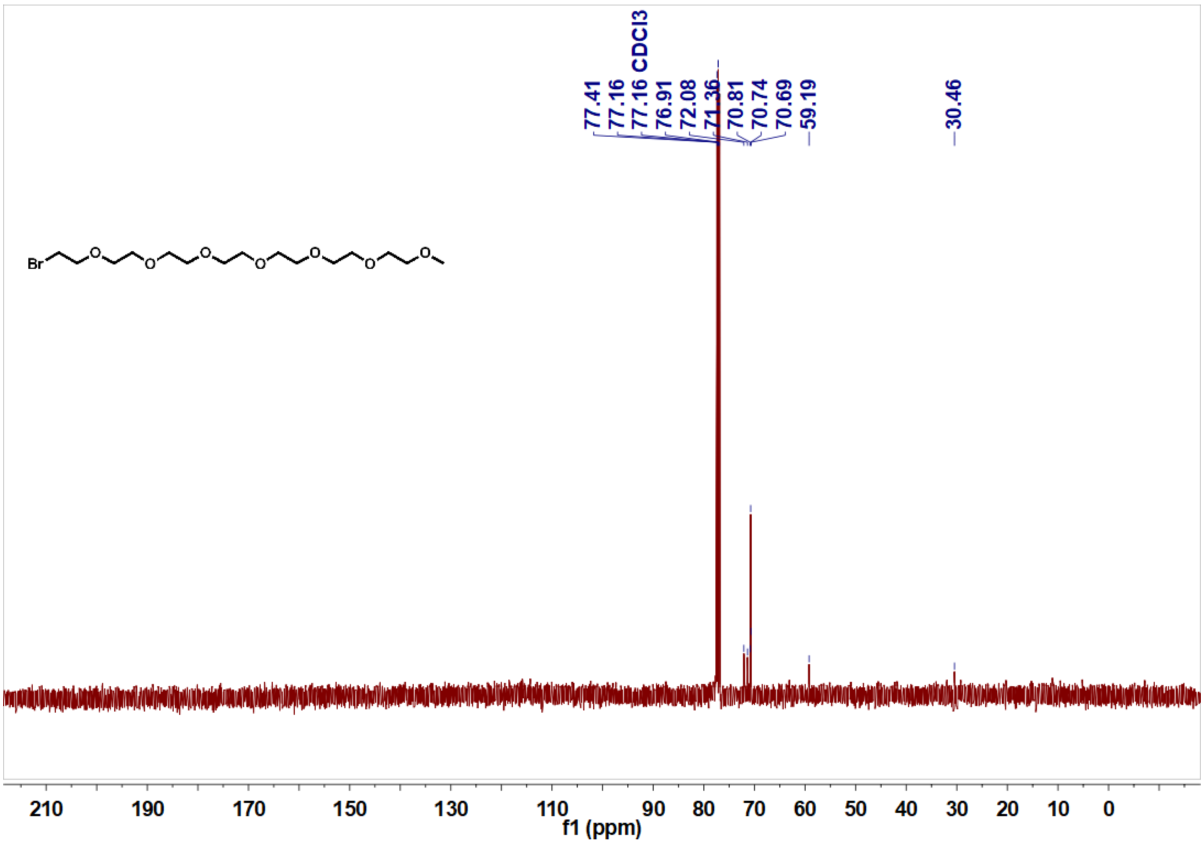

Supplementary Fig. 131: ^{1}H NMR spectrum of PEG$_7$-Br in $CDCl_3$.

TAA-PEG$_7$

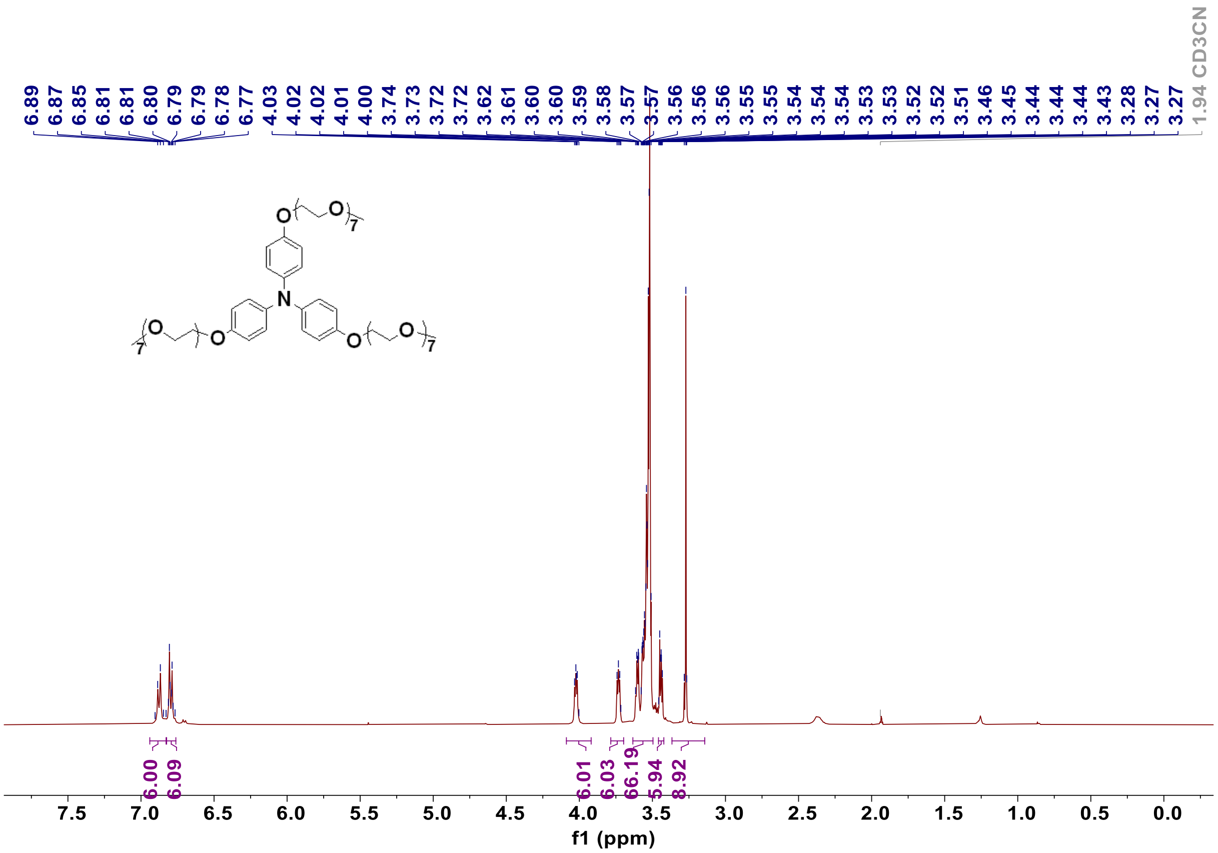

Supplementary Fig. 132: ^{1}H NMR spectrum of TAA-PEG$_7$ in CD_3CN.

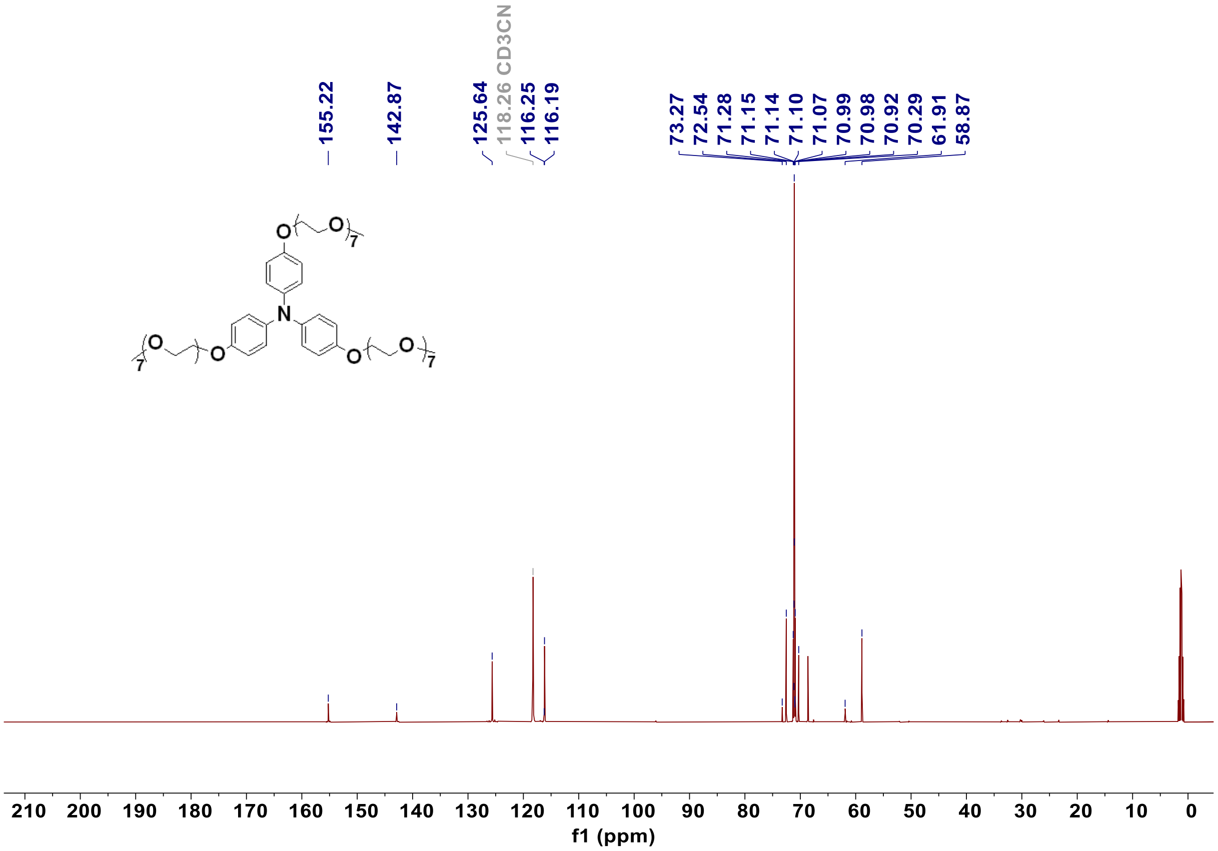

Supplementary Fig. 133: ^{13}C NMR spectrum of TAA-PEG$_7$ in CD_3CN.

11. References

1 Abrahamsson, M. *et al.* A 3.0 µs Room Temperature Excited State Lifetime of a Bistridentate Ru^{II}-Polypyridine Complex for Rod-like Molecular Arrays. *J. Am. Chem. Soc.* **128**, 12616–12617 (2006).

2 Cai, L. *et al.* Effects of solvent and base on the palladium-catalyzed amination: $PdCl_2(Ph_3P)_2/Ph_3P$-catalyzed selective arylation of primary anilines with aryl bromides. *Tetrahedron* **70**, 4754-4759 (2014).

3 Tlili, A., Monnier, F. & Taillefer, M. Selective one-pot synthesis of symmetrical and unsymmetrical di- and triarylamines with a ligandless copper catalytic system. *Chem. Commun.* **48**, 6408-6410 (2012).

4 Wahba, A. E. & Hamann, M. T. Reductive *N*-alkylation of nitroarenes: a green approach for the *N*-alkylation of natural products. *J. Org. Chem.* **77**, 4578-4585 (2012).

5 Forni, J. A., Brzozowski, M., Tsanaktsidis, J., Savage, G. P. & Polyzos, A. Rapid Microwave-Assisted Synthesis of *N*-Aryl 1,2,3,4-Tetrahydroisoquinolines. *Aust. J. Chem.* **68**, 1890-1893 (2015).

6 Bürgin, T. H., Glaser, F. & Wenger, O. S. Shedding Light on the Oxidizing Properties of Spin-Flip Excited States in a Cr^{III} Polypyridine Complex and Their Use in Photoredox Catalysis. *J. Am. Chem. Soc.* **144**, 14181-14194 (2022).

7 Gottlieb, H. E., Kotlyar, V. & Nudelman, A. NMR Chemical Shifts of Common Laboratory Solvents as Trace Impurities. *J. Org. Chem.* **62**, 7512-7515 (1997).

8 Zhu, Y., Li, S., Yang, X., Wang, S. & Zhang, Y. Direct synthesis of triphenylamine-based ordered mesoporous polymers for metal-free photocatalytic aerobic oxidation. *J. Mater. Chem. A* **10**, 13978-13986 (2022).

9 Chen, M. *et al.* Self-Organization and Vesicle Formation of Amphiphilic Fulleromonodendrons Bearing Oligo(poly(ethylene oxide)) Chains. *Langmuir* **32**, 2338-2347 (2016).

10 Stone, M. T. & Moore, J. S. A Water-Soluble *m*-Phenylene Ethynylene Foldamer. *Org. Lett.* **6**, 469-472 (2004).

11 Liu, X. *et al.* Monitoring Fluorinated Dendrimer-Based Self-Assembled Drug-Delivery Systems with ^{19}F Magnetic Resonance. *Eur. J. Org. Chem.* **2017**, 4461-4468 (2017).

12 Samanta, D., Sawoo, S. & Sarkar, A. In situ generation of gold nanoparticles on a protein surface: Fischer carbene complex as reducing agent. *Chem. Commun.*, 3438-3440 (2006).

13 Lu, Y. *et al.* The Critical Role of Dopant Cations in Electrical Conductivity and Thermoelectric Performance of n-Doped Polymers. *J. Am. Chem. Soc.* **142**, 15340-15348 (2020).

14 Aydogan, A. *et al.* Accessing Photoredox Transformations with an Iron(III) Photosensitizer and Green Light. *J. Am. Chem. Soc.* **143**, 15661-15673 (2021).

15 Zhong, J. J. *et al.* Cross-coupling hydrogen evolution reaction in homogeneous solution without noble metals. *Org. Lett.* **16**, 1988-1991 (2014).

16 Oyama, M., Goto, M. & Park, H. Apparent acid–base reaction between the *N*,*N*-dimethyl-*p*-toluidine cation radical and the neutral molecule in acetonitrile. *Electrochem. Commun.* **4**, 110–114 (2002).

17 Venturi, M., Mulazzani, Q. G., Ciano, M. & Hoffman, M. Z. Radiolytic and Electrochemical Reduction of $Ru(bpz)_3^{2+}$ in Aqueous Solution. Stability, Redox, and Acid-Base Properties of $Ru(bpz)_3^{+1}$. *Inorg. Chem.* **25**, 4493-4498 (1986).

18 Bronner, C. & Wenger, O. S. Kinetic Isotope Effects in Reductive Excited-State Quenching of $Ru(2,2'\text{-bipyrazine})_3^{2+}$ by Phenols. *J. Phys. Chem. Lett.* **3**, 70-74 (2011).

19 Sreenath, K., Thomas, T. G. & Gopidas, K. R. Cu(II) Mediated Generation and Spectroscopic Study of the Tris(4-anisyl)amine Radical Cation and Dication. Unusually Shielded Chemical Shifts in the Dication. *Org. Lett.* **13**, 1134–1137 (2011).

20 Connelly, N. G. & Geiger, W. E. Chemical Redox Agents for Organometallic Chemistry. *Chem. Rev.* **96**, 877–910 (1996).

21 DiMarco, B. N., Troian-Gautier, L., Sampaio, R. N. & Meyer, G. J. Dye-sensitized electron transfer from TiO_2 to oxidized triphenylamines that follows first-order kinetics. *Chem. Sci.* **9**, 940-949 (2018).

22 Hoekstra, R. M., Dibrell, M. M., Weaver, M. N., Nelsen, S. F. & Zink, J. I. Three-Chromophore Excited-State Mixed Valence. *J. Phys. Chem. A* **113**, 456–463 (2009).

23 Vardhaman, A. K. *et al.* Enhanced Electron Transfer Reactivity of a Nonheme Iron(IV)-Imido Complex as Compared to the Iron(IV)-Oxo Analogue. *Angew. Chem. Int. Ed.* **55**, 3709-3713 (2016).

24 Winter, A. H., Gibson, H. H. & Falvey, D. E. Carbazolyl Nitrenium Ion: Electron Configuration and Antiaromaticity Assessed by Laser Flash Photolysis, Trapping Rate Constants, Product Analysis, and Computational Studies. *J. Org. Chem.* **72**, 8186 - 8195 (2007).

25 Zador, E., Warman, J. M. & Hummel, A. Intermediates in the Nanosecond Pulse Radiolysis of Dimethylaniline Solutions in Cyclohexane *J. Chem. Soc., Faraday Trans. 1* **72**, 1368-1376 (1976).

26 Holcman, J. & Sehested, K. Dissociation of the OH Adduct of *N*,*N*-Dimethylaniline in Aqueous Solution. *J. Phys. Chem.* **81**, 20 (1997).

27 Rees, N. V., Klymenko, O. V., Compton, R. G. & Oyama, M. The electro-oxidation of *N*,*N* dimethyl-*p*-toluidine in acetonitrile: a microdisk voltammetry study. *J. Electroanal. Chem.* **531**, 33-42 (2002).

28 Kjær, K. S. *et al.* Luminescence and reactivity of a charge-transfer excited iron complex with nanosecond lifetime. *Science* **363**, 249–253 (2019).

29 Pohlers, G. & Scaiano, J. C. A Novel Photometric Method for the Determination of Photoacid Generation Efficiencies Using Benzothiazole and Xanthene Dyes as Acid Sensors. *Chem. Mater.* **9**, 3222-3230 (1997).

30 Neumann, S., Kerzig, C. & Wenger, O. S. Quantitative insights into charge-separated states from one- and two-pulse laser experiments relevant for artificial photosynthesis. *Chem. Sci.* **10**, 5624-5633 (2019).

31 Jones, R. W. *et al.* Direct Determination of the Rate of Intersystem Crossing in a Near-IR Luminescent Cr(III) Triazolyl Complex. *J. Am. Chem. Soc.* **145**, 12081-12092 (2023).

32 Juban, E. A. & McCusker, J. K. Ultrafast Dynamics of ^2E State Formation in $Cr(acac)_3$. *J. Am. Chem. Soc.* **127**, 6857–6865 (2005).

33 Zobel, J. P., Radatz, H. & González, L. Photodynamics of the Molecular Ruby $[Cr(ddpd)_2]^{3+}$. *Molecules* **28**, 1668 (2023).

34 Jiménez, J. R. *et al.* Chiral Molecular Ruby $[Cr(dqp)_2]^{3+}$ with Long-Lived Circularly Polarized Luminescence. *J. Am. Chem. Soc.* **141**, 13244-13252 (2019).

35 Wang, C. *et al.* Efficient Triplet-Triplet Annihilation Upconversion Sensitized by a Chromium(III) Complex via an Underexplored Energy Transfer Mechanism. *Angew. Chem. Int. Ed.* **61**, e202202238 (2022).

36 Sittel, S. *et al.* Visible-Light Induced Fixation of SO_2 into Organic Molecules with Polypyridine Chromium(III) Complexes. *ChemCatChem* **15**, e202201562 (2023).

37 Maestri, M. *et al.* Mechanism of the photochemistry and photophysics of the tris(2,2'-bipyridine)chromium(III) ion in aqueous solution. *J. Am. Chem. Soc.* **100**, 2694–2701 (1978).

38 Berera, R., van Grondelle, R. & Kennis, J. T. Ultrafast transient absorption spectroscopy: principles and application to photosynthetic systems. *Photosynth. Res.* **101**, 105-118 (2009).

39 Reynolds, R., Line, L. L. & Nelson, R. F. Electrochemical Generation of Carbazoles from Aromatic Amines. *J. Am. Chem. Soc.* **96**, 1087–1092 (1974).

40 Barham, J. P., John, M. P. & Murphy, J. A. Contra-thermodynamic Hydrogen Atom Abstraction in the Selective C-H Functionalization of Trialkylamine *N*-CH_3 Groups. *J. Am. Chem. Soc.* **138**, 15482-15487 (2016).

41 Montalti, M., Credi, A., Prodi, L. & Gandolfi, M. T. 495-497 (CRC Press, 2006).

42 Sun, H., Neshvad, G. & Hoffman, M. Z. Energy Gap Dependence of the Efficiency of Charge Separation upon the Sacrificial Reductive Quenching of the Excited States of Ru(II)-Diimine Photosensitizers in Aqueous Solution. *Mol. Cryst. Liq. Cryst.* **194**, 141-150 (2006).

43 DiLuzio, S., Connell, T. U., Mdluli, V., Kowalewski, J. F. & Bernhard, S. Understanding Ir(III) Photocatalyst Structure-Activity Relationships: A Highly Parallelized Study of Light-Driven Metal Reduction Processes. *J. Am. Chem. Soc.* **144**, 1431-1444 (2022).

44 Hoffman, M. Z. Cage Escape Yields from the Quenching of $*Ru(bpy)_3^{2+}$ by Methylviologen in Aqueous Solution. *J. Phys. Chem.* **92**, 3458–3464 (1988).

45 Gibbons, D. J., Farawar, A., Mazzella, P., Leroy-Lhez, S. & Williams, R. M. Making triplets from photo-generated charges: observations, mechanisms and theory. *Photochem. Photobiol. Sci.* **19**, 136-158 (2020).

46 Olmsted III, J. & Meyer, T. J. Factors affecting cage escape yields following electron-transfer quenching. *J. Phys. Chem.* **91**, 1649–1655 (1987).

47 Kikuchi, K., Hoshi, M., Niwa, T., Takahashi, Y. & Miyashi, T. Heavy-atom effects on the excited singlet-state electron-transfer reaction. *J. Phys. Chem.* **95**, 38–42 (1991).

48 Wolff, H.-J., Bürßner, D. & Steiner, U. E. Spin-orbit coupling controlled spin chemistry of $Ru(bpy)_3^{2+}$ photooxidation: Detection of strong viscosity dependence of in-cage backward electron transfer rate. *Pure Appl. Chem.* **67**, 167-174 (1995).

49 Jayanthi, S. & Ramamurthy, P. Photoinduced electron transfer reactions of 2,4,6 triphenylpyrylium: solvent effect and charge-shift type of systems. *Phys. Chem. Chem. Phys.* **1**, 4751-4757 (1999).

50 Meidlar, K. & Das, P. K. Tris(2,2'-bipyridine)ruthenium(II)-Sensitized Photooxidation of Phenols. Environmental Effects on Electron Transfer Yields and Kinetics. *J. Am. Chem. Soc.* **104**, 7462-7469 (1982).

51 Ohno, T. & Lichtin, N. N. Electron Transfer in the Quenching of Triplet Methylene Blue by Complexes of Iron(II). *J. Am. Chem. Soc.* **102**, 4636-4643 (1980).

52 Gould, I. R., Ege, D., Moser, J. E. & Farid, S. Efficiencies of Photoinduced Electron-Transfer Reactions: Role of the Marcus Inverted Region in Return Electron Transfer within Geminate Radical-Ion Pairs. *J. Am. Chem. Soc.* **112**, 4290-4301 (1990).

53 Kalyanasundaram, K. & Neumann-Spallart, M. Influence of added salts on the cage escape yields in the photoredox quenching of $Ru(bpy)_2^{+3}$ excited states. *Chem. Phys. Lett.* **88**, 7-12 (1982).

54 Delaire, J. A. & Sanquer-Barrie, M. Role of Electrostatic Interaction in Light-Induced Charge Separation in Polyelectrolyte Bound Vinyldiphenylanthracene. *J. Phys. Chem.* **92**, 1252-1257 (1988).

55 Das, P. K., Encinas, V. & Scaiano, J. C. Laser flash photolysis study of the reactions of carbonyl triplets with phenols and photochemistry of *p*-hydroxypropiophenone. *J. Am. Chem. Soc.* **103**, 4154-4162 (1981).

56 Rehm, D. & Weller, A. Kinetik und Mechanismus der Elektronübertragung bei der Fluoreszenzlöschung in Acetonitril. *Ber. Bunsen-Ges. Phys. Chem.* **73**, 834– 839 (1969).

57 Rehm, D. & Weller, A. Kinetics of Fluorescence Quenching by Electron and H-Atom Transfer. *Isr. J. Chem.* **8**, 259– 271 (1970).

58 Shioyama, H., Masuhara, H. & Malaga, N. Radical yield in electron transfer quenching of the excited tris(2,2'-bipyridine)ruthenium(II) complex. *Chem. Phys. Lett.* **88**, 161-165 (1982).

59 Hore, P. J. & Mouritsen, H. The Radical-Pair Mechanism of Magnetoreception. *Annu. Rev. Biophys.* **45**, 299-344 (2016).

60 Kavarnos, G. J. T., Nicholas J. . Photosensitization by Reversible Electron Transfer: Theories, Experimental Evidence, and Examples. *Chem. Rev.* **86**, 401–449 (1986).

61 Prasad, D. R., Hessler, D., Hoffman, Z. & Serpone, N. Quantum yield of formation of methylviologen radical cation from the photolysis of the $Ru(bpz)_3^{2+}$/methylviologen/EDTA system. *Chem. Phys. Lett.* **121** (1985).

62 Crutchley, R. J. & Lever, A. B. P. Ruthenium(II) Tris(bipyrazyl) Dication - A New Photocatalyst. *J. Am. Chem. Soc.* **102**, 7128–7129 (1980).
63 Otto, S., Scholz, N., Behnke, T., Resch-Genger, U. & Heinze, K. Thermo-Chromium: A Contactless Optical Molecular Thermometer. *Chem. Eur. J.* **23**, 12131–12135 (2017).
64 Wang, C., Otto, S., Dorn, M., Heinze, K. & Resch-Genger, U. Luminescent TOP Nanosensors for Simultaneously Measuring Temperature, Oxygen, and pH at a Single Excitation Wavelength. *Anal. Chem.* **91**, 2337–2344 (2019).
65 Scarborough, C. C., Sproules, S., Weyhermüller, T., DeBeer, S. & Wieghardt, K. Electronic and molecular structures of the members of the electron transfer series $[Cr(^{t}bpy)_3]^n$ (n = 3+, 2+, 1+, 0): an X-ray absorption spectroscopic and density functional theoretical study. *Inorg. Chem.* **50**, 12446-12462 (2011).
66 Scarborough, C. C. *et al.* Experimental fingerprints for redox-active terpyridine in $[Cr(tpy)_2](PF_6)_n$ (n = 3-0), and the remarkable electronic structure of $[Cr(tpy)_2]^{1-}$. *Inorg. Chem.* **51**, 3718-3732 (2012).
67 El-Sayed, M. A. The Triplet State: Its Radiative and Nonradiative Properties. *Acc. Chem. Res.* **1**, 8–16 (1968).
68 Yu-Tzu Li, E., Jiang, T. Y., Chi, Y. & Chou, P. T. Semi-quantitative assessment of the intersystem crossing rate: an extension of the El-Sayed rule to the emissive transition metal complexes. *Phys. Chem. Chem. Phys.* **16**, 26184-26192 (2014).
69 Lucia, L. A. & Schanze, K. S. Cage escape yields for photoinduced bimolecular electron transfer reactions of Re(I) complexes *Inorg. Chim. Acta* **225**, 41-49 (1994).
70 Chai, J., Lashgari, A., Wang, X., Williams, C. K. & Jiang, J. J. All-PEGylated redox-active metal-free organic molecules in non-aqueous redox flow battery. *J. Mater. Chem. A* **8**, 15715-15724 (2020).
71 Hotchen, C. E. *et al.* Amplified electron transfer at poly-ethylene-glycol (PEG) grafted electrodes. *Phys. Chem. Chem. Phys.* **17**, 11260-11268 (2015).
72 Zou, Y. Q. *et al.* Highly efficient aerobic oxidative hydroxylation of arylboronic acids: photoredox catalysis using visible light. *Angew. Chem. Int. Ed.* **51**, 784-788 (2012).
73 Pitre, S. P., McTiernan, C. D., Ismaili, H. & Scaiano, J. C. Mechanistic insights and kinetic analysis for the oxidative hydroxylation of arylboronic acids by visible light photoredox catalysis: a metal-free alternative. *J. Am. Chem. Soc.* **135**, 13286-13289 (2013).
74 Xie, H. Y. *et al.* N-Substituted 3(10*H*)-Acridones as Visible-Light, Water-Soluble Photocatalysts: Aerobic Oxidative Hydroxylation of Arylboronic Acids. *J. Org. Chem.* **82**, 5236-5241 (2017).
75 Sittel, S., Naumann, R. & Heinze, K. Molecular Rubies in Photoredox Catalysis. *Front. Chem.* **10**, 887439 (2022).
76 Narayanam, J. M. R., Tucker, J. W. & Stephenson, C. R. J. Electron-Transfer Photoredox Catalysis: Development of a Tin-Free Reductive Dehalogenation Reaction. *J. Am. Chem. Soc.* **131**, 8756-8757 (2009).
77 Nicewicz, D. A. & MacMillan, D. W. C. Merging Photoredox Catalysis with Organocatalysis: The Direct Asymmetric Alkylation of Aldehydes. *Science* **322**, 77-80 (2008).
78 Jung, J., Kim, J., Park, G., You, Y. & Cho, E. J. Selective Debromination and α-Hydroxylation of α-Bromo Ketones Using Hantzsch Esters as Photoreductants. *Adv. Synth. Catal.* **358**, 74-80 (2016).
79 Katritzky, A. R., Lang, H. & Lan, X. Novel Routes to 4-Substituted N,N-Dialkylanilines, N-Alkylanilines and Anilines *Tetrahedron* **49**, 1445-1454 (1993).
80 Cao, X. *et al.* Engineering Lattice Disorder on a Photocatalyst: Photochromic BiOBr Nanosheets Enhance Activation of Aromatic C-H Bonds via Water Oxidation. *J. Am. Chem. Soc.* **144**, 3386-3397 (2022).
81 Shi, H. *et al.* Lewis Acid Assisted Electrophilic Fluorine-Catalyzed Pinacol Rearrangement of Hydrobenzoin Substrates: One-Pot Synthesis of (±)-Latifine and (±)-Cherylline. *J. Org. Chem.* **83**, 1312-1319 (2018).

82 Maji, T., Karmakar, A. & Reiser, O. Visible-Light Photoredox Catalysis: Dehalogenation of Vicinal Dibromo-, α-Halo-, and α,α-Dibromocarbonyl Compounds. *J. Org. Chem.* **76**, 736-739 (2011).
83 Neumann, M., Füldner, S., König, B. & Zeitler, K. Metal-free, cooperative asymmetric organophotoredox catalysis with visible light. *Angew. Chem. Int. Ed.* **50**, 951-954 (2011).
84 Wang, Z. J., Ghasimi, S., Landfester, K. & Zhang, K. A. I. A conjugated porous poly-benzobisthiadiazole network for a visible light-driven photoredox reaction. *J. Mater. Chem. A* **2**, 18720-18724 (2014).
85 Luo, J., Zhang, X. & Zhang, J. Carbazolic Porous Organic Framework as an Efficient, Metal-Free Visible-Light Photocatalyst for Organic Synthesis. *ACS Catal.* **5**, 2250-2254 (2015).
86 Zhu, X., Lin, Y., Sun, Y., Beard, M. C. & Yan, Y. Lead-Halide Perovskites for Photocatalytic α-Alkylation of Aldehydes. *J. Am. Chem. Soc.* **141**, 733-738 (2019).
87 Zhang, J., Chen, J. R., Chen, J. R., Luo, Y. & Xia, Y. Solvent as photoreductant for dehalogenation of α-haloketones under catalyst-free conditions. *Tetrahedron Lett.* **98**, 153835 (2022).
88 Mcgimpsey, W. & Scaiano, J. Photochemistry of α-chloro- and α-bromoacetophenone. Determination of extinction coefficients for halogen–benzene complexes. *Can. J. Chem.* **66**, 1474-1478 (1988).
89 Dorrestijn, E. *et al.* The Reduction of α-X-Acetophenones (X = PhO, Br, Cl) in Hydrogen-Donating Solvents at Elevated Temperatures. *Eur. J. Org. Chem.* **1999**, 607-616 (1999).
90 Glaser, F., Kerzig, C. & Wenger, O. S. Sensitization-initiated electron transfer via upconversion: mechanism and photocatalytic applications. *Chem. Sci.* **12**, 9922-9933 (2021).
91 Beckett, A., Osborne, A. D. & Porter, G. Primary photochemical processes in aromatic molecules. Part 11.—Radicals and radical anions derived from benzaldehyde, acetophenone and benzil. *Trans. Faraday Soc.* **60**, 873-881 (1964).
92 Condie, A. G., González-Gómez, J. C. & Stephenson, C. R. J. Visible-Light Photoredox Catalysis: Aza-Henry Reactions via C-H Functionalization. *J. Am. Chem. Soc.* **132**, 1464–1465 (2010).
93 Bartling, H., Eisenhofer, A., König, B. & Gschwind, R. M. The Photocatalyzed Aza-Henry Reaction of *N*-Aryltetrahydroisoquinolines: Comprehensive Mechanism, $H^{\bullet}$ - versus H^{+}-Abstraction, and Background Reactions. *J. Am. Chem. Soc.* **138**, 11860-11871 (2016).
94 Serpone, N. Relative photonic efficiencies and quantum yields in heterogeneous photocatalysis. *J. Photochem. Photobiol. A: Chem.* **104**, 1-12 (1997).
95 Pope, R. M. & Fry, E. S. Absorption spectrum (380–700 nm) of pure water. II. Integrating cavity measurements. *Appl. Opt.* **36**, 8710-8723 (1997).
96 McNaught, A. D. & Wilkinson, A. *IUPAC Compendium of Chemical Terminology (The "Gold Book")*. 2nd edn, (Blackwell Scientific Publications, 1997).
